# Plant protein peptidase inhibitors: an evolutionary overview based on comparative genomics

**DOI:** 10.1186/1471-2164-15-812

**Published:** 2014-09-25

**Authors:** María Estrella Santamaría, Mercedes Diaz-Mendoza, Isabel Diaz, Manuel Martinez

**Affiliations:** Centro de Biotecnología y Genómica de Plantas, Universidad Politécnica de Madrid, Campus Montegancedo, Pozuelo de Alarcón, Madrid, 28223 Spain

**Keywords:** Comparative genomics, Peptidase inhibitors, Plant evolution, Protein families

## Abstract

**Background:**

Peptidases are key proteins involved in essential plant physiological processes. Although protein peptidase inhibitors are essential molecules that modulate peptidase activity, their global presence in different plant species remains still unknown. Comparative genomic analyses are powerful tools to get advanced knowledge into the presence and evolution of both, peptidases and their inhibitors across the Viridiplantae kingdom.

**Results:**

A genomic comparative analysis of peptidase inhibitors and several groups of peptidases in representative species of different plant taxonomic groups has been performed. The results point out: i) clade-specific presence is common to many families of peptidase inhibitors, being some families present in most land plants; ii) variability is a widespread feature for peptidase inhibitory families, with abundant species-specific (or clade-specific) gene family proliferations; iii) peptidases are more conserved in different plant clades, being C1A papain and S8 subtilisin families present in all species analyzed; and iv) a moderate correlation among peptidases and their inhibitors suggests that inhibitors proliferated to control both endogenous and exogenous peptidases.

**Conclusions:**

Comparative genomics has provided valuable insights on plant peptidase inhibitor families and could explain the evolutionary reasons that lead to the current variable repertoire of peptidase inhibitors in specific plant clades.

**Electronic supplementary material:**

The online version of this article (doi:10.1186/1471-2164-15-812) contains supplementary material, which is available to authorized users.

## Background

Proteolysis is a ubiquitous mechanism required to maintain the life cycle in all known organisms. Degrading and recycling of proteins are crucial events to control protein functionality and to achieve that proteins act in correct spatial and temporal locations. In plants, peptidases are key players in numerous physiological processes [1, 2]. During plant development they are involved in the regulation of protein functionality and the breakdown of storage compounds in the seed and other plant tissues [3–5]. In relation with biotic and abiotic stresses, they are taking part in the regulation of both endogenous and exogenous proteins to fight against these natural plant stresses [6, 7]. As proteolysis is an irreversible mechanism, peptidases must be precisely controlled. Peptidase activity may be regulated at the transcriptional and translational levels, but the most important control is achieved at the protein level. Peptidase inhibitors are proteinaceous molecules that exert their action by regulating peptidase activity. In plant development, peptidase inhibitors are involved in the same physiological processes than the peptidases they control [8–10]. As defence proteins, they are inhibiting peptidases from the pests and pathogens that attack the plant [11, 12].

The MEROPS database is dedicated to the analyses of peptidase and peptidase inhibitors [13]. In this database both peptidases and their inhibitors are classified into clans based on structural similarity or sequence features. All members of a clan share a similar protein fold. Clans are divided in families based on common ancestry. All the members of a family are homologous proteins. At present, 75 peptidase inhibitor families are compiled in the database.

The unique study focused on the different peptidase inhibitor families that exist in a specific life clade has been performed on the prokaryotes kingdom [14]. In plants, several peptidase inhibitor families such as I4 Serpins (MEROPS family identifier and common name), I13 Potato type I (Pin-I), I25 cystatins or I20 Potato type II (Pin-II) have been already reviewed [4, 9, 15, 16]. However, an evolutionary and global analysis of the different inhibitor families in different plant species has still not been performed. The field of genomics has been conveniently developed in last years and numerous tools have arisen to deal with the enormous number of sequences deposited in the databases. Nowadays, a great number of plant genomes have been sequenced and annotated, including species from basal taxonomic groups [17]. These genomic sequences have been included in several comparative genomic programs, such as Phytozome, PLAZA or GreenPhylDB [18–20], simplifying the process to extract and compare information on the family members coming from different plant species [17, 21]. Using these strong last generation tools, the evolutionary features regarding the distribution of protein peptidase inhibitors in the plant kingdom have been analyzed in this work.

## Results

### Protein peptidase inhibitors families in plants

To get the complete number of protein peptidase inhibitors in plants, several species were selected. The genomes of these species have been completely sequenced and annotated, and drafts of these sequences are available on the web. These species were: fifteen eudicots (*Ricinus communis* [22], *Populus trichocarpa* [23], *Medicago truncatula* [24], *Glycine max* [25], *Cucumis sativus* [26], *Prunus persica* [27], *Fragaria vesca* [28], *Arabidopsis thaliana* [29, 30], *Carica papaya* [31], *Theobroma cacao* [32], *Vitis vinifera* [33], *Mimulus guttatus* [34]), four monocots (*Sorghum bicolor* [35], *Zea mays* [36], *Oryza sativa* [37, 38], *Brachypodium distachyon* [39]), one pseudofern (*Selaginella moellendorffii* [40]), one moss (*Physcomitrella patens* [41, 42]), and five algae (*Chlamydomonas reinhardtii* [43], *Volvox carteri* [44], *Coccomyxa subellipsoidea* [45], *Micromonas pusilla* [46], *Ostreococcus lucimarinus* [47]). All the genomes of these plant species are accessible at Phytozome comparative genomics database, and most of them also at GreenPhylDB comparative genomics database. Gene prediction quality varies among the annotation stage of the different genomes and the gene family distribution and size could slightly be modified when new annotation versions will be released.

Firstly, the protein peptidase inhibitor families present in each plant species were determined. For that, their kingdom distribution was analyzed in MEROPS database. Using the information present in MEROPS database and after searches in the genomes of the selected plant species, twenty-one families with members described in plants were identified. Table 1 shows the global distribution of these families in the plant kingdom. Several peptidase inhibitor families are conserved in most clades of Viridiplantae (families I1, I3, I4, I6, I9, I12, I13, I20, I25, I29, I51) whereas some others are restricted to one specific clade or even to a specific group of species inside a clade (families I2, I7, I18, I37, I39, I55, I67, I73, I83, I90).

**Table 1:** **Distribution of protein peptidase inhibitor families in the Viridiplantae**

Family	Distribution
I1 Kazal	Chlorophyta, Bryophyta, Lycopodiophyta, Monocots, Eudicots
I2 Kunitz-A	Chlorophyta (Chlorophyceae, Trebouxiophyceae)
I3 Kunitz-P	Chlorophyta, Bryophyta, Lycopodiophyta, Monocots, Eudicots
I4 Serpin	Chlorophyta, Bryophyta, Lycopodiophyta, Monocots, Eudicots
I6 Cereal	Monocots, Eudicots
I7 Squash serine	Eudicots (Cucurbitales)
I9 Subtilisin propeptide	Chlorophyta, Bryophyta, Lycopodiophyta, Monocots, Eudicots
I12 Bowman-Birk	Monocots, Eudicots
I13 Pin-I	Chlorophyta, Bryophyta, Lycopodiophyta, Monocots, Eudicots
I18 MTI-2	Eudicots (Brassicales)
I20 Pin-II	Lycopodiophyta, Monocots, Eudicots
I25 Cystatin	Chlorophyta, Bryophyta, Lycopodiophyta, Monocots, Eudicots
I29 Papain propeptide	Chlorophyta, Bryophyta, Lycopodiophyta, Monocots, Eudicots
I37 PMCPI	Eudicots (Solanales)
I39 Alpha-2 macroglobulin	Chlorophyta (Mamiellophyceae)
I51 SCPY Inhibitor	Chlorophyta, Bryophyta, Lycopodiophyta, Monocots, Eudicots
I55 SQAPI	Eudicots (Cucurbitales)
I67 Bromein	Monocots (Poales)
I73 VTI	Eudicots (Lamiales)
I83 ANT Inhibitor	Coniferophyta
I90 MJTI	Eudicots (Caryophyllales)

### Distribution of the restricted protein peptidase inhibitor families

The following families have a restricted distribution in plants, being specific of clades ranging from algae to land plants:

Family I2: named Kunitz-A, includes mainly animal serine peptidase inhibitors. BLAST searches have not identified these inhibitors in land plants, only in the algae *C. reinhardtii*. The MEROPS database shows that they are also present in other algae species.

Family I7: named squash serine peptidase inhibitors, are specific for plants and they have only been described in Cucurbitales. BLAST searches indicate the existence of two members in *C. sativus*.

Family I18: mustard family of serine peptidase inhibitors specific for plants and only described in Brassicales. BLAST searches identified six different members in *A. thaliana*.

Family I37: potato carboxypeptidase inhibitor family, inhibitors of metallopeptidases of the M14 family. Exclusively described in Solanales and not found in BLAST searches on the selected genomes.

Family I39: named alpha-2 macroglobulins, are proteins that interact with peptidases regardless of catalytic type. Abundant in bacteria and animals, according to MEROPS database they are also present in *M. pusilla* and *P. trichocarpa*. BLAST searches confirm their existence in the algae *M. pusilla*, but not in *P. trichocarpa*.

Family I55: named squash aspartic peptidase inhibitors, are specific for plants and they have mainly been described in Cucurbitales. BLAST searches reveal their specificity for Cucurbitales, where three members were identified in *C. sativus*.

Family I67: named bromeins, are inhibitors of the cysteine peptidase bromelain. Only described in the monocot *Ananas comosus* and not found by BLAST searches on the selected genomes.

Family I73: Veronica trypsin inhibitor family merely described in the eudicot *Veronica hederifolia* and not found by BLAST searches on the selected genomes.

Family I83: inhibitors of serine endopeptidases present in insect species and also in the Conifer *Picea sitchensis*. Not found by BLAST searches on the selected genomes.

Family I90: trypsin inhibitors only described in eudicot plants from the order Caryophyllales, and not found by BLAST searches on the selected genomes.

### Evolution of the main protein peptidase inhibitor families

**Figure 1:**
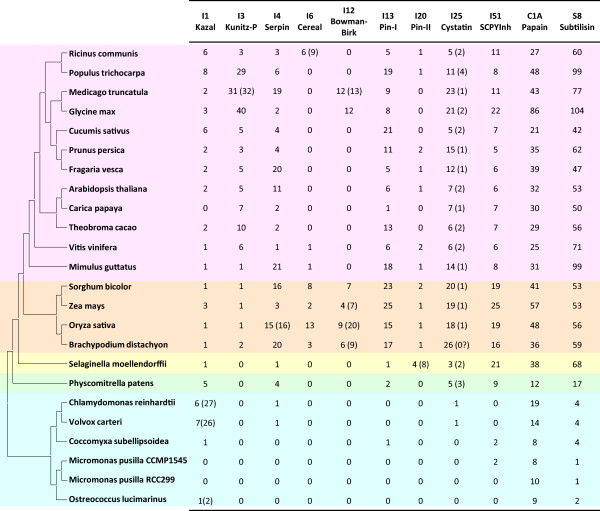
**Number of peptidases and their inhibitors in selected plant species.** Schematic evolutionary tree of fully sequenced plants including for each species the number of peptidase (C1A Papain and S8 Subtilisin) and peptidase inhibitory sequences (I plus number and name). In brackets the number of inhibitory domains for families I1, I3, I4, I6, I12 and I20; or the number of sequences with an additional cystatin-like domain for I25 family. Algae species are coloured in blue, moss in green, pseudofern in yellow, monocots in orange and eudicots in pink.

Families of peptidase inhibitors presented in most plant clades were selected for a deeper analysis. The I9 and I29 families comprise the inhibitory propeptides of the S8 subtilisin and C1A papain peptidase families, which are always contained in the same molecule. Then, they were excluded for the evolutionary study. Genome extensive searches were done for the rest of the families to know the distribution and the number of members of each one in each plant species. The results obtained compared with the location of the species in the phylogenetic tree of plants are summarized in Figure 1. In a general view, it is remarkable the lack of most peptidase inhibitor families in several algae. For example, any inhibitory sequence was detected in the genome annotation of the strain RCC299 of *M. pusilla*. The number of peptidase inhibitor families and members of each family increases with evolution. In monocot species all inhibitor families are present and, in general, with higher number of members than in eudicot species. In eudicots, some families are lacked in some clades, and there is a great variability of the number of members of each family. For example, Kunitz-P members rank from only one in *M. guttatus* to 40 in *G.max*. An evolutionary landscape for each one of these peptidase inhibitory families is showed in next sections.

### Gene content evolution of I1 Kazal in plants

**Figure 2:**
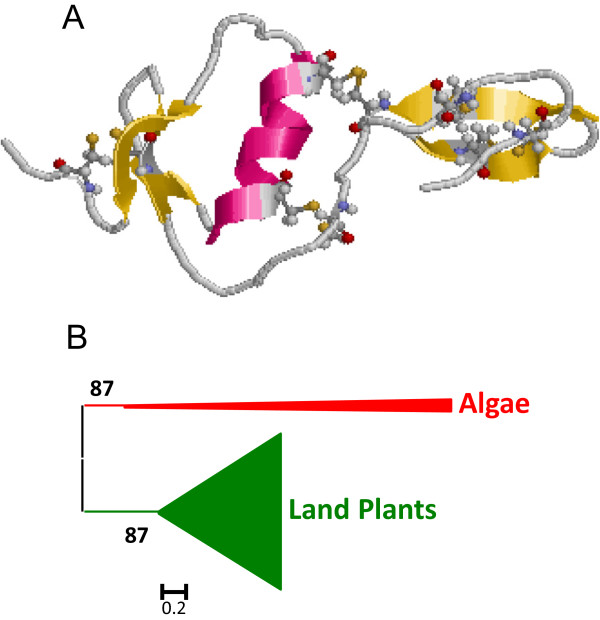
**Features of I1 Kazal peptidase inhibitors. (A)** Three-dimensional structure of a typical I1 inhibitor (2KCX). Cysteines are highlighted as balls and sticks and coloured in CPK. Red, α-helix; yellow, β-sheets. **(B)** Schematic PhyML phylogenetic tree using the selected Kazal sequences from the different plant species. Coloured triangles show clade-specific gene proliferations.

I1 Kazal peptidase inhibitors were present in all clades analyzed, from algae to land plants, with a number of members ranking from 0 in the two *Micromonas* species and in the eudicot *C. papaya* to 8 in *P. trichocarpa* (Figure 1). However, architectures for proteins containing domains of Kazal lineage vary among different clades. Whereas in land plants Kazal inhibitors were single domain proteins, in algae multidomain Kazal inhibitors were found (Figure 1), with a maximum of 10 different Kazal domains in a *V. carteri* protein. As a consequence, the number of I1 domains in the Chlorophylaceae algae is higher than that found in land plants. I1 Kazal proteins have a semi-extended structure composed by one α-helix and two β-sheets and stabilized by five disulphide bridges (Figure 2A).

To understand how the I1 Kazal lineage has evolved in the different plant clades, the individual Kazal domains from single domain proteins were aligned (see Additional file 1A). Extensive amino acid differences avoid the construction of a robust phylogenetic tree using all the Kazal sequences. Thus, sequences contributing to extensive gaps in the conserved regions of the alignment were discarded and a phylogenetic tree was constructed (see Additional file 2A). The corresponding schematic cladogram is shown in Figure 2B. As highlighted, two main clades were found, one from algae sequences and the other one from land plants. The evolutionary groups in the land plant sequences could not be clearly established in the tree. Eudicot sequences were mixed in different groups, with no evidences of species-specific proliferations. Monocot and moss sequences were grouped in separated clades supported by approximate likelihood-ratio test values (aLRT) higher than 65% but in a monophyletic clade common to eudicot sequences. This cladogram suggests that the Kazal family in plants has evolved differently between algae and land plants and that extensive sequence variations have took place in angiosperm species.

### Gene content evolution of I3 Kunitz-P in plants

**Figure 3:**
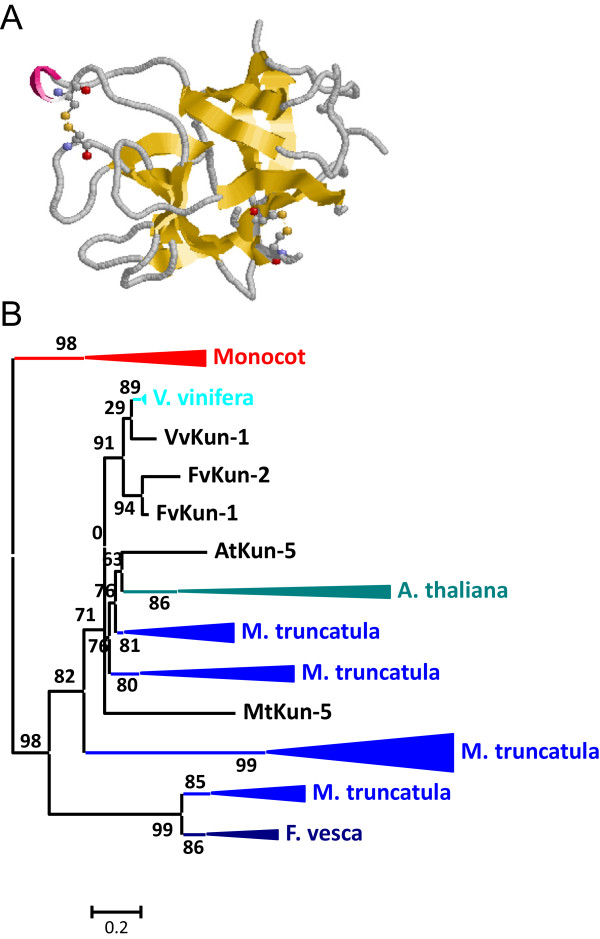
**Features of I3 Kunitz-P peptidase inhibitors. (A)** Three-dimensional structure of a typical I3 inhibitor (1AVU). Cysteines are highlighted as balls and sticks and coloured in CPK. Yellow, β-sheets. **(B)** Schematic PhyML phylogenetic tree using the selected Kunitz-P sequences from the different plant species. Coloured triangles show clade-specific gene proliferations.

I3 Kunitz-P peptidase inhibitors were only found in angiosperm species (Figure 1). The number of members of this family in each species varies considerably. In monocot species only 1 or 2 members are present. In eudicot species its number ranges from 1 in *M. guttatus* to 40 in *G. max*. All sequences were single domain proteins with the exception of a *M. truncatula* sequence that possess two different Kunitz-P domains in the same protein. Kunitz-P members are globular proteins composed by several β-sheets and stabilized by two disulphide bridges (Figure 3A).

To avoid the difficulties to create and explain a phylogenetic tree using the 174 sequences, several of them were selected. The sequences from the eudicot species *A. thaliana*, *M. truncatula* and *F. vesca* and all the monocot species were chosen. The individual Kunitz-P domains were aligned (see Additional file 1B). Sequences contributing to extensive gaps in the conserved regions of the alignment were discarded and a phylogenetic tree was constructed (see Additional file 2B). The corresponding schematic cladogram is shown in Figure 3B. As highlighted, monocot and eudicot clades are separated. In the eudicot clade, several species-specific proliferations are detected, with sequences ranging from 3 to 11, which are supported by aLRT values higher than 80%. These expansions suggest that the evolution of the Kunitz-P family in eudicots is the result of extensive duplications in specific species.

### Gene content evolution of I4 Serpin in plants

**Figure 4:**
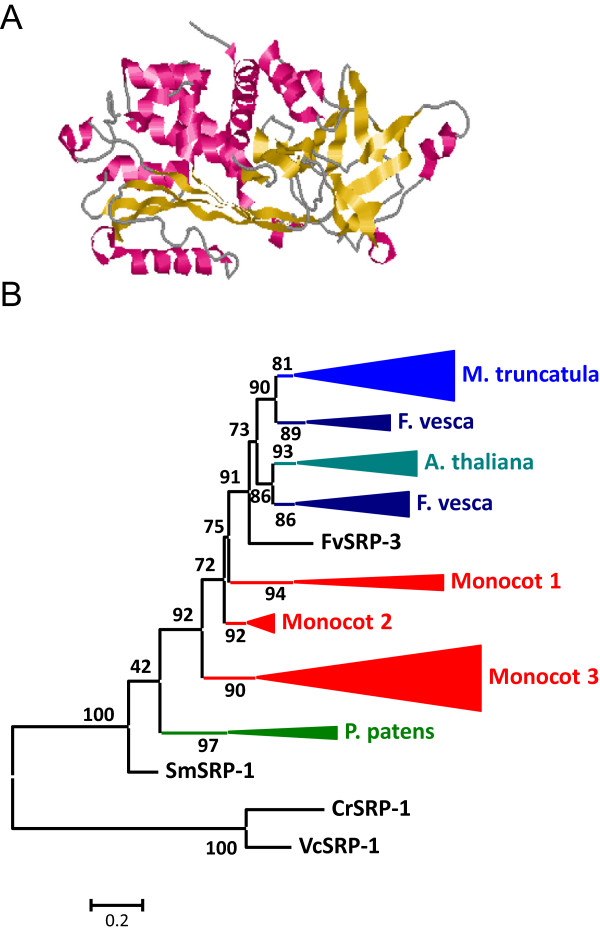
**Features of I4 Serpin peptidase inhibitors. (A)** Three-dimensional structure of a typical I4 inhibitor (3LE2). Red, α-helices; yellow, β-sheets. **(B)** Schematic PhyML phylogenetic tree using the selected Serpin sequences from the different plant species. Coloured triangles show clade-specific gene proliferations.

I4 Serpin peptidase inhibitors were present in all land plants analyzed and in the Chlorophyceae algae *C. reinhardttii* and *V. carteri*. Many genes putatively belonging to this family were extensively truncated and were not included in the study. The number of members of this family was low in basal plants, 1 in the algae and the pseudofern, and 4 in the moss. In higher plants, the number of members was very variable. In monocots, it ranges from 3 in *Z. mays* to 20 in *B. distachyon*, and in eudicots from 1 in *V. vinifera* to 21 in *M. guttatus* (Figure 1). All Serpin members were single domain proteins with the exception of an *O. sativa* protein that has two fully serpin domains. I4 Serpin proteins have a globular structure composed by several α-helix and β-sheets and without any disulphide bridge (Figure 4A).

Similar to that performed for the I3 family, several sequences were chosen to create the phylogenetic tree. The algae, pseudofern and moss sequences, as well as the sequences from the eudicot species *A. thaliana*, *M. truncatula* and *F. vesca* and the monocot species *S. bicolor* and *O. sativa* were selected. Proteins were aligned (see Additional file 1C), sequences contributing to extensive gaps in the conserved regions of the alignment were discarded and a phylogenetic tree was constructed (see Additional file 2C). The corresponding schematic cladogram is shown in Figure 4B. Two main clades were found, one from algae sequences and the other from land plants including the moss and pseudofern sequences. As highlighted, different clado-specific proliferations were detected, supported by aLRT values higher than 80%. Three different lineages from monocot sequences were found including sequences from both, *S. bicolor* and *O. sativa*. From eudicots, most of the sequences from *A. thaliana*, *M. truncatula* and *F. vesca* were found in separated groups, suggesting species-specific (or clade-specific) proliferations.

### Gene content evolution of I6 cereal in plants

**Figure 5:**
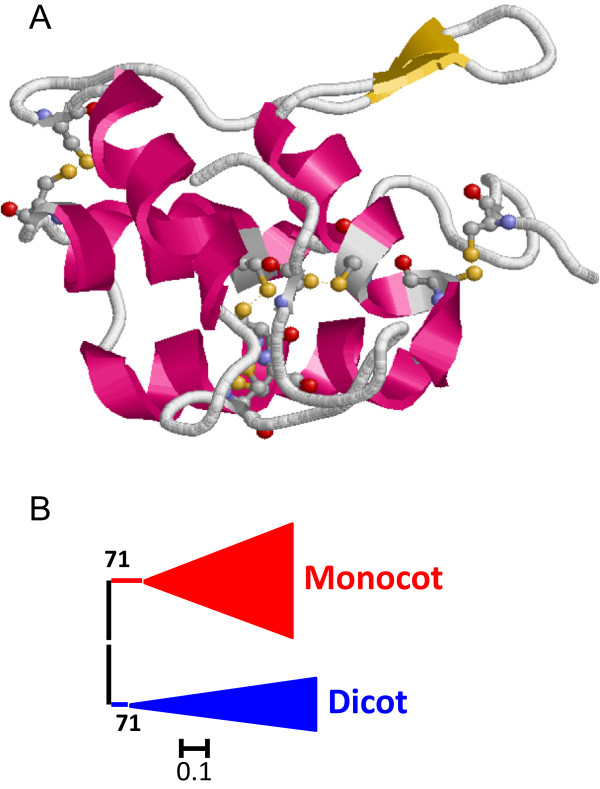
**Features of I6 Cereal peptidase inhibitors. (A)** Three-dimensional structure of a typical I1 inhibitor (1B1U). Cysteines are highlighted as balls and sticks and coloured in CPK. Red, α-helices; yellow, β-sheets. **(B)** Schematic PhyML phylogenetic tree using the selected Cereal sequences from the different plant species. Coloured triangles show clade-specific gene proliferations.

I6 Cereal peptidase inhibitors were present in all monocot species and in several eudicot species. The number of members ranged from 2 to 13 in monocot species and from 1 to 6 in eudicot species (Figure 1). All proteins where single domain inhibitors with the exception of 3 proteins from *R. communis* that had two different Cereal domains. Cereal proteins have a globular structure supported by five disulphide bridges (Figure 5A). Whereas most monocot members have the ten conserved cysteine residues essential to maintain this structure, eudicot members lack two cysteines and loss their ability to form one of the disulphide bridges.

To understand how the I6 Cereal lineage has evolved the Cereal proteins were aligned (see Additional file 1D). Two sequences from rice with extensive gaps that disturbed the alignment were discarded and a phylogenetic tree was constructed (see Additional file 2D). The corresponding schematic cladogram is shown in Figure 5B. Two different lineages, one for monocots and other for eudicot species were found, supported by aLRT values higher than 70%.

### Gene content evolution of I12 Bowman-Birk in plants

**Figure 6:**
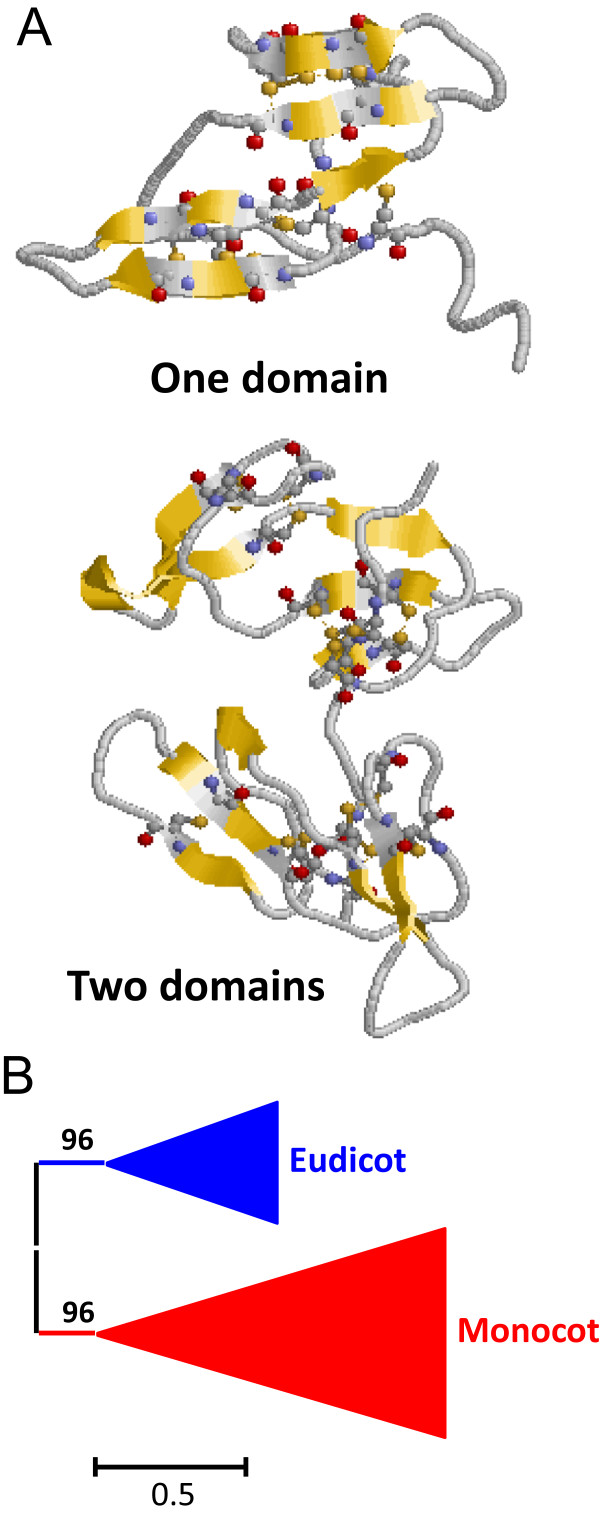
**Features of I12 Bowman-Birk peptidase inhibitors. (A)** Three-dimensional structure of typical I12 inhibitors with one domain (1BBI) or two domains (2FJ8). Cysteines are highlighted as balls and sticks and coloured in CPK. Yellow, β-sheets. **(B)** Schematic PhyML phylogenetic tree using the selected Bowman-Birk sequences from the different plant species. Coloured triangles show clade-specific gene proliferations.

I12 Bowman-Birk peptidase inhibitors have evolved similarly to I6 inhibitors. I12 sequences were present in all monocot species and in some eudicot species. The number of members ranged from 4 to 9 in monocot species and 12 in the two eudicot species (Figure 1). Most proteins where single domain inhibitors. One sequence from *M. truncatula*, three from *B. distachyon* and *Z. mays*, and five from *O. sativa* had two inhibitory domains, and three sequences from *O. sativa* had three. Bowman-Birk proteins have a globular structure composed by several β-sheets and supported by six disulphide bridges in the single domain proteins, and four or five disulphide bridges in the proteins with two inhibitory domains (Figure 6A).

The Bowman-Birk proteins were aligned (Additional file 1E) and a phylogenetic tree was constructed (Additional file 2E). As for I6 Cereal family, the corresponding schematic cladogram shows two different lineages, one for monocots and another one for eudicot species, supported by aLRT values higher than 95% (Figure 6B).

### Gene content evolution of I13 Pin-I in plants

**Figure 7:**
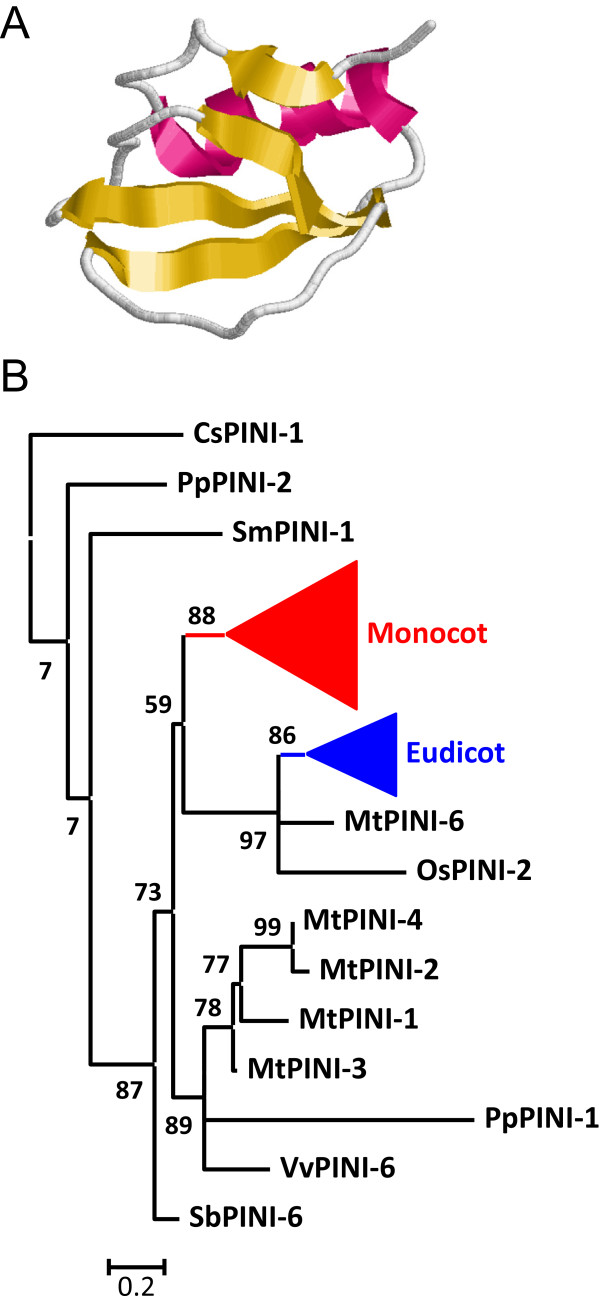
**Features of I13 Pin-I peptidase inhibitors. (A)** Three-dimensional structure of a typical I1 inhibitor (2CI2). Red, α-helix; yellow, β-sheets. **(B)** Schematic PhyML phylogenetic tree using the selected Pin-I sequences from the different plant species. Coloured triangles show clade-specific gene proliferations.

I13 Pin-I peptidase inhibitors were present in all land plants studied and in the Trebouxiophyceae algae *C. subellipsoidea*. The number of members of this family was low in basal plants, 1 or 2, and was elevated in all monocot species, from 15 to 25 members. In eudicot species, a wide range of inhibitors was found, from 1 member in *C. papaya* to 21 members in *C. sativus* (Figure 1). All Pin-I members were single domain proteins (Figure 7A). I13 Pin-I proteins have a globular structure mainly composed by β-sheets and without any disulphide bridge (Figure 7A).

As for some other protein families, to avoid the difficulties to create and understand a phylogenetic tree using the 242 sequences, several of them were selected. The algae, pseudofern and moss sequences, as well as the sequences from the eudicot species *A. thaliana*, *M. truncatula*, *F. vesca* and *V. vinifera*, and the monocot species *S. bicolor* and *O. sativa* were chosen. Proteins were aligned (see Additional file 1F) and a phylogenetic tree was constructed (see Additional file 2F). The corresponding schematic cladogram is shown in Figure 7B. As highlighted, two clado-specific proliferations were detected, supported by aLRT values higher than 85%. The lineage from monocot sequences included 36 sequences, and the lineage for eudicots included 20 sequences. The most divergent monocot and eudicot sequences were not included in these clades.

### Gene content evolution of I20 Pin-II in plants

**Figure 8:**
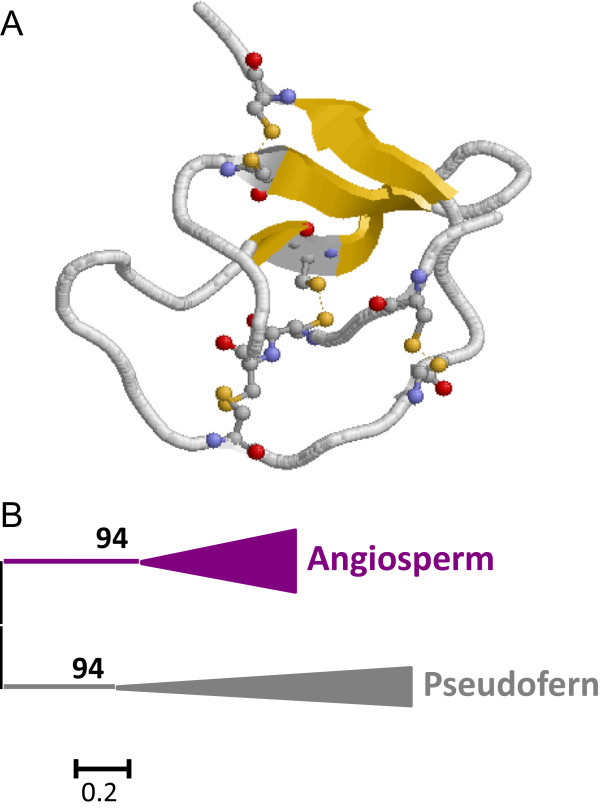
**Features of I20 Pin-II peptidase inhibitors. (A)** Three-dimensional structure of a typical I20 inhibitor (4SGB). Cysteines are highlighted as balls and sticks and coloured in CPK. Yellow, β-sheets. **(B)** Schematic PhyML phylogenetic tree using the selected Pin-II sequences from the different plant species. Coloured triangles show clade-specific gene proliferations.

I20 Pin-II peptidase inhibitors were scattered represented in the Viridiplantae. They were absent in algae and mosses and 4 members were present in the pseudofern. In Angiosperms, all monocot species had 1 or 2 members. In eudicots, whereas several species had 1 or 2 members some other lacks this kind of inhibitors (Figure 1). All Angiosperm Pin-II members were single domain proteins and the pseudofern members included two different Pin-II domains in each inhibitory protein. Pin-II proteins have a globular structure stabilized by four disulphide bridges with a large number of amino acids not included in a typical secondary structure (Figure 8A). All angiosperm sequences have eight conserved cysteines to form four disulphide bridges. The *S. moellendorffii* sequences lack one or three of these cysteines but contain six additional cysteines in their sequence that suggests a different three-dimensional structure for these inhibitors.

To understand how the I20 Pin-II lineage has evolved in the different plant clades, all the individual Pin-II domains were aligned (see Additional file 1G) and a phylogenetic tree was constructed (see Additional file 2G). The corresponding schematic cladogram is shown in Figure 8B. Two different branches have been found, one of them comprised by the pseudofern sequences and the other one by the Angiosperm sequences, supported by aLRT values higher than 90%. The monocot and eudicot sequences were not separated in the clade suggesting a common evolution and, probably, a loss of this type of inhibitors in several species during evolution. The phylogram and the extensive variations in sequence also suggest a different origin of pseudofern and angiosperm sequences.

### Gene content evolution of I25 cystatin in plants

**Figure 9:**
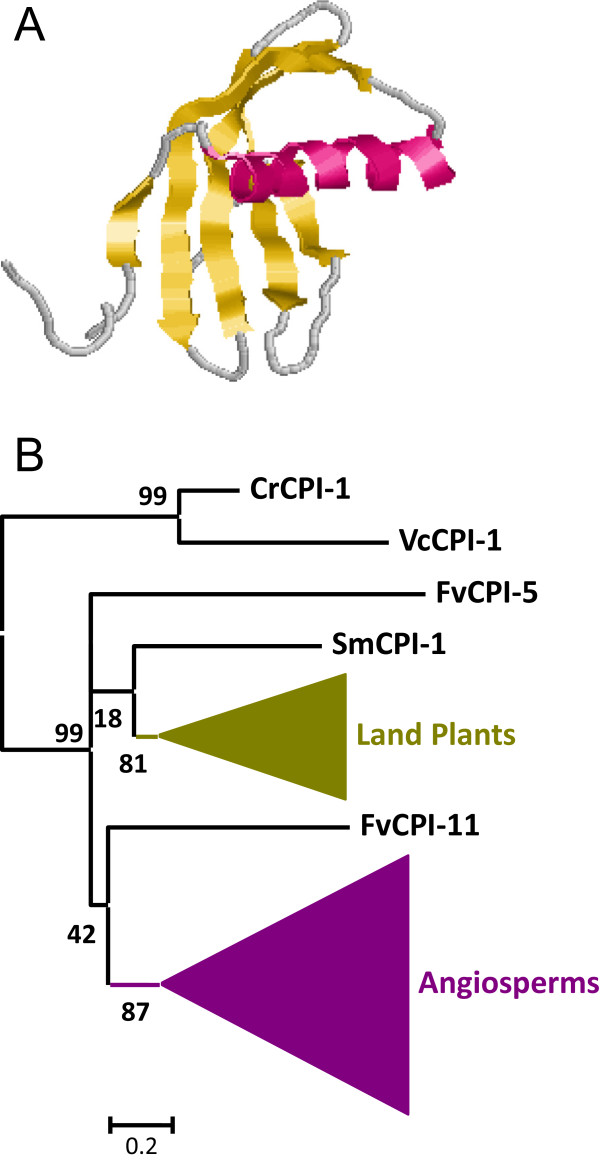
**Features of I25 Cystatin peptidase inhibitors. (A)** Three-dimensional structure of a typical I25 inhibitor (1EQK). Red, α-helix; yellow, β-sheets. **(B)** Schematic PhyML phylogenetic tree using the selected Cystatin sequences from the different plant species. Coloured triangles show clade-specific gene proliferations.

I25 Cystatin peptidase inhibitors were present in all land plants and in the Chlorophyceae algae. Their number progressively increases on evolution from 1 member in algae species to 3 or 5 in basal plants and ranking between 5 and 26 in angiosperms (Figure 1). All members are single domain proteins, although most species, with the exception of the algae and, apparently, the monocot *B. distachyon* had at least 1 member with a cystatin-like C-terminal extension responsible to inhibit C13 legumain peptidases. I25 Cystatin proteins have a globular structure mainly composed by β-sheets and without any disulphide bridge (Figure 9A).

As for some other families, the algae, pseudofern and moss sequences, as well as the sequences from the eudicot species *A. thaliana*, *M. truncatula*, *F. vesca* and *V. vinifera*, and the monocot species *S. bicolor* and *O. sativa* were selected. After discarding sequences contributing to extensive gaps in the conserved regions of the alignment, a phylogenetic tree was constructed (see Additional files 1H and 2H). The corresponding schematic cladogram is shown in Figure 9B. As highlighted, two different clado-specific proliferations are detected, supported by aLRT values higher than 80%. One is composed by sequences from all land plant species and the second is composed only by angiosperm sequences. This cladogram suggests that evolution of the cystatin family in plants is the result of extensive duplications from ancestral genes, and the divergence of these sequences in single clades.

### Gene content evolution of I51 Serine Carboxypeptidase Y Inhibitors in plants

**Figure 10:**
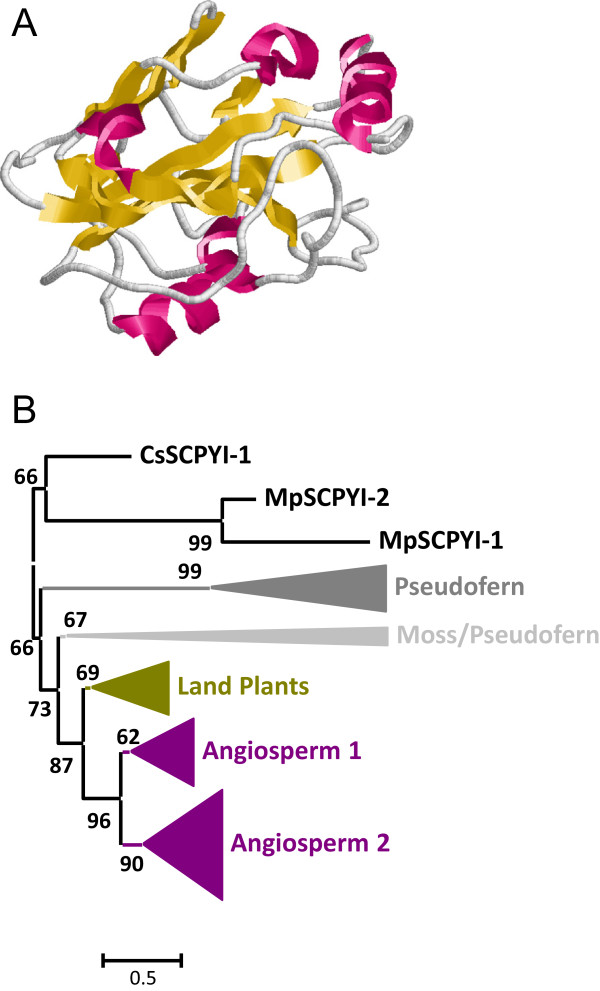
**Features of I51 SCPYInh peptidase inhibitors. (A)** Three-dimensional structure of a typical I51 inhibitor (1KN3). Red, α-helices; yellow, β-sheets. **(B)** Schematic PhyML phylogenetic tree using the selected SCPYInh sequences from the different plant species. Coloured triangles show clade-specific gene proliferations.

I51 Serine Carboxypeptidase Y Inhibitors (SCPYInh) were present in all land plants, and in the algae *C. subellipsoidea* and *M. pusilla* CCMP1545. The number of members of this family was low in algae, with only 2 members, and increase in the moss and pseudofern, with 9 and 21 members, respectively. This range of inhibitors was narrow in monocots, from 18 to 26 members, and was enlarged in eudicot species, ranging from 5 members in *P. persica* to 22 members in *G. max* (Figure 1). All I51 SCPYInh were single domain proteins. I51 SCPYInh proteins have a globular structure composed by β-sheets and α-helix without any disulphide bridge (Figure 10A).

The high number of sequences in this family prompted us to select the sequences from the same plant species that were used for the cystatin family. Proteins were aligned (see Additional file 1I), and a phylogenetic tree was constructed (see Additional file 2I). The corresponding schematic cladogram is shown in Figure 10B. As highlighted, different clades were detected, which were supported by aLRT values higher than 60%. Basal clades were composed by algae, moss and pseudofern sequences, and include a proliferation of *S. moellendorffii* sequences in a specific pseudofern clade. Angiosperm sequences were found in three different lineages. Two of them were only formed by monocot and eudicot sequences, and the third lineage was also formed by moss and pseudofern sequences.

### Coevolution of peptidases and their inhibitors in plants

To analyze the evolution of the different peptidase families, targets of the peptidase inhibitor families, is a key point to understand the meaning of the actual gene content of these peptidase inhibitory families. From the wide number of peptidase families C1A Papain and S8 Subtilisin families have been selected in function of their physiological importance in the plant, and the capacity of most inhibitor families to inhibit them. C1A Papain members are inhibited by I25 Cystatin inhibitors and by some I4 Serpin inhibitors. S8 Subtilisin members are inhibited by I1 Kazal, I3 Kunitz-P, I4 Serpin, I6 Cereal, I12 Bowman-Birk, I13 Pin-I and I20 Pin-II inhibitors.

The number of members of these two peptidase families in the different plant species analyzed in this work is present in Figure 1. Algae species have low number of peptidases, with more Papain than Subtilisin members. Moss has moderate number of members, with more Subtilisins. Pseudofern and angiosperms have higher numbers of peptidases, with some variability. Most angiosperms have Papain families ranging from 21 to 57 members, although *G. max* have more than 80 members. For Subtilisins, most angiosperm species present between 42 and 77 members, having *P. trichocarpa*, *G. max* and *M. guttatus* around 100 members.

**Figure 11:**
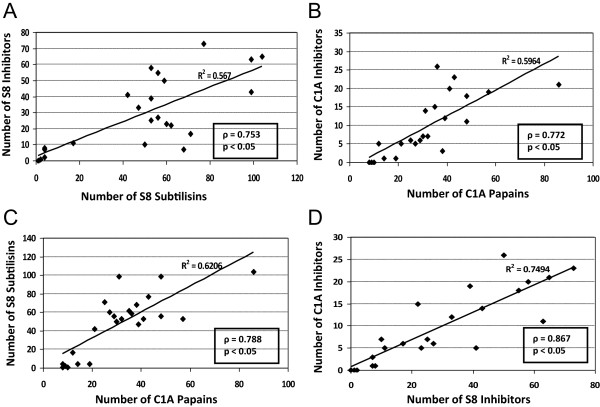
**Evolutionary correlations between peptidases and their inhibitors.** Dispersion graphs showing the linear trend of the two variables represented, the correlation coefficient of the line (R^2^) and the statistical result of the correlation statistical analysis (ρ; p < 0.05). Variables represented: **(A)** Number of S8 Subtilisins and their inhibitors. **(B)** Number of C1A Papains and their inhibitors. **(C)** Number of C1A Papains and S8 Subtilisins. **(D)** Number of S8 inhibitors and C1A inhibitors.

On an evolutionary context, the genomic content of both peptidases and their inhibitors could be correlated. Figure 11 shows the linear trend and statistical analysis of the correlation among the number of members of peptidases and their inhibitors. Statistical analyses indicate that there is a positive correlation between the number of C1A or S8 peptidases and their putative inhibitors, with a high variability determined by points that are far away of the regression lines. The number of C1A and S8 peptidases is also positively correlated, whereas the strongest correlation was found between the number of S8 and C1A inhibitors.

## Discussion

Identification of inhibitory peptidase families in plants provides a working definition of a basal core shared by most plant clades and a starting point to figure out the evolutionary cues regarding the expansion of peptidase inhibitory networks. Variability is the key word that defines this kind of proteins. None of the peptidase inhibitory families is ubiquitously present in the genomes of all species analyzed, mainly due to their lack in some algae genomes. In this way, the genome of *M. pusilla* RCC299 does not have any member of the nine most conserved peptidase inhibitory families, the most represented algae species have only members of three of these families, and four of these families are not present in any algae genome. In addition, conservation of peptidase inhibitory families is also partial in land plants. The basal moss and pseudofern species lack members of the I3, I6 and I12 families, and several eudicot species lack members of some of the most conserved peptidase inhibitor families. The number of members of each family is another feature that confirms this global variability. Although angiosperms own in general higher number of members than basal plants, the highest number of I1 Kazal domains is in the Chlorophyceae algae *C. reinhardtii* and *V. carteri*, and of the I20 Pin-II proteins is in the pseudofern *S. moellendorffii*. Among angiosperms, the number of members of different families presents a strong variation. In some families such as I3 Kunitz-P, I4 Serpin or I13 Pin-I there are eudicot species with more than 20 members and others with only 1 member of the same family. This strong variability has also been found in prokaryotes, where mostly the occurrence of individual types of inhibitors is limited to few bacterial species scattered among phylogenetically distinct orders or even phyla of microbiota [14]. Thus, variability has been confirmed as the main feature of peptidase inhibitory families.

At this point, it is desirable to know the evolutionary reasons that force this variability. Peptidase inhibitors may have two different functions. They are inhibitors of the endogenous peptidases, regulating the activity of the own plant peptidases to avoid an indiscriminate degradative action when it is not convenient [8, 9]. Furthermore, they could be also regulating the activity of exogenous peptidases, such as the peptidases that several pests and pathogens use to feed and to survive in the plant species they attack [11, 12, 48]. To understand which are the mechanisms related to this evolutionary variability, correlations between the number of peptidases and their inhibitors add some valuable information. The number of endogenous C1A or S8 inhibitors is positively correlated with the number of the peptidases they inhibit. Plant species with a high number of peptidases also contains a high number of inhibitors. This result is congruent with an evolutionary scenario in which endogenous peptidase proliferations are followed by peptidase inhibitor gene expansions. But this correlation is not perfect and some species have more or less inhibitors than those expected by their peptidase repertoire. Two possible reasons may explain these discrepancies: i) Several peptidases are not functional and, therefore, they have not force the increasing of the inhibitor members to regulate them; ii) Several inhibitors are not regulating endogenous peptidases and have proliferated to actually inhibit the peptidases used by the pests and pathogens to attack the plant. This second possibility has been previously appointed [49]. A diversity of mechanisms, such as the recruitment of additional protein-folding families as inhibitors, the combination of different inhibitor domains into a single molecule, the high rate of retention of gene duplication events and the hypervariation of contact residues have been postulated [49]. In the case of the plants, a mixed combination of evolutionary forces, the increase of endogenous peptidases and the fight against exogenous peptidases, will explain the actual repertoire of peptidase inhibitor present in land plants.

Another feature that supports the strong variability in the peptidase inhibitor repertoires and the possibility of a quick evolution mediated by pests and pathogens is the existence of small peptidase inhibitory families that are restricted to single species/clades. Ten of the 21 peptidase inhibitor families identified in plants are restricted to a clade: I2 and I39 to some algae lineages, I7, I18, I37, I55 and I67 to a eudicot or monocot order, and I73, I83 and I90 to a single angiosperm species. New gene families typically originate either from duplicate copies of a gene that become sufficiently divergent and are no longer recognized as members of the same family, from genes horizontally transferred, or from genes originated *de novo* from previously non-coding sequences [50]. The small peptidase inhibitor families of plants are most probably derived from duplications followed by strong sequence divergence. For example, the I55 SQAPI family, only present in Cucurbitales presents a three-dimensional structure similar to that of the members of the I25 phytocystatin family, suggesting a common ancestor gene for both families [51, 52]. Likewise, the three-dimensional structure of the I18 MTI-2 family resembles the structure of the I13 Pin-I family [53, 54]. In this way, the selective losses of cysteine residues, and the conformational changes derived from it, have been postulated as a manner to get variability to be more effective against pathogen/pest attack [55]. In contrast to the birth of new gene peptidase inhibitor families, the death of peptidase inhibitor families is a process that should be further investigated in plants. The loss of members from a family in some clades/species can be due to the loss of the physiological constraints that previously impose as deleterious the absence of this family [50]. In the case of the plants, endogenous physiological activity of peptidases should be carefully regulated. The existence of a statistically significant correlation between peptidases and inhibitors in the plant kingdom supports that the loss of a specific physiological mechanism controlled by a peptidase could be correlated to the loss of some specific inhibitors of this peptidase. However, strong variations in the number of inhibitors in a specific peptidase inhibitor family pointed to a more active evolutionary mechanism based in the interaction with biotic stresses. Thus, the loss of peptidase inhibitor members should be most probably related with the absence of the driving force, for example, with the loss of the deleterious effects induced by a specific pathogen/pest species.

## Conclusions

In conclusion, comparative genomics has allowed us to obtain further insights on the present repertoire of peptidase inhibitors in plants, and on the evolution of these peptidase inhibitor families. Variability in response to the endogenous and exogenous peptidases that have to be regulated by the inhibitors is the main feature of this kind of proteins. While new families commonly restricted to a specific species/clade will be probably found in next year’s, the evolutionary mechanisms that allow this strong diversity should be in deep investigated.

## Methods

### Sequence searches

MEROPS v9.10 database [13] of peptidases and their inhibitors was used to establish the protein peptidase inhibitor families present in plants by looking for the distribution of each family in the different kingdoms. Then, Blast searches for peptidases and peptidase inhibitors were performed in publicly available genome databases. Sequences were identified by searching the current genome releases at the Phytozome v9.1 comparative genomic database [18]. Blast searches were made in a recurrent way. First, a complete amino acid plant sequence from data banks corresponding to a protein of the family was used. Then, the protein sequences of each plant species were employed to search in the same species. Finally, after an alignment of the proteins found in plants, the conserved region surrounding the catalytic sites from the species most related was used to a final search in each plant species. To test the accuracy of the results, retrieved sequences were compared, when possible, with the identified sequences in each plant species of the same family in the GreenPhylDB v3.0 comparative genomics database [19].

### Domain architecture prediction

Amino acid sequences for plant proteins putatively including at least one peptidase or protein peptidase inhibitory domain were subjected to a sequence search in the Pfam database v27.0 [56] to know the combination of domains within each protein.

### Protein alignments and phylogenetic trees

Alignments of the amino acid sequences were performed using the default parameters of MUSCLE v3.8 [57]. Sequences with extensive gaps were manually excluded from phylogenetic studies. Phylogenetic and molecular evolutionary analyses were conducted using the programs PhyML v3.0 and MEGA v5.2 [58, 59]. The displayed protein peptidase inhibitor trees were constructed by means of a maximum likelihood PhyML method at Phylogeny.fr home using a BIONJ starting tree [60]. The approximate likelihood-ratio test (aLRT) based on a Shimodaira-Hasegawa-like procedure was applied as statistical test for non-parametric branch support [61]. All families were also analysed with the Maximum parsimony and the Neighbour-Joining algorithms, and with different gap penalties. No significant differences in the tree topologies were detected. Information about gene models for all proteins used to construct the phylogenetic trees is compiled in Additional file 3.

### Statistical methods

A linear trend line has been drawn through the number of peptidases and their inhibitors in different plant species. The R^2^ value indicates how well data fits the line. To test the statistical significance of the correlation results between the number of peptidases and their inhibitors in different plant species, a Pearson Product Moment Correlation test was performed using SigmaStat v3.5 software. A correlation coefficient (ρ) positive and a p value lower than 0.05 means that the two variables tends to increase in a concerted manner.

## Electronic supplementary material

Additional file 1: **Comparison of the amino acid sequences of the different peptidase inhibitor families.** (PDF 129 KB)

Additional file 2: **Phylograms of the peptidase inhibitor families.** (PDF 214 KB)

Additional file 3: **Information about the sequences used in the phylogenetic trees**. (PDF 43 KB)
