# Supplementary material for: Plant protein peptidase inhibitors: an evolutionary overview based on comparative genomics
Source: BMC Genomics. 2014 Sep 25;15(1):812. doi: 10.1186/1471-2164-15-812 (PMC4189545; doi:10.1186/1471-2164-15-812)
Supplement: Supplementary file 1 — Additional file 1: Comparison of the amino acid sequences of the different peptidase inhibitor families. (PDF 129 KB) [file 12864_2014_6493_MOESM1_ESM.pdf]

Additional File 1. Comparison of the amino acid sequences of the different peptidase inhibitor families. The alignments were generated using the MUSCLE program. Rc, *Ricinus communis*; Pt, *Populus trichocarpa*; Mt, *Medicago truncatula*; Gm, *Glycine max*; Cs, *Cucumis sativus*; Ppe, *Prunus persica*; Fv, *Fragaria vesca*; At, *Arabidopsis thaliana*; Tc, *Theobroma cacao*; Vv, *Vitis vinifera*; Mg, *Mimulus guttatus*; Sb, *Sorghum bicolor*; Zm, *Zea mays*; Os, *Oryza sativa*; Bd, *Brachypodium distachyon*; Sm, *Selaginella moellendorffii*; Pp, *Physcomitrella patens*; Cr, *Chlamydomonas reinhardtii*; Vc, *Volvox carteri*; Csu, *Coccomyxa subellipsoidea*; Mp, *Micromonas pusilla*.

A. I1 Kazal.

```
CsuKaz-1        -----------------------------------TSGAQAPKAAAPTAQPPKAATPGQA
VcKaz-2         ---------------------------------------------KSICQELAGAMPQGG
CrKaz-3         -----------------------------------------------------MSKSRRI
PpKaz-3         ------------------------------------------------------MATSGW
PpKaz-2         -------------------------------------------------------MAALG
PpKaz-1         ------------------------------------------------------------
SmKaz-1         ----------------------------------------------------------MG
PpKaz-4         -----------------------------------------------------MATPGSK
RcKaz-2         -----------------------------------------------------------M
PtKaz-5         -----------------------------------------------------------M
PtKaz-3         -----------------------------------------------------------M
PtKaz-4         -----------------------------------------------------------M
RcKaz-4         -----------------------------------------------------------M
RcKaz-3         ------------------------------------------------------------
PtKaz-6         ----------------------------------------------------------MA
AtKaz-2         ------------------------------------------------------------
AtKaz-1         ------------------------------------------------MKFKIRISAMST
ZmKaz-3         ------------------QRKLLKAPTGFHGRNFNRPHRRGLLLTDPEGRLDSRPSPATP
OsKaz-1         -------------------------------------------------------MPPSY
BdKaz-1         ------------------------------------------------------------
ZmKaz-2         ------------------------------------------------------------
SbKaz-1         ------------------------------------------------------------
ZmKaz-1         ------------------------------------------------------------
PtKaz-2         -----------------------------------------------------------M
PtKaz-7         -----------------------------------------------------------M
TcKaz-2         -----------------------------------------------------------M
RcKaz-5         ----------------------------------------------------------MA
VvKaz-1         ---------------------------------------------------------MEF
PtKaz-1         ------------------------------------------------MIYPKKKMMMMM
RcKaz-1         -----------------------------------------------------MIPILFP
TcKaz-1         MLISFLVVSDKVNGTCPVWKILDLGPFPFHPYYYNFYPNWGLGFGFENLLQTILSIAAQM
GmKaz-3         ----------------------------------------------------------MA
MgKaz-1         -------------------------------------------------------MQLFS
MtKaz-1         ----------------------------------------------------------MA
MtKaz-2         ------------------------------------------------------------
GmKaz-1         ------------------------------------------------------------
GmKaz-2         ------------------------------------------------------------
CsKaz-1         ------------------------------------------------------------
FvKaz-2         ---------------------------------------------------------MDL
FvKaz-1         -----------------------------------------------------MEHTHFL
PpeKaz-1        -------------------------------------------------------MEPQF
PpeKaz-2        ------------------------------------------------------MDTQFP


CsuKaz-1        PSPAPLPPCNCAGVAESLVCGFDDKT----------------------------------
VcKaz-2         SRFRGACDSFTLGAVFDLSSPDAAPLPWSFQSFTVNSQVDLERMRSTLGASLEYMEVDLQ
CrKaz-3         GLARLQPVRSSLVVAFWLLLAVSRLA----------------------------------
PpKaz-3         NPRRVHVFVVLFVTVFSFAAVVHARTEE-------NSFVVSRHRAEGSVNVVVGGGSPCD
PpKaz-2         RGPRCFEALLLLLSVVVFHCGVVIQARTEENSFVVSQHGGGGGGGDGSVNLVTRGASPCR
PpKaz-1         MESVRECPSSVLLLLLALSLMFAVSAEE--------------------------LFLGKP
SmKaz-1         RKKNSLLWALQLLAFLALVSGDQDGQ----------------------------------
PpKaz-4         QLRLLFCVASLMLFFFANVNYAAVEETWSSVSGDAGEGTLGVVLADTSPCHSPFETQYRQ
RcKaz-2         ATFKNIAPLCMMLLVFIVCVSVAQSQ----------------------------------
PtKaz-5         ATSKILAPLCLMVLVFGLCLSMVESQ----------------------------------
PtKaz-3         ATSKIVAPLCLMVLVFGLCLPKAQSQ----------------------------------
PtKaz-4         ATSKIVAPLCLMVLVFCLSLSMVKSQ----------------------------------
RcKaz-4         TTSFKKRTLLCMIVVVATCVLLLTNG----------------------------------
RcKaz-3         ---------MMMVVVLGVCLTMVESK----------------------------------
PtKaz-6         TSIRVAQLCIVMVLVLGLCLTMVQAQ----------------------------------
AtKaz-2         ---MPSLTLSIRFLFLVLCLIGLQAA-D--------------------------------
AtKaz-1         ISPSLAVIAFLFLILLNLSSVFADPS---------------------------------T
ZmKaz-3         GMPSSRLVHLILLATLSLLLAQTLAS----------------------------------
OsKaz-1         TPTARLRLLVLFLASLSLLFAQTLASSS--------------------------------
BdKaz-1         -TTARFLLFVAALSLVLLPFPGTLAA----------------------------------
ZmKaz-2         -MPSSRLVHLILLATLSLLLAQTLAS----------------------------------
SbKaz-1         -MPPSRLVHLILLATLSLLLAQTLAT----------------------------------
ZmKaz-1         ---------------LASSSPAPASA----------------------------------
```

```
PtKaz-2         SKFSAIFTCVTIFLLSSLCFPIALSE---------------------------------T
PtKaz-7         SKLSLISTLCIAIFLSSLCFQIARSE---------------------------------P
TcKaz-2         SFPIPFRKPALSLLLLLLVVPFSSSS----------------------------------
RcKaz-5         RSVPILAPLCLLLALIGLCWPVEAQS--------------------------------KP
VvKaz-1         NRSRLLSSFVVIFIVFAICFPPTVTSS---------------------------------
PtKaz-1         MIPSTLLIHFLLIASIALFSAPTVRS----------------------------------
RcKaz-1         FSDSTFRRPIFLFLIFSLCLIPVLRS----------------------------------
TcKaz-1         PCFTATALHILTFVLLLFCFSAPIAQSL-------------------------------Q
GmKaz-3         GITKTSHFSVVLIAVVALCVFPAATS--------------------------------DN
MgKaz-1         CKPPSIFILAVLLTTLLPATVRSDPN----------------------------------
MtKaz-1         GISAVIIVISIFLMVLVVADDMSSSS-L------------------------------SS
MtKaz-2         ---MPKFSIILTFIALIFIFPILTTA--------------------------------EN
GmKaz-1         -MPKSSRFLVVAIAVAALCVILPVAAAA------------------------------DL
GmKaz-2         MPKFSRFLAVVAIAVAALCVILPVAAAV------------------------------DL
CsKaz-1         -------------------RSEDISS----------------------------------
FvKaz-2         KSPKSHLLLFTVFIITILCSFSAVRS----------------------------------
FvKaz-1         KSPISLILPILFTAFILGCFPTVRSE----------------------------------
PpeKaz-1        AKSPIPLILLILFPIFILCSFPTVRS---------------------------------E
PpeKaz-2        KSLISFFTLILFAFFIVLCSFPTVRSEL-------------------------------Q


CsuKaz-1        ----YTSSCAAGCANVGVKSQGACPAV------GP------------AP----------G
VcKaz-2         ASLGVVTTHS---NTANGTSSNPRGGPNASASPKP------------VPR----EDC---
CrKaz-3         ------------------GAADAAL--------SE------------ES-----FDC--R
PpKaz-3         SAVEVQSDEPLIQSVTSEDAADVASNLSKGPCIRQ------------TT-----MSC--P
PpKaz-2         SGAELRPEESLIRSVVSKAAADGANNVLIEPCILQ------------TT-----KSC--P
PpKaz-1         NQGSAWELPL-QHAAPCDHFSLVCGEEKGDNFKSSGLEADFNILSEGDPPIEKEPLC--T
SmKaz-1         ------RLSIIDSGSRVLDGGDAAK-----------------------------IEC--P
PpKaz-4         LSQGVASVAS--GGGASDTPTELCNHK------MK------------KL-----TSC--P
RcKaz-2         ------------------ITINLCPGP------MS------------PP-----EGC--P
PtKaz-5         -------------------SYGVCEGF------DP------------EA-----PRC--A
PtKaz-3         ---------------------DVCAGVE-----RP------------DPET---IPC--T
PtKaz-4         -------------------SYGVCAGAA-----RP------------DPET---IPC--T
RcKaz-4         -----------------VQAQNLCPG-------SE------------PP-----PSCAGP
RcKaz-3         -----VQVPNLCDLAKSASPGKDCS----------------------------------A
PtKaz-6         -----------------RGSVNLCPG-------ST------------SR-----GTCTGP
AtKaz-2         ------DFPD-------KSRGDVCPR-------VK------------DR-----GGC--T
AtKaz-1         EGGEIIRLPS-EKINGEKNRGEFCEG-------IA------------KP-----ASC--P
ZmKaz-3         ----SAPVPA--AGSASAESGDPCAATVADG--EA------------DV-----PLC--P
OsKaz-1         ---------------AEAEVSDPCAAPVSDG--GS------------EA-----QLC--P
BdKaz-1         -----------------SAAADPCAAAAESDAASG------------EI-----PLC--P
ZmKaz-2         ----SAPVPA--AASASTESGDPCAAAVADG--EA------------DV-----PLC--P
SbKaz-1         ----SSPTPAAASAAAEAESGDPCAAAVAD-GDGD------------VP------LC--P
ZmKaz-1         --------------ATAGEPGDPCAAAVADG--DG------------DV-----PLC--P
PtKaz-2         GAGILIQEVT-----REDGKGDACAG-------LK------------AP-----ASC--P
PtKaz-7         DAAILIQEVT-----NKDGKGEACAG-------LK------------AP-----ASC--P
TcKaz-2         TGGSQQQLSA----TRLLSEADLCAS-------TP------------QP-----SSC--P
RcKaz-5         ENGMLIEQIT------SKGNGDVCKG-------VT------------AP-----ASC--P
VvKaz-1         ----VIRLPS--DAVDGESDGGLCGG-------SE------------VA-----ASC--P
PtKaz-1         -DANPIRLPTLDHNNNNDDDDDACGEWS-----RR------------SPV----SSC--P
RcKaz-1         -DPNPIRLPT-HIIDNQDDNVDPCGE-------YG------------RP-----VSC--P
TcKaz-1         DNSGSIRLPS-DGFTSAHENEGVCAR-------FT------------KP-----ASC--P
GmKaz-3         VASAVLGLPS----HVAGEGKNLCSAA--------------------AP-----SSC--P
MgKaz-1         ----FLKLPS-------DYENDVCPL-------NS------------DA-----DTC--P
MtKaz-1         SSSSVIRLPS----KVTAEGKNVCAGA------VA------------S------SWC--P
MtKaz-2         EESSVLRLPS----------QNVCS--------VT------------TP-----SSC--P
GmKaz-1         EDPGVLRLPS----------DSLCG--------KT------------TP-----SSC--P
GmKaz-2         EDPGVLQLPS----------ESLCG--------KT------------MP-----LSC--P
CsKaz-1         ----AIRLPS--EATNNHGDIDLCS--------VS------------AP-----SYC--P
FvKaz-2         --DPIIKLPS-----DSDSDSGACGGSSL----SR------------SP-----VVC--P
FvKaz-1         QEPSFIKLPS------ESQPADVCPVV------VK------------SPVHA--LSC--P
PpeKaz-1        HESTIIRLPS------DSQSADRCGSTPSSPLSSS------------SSPWSR-PMC--P
PpeKaz-2        TESTIIRLPS------ESEPADACAGS------IR------------SP-----SLC--P


CsuKaz-1        VQCADS-TEVCGGDGVTYSNTCT-PKSKGVAIINNGPCK---------------------
VcKaz-2         -VCAEVYKPVCGSDGQDYSSAC-HAWCAGLNSWAAGTCNGTGVDD----------ASNGG
CrKaz-3         AGCKRAFKPACGADGRTYASACL-AKCQGVQVAHHGPC----------------------
PpKaz-3         VKCFRS-YPVCGTDKVTYRCGAADANCAGVEVAYDGFCNLWEGDR----------NVGSS
PpKaz-2         VKCVRT-DPVCGVDKVTYWCGAADAKCAGVEVAHDGSCNLWELGT----------ATSST
PpKaz-1         VNCFRA-DPVCGSDGVTYWCGVAEAKCAKVEVAHDGYCDI----W----------NGGTK
SmKaz-1         VSCLRA-DPVCGANGVTYWCGSRDAACDGVEISHTGYCSP----G----------N-GGA
PpKaz-4         VQCFRA-DPVCGTDNITYWCGAADAKCAGVEVAHDGYCNVWELDA----------NVGST
RcKaz-2         IACLVP-DPVCGANGVTYWCGCPDALCAGVRVVKFGEC----------------------
PtKaz-5         VRCSVP-DYVCGTDGVTYTCGCKDAFCNGVDVVKKGKC----------------------
PtKaz-3         INCFVP-DPVCGTDGVTYSCGCLDAFCHGVDVVKEGEC----------------------
PtKaz-4         INCLVA-DPVCGTDGVTYTCGCYDAFCHGVEVVKKGEC----------------------
RcKaz-4         INCFRP-DPVCGANGVTYWCGCPDAACAGVPVVKLEAC----------------------
RcKaz-3         IRCLRY-DPVCGVNGVTYGCGCPEANFYAVRVGKLGAC----------------------
PtKaz-6         INCFRA-DPVCGANGVTYGCGCPEAACARVRVVKLGAC----------------------
AtKaz-2         INCFRA-DPVCGTDGVTYWCGCPDAACHGARVVKKGACDT----G----------N-AGS
AtKaz-1         VQCFRP-DPVCGEDSVTYWCGCADALCHGVRVVKQGACDV----G----------N-GVG
ZmKaz-3         VRCFRP-DPVCGADGVTYWCGCPEATCAGARVARRGYCEVGSGMGVADPRHHGRAQRGGA
```

```
OsKaz-1         VRCFRP-DPVCGADGVTYWCGCPEAACAGARVARRGYCEV----G----------A--GS
BdKaz-1         VRCFRP-DPVCGADGVTYWCGCPEAVCAGARVARRGYCEV----G----------A--GS
ZmKaz-2         VRCFRP-DPVCGADGVTYWCGCPEATCAGARVARRGYCEV----G----------AGSGS
SbKaz-1         VRCFRP-DPVCGADGVTYWCGCPEATCAGARVARRGYCEV----G----------A--GS
ZmKaz-1         VRCFRP-DPVCGADGVTYWCGCPEAACAGARVARRGYCEV----G----------A--GS
PtKaz-2         INCFRA-DPVCGVDGVTYWCGCADALCSGTRVDKLGACEV----G----------S-GGS
PtKaz-7         INCFRA-DPVCGFDGVTYWCGCADAMCSGTRVAKLGACEV----G----------S-GGS
TcKaz-2         INCFRA-DPVCGDDGVTYWCGCADAHCAGAKVVKIGFCEV----G----------NSGGN
RcKaz-5         INCFRA-DPVCGVDGRTYWCGCDDALCSGTTVAKIGACDM----G----------N-GGS
VvKaz-1         VKCFRP-DPVCGVDGVTYWCGCTEARCAGTAVAHFGICEV----G----------N-GSA
PtKaz-1         VKCFRP-DPVCGVDGVTYWCGCDDARCAGTKVTKKGFCEV----G----------N-NGA
RcKaz-1         VNCFRT-DPVCGVDGVTYWCGCRDAWCAGTKVAKKGFCEV----G----------N-NGA
TcKaz-1         VNCFRT-EPVCGVDGVTYWCGCADALCAGTRVAKLGFCDV----G----------N-GGS
GmKaz-3         VKCFRT-DPVCSVDGVTYWCGCSEAAYASAQIAKLGFCEV----G----------N-GGS
MgKaz-1         VKCFRT-DPVCGVDGVTYWCGCAEAHCAGARVKKFGFCEV----G----------N-GGP
MtKaz-1         VKCFRT-DPVCGVDGVTYWCGCAEAACAGVKVGKMGFCEV----G----------S-GGS
MtKaz-2         AKCFRT-DPVCGADGVTYWCGCAEAACAGAKVAKLGFCEV----G----------N-GGS
GmKaz-1         AKCFRA-DPVCGADGVTYWCGCAEAACAGVEVAKLGFCEV----G----------N-GGS
GmKaz-2         AKCFRA-DPVCGADGVTYWCGCAEAACAGVEVAKFGFCEV----G----------N-GGS
CsKaz-1         VKCFRT-DPVCGVDGVTYWCGCADALCSGVKVAKMGFCEV----G----------N-GGS
FvKaz-2         VNCFRP-DPVCGVDGVTYWCGCPDAQCAGVKVAKLGFCEV----G----------N-GGS
FvKaz-1         VNCFRP-DPVCGVNGVTYWCGCQEAQCAGVKVAKLGFCEV----G----------N-GGA
PpeKaz-1        VNCFRP-DPVCGVDGVTYWCGCQEAQCAGVKVAKLGFCEV----G----------N-GGS
PpeKaz-2        VNCFRP-DPVCGVDGVTYWCGCQDAQCAGVKVAKLGFCEV----G----------S-GGS
                  *      .*. :   *      .            *

CsuKaz-1        ------PVTA--------------------------------------------------
VcKaz-2         FR----SAETSESNPRSW-------------------GLD--------------------
CrKaz-3         ------------------------------------------------------------
PpKaz-3         TALY--AVQSLQLVHMLWLVF---------------AGSF--------------------
PpKaz-2         TALY--ALHSLQLNRMFCLVI---------------AGLF--------------------
PpKaz-1         ANLGLHAAQSLQLVHMVWLVL---------------AGLL--------------------
SmKaz-1         MTIAITISSNLVGISVVRLVP---------------CGCPQQS-----------------
PpKaz-4         TAVR--AAQSLQLVHMLWLVV---------------AGLL--------------------
RcKaz-2         ------------------------------------------------------------
PtKaz-5         ------------------------------------------------------------
PtKaz-3         ------------------------------------------------------------
PtKaz-4         ------------------------------------------------------------
RcKaz-4         ------------------------------------------------------------
RcKaz-3         ------------------------------------------------------------
PtKaz-6         ------------------------------------------------------------
AtKaz-2         ASV---PGQALLLIHIVWLFL---------------LGLS--------------------
AtKaz-1         LSV---PGQALLLIHIVWMML---------------LGFS--------------------
ZmKaz-3         GAG---AGQQLLCRGLRRAAVRCGRPLLDGSGGSLQMALN--------------------
OsKaz-1         APV---SGQALLLVHIVWLFV---------------LGAA--------------------
BdKaz-1         APV---SGQALLLVHIVWLFV---------------LGAA--------------------
ZmKaz-2         APV---SGQALLLVHIVWLFV---------------LGAA--------------------
SbKaz-1         APV---SGQALLLVHIVWLFV---------------LGAA--------------------
ZmKaz-1         APV---SGQALLLVHIVWLFV---------------LGAA--------------------
PtKaz-2         SSL---PGQALLLIHIVWLIL---------------LGFS--------------------
PtKaz-7         ASL---PGQALLLIHIVWLIL---------------IGFS--------------------
TcKaz-2         GSF---PGQALLLVHILWLIV---------------LGFS--------------------
RcKaz-5         ASL---PRQALLLVHILWLIL---------------LGFS--------------------
VvKaz-1         -------GQALLLVHIVWLMV---------------LGFS--------------------
PtKaz-1         A------AQALLLVHIVWLIV---------------LGFS--------------------
RcKaz-1         A------AQALLLLHIVWLIV---------------LGFC--------------------
TcKaz-1         ASF---PGQALLLVHIVWLIL---------------LGFS--------------------
GmKaz-3         VTL---SGQALLLVHIVWLIV---------------LGFS--------------------
MgKaz-1         A------GQALLLVHIVWLIL---------------LGVF--------------------
MtKaz-1         APL---SAQAFLLLHIVWLIV---------------LAFS--------------------
MtKaz-2         ATF---PGQALLLVHIVWLIV---------------LGFS--------------------
GmKaz-1         API---PGQALLLVHIVWLIV---------------LGFS--------------------
GmKaz-2         API---PGQALLLVHIVWLIV---------------LGFS--------------------
CsKaz-1         API---PAQALLLVHILWLII---------------LGVS--------------------
FvKaz-2         AP------QALLLVHIVWLIV---------------LGISKGVGYSDFLYEQAVSQRSLI
FvKaz-1         APL---SAQALLLVHIVWLIV---------------LGFS--------------------
PpeKaz-1        APL---SAQALLLVHIVWLIV---------------LGFS--------------------
PpeKaz-2        APH---SAQALLLVHIVWLIV---------------LGFS--------------------


CsuKaz-1        ------------------------------------------------------------
VcKaz-2         ---------RI-------------------------------------------------
CrKaz-3         ------------------------------------------------------------
PpKaz-3         -----VVLGML-------------------------------------------------
PpKaz-2         -----VALRVV-------------------------------------------------
PpKaz-1         -----IVIGLL-------------------------------------------------
SmKaz-1         -----CTKGI--------------------------------------------------
PpKaz-4         -----IVLGSV-------------------------------------------------
RcKaz-2         ------------------------------------------------------------
PtKaz-5         ------------------------------------------------------------
PtKaz-3         ------------------------------------------------------------
PtKaz-4         ------------------------------------------------------------
RcKaz-4         ------------------------------------------------------------
```

```
RcKaz-3         ------------------------------------------------------------
PtKaz-6         ------------------------------------------------------------
AtKaz-2         -----LLVGGF-------------------------------------------------
AtKaz-1         -----ILFGLF-------------------------------------------------
ZmKaz-3         -----SCRDLFW------------------------------------------------
OsKaz-1         -----VLLGFL-------------------------------------------------
BdKaz-1         -----VLLGFL-------------------------------------------------
ZmKaz-2         -----VLLGFL-------------------------------------------------
SbKaz-1         -----VLLGFL-------------------------------------------------
ZmKaz-1         -----VLLGFL-------------------------------------------------
PtKaz-2         -----LLFGFF-------------------------------------------------
PtKaz-7         -----LLFGFF-------------------------------------------------
TcKaz-2         -----VLFGLI-------------------------------------------------
RcKaz-5         -----LLFGLF-------------------------------------------------
VvKaz-1         -----VLFGLF-------------------------------------------------
PtKaz-1         -----ILFGLF-------------------------------------------------
RcKaz-1         -----VLFGIF-------------------------------------------------
TcKaz-1         -----VLCGLF-------------------------------------------------
GmKaz-3         -----VFFGLF-------------------------------------------------
MgKaz-1         -----VLFGLL-------------------------------------------------
MtKaz-1         -----VFFGLF-------------------------------------------------
MtKaz-2         -----VLFGFF-------------------------------------------------
GmKaz-1         -----VLFGLF-------------------------------------------------
GmKaz-2         -----VLFGLF-------------------------------------------------
CsKaz-1         -----VLFGLF-------------------------------------------------
FvKaz-2         QRLLVLVFPVFRGTYVEHGGTFTGSSTGCVGGKTDSRFGCREDIGLQKAGAGFAAGRTLA
FvKaz-1         -----VLFGLF-------------------------------------------------
PpeKaz-1        -----VLFGLF-------------------------------------------------
PpeKaz-2        -----VLFGLF-------------------------------------------------


CsuKaz-1        ----------
VcKaz-2         ----------
CrKaz-3         ----------
PpKaz-3         ----------
PpKaz-2         ----------
PpKaz-1         ----------
SmKaz-1         ----------
PpKaz-4         ----------
RcKaz-2         ----------
PtKaz-5         ----------
PtKaz-3         ----------
PtKaz-4         ----------
RcKaz-4         ----------
RcKaz-3         ----------
PtKaz-6         ----------
AtKaz-2         ----------
AtKaz-1         ----------
ZmKaz-3         ----------
OsKaz-1         ----------
BdKaz-1         ----------
ZmKaz-2         ----------
SbKaz-1         ----------
ZmKaz-1         ----------
PtKaz-2         ----------
PtKaz-7         ----------
TcKaz-2         ----------
RcKaz-5         ----------
VvKaz-1         ----------
PtKaz-1         ----------
RcKaz-1         ----------
TcKaz-1         ----------
GmKaz-3         ----------
MgKaz-1         ----------
MtKaz-1         ----------
MtKaz-2         ----------
GmKaz-1         ----------
GmKaz-2         ----------
CsKaz-1         ----------
FvKaz-2         WKTKAGAVAW
FvKaz-1         ----------
PpeKaz-1        ----------
PpeKaz-2        ----------
```

B. I3 Kunitz-P.

```
AtKun-2      -----------------------MKNPSVIS---FLIILLFAATICTHGN----------
AtKun-1      -----------------------MKATISIT---TIFLVVALAAPSLARP----------
MtKun-7      ------------------------------------------------------------
MtKun-11     ---------------NVDDSVRICQSTAVKCLERFAIDVYHIFEAKYLGMPTNEYIERVL
MtKun-9      -----------------------MKPTLVTT---LCFLLFSFTIYFPLPF---------T
MtKun-14     -----------------------MKPTLVTT---LCFLLFSFTIYFPLPF---------T
MtKun-20     -----------------------MKSTLFT----FSLLLFSFTYFPLAFT----------
MtKun-24     -----------------------MKSTLF-----TFSLLFSFTYFPLAFT----------
MtKun-15     -----------------------MKPTFLT----LSLILFALTICFSLAF----------
MtKun-16     -----------------------MKPTFLTT---LSLLLFALATYFPLAF----------
MtKun-19     -----------------------MNSVCSLTILSLSFLLFVFITNLSLVF---------S
MtKun-17     -----------------------MNPILSLT---ISFFLFAFITNLSPNN----------
MtKun-22     -----------------------MKHVLSLT---LSFLLFVFTTNLSLAF---------S
MtKun-25     -----------------------MKHLLSLT---LSFFIFVFITNLSLAT----------
AtKun-3      ----------------------MEKLTLSFI---TLTVLSAIFTAASAAD----------
BdKun-1      -----------------------MKILLPL----LQVLAIALLCLANDAT----------
MtKun-1b     EPYYVRFQKASTFAQSNNQDLKTMKFIQLCF---LISLLVLLNTKALQGA----------
MtKun-1a     -----------------------MKFIQLFC---FLSLLVLFNTKPLQGA----------
MtKun-4      -----------------------MKFIQLFC---FLSLSALFNTKALQGD----------
MtKun-2      ------------------------MSIRPLT---IFIIAHVWLFMITTSV----------
MtKun-3      ------------------------MSTRSLT---IFIIAHVLLFMITTSV----------
MtKun-26     -----------------------MSMRLSI----RTLIILAHVCLFITTT----------
FvKun-3      ----------------------------------SMISSIFMAVETINQT----------
FvKun-4      -----------------------MMKSMKMI---GSLSCCMWLVMAMVTS----------
FvKun-5      -----------------------MSMKLIG----SIWLVMAMATVAQADN----------
MtKun-27     ---------------------MSTTLIKITS---LSLMLCLFMSIKTLAQ----------
MtKun-28     ---------------------MTTRMIKITS---LSLMLWLFMTIPTSAQ----------
BdKun-2      -------------------MSSSPRARLLLI---SLVAAATLLVSCRGAA---------A
OsKun-1      -----------------------MVSLRL-----PLILLSLLAISFSCSA----------
SbKun-1      --------------------MGIPRAARLLV---LVSVLAAISLSCGAAA----------
ZmKun-1      ------------------MPMSIPRAAHLLV---LLSVLAISLSSCGAAA----------
AtKun-4      -----------------------MTKTTKTMNPKFYLVLALTAVLASNAY----------
MtKun-5      -----------------------MKLHFPL----LFLLLTLFTTKPLQGA----------
MtKun-8      -----------------------MKSTFL-----AFLLLIALTSQPLLSS----------
MtKun-12     -----------------------MKNTLLA----FFFLFTFLSSQPLLGA---------A
MtKun-21     -----------------------MKNTLLA----FFFLFTFLSSQPLLGA---------A
MtKun-10     -----------------------MKCTIML----ALLLLLALSSQPLFVS---------S
MtKun-18     -----------------------MKGTML-----AFLLLFALSSQPLLGS---------A
MtKun-13     -----------------------MKTSFLA----FSILCLAFICKTIAA-----------
MtKun-23     -----------------------MKTSFLA----FSIIFLAFICKTFAA-----------
AtKun-5      -------------------------MSSLLY---IFLLLAVFISHRGVTT----------
MtKun-6      -----------------------MKSICI-----LLAVLFALSTQPLLGE---------A
FvKun-1      -------------------MRFCTLASQVLI---CYLSLLAFRGKPLLVA-------VHA
FvKun-2      -----------MFLSRFPAVRLLTLASQVLI---CYLSLLAFRGKPLLVA--------VH
VvKun-1      -----------------------MKTSSLLL---FSLFLIALAFNPLPGA---------A
VvKun-4      -----------------------MKTTLFL----FSLLLIALAVKPFPVA---------A
VvKun-2      -MLSKTSSRHQMVSHISSKRSTTMKTTSLL----FSLLLIALAVKPFSVA---------A
VvKun-3      ------------------------------------------------------------


AtKun-2      ----EPVKDTAGNPLNTREQYFIQPVKTE--SKNG------GGLVPAAITVL-----PF-
AtKun-1      ---DNHVEDSVGRLLRPGQTYHIVPAN----PETG------GGIFSNS-EEI--------
MtKun-7      -------MDTLGAPLRSGESYQISVVV----ADHP------GALTIGK-TDD-----LD-
MtKun-11     QMGEAQLRDKNGNPILVSKKYFIWPA-----DGSG------GGLRLNE-TEQ--------
MtKun-9      HANDFIVKDIFGNPVVPSGSYYIWPDY----LVSG------GELRLGE-TEN-----ST-
MtKun-14     HANKIIVKDIFGNPVVPSGSYYIWPDY----LING------GELRLGE-TEN-----ST-
MtKun-20     ----ETVEDINGNPVFPGGKYYIAPLIS---KGGG------GGLKLGK-TGD-----SE-
MtKun-24     ----ETVEDINGNPVFPGGKYYIAPLIS---KGGG------GGLKLGK-TGD-----SE-
MtKun-15     AQVSEQVFDTNGNPIFPGGTFYIMPSIF---GAAG------GGLRLGK-TKN-----SK-
MtKun-16     ---TEQVRDSNGNPIFFSSRFYVKPSIF---GAAG------GGVKLGE-TGN-----SS-
MtKun-19     NDNVEIVVDKNGIPLIPGTSYYISPAN------TG------GRITLGK-TVD-----SD-
MtKun-17     --AAKQVLDIHGTPLIPGSQYYIFPASE---NPNS------GGLTLNK-VGD-----LE-
MtKun-22     NDAVEQVLDINGNPIFPGGKYYILPAIR---GPLG------GGLRLGK-SSN-----SD-
MtKun-25     SNDVEQVLDINGNPIFPGGQYYILPALR---GPGG------GGVRLGR-TGD-----LK-
AtKun-3      ATPSQVVLDIAGHPVQSNVQYYIIPAK----IGTG------GGLIPSN-RNL-----STQ
BdKun-1      ----QLAHDTQGKTLSSRESYYILPAK----QGSG------GGLTATP-NGQ-----R--
MtKun-1b     --KPDRVVDRHGKPLESGKGYYVWQFW----AHDI------GGLTLSS-TRN-----KT-
MtKun-1a     --EPEAVVDKQGNPLKPGEGYYVFPLW----ADN-------GGITLGH-TRN-----KT-
MtKun-4      --KPEAVVDKQGNPLKPGEGYYVFPLW----ADN-------GGITLGQ-TRN-----KT-
MtKun-2      ---AQIVIDTSGEPVEDDEEYFIRPAI----TGNG------GGSILVTRNGP--------
MtKun-3      ---AQIVIDTSGEPVEDDEEYFIRPAI----TGNG------GGSILVTRNAP--------
MtKun-26     -TIAQFVLDTVGEPVEGDEEYFIRPVI----TNKG------GRSTMVSRNES--------
FvKun-3      ----SPVFDTSGKALKSGIDYYIKPAG----TDKG------GRLTLIN-REP-----KL-
FvKun-4      AQDNAPVLDTSGQALQSGVDYFIKPAI----TDIG------GRFTLID-RND-----S--
FvKun-5      ----AAVLDTTGQALQSGVDYYIKPAI----TDNG------GRFTLID-RNN-----S--
MtKun-27     -SENEKILDTKGHPLERGKEYYIKPAI----TDSG------GRFTLID-RNG-----S--
MtKun-28     -PENEEVLDLTGRPLESGRKYYIRSDV----SDFG------GRITLVN-KNG-----S--
BdKun-2      SPPPPPVYDTDGHELRADTSYHVLPLI----RRGGPYGARRGGLTLAPLHGG-----QR-
OsKun-1      --APPPVYDTEGHELSADGSYYVLPASP----GHG------GGLTMAP-RVL-----P--
SbKun-1      -AALPPVYDTDGHELSADADYYVLPVPHG--SGSG------GGLTMAP-NGL-----R--
ZmKun-1      ---ALPVYDTDGHELSADADYYVLPAPPRGSGGGG------GGLTMAP-KGL-----HP-
```

```
AtKun-4         ----GAVVDIDGNAMF-HESYYVLPVI----RGRG------GGLTLAG-RGG-----QP-
MtKun-5         -EQPEEVRDTSGNLVRNSINYFILPSSIQ--CGTR------CEMALLNTNKT--------
MtKun-8         --SLEHVVDITGKNLRANAYYNVLLSMPYTNSRSP------EGLGLSNNIGQ-----P--
MtKun-12        EASNEQVVDTLGKKLRADANYYIIPVPIYKCGPYGKCRSS-GSSLALA-SNG-----KT-
MtKun-21        EASNEQVVDTLGKKLRADANYYIIPVPIYKCGPYGKCRSS-GSSLALA-SIG-----KT-
MtKun-10        KTSLNQVLDISGKKLRTDSDYYIIPA-------NG------GDISLES-SIG-----ES-
MtKun-18        EASPDQVIDTSGKKLRADTNYYIIPAKPFTTCGFVSCFNS-GGIALET-VGE-----S--
MtKun-13        ---PEPVLDISGKQLTTGVKYYILPVI----RGKG------GGLTVAN-HGE-----NNQ
MtKun-23        ---PEPVLDISGKKVTTGVKYYILPV----IRGKG------GGLTVVN-ENNLNGNNNT-
AtKun-5         EAAVEPVKDINGKSLLTGVNYYILPVI----RGRG------GGLTMSN-LKT-----ET-
MtKun-6         DASPEQVVDTEGKKVRAGVDYYIRPVPTTPCDGRGPCVVGSGFVLIAR-SPN-----ET-
FvKun-1         DSDPAPVLDIKGHNIQTGVEYWILPVI----RGRG------GGLTLAT-TGN-----RNR
FvKun-2         AADPAPVRDIKGDNLQTGVEYWILPVI----RGKG------GGLTLAS-NRN-----KTI
VvKun-1         EAAPDPVLDIEGKQLRSGVDYYILPVI----RGRG------GGLTVAS-VRN-----KT-
VvKun-4         ESSPDPVLDTEGKQLWSGVDYYILPVI----RGRG------GGLTLAS-TGN-----EN-
VvKun-2         ESAPDPVLDTEGKKLRSGVDYYILPVF----RGRG------GGLTLAS-TGN-----ET-
VvKun-3         --------------MSGVDYYILPVF----RGRG------GGLTLAS-TGN-----ET-
                                    : :

AtKun-2         --C-PLGITQTLLPYQPGLPVSF-VLALG--------VGST----VMTSSA-VNIE-FKS
AtKun-1         --C-PLDIFQSNNPLDLGLPIKF-KS-----------ELWF----VKEMNS-ITIE-FEA
MtKun-7         --C--VYLVSSQDDSSHGLSVKF----------------------HSTD------ILG
MtKun-11        --C-PLVVQQAFSEDVKSLPLKF-IPTEN--------INDF----IFTGYTSLDIV-FEK
MtKun-9         --C-PFTVLQDYSNLGPGLPVKF-TPQNQ--------TSGDDP--ITLSLH-IDIA-FEN
MtKun-14        --C-PFTVLQDYSNLGPGLPVKF-TPQNQ----TS--SDDP----ITLMLP-IEIT-FEN
MtKun-20        --C-PVTVLQDFSEVVRGLPVRF-TIIV---------KRGV----IFTTDE-VDIE-FVK
MtKun-24        --C-PVTVIQDFSEVVRGFPVRF-IIRV---------RRGV----IFTTDE-LDIE-FVK
MtKun-15        --C-PLTVLQDYSEVVNGLPVKF-TRLEA--------GHDI----ISTNTA-LDIA-FTT
MtKun-16        --C-PLTVLQDYSEVVNGLPVKFSTDAEI--------FIDL----ISTDTSRVDIV-FPE
MtKun-19        --C-SFLVLQDDEKMIYGRQVKF-SLSVG----II--PASL----IFTNTA-LDIE-FVY
MtKun-17        --C-PVTVLQNNAMI--GLPVKFTVPEN---------NTGN----ILTGTD-LEIE-FTK
MtKun-22        --C-EVTVVQDYNEVINGVPVKF-SIPEI--------SPGI----IFTGTP-IDIE-FTK
MtKun-25        --C-PVTVLQDRREVKNGLPVKF-TIPGI--------SPGI----IFTGTP-LEIE-YTK
AtKun-3         DLCLNLDIVQSSSPFVSGLPVTF-SPLNT--------KVKH----VQLSAS-LNLE-FDS
BdKun-1         --C-LAFVFQVRDETFLGDPLRF-TPLPP--------NHSADEP-IRLSTD-IWIE-FRN
MtKun-1b        --C-PLDVIRNPKELGS--PVYFSAP-----------GFKH----IPTQTD-LSIK-IRF
MtKun-1a        --C-PLDVIRNPDAIGT--PVYF-SAS----------GLDY----IPTLTD-LTIE-IPI
MtKun-4         --C-PLDVIRNPEAIGS--PVYF-YEY----------EHDY----IPTLTD-LTVE-IPI
MtKun-2         --C-PLHVGLGNSEGTLGMAVKF-TPFAP--------RHDDDDDDVRLNRD-LRVT-FQG
MtKun-3         --C-PLHVGLGNSEGTLGVAVKF-TPFAP--------RHDDDDDDVRLNRD-LRVT-FQG
MtKun-26        --C-PLHVGLELTGLGRGLVVKF-TPFAP--------HHDFDD--VRVNRD-LRIT-FQA
FvKun-3         --S-PLYVGQGNIS---GLPFHF-KPFFT--------GETV----VRESRD-QMII-SSV
FvKun-4         --C-PLYVGQKNTSGSEDFPVTF-APFVE--------GETV----VREGRD-QKIT-FSA
FvKun-5         --C-PLYVGQENTSGPEGFPVIF-APFEE--------GETV----VREGRD-QKIT-FSA
MtKun-27        --C-PLYVGQENTDLGKGLPVIF-TPFAK--------EDKV----IKDSRD-FKVK-FSA
MtKun-28        --C-PLYVGQETTDFGQGLSVIL-TPLEN--------DDTV----VKVNRD-FKVK-FSS
BdKun-2         --C-PLFVAQDASREHLGLPVRF-APHGKASASAD--PTTV----VRVSTD-VRVS-FRA
OsKun-1         --C-PLLVAQETDERRKGFPVRF-TPWGG----AAAPEDRT----IRVSTD-VRIR-FNA
SbKun-1         --C-PLFVAQEADPLRKGFPVRF-TPLPQ----Q---GDRT----VRVASD-VGVH-FAA
ZmKun-1         --C-PLFVAQETDPLRKGFPVRF-APLQQ--------DQGGSDRAVRVSSD-VGVH-FAA
AtKun-4         --C-PYDIVQESSEVDEGIPVKF-SNWRL--------KVAF----VPESQN-LNIE-TDV
MtKun-5         --C-PLDVVEEEEAM----QFSF-VPFNF--------KKGV----IRVSTD-LNVI-HSF
MtKun-8         --C-PLDVIV--VSRYQSLPIRF-TPLNL--------KKGV----IRVSSD-LNIM-FRS
MtKun-12        --C-PLDVVV--VDRYQALPLTF-IPVNP--------KKGV----IRVSTD-LNIK-FSS
MtKun-21        --C-PLDVVV--VDRYQALPLTF-IPVNP--------KKGV----IRVSTD-LNIK-FSS
MtKun-10        --C-PLHVVVVKHRQGLGFPLRL-AP-----------VKGD----IRVSTD-LNIM-LGN
MtKun-18        --C-PLDVVV--VKHNQGLPLRF-TPVNN--------KKGV----VRVSTD-LNIK-FSN
MtKun-13        T-C-PLYVVQEKLEVKNGEAVTF-TPYNA--------KQGV----ILTSTD-LNIKSFVT
MtKun-23        --C-PLYVLQEKLEVKNGQAVTF-TPYNA--------KKGV----ILTSTD-LNIKSYVT
AtKun-5         --C-PTSVIQDQFEVSQGLPVKF-SPYD---------KSRT----IPVSTD-VNIK-FS-
MtKun-6         --C-PLNVVV--VEGFRGQGVTF-TPVNP--------KKGV----IRVSTD-LNIK-TSL
FvKun-1         SIC-PLDVVQEQQEVSNGLPLTF-SPVNL--------KIGV----VRLSTD-LNIK-FST
FvKun-2         G-C-PLDVVQEQSEVSNGLPLTF-SPVNL--------TKGV----VRLSTD-LNIK-FST
VvKun-1         --C-PLDVVQDKLEVSHGLPLTF-TPVNP--------KQDV----IRVSTD-HNIK-FSA
VvKun-4         --C-PLDVVQEQHEVSNGLPLTF-TPVNP--------KKGV----IRVSTD-HNIK-FSA
VvKun-2         --C-PLDVVQEQQEVSNGLPLTF-TPVNP--------KKGV----IRVSTD-HNIK-FSA
VvKun-3         --C-PLDVVQEQQEVSNGLPLTF-TPVNP--------KKGV----IRVSTD-HNIK-FSA
                  .    :           . :

AtKun-2         NIW-PFCKEFSK-FWEVDDSSS-----------APKEPSILIGGKMGDR------NSSF
AtKun-1         PNW-FLCPKESK-GWRVVYSEE-----------FKKSLIISTGGSSNPSG--------F
MtKun-7         S---PACVESA--KWLVFVDSELD----------PLPIHYVGIGGPENYPSHTEIFDGTF
MtKun-11        K---TKCAESS--KWVVVKG-------------GFMEPWIGIGGGVNGKSV---IDGLF
MtKun-9         K---PDCAESS--KWLVVEAEN-----------EYPTPWLAIDGTGKKVYD----DGWF
MtKun-14        K---PDCAESS--KWLVVEAEN-----------EYPTPWVTIDGTNKNVY-----DGYF
MtKun-20        K---PKCAESA--KWVLAHD-------------DFPTSWVGIGDNIDAF------QGKF
MtKun-24        K---PKCAESA--KWVLAHD-------------DFPTSWVGIGDNIDA------FQGKF
MtKun-15        K---PDCAESS--KWVLVDDFN-----------KLTGPWVGIGGTEDNEDI--------
MtKun-16        K---PECAESS--KWLLIED-------------DFPRPWVGIGGIEDYIGK-HIIDGKF
MtKun-19        K---DSCVESS--KWLIFVDNV-----------NNNKSFVGIGGPENYPQGTQILNGKF
MtKun-17        K---PDCAESS--KWLMFLDH------------NTQLSCVGIGGATNYHGI-ETISGKF
MtKun-22        K---PNCVESS--KWLIFVDS------------VIQKACVGIGGPENYPGF-RTLSGTF
MtKun-25        K---PSCAAST--KWLIFVDN------------VIGKACIGIGGPENYPGV-QTLKGKF
```

```
AtKun-3         TV--WLCPDSK--VWRIDHSV------------QLRKSFVSIGGQKGKG------NSWF
BdKun-1         LS--NFCVERL--DWHLTNKSP-----------ETAGLHVAAGNEDGTRS-----FGLF
MtKun-1b        RS--SSCNQSK--VLKLSKE-------------GSGFWFLSTGGVAGDV------VSKF
MtKun-1a        LG--SPCNEPK--VWRLLKV-------------GSGFWFVSTGGAAGDL------VSKF
MtKun-4         LG--SPCSERK--VWKISKEGT-----------RARFWFVSTGGFPGNL------FSQF
MtKun-2         F---TGCGQST--DWRLGEKDA-----------TSGRRLIVTGRDNGAGSH----GNFF
MtKun-3         F---TGCGQST--DWRLGEKDA-----------TSGRRLIVTGRDNGAGSH----GNFF
MtKun-26        S---SSCVQST--EWRLGEKDT-----------KSGRRLIITGTDSATNGS---YGNFF
FvKun-3         S---THCRRFS--SWSIVTANA-----------EDQRKLIVMREPARGKVP-DSI--YF
FvKun-4         V---TTCVQST--TWKVGEKQS-----------ETQRRLIVTG-IDQNEGIAGPAGNYF
FvKun-5         S---TTCVQST--TWKLGEKQA-----------ETQRRLIVTGSDEVESPR-GATSNYF
MtKun-27        S---SICVQST--EWKLGDRDT-----------KSGRRVIIAGSD----------GSYF
MtKun-28        S---SSCGQST--EWKLGDRDN-----------RSGRRLIIAGSD----------GNSF
BdKun-2         Y---TTCVQST--EWHVESGKNI----------LGARRHVVTGPVVGGPSPISGRENAF
OsKun-1         A---TICVQST--EWHVGDEP------------LTGARRVVTGPLIGPSPSGR--ENAF
SbKun-1         V---TTCVQTT--EWHVSGDGDALATAPAPAPVLSSGRRLVLTGPVR-SPSP-NGREKVF
ZmKun-1         A---TTCVQTT--EWHVSGDGAAP---------RSGRRLVLTGPVLSSSP--NGREKVF
AtKun-4         GA--TICIQST--YWRVGEFDH-----------ERKQYFVVAGPKPEGFGQ-DSLKSFF
MtKun-5         P---TNCSTSSVTVWKVDKVDV-----------ATSQRFVTTGGVQGNPGR-ETVDNWF
MtKun-8         N---SSCPYHTT-VWKLDRFDA-----------SKGKSFVTTDGFIGNPGP-QSISNWF
MtKun-12        R---ATCLHHSM-VWKLDRFNV-----------SKRQWFITIGGVAGNPGW-ETINNWF
MtKun-21        R---ATCLHHSM-VWKLDRFNV-----------SKRQWFITIGGVAGNPGW-ETINNWF
MtKun-10        YD--DRCPNYSV-VWKIDPY-------------SKEATFVTTNGILGHPGS-NSIHSWF
MtKun-18        DAYDSRCPNHSL-VWKIDPF-------------SKEETFVTTNGVLGNPGS-NTIHNWF
MtKun-13        K---TKCPQTQ--VWKLLKE-------------LTGVWFLATGGVEGNPSM-ATVGNWF
MtKun-23        K---TTCAQSQ--VWKLNKV-------------LSGVWFLATGGVEGNPGF-DTIFNWF
AtKun-5         ----PTS------IWELANFDE-----------TTKQWFISTCGVEGNPGQ-KTVDNWF
MtKun-6         N---TSCEEST--IWTLDDFDS-----------STGQWFVTTGGVLGNPGK-DTVDNWF
FvKun-1         SAAVTICLNTSK-VWKLDNLDE-----------STGQRFVTTGGVEGNPGP-KTISNWF
FvKun-2         SAAVTLCPQTSK-VWKLDEVDE-----------STGQRSVTTGGVEGNPSP-KTLMNWF
VvKun-1         A---TICAQST--VWKLEYDE------------STGQRFITTGGVEGNPGR-GTLSNWF
VvKun-4         S---TICVQST--LWKLEYDE------------SSGQRFVTTGGVEGNPGH-ETLDNWF
VvKun-2         S---TICVQST--LWKLEYDE------------SSGQRFVTTGGVEGNPGR-ETLDNWF
VvKun-3         S---TICVQST--LWKLEYDE------------SSGQRFVTTGGVEGNPGR-ETLDNWF
                      .         :                      :

AtKun-2         KIEK--AGEGARANV------YKLTTFYGT----------VGAIP-GVWL----------
AtKun-1         QIHR--VDGGA----------YKI-VYCTNISTTT-----CMNV--GIFT---DIS----
MtKun-7         SIQR--SELFPLA--------YTL-NYCRMDH--------CSYV--GINKVLIGNE----
MtKun-11        KIETIRSFRG-----------YKL-VFCPTISDPTGQ---CNNI--GRFF---DNE----
MtKun-9         EIIG--YKKTG----------YLI-YFCHK-LSPTLG-E-CIYL--SRKN---DKN----
MtKun-14        MIVG--FKKTG----------YLI-FFCHKLLSPTPGV--CIYL--SRRN---DEN----
MtKun-20        KIET--LGSGSGA--------YKL-VYCPL-FSAPPG-A-CSDI--GRYR---DEN----
MtKun-24        KIET--LGSGSGA--------YKL-VYCPL-FSAPPG-A-CSDI--GRYR---DEN----
MtKun-15        ------------------------TAPPG--------A-CYDI--GRH----DDF----
MtKun-16        KIVK--HGFG-----------YKL-VFCPT-FTAPPG-L-CHDI--GRY----DDK----
MtKun-19        NIKK--SGSENA---------YKF-GFCVKETPS------CWDI--GRYM-SIGEE----
MtKun-17        LIVK--HGSGHV---------YRL-GFCLDVTG-----D-CGYIGLQMFN---SEE----
MtKun-22        NIEK--HESGFG---------YRL-GYCVKDSP-----T-CLDI--GRAHEEVEDE----
MtKun-25        NIQK--HASGFG---------YNL-GFCVT-GSP----T-CLDI--GRFD---NDE----
AtKun-3         QIQE--DGDA-----------YKL-MYCPI-----SSIVACINV--SLEI---DDH----
BdKun-1         RIER--HGTDTTG--------YKL-MSCAKKGS-------CRYL--GLHV---FKGMNWL
MtKun-1b        KIEK--LEGDTGIPI------YIF-KFCPS----VPGAL-CAPV--RTFT---DTD----
MtKun-1a        KIER--LAGEHAYEI------YSF-KFCPS----VPGVL-CAPV--GTFV---DTD----
MtKun-4         KIER--LEGEHAYEI------YSF-LYCPS----VPGTL-CAPV--GTFV---DTD----
MtKun-2         RIVQ--TQTGGI---------YNI-QWCPTEACPSCKVQ-CGTV--GVIR----EN----
MtKun-3         RIVQ--TQTGGI---------YNI-QWCPTEACPSCKVQ-CGTV--GVIR----EN----
MtKun-26        RIVE--TPLEGM---------YNI-QWCPTEVCPSCKFE-CGTV--DMLN----EN----
FvKun-3         RITRSQPEVDGNS--------YIL-RWCPTDVCPNCTFADCGNI--GSLV---ASR----
FvKun-4         RINKDADIDGV----------YSL-QWCPTELCPTCRFI-CGDV--GALL----EN----
FvKun-5         KINKQADFDGI----------YYL-QWCPTEVCPICKFI-CGNV--GALV----EN----
MtKun-27        RIVK--AEFEGV---------YNI-RFCPTDTCSFCRFD-CGFV--GGLR----EN----
MtKun-28        RILKISFGIEGVIGN------YNI-RFCPSDTV-----N-CGTV--GNLR----EN----
BdKun-2         RVER--VERYGGVATDDEAREYKL-MWCGDE------AA-CQAL--GVFR---DGE----
OsKun-1         RVEK--YGGG-----------YKL-VSCRD--------S-CQDL--GVSR-----D----
SbKun-1         RVEK--HGRGG----------YKL-VWCGGAASGSTSSS-CQDL--GVFR---DDG----
ZmKun-1         RVEK--HSRG-----------YKL-VWCGGSSASTS----CQDL--GVFR---DDG----
AtKun-4         KIEK--SGEDA----------YKF-VFCPR-TCDSGNPK-CSDV--GIFI---DEL----
MtKun-5         KIER--FESG-----------YKL-VFCPT-VCRECEVV-CKDI--GIFL---DEN----
MtKun-8         KIEK--YVEG-----------YKL-VYCPI-VCPSCKHE-CKNV--GLFE---DEN----
MtKun-12        KIEK--YGDA-----------YKL-VFCPS-VVQSFKHM-CKDV--GVFV---DEN----
MtKun-21        KIEK--YGDA-----------YKL-VFCPS-VVQSFKHM-CKDV--GVFV---DEN----
MtKun-10        KIEK--YEDA-----------YKL-VYCPN-VCPSCNHV-CKDI--GIYK---YKN----
MtKun-18        QIEK--YEDA-----------YKL-VYCPN-VCPSCKHV-CKDI--GIYV---YKY----
MtKun-13        KIEK--ADKD-----------YVL-SFCPAEAC-KCQTL-CREL--GLFV---DDK----
MtKun-23        KIEK--ADKD-----------YVF-SFCPS-VC-KCQTL-CREL--GLYV---YDH----
AtKun-5         KIDK--FEKD-----------YKI-RFCPT-VCNFCKVI-CRDV--GVFV----QD----
MtKun-6         KIEK--YEDD-----------YKF-VFCPT-VCNFCKVM-CRNV--GIFR---DSN----
FvKun-1         KIEK--YDKD-----------YKL-VFCPT-VCNFCKVI-CGDV--GIYL----EG----
FvKun-2         KIEK--YDDD-----------YKL-VFCPK-VCRFCRVI-CGDV--GVHL----ES----
VvKun-1         KIEK--YGDD-----------YKL-VFCPT-VCNFCKVI-CRDV--GVYI----QK----
VvKun-4         KIEK--YEDD-----------YKL-VFCPT-VCDFCKPV-CGDI--GIYI----QN----
VvKun-2         KIEK--YEDD-----------YKL-VFCPT-VCDFCKPV-CGDI--GIYI----QN----
```

```
VvKun-3         KIEK--YEDD-----------YKL-VFCPT-VCDFCKPV-CGDI--GIYI----QN----
                                                            :

AtKun-2         -------------------SAPQLIITKDTAK--TLLVKFKKVDDATTATSNLYFPG---
AtKun-1         -------------------GARRLALTSDE----ALLVKFQK-AATPKADLKTKLRMFPF
MtKun-7         -------------------SDRRLMLRQ------AIVVVFEHDRFSL-------------
MtKun-11        -------------------NGLRLIM-SENFK--PFEVVFVD-VEDTAGFGRSVV-----
MtKun-9         -------------------GMRLVYEMDGD----ALAAVFVNINDAARARRSSAI-----
MtKun-14        -------------------GMRLVYEMDGD----ALGAVFVN-VNDAARARRSSMRNVSA
MtKun-20        -------------------GWRLVPTEND-----PFRVVFID-ATESEKAVV--------
MtKun-24        -------------------GWRLVPTEND-----PFRVVFVD-ATESEKAVV--------
MtKun-15        -------------------TGRLLVLANND----PYEVVFVD-AMGN-------------
MtKun-16        -------------------NGRRLILTEDD----PYEVVFEHVAIGTERSVV--------
MtKun-19        -------------------GGRRLSFNATE----DFEAVFA--AIAT-------------
MtKun-17        -------------------GGSRLFLTAVD----AYSVVFVD-ANGNSALSI--------
MtKun-22        -------------------GGSRLHLTHQV----AFAVVFVD-AASYEAGIIKSVA----
MtKun-25        -------------------AGRRLNLTEHE----VYQVVFVD-AATYEAEYIKSVV----
AtKun-3         -------------------GVRRLVLSTDQ----SFVVKFQK-AYDSNSNCNLKSNSRMF
BdKun-1         TSQSLAYRQCLQGWQIAVSAPMFWFFATP-----PSTLNFRK-PDDGGIYRGPAWPAGFG
MtKun-1b        -------------------GTKVMAV-GDGNDLEPYYVRFQR-VSTFTPKNNMADQDSID
MtKun-1a        -------------------GTKVMAVGDGIE-----------------------------
MtKun-4         -------------------GTKVMALGAGIEE--PYYVRFQK-ASTFTQNKNQDFSGV--
MtKun-2         -------------------GKILLALDGG-----ALPVVFQK-E----------------
MtKun-3         -------------------GKILLALDGG-----ALPVVFQK-E----------------
MtKun-26        -------------------GKILLALDGG-----PLPLVFQK-E----------------
FvKun-3         -------------------GKRFLALNGS-----ELPVVFER-ARG--------------
FvKun-4         -------------------GKRLLALDGS-----ALPVVFER-A----------------
FvKun-5         -------------------DKRLLALDGS-----VLPVVFER-A----------------
MtKun-27        -------------------GKILLALDGG-----VLPVVFECYTKNKRKLKQEPKNNLRS
MtKun-28        -------------------GKILLALDDRN----VLRVGFER-A----------------
BdKun-2         -------------------G-GAWFLGAAAEQ--VHVVVFQK-APSV-------------
OsKun-1         -------------------GARAWLGASQP----PHVVVFKK-ARPSPPE----------
SbKun-1         -------------------DRRAWLGTTETDR--AHAVKFEK-AATVHA-----------
ZmKun-1         -------------------DRRAWLGTTETDR--AHAVVFQK-DTTMHA-----------
AtKun-4         -------------------GVRRLALSDK-----PFLVMFKK-ANVTEVSSKTM------
MtKun-5         -------------------RNTRFVL-SDF----PFGVKFQR-ACCE-------------
MtKun-8         -------------------GNKRLAL-SDV----PYQVKFVK-V----------------
MtKun-12        -------------------GNKRLAL-SDV----PLKVKFQQ-A----------------
MtKun-21        -------------------GNKRLAL-SDV----PLKVKFQQ-A----------------
MtKun-10        -------------------REMRLAL-TNV----PLKIKFQQ-A----------------
MtKun-18        -------------------REMRLAL-TNV----PFKVKFQK-A----------------
MtKun-13        -------------------GNKHLALSDQIP---SFRVVFKR-A----------------
MtKun-23        -------------------GKKHLALSDQVP---SFRVVFKR-A----------------
AtKun-5         -------------------GKRRLAL-SDV----PLKVMFKR-AY---------------
MtKun-6         -------------------GNQRVAL-TDV----PYKVRFQPSA----------------
FvKun-1         -------------------GVRRLVL-SKV----PLKVMFKR-V----------------
FvKun-2         -------------------GVRRLAL-SDVIV--PLKVMFKR-V----------------
VvKun-1         -------------------GYRRLALTTDA----PFRVMFKK-A----------------
VvKun-4         -------------------GYRRLAL-SDV----PFKVMFKK-A----------------
VvKun-2         -------------------EYRRLAL-SDV----PFKVMFKK-A----------------
VvKun-3         -------------------GYRRLAL-SDV----PFKVMFKK-A----------------


AtKun-2         ------------------------------------------------------------
AtKun-1         Y-----------------------------------------------------------
MtKun-7         ------------------------------------------------------------
MtKun-11        ------------------------------------------------------------
MtKun-9         ------------------------------------------------------------
MtKun-14        DTLTLQLLSQYREKEDITKHATDGLEVGIEDYAMIVQDRDIQVAGGDLWVIPKGCTKAAD
MtKun-20        ------------------------------------------------------------
MtKun-24        ------------------------------------------------------------
MtKun-15        ------------------------------------------------------------
MtKun-16        ------------------------------------------------------------
MtKun-19        ------------------------------------------------------------
MtKun-17        ------------------------------------------------------------
MtKun-22        ------------------------------------------------------------
MtKun-25        ------------------------------------------------------------
AtKun-3         LFL---------------------------------------------------------
BdKun-1         ------------------------------------------------------------
MtKun-1b        PSKMIGGRRAMTQSHRRERQSGHIPNRGRGRRVDGSGSSQEASQATQSEESQVVDPSQQM
MtKun-1a        ------------------------------------------------------------
MtKun-4         ------------------------------------------------------------
MtKun-2         ------------------------------------------------------------
MtKun-3         ------------------------------------------------------------
MtKun-26        ------------------------------------------------------------
FvKun-3         ------------------------------------------------------------
FvKun-4         ------------------------------------------------------------
FvKun-5         ------------------------------------------------------------
MtKun-27        HKRKLNKMFRPTLDLL--------------------------------------------
MtKun-28        ------------------------------------------------------------
BdKun-2         ------------------------------------------------------------
OsKun-1         ------------------------------------------------------------
SbKun-1         ------------------------------------------------------------
```

```
ZmKun-1         ------------------------------------------------------------
AtKun-4         ------------------------------------------------------------
MtKun-5         ------------------------------------------------------------
MtKun-8         ------------------------------------------------------------
MtKun-12        ------------------------------------------------------------
MtKun-21        ------------------------------------------------------------
MtKun-10        ------------------------------------------------------------
MtKun-18        ------------------------------------------------------------
MtKun-13        ------------------------------------------------------------
MtKun-23        ------------------------------------------------------------
AtKun-5         ------------------------------------------------------------
MtKun-6         ------------------------------------------------------------
FvKun-1         ------------------------------------------------------------
FvKun-2         ------------------------------------------------------------
VvKun-1         ------------------------------------------------------------
VvKun-4         ------------------------------------------------------------
VvKun-2         ------------------------------------------------------------
VvKun-3         ------------------------------------------------------------


AtKun-2         ------------------------------------------------------------
AtKun-1         ------------------------------------------------------------
MtKun-7         ------------------------------------------------------------
MtKun-11        ------------------------------------------------------------
MtKun-9         ------------------------------------------------------------
MtKun-14        ANVCGIGGLIGVKYKGDKGNIFNVLSKEVRRSFREEVGRIFFCGGLLSSTIDGASGGLLT
MtKun-20        ------------------------------------------------------------
MtKun-24        ------------------------------------------------------------
MtKun-15        ------------------------------------------------------------
MtKun-16        ------------------------------------------------------------
MtKun-19        ------------------------------------------------------------
MtKun-17        ------------------------------------------------------------
MtKun-22        ------------------------------------------------------------
MtKun-25        ------------------------------------------------------------
AtKun-3         ------------------------------------------------------------
BdKun-1         ------------------------------------------------------------
MtKun-1b        MMTLQQLLPGGVTCGPFLSWRIDKPIFVALLCQSCGVATLVRKLRVVKPINHGES-----
MtKun-1a        ------------------------------------------------------------
MtKun-4         ------------------------------------------------------------
MtKun-2         ------------------------------------------------------------
MtKun-3         ------------------------------------------------------------
MtKun-26        ------------------------------------------------------------
FvKun-3         ------------------------------------------------------------
FvKun-4         ------------------------------------------------------------
FvKun-5         ------------------------------------------------------------
MtKun-27        ------------------------------------------------------------
MtKun-28        ------------------------------------------------------------
BdKun-2         ------------------------------------------------------------
OsKun-1         ------------------------------------------------------------
SbKun-1         ------------------------------------------------------------
ZmKun-1         ------------------------------------------------------------
AtKun-4         ------------------------------------------------------------
MtKun-5         ------------------------------------------------------------
MtKun-8         ------------------------------------------------------------
MtKun-12        ------------------------------------------------------------
MtKun-21        ------------------------------------------------------------
MtKun-10        ------------------------------------------------------------
MtKun-18        ------------------------------------------------------------
MtKun-13        ------------------------------------------------------------
MtKun-23        ------------------------------------------------------------
AtKun-5         ------------------------------------------------------------
MtKun-6         ------------------------------------------------------------
FvKun-1         ------------------------------------------------------------
FvKun-2         ------------------------------------------------------------
VvKun-1         ------------------------------------------------------------
VvKun-4         ------------------------------------------------------------
VvKun-2         ------------------------------------------------------------
VvKun-3         ------------------------------------------------------------


AtKun-2         ------------------------------------------------------------
AtKun-1         ------------------------------------------------------------
MtKun-7         ------------------------------------------------------------
MtKun-11        ------------------------------------------------------------
MtKun-9         ------------------------------------------------------------
MtKun-14        VWDRSCVDVSSTSSFPHVLVIRGRVLKTNEKFVIDDVYPLCDTTAKQVLWDQLSHFDNNS
MtKun-20        ------------------------------------------------------------
MtKun-24        ------------------------------------------------------------
MtKun-15        ------------------------------------------------------------
MtKun-16        ------------------------------------------------------------
MtKun-19        ------------------------------------------------------------
MtKun-17        ------------------------------------------------------------
MtKun-22        ------------------------------------------------------------
```

```
MtKun-25        ------------------------------------------------------------
AtKun-3         ------------------------------------------------------------
BdKun-1         ------------------------------------------------------------
MtKun-1b        ------------------------------------------------------------
MtKun-1a        ------------------------------------------------------------
MtKun-4         ------------------------------------------------------------
MtKun-2         ------------------------------------------------------------
MtKun-3         ------------------------------------------------------------
MtKun-26        ------------------------------------------------------------
FvKun-3         ------------------------------------------------------------
FvKun-4         ------------------------------------------------------------
FvKun-5         ------------------------------------------------------------
MtKun-27        ------------------------------------------------------------
MtKun-28        ------------------------------------------------------------
BdKun-2         ------------------------------------------------------------
OsKun-1         ------------------------------------------------------------
SbKun-1         ------------------------------------------------------------
ZmKun-1         ------------------------------------------------------------
AtKun-4         ------------------------------------------------------------
MtKun-5         ------------------------------------------------------------
MtKun-8         ------------------------------------------------------------
MtKun-12        ------------------------------------------------------------
MtKun-21        ------------------------------------------------------------
MtKun-10        ------------------------------------------------------------
MtKun-18        ------------------------------------------------------------
MtKun-13        ------------------------------------------------------------
MtKun-23        ------------------------------------------------------------
AtKun-5         ------------------------------------------------------------
MtKun-6         ------------------------------------------------------------
FvKun-1         ------------------------------------------------------------
FvKun-2         ------------------------------------------------------------
VvKun-1         ------------------------------------------------------------
VvKun-4         ------------------------------------------------------------
VvKun-2         ------------------------------------------------------------
VvKun-3         ------------------------------------------------------------


AtKun-2         -------------------------------
AtKun-1         -------------------------------
MtKun-7         -------------------------------
MtKun-11        -------------------------------
MtKun-9         -------------------------------
MtKun-14        VANLCLCGDFNSVRSDGIKPQVHDYIEVVKR
MtKun-20        -------------------------------
MtKun-24        -------------------------------
MtKun-15        -------------------------------
MtKun-16        -------------------------------
MtKun-19        -------------------------------
MtKun-17        -------------------------------
MtKun-22        -------------------------------
MtKun-25        -------------------------------
AtKun-3         -------------------------------
BdKun-1         -------------------------------
MtKun-1b        -------------------------------
MtKun-1a        -------------------------------
MtKun-4         -------------------------------
MtKun-2         -------------------------------
MtKun-3         -------------------------------
MtKun-26        -------------------------------
FvKun-3         -------------------------------
FvKun-4         -------------------------------
FvKun-5         -------------------------------
MtKun-27        -------------------------------
MtKun-28        -------------------------------
BdKun-2         -------------------------------
OsKun-1         -------------------------------
SbKun-1         -------------------------------
ZmKun-1         -------------------------------
AtKun-4         -------------------------------
MtKun-5         -------------------------------
MtKun-8         -------------------------------
MtKun-12        -------------------------------
MtKun-21        -------------------------------
MtKun-10        -------------------------------
MtKun-18        -------------------------------
MtKun-13        -------------------------------
MtKun-23        -------------------------------
AtKun-5         -------------------------------
MtKun-6         -------------------------------
FvKun-1         -------------------------------
FvKun-2         -------------------------------
VvKun-1         -------------------------------
VvKun-4         -------------------------------
```

```
VvKun-2         --------------------------------
VvKun-3         --------------------------------
```

C. I4 Serpin.

```
CrSRP-1         ------------------------------------------------------------
VcSRP-1         ------------------------------------------------------------
SbSRP-11        ------------------------------------------------------------
OsSRP-14        ------------------------------------------------------------
SmSRP-1         ------------------------------------------------------------
PpSRP-1         ------------------------------------------------------------
PpSRP-2         ------------------------------------------------------------
PpSRP-3         ------------------------------------------------------------
PpSRP-4         ------------------------------------------------------------
SbSRP-8         ------------------------------------------------------------
SbSRP-4         ------------------------------------------------------------
OsSRP-7         ------------------------------------------------------------
MtSRP-8         ------------------------------------------------------------
SbSRP-7         ------------------------------------------------------------
OsSRP-3         ------------------------------------------------------------
AtSRP-4         ------------------------------------------------------------
AtSRP-7         ------------------------------------------------------------
AtSRP-1         ------------------------------------------------------------
AtSRP-6         ------------------------------------------------------------
FvSRP-12        ------------------------------------------------------------
FvSRP-7         ------------------------------------------------------------
FvSRP-11        ------------------------------------------------------------
FvSRP-2         ------------------------------------------------------------
FvSRP-1         ------------------------------------------------------------
FvSRP-10        ------------------------------------------------------------
FvSRP-9         ------------------------------------------------------------
FvSRP-14        ------------------------------------------------------------
FvSRP-6         ------------------------------------------------------------
FvSRP-4         MTDAFLTLYGVFGQTQSTAFGASSSPAFGQQSSAFGAFSTPVFGGSSNQRSPFGGASGTS
FvSRP-13        ------------------------------------------------------------
FvSRP-5         ------------------------------------------------------------
FvSRP-8         ------------------------------------------------------------
AtSRP-2         ------------------------------------------------------------
AtSRP-3         ------------------------------------------------------------
AtSRP-8         ------------------------------------------------------------
SbSRP-16        ------------------------------------------------------------
OsSRP-6b        ------------------------------------------------------------
OsSRP-5         ------------------------------------------------------------
OsSRP-6a        ------------------------------------------------------------
SbSRP-2         ------------------------------------------------------------
SbSRP-3         ------------------------------------------------------------
AtSRP-5         ------------------------------------------------------------
FvSRP-3         ------------------------------------------------------------
MtSRP-11        ------------------------------------------------------------
MtSRP-9         ------------------------------------------------------------
MtSRP-6         ------------------------------------------------------------
MtSRP-16        ------------------------------------------------------------
MtSRP-3         ------------------------------------------------------------
MtSRP-4         ------------------------------------------------------------
MtSRP-10        ------------------------------------------------------------
MtSRP-12        ------------------------------------------------------------
MtSRP-1         ------------------------------------------------------------
MtSRP-13        ------------------------------------------------------------
MtSRP-14        ------------------------------------------------------------
MtSRP-5         ------------------------------------------------------------
MtSRP-2         ------------------------------------------------------------
MtSRP-7         ------------------------------------------------------------
MtSRP-15        ------------------------------------------------------------
SbSRP-12        ------------------------------------------------------------
SbSRP-1         ------------------------------------------------------------
SbSRP-9         ------------------------------------------------------------
OsSRP-10        ------------------------------------------------------------
OsSRP-8         ------------------------------------------------------------
OsSRP-12        ------------------------------------------------------------
SbSRP-15        ------------------------------------------------------------
OsSRP-9         ------------------------------------------------------------
OsSRP-11        ------------------------------------------------------------
OsSRP-15        ------------------------------------------------------------
SbSRP-10        ------------------------------------------------------------
OsSRP-13        ------------------------------------------------------------
SbSRP-13        ------------------------------------------------------------
SbSRP-14        ------------------------------------------------------------
OsSRP-1         ------------------------------------------------------------
OsSRP-2         ------------------------------------------------------------
SbSRP-5         ------------------------------------------------------------
SbSRP-6         ------------------------------------------------------------
OsSRP-4         ------------------------------------------------------------


CrSRP-1         ------------------------------------------------------------
```

```
VcSRP-1         ------------------------------------------------------------
SbSRP-11        ------------------------------------------------------------
OsSRP-14        ------------------------------------------------------------
SmSRP-1         ------------------------------------------------------------
PpSRP-1         ------------------------------------------------------------
PpSRP-2         ------------------------------------------------------------
PpSRP-3         ------------------------------------------------------------
PpSRP-4         ------------------------------------------------------------
SbSRP-8         ------------------------------------------------------------
SbSRP-4         ------------------------------------------------------------
OsSRP-7         ------------------------------------------------------------
MtSRP-8         ------------------------------------------------------------
SbSRP-7         ------------------------------------------------------------
OsSRP-3         ------------------------------------------------------------
AtSRP-4         ------------------------------------------------------------
AtSRP-7         ------------------------------------------------------------
AtSRP-1         ------------------------------------------------------------
AtSRP-6         ------------------------------------------------------------
FvSRP-12        ------------------------------------------------------------
FvSRP-7         ------------------------------------------------------------
FvSRP-11        ------------------------------------------------------------
FvSRP-2         ------------------------------------------------------------
FvSRP-1         ------------------------------------------------------------
FvSRP-10        ------------------------------------------------------------
FvSRP-9         ------------------------------------------------------------
FvSRP-14        ------------------------------------------------------------
FvSRP-6         ------------------------------------------------------------
FvSRP-4         VLGQKPAFGAFGLSPAQTSLFGSTAQPSQPAFRSNMFGNTSSPFGGSQPAFGTNTTPAFG
FvSRP-13        ------------------------------------------------------------
FvSRP-5         ------------------------------------------------------------
FvSRP-8         ------------------------------------------------------------
AtSRP-2         ------------------------------------------------------------
AtSRP-3         ------------------------------------------------------------
AtSRP-8         ------------------------------------------------------------
SbSRP-16        ------------------------------------------------------------
OsSRP-6b        ------------------------------------------------------------
OsSRP-5         ------------------------------------------------------------
OsSRP-6a        ------------------------------------------------------------
SbSRP-2         ------------------------------------------------------------
SbSRP-3         ------------------------------------------------------------
AtSRP-5         ------------------------------------------------------------
FvSRP-3         ------------------------------------------------------------
MtSRP-11        ------------------------------------------------------------
MtSRP-9         ------------------------------------------------------------
MtSRP-6         ------------------------------------------------------------
MtSRP-16        ------------------------------------------------------------
MtSRP-3         ------------------------------------------------------------
MtSRP-4         ------------------------------------------------------------
MtSRP-10        ------------------------------------------------------------
MtSRP-12        ------------------------------------------------------------
MtSRP-1         ------------------------------------------------------------
MtSRP-13        ------------------------------------------------------------
MtSRP-14        ------------------------------------------------------------
MtSRP-5         ------------------------------------------------------------
MtSRP-2         ------------------------------------------------------------
MtSRP-7         ------------------------------------------------------------
MtSRP-15        ------------------------------------------------------------
SbSRP-12        ------------------------------------------------------------
SbSRP-1         ------------------------------------------------------------
SbSRP-9         ------------------------------------------------------------
OsSRP-10        ------------------------------------------------------------
OsSRP-8         ------------------------------------------------------------
OsSRP-12        ------------------------------------------------------------
SbSRP-15        ------------------------------------------------------------
OsSRP-9         ------------------------------------------------------------
OsSRP-11        ------------------------------------------------------------
OsSRP-15        ------------------------------------------------------------
SbSRP-10        ------------------------------------------------------------
OsSRP-13        ------------------------------------------------------------
SbSRP-13        ------------------------------------------------------------
SbSRP-14        ------------------------------------------------------------
OsSRP-1         ------------------------------------------------------------
OsSRP-2         ------------------------------------------------------------
SbSRP-5         ------------------------------------------------------------
SbSRP-6         ------------------------------------------------------------
OsSRP-4         ------------------------------------------------------------


CrSRP-1         ------------------------------------------------------------
VcSRP-1         ------------------------------------------------------------
SbSRP-11        ------------------------------------------------------------
OsSRP-14        ------------------------------------------------------------
SmSRP-1         ------------------------------------------------------------
```

```
PpSRP-1         ------------------------------------------------------------
PpSRP-2         ------------------------------------------------------------
PpSRP-3         ------------------------------------------------------------
PpSRP-4         ------------------------------------------------------------
SbSRP-8         ------------------------------------------------------------
SbSRP-4         ------------------------------------------------------------
OsSRP-7         ------------------------------------------------------------
MtSRP-8         ------------------------------------------------------------
SbSRP-7         ------------------------------------------------------------
OsSRP-3         ------------------------------------------------------------
AtSRP-4         ------------------------------------------------------------
AtSRP-7         ------------------------------------------------------------
AtSRP-1         ------------------------------------------------------------
AtSRP-6         ------------------------------------------------------------
FvSRP-12        ------------------------------------------------------------
FvSRP-7         ------------------------------------------------------------
FvSRP-11        ------------------------------------------------------------
FvSRP-2         ------------------------------------------------------------
FvSRP-1         ------------------------------------------------------------
FvSRP-10        ------------------------------------------------------------
FvSRP-9         ------------------------------------------------------------
FvSRP-14        ------------------------------------------------------------
FvSRP-6         ------------------------------------------------------------
FvSRP-4         STNTTAFGSTNTPAFGTTTNPAFGGQATPAFGATSASPFGGTGTPAFGAPSTPTFGSTPS
FvSRP-13        ------------------------------------------------------------
FvSRP-5         ------------------------------------------------------------
FvSRP-8         ------------------------------------------------------------
AtSRP-2         ------------------------------------------------------------
AtSRP-3         ------------------------------------------------------------
AtSRP-8         ------------------------------------------------------------
SbSRP-16        ------------------------------------------------------------
OsSRP-6b        ------------------------------------------------------------
OsSRP-5         ------------------------------------------------------------
OsSRP-6a        ------------------------------------------------------------
SbSRP-2         ------------------------------------------------------------
SbSRP-3         ------------------------------------------------------------
AtSRP-5         ------------------------------------------------------------
FvSRP-3         ------------------------------------------------------------
MtSRP-11        ------------------------------------------------------------
MtSRP-9         ------------------------------------------------------------
MtSRP-6         ------------------------------------------------------------
MtSRP-16        ------------------------------------------------------------
MtSRP-3         ------------------------------------------------------------
MtSRP-4         ------------------------------------------------------------
MtSRP-10        ------------------------------------------------------------
MtSRP-12        ------------------------------------------------------------
MtSRP-1         ------------------------------------------------------------
MtSRP-13        ------------------------------------------------------------
MtSRP-14        ------------------------------------------------------------
MtSRP-5         ------------------------------------------------------------
MtSRP-2         ------------------------------------------------------------
MtSRP-7         ------------------------------------------------------------
MtSRP-15        ------------------------------------------------------------
SbSRP-12        ------------------------------------------------------------
SbSRP-1         ------------------------------------------------------------
SbSRP-9         ------------------------------------------------------------
OsSRP-10        ------------------------------------------------------------
OsSRP-8         ------------------------------------------------------------
OsSRP-12        ------------------------------------------------------------
SbSRP-15        ------------------------------------------------------------
OsSRP-9         ------------------------------------------------------------
OsSRP-11        ------------------------------------------------------------
OsSRP-15        ------------------------------------------------------------
SbSRP-10        ------------------------------------------------------------
OsSRP-13        ------------------------------------------------------------
SbSRP-13        ------------------------------------------------------------
SbSRP-14        ------------------------------------------------------------
OsSRP-1         ------------------------------------------------------------
OsSRP-2         ------------------------------------------------------------
SbSRP-5         ------------------------------------------------------------
SbSRP-6         ------------------------------------------------------------
OsSRP-4         ------------------------------------------------------------


CrSRP-1         ------------------------------------------------------------
VcSRP-1         ------------------------------------------------------------
SbSRP-11        ------------------------------------------------------------
OsSRP-14        ------------------------------------------------------------
SmSRP-1         ------------------------------------------------------------
PpSRP-1         ------------------------------------------------------------
PpSRP-2         ------------------------------------------------------------
PpSRP-3         ------------------------------------------------------------
PpSRP-4         ------------------------------------------------------------
```

```
SbSRP-8         ------------------------------------------------------------
SbSRP-4         ------------------------------------------------------------
OsSRP-7         ------------------------------------------------------------
MtSRP-8         ------------------------------------------------------------
SbSRP-7         ------------------------------------------------------------
OsSRP-3         ------------------------------------------------------------
AtSRP-4         ------------------------------------------------------------
AtSRP-7         ------------------------------------------------------------
AtSRP-1         ------------------------------------------------------------
AtSRP-6         ------------------------------------------------------------
FvSRP-12        ------------------------------------------------------------
FvSRP-7         ------------------------------------------------------------
FvSRP-11        ------------------------------------------------------------
FvSRP-2         ------------------------------------------------------------
FvSRP-1         ------------------------------------------------------------
FvSRP-10        ------------------------------------------------------------
FvSRP-9         ------------------------------------------------------------
FvSRP-14        ------------------------------------------------------------
FvSRP-6         ------------------------------------------------------------
FvSRP-4         PAFGSTGSAFGTTSSSLFGTGGAFGATPSTPSFGQSSSAFGTTTSAPAFGQSSTGFGFSS
FvSRP-13        ------------------------------------------------------------
FvSRP-5         ------------------------------------------------------------
FvSRP-8         ------------------------------------------------------------
AtSRP-2         ------------------------------------------------------------
AtSRP-3         ------------------------------------------------------------
AtSRP-8         ------------------------------------------------------------
SbSRP-16        ------------------------------------------------------------
OsSRP-6b        ------------------------------------------------------------
OsSRP-5         ------------------------------------------------------------
OsSRP-6a        ------------------------------------------------------------
SbSRP-2         ------------------------------------------------------------
SbSRP-3         ------------------------------------------------------------
AtSRP-5         ------------------------------------------------------------
FvSRP-3         ------------------------------------------------------------
MtSRP-11        ------------------------------------------------------------
MtSRP-9         ------------------------------------------------------------
MtSRP-6         ------------------------------------------------------------
MtSRP-16        ------------------------------------------------------------
MtSRP-3         ------------------------------------------------------------
MtSRP-4         ------------------------------------------------------------
MtSRP-10        ------------------------------------------------------------
MtSRP-12        ------------------------------------------------------------
MtSRP-1         ------------------------------------------------------------
MtSRP-13        ------------------------------------------------------------
MtSRP-14        ------------------------------------------------------------
MtSRP-5         ------------------------------------------------------------
MtSRP-2         ------------------------------------------------------------
MtSRP-7         ------------------------------------------------------------
MtSRP-15        ------------------------------------------------------------
SbSRP-12        ------------------------------------------------------------
SbSRP-1         ------------------------------------------------------------
SbSRP-9         ------------------------------------------------------------
OsSRP-10        ------------------------------------------------------------
OsSRP-8         ------------------------------------------------------------
OsSRP-12        ------------------------------------------------------------
SbSRP-15        ------------------------------------------------------------
OsSRP-9         ------------------------------------------------------------
OsSRP-11        ------------------------------------------------------------
OsSRP-15        ------------------------------------------------------------
SbSRP-10        ------------------------------------------------------------
OsSRP-13        ------------------------------------------------------------
SbSRP-13        ------------------------------------------------------------
SbSRP-14        ------------------------------------------------------------
OsSRP-1         ------------------------------------------------------------
OsSRP-2         ------------------------------------------------------------
SbSRP-5         ------------------------------------------------------------
SbSRP-6         ------------------------------------------------------------
OsSRP-4         ------------------------------------------------------------


CrSRP-1         ------------------------------------------------------------
VcSRP-1         ------------------------------------------------------------
SbSRP-11        ------------------------------------------------------------
OsSRP-14        ------------------------------------------------------------
SmSRP-1         ------------------------------------------------------------
PpSRP-1         ------------------------------------------------------------
PpSRP-2         ------------------------------------------------------------
PpSRP-3         ------------------------------------------------------------
PpSRP-4         ------------------------------------------------------------
SbSRP-8         ------------------------------------------------------------
SbSRP-4         ------------------------------------------------------------
OsSRP-7         ------------------------------------------------------------
MtSRP-8         ------------------------------------------------------------
```

```
SbSRP-7         ------------------------------------------------------------
OsSRP-3         ------------------------------------------------------------
AtSRP-4         ------------------------------------------------------------
AtSRP-7         ------------------------------------------------------------
AtSRP-1         ------------------------------------------------------------
AtSRP-6         ------------------------------------------------------------
FvSRP-12        ------------------------------------------------------------
FvSRP-7         ------------------------------------------------------------
FvSRP-11        ------------------------------------------------------------
FvSRP-2         ------------------------------------------------------------
FvSRP-1         ------------------------------------------------------------
FvSRP-10        ------------------------------------------------------------
FvSRP-9         ------------------------------------------------------------
FvSRP-14        ------------------------------------------------------------
FvSRP-6         ------------------------------------------------------------
FvSRP-4         STPAFGQSSSTFGSTQFGASSPFGGQSSSFGVQSTTPTLGNNAFGQSTFGAQQGGSRATP
FvSRP-13        ------------------------------------------------------------
FvSRP-5         ------------------------------------------------------------
FvSRP-8         ------------------------------------------------------------
AtSRP-2         ------------------------------------------------------------
AtSRP-3         ------------------------------------------------------------
AtSRP-8         ------------------------------------------------------------
SbSRP-16        ------------------------------------------------------------
OsSRP-6b        ------------------------------------------------------------
OsSRP-5         ------------------------------------------------------------
OsSRP-6a        ------------------------------------------------------------
SbSRP-2         ------------------------------------------------------------
SbSRP-3         ------------------------------------------------------------
AtSRP-5         ------------------------------------------------------------
FvSRP-3         ------------------------------------------------------------
MtSRP-11        ------------------------------------------------------------
MtSRP-9         ------------------------------------------------------------
MtSRP-6         ------------------------------------------------------------
MtSRP-16        ------------------------------------------------------------
MtSRP-3         ------------------------------------------------------------
MtSRP-4         ------------------------------------------------------------
MtSRP-10        ------------------------------------------------------------
MtSRP-12        ------------------------------------------------------------
MtSRP-1         ------------------------------------------------------------
MtSRP-13        ------------------------------------------------------------
MtSRP-14        ------------------------------------------------------------
MtSRP-5         ------------------------------------------------------------
MtSRP-2         ------------------------------------------------------------
MtSRP-7         ------------------------------------------------------------
MtSRP-15        ------------------------------------------------------------
SbSRP-12        ------------------------------------------------------------
SbSRP-1         ------------------------------------------------------------
SbSRP-9         ------------------------------------------------------------
OsSRP-10        ------------------------------------------------------------
OsSRP-8         ------------------------------------------------------------
OsSRP-12        ------------------------------------------------------------
SbSRP-15        ------------------------------------------------------------
OsSRP-9         ------------------------------------------------------------
OsSRP-11        ------------------------------------------------------------
OsSRP-15        ------------------------------------------------------------
SbSRP-10        ------------------------------------------------------------
OsSRP-13        ------------------------------------------------------------
SbSRP-13        ------------------------------------------------------------
SbSRP-14        ------------------------------------------------------------
OsSRP-1         ------------------------------------------------------------
OsSRP-2         ------------------------------------------------------------
SbSRP-5         ------------------------------------------------------------
SbSRP-6         ------------------------------------------------------------
OsSRP-4         ------------------------------------------------------------


CrSRP-1         ------------------------------------------------------------
VcSRP-1         ------------------------------------------------------------
SbSRP-11        ------------------------------------------------------------
OsSRP-14        ------------------------------------------------------------
SmSRP-1         ------------------------------------------------------------
PpSRP-1         ------------------------------------------------------------
PpSRP-2         ------------------------------------------------------------
PpSRP-3         ------------------------------------------------------------
PpSRP-4         ------------------------------------------------------------
SbSRP-8         ------------------------------------------------------------
SbSRP-4         ------------------------------------------------------------
OsSRP-7         ------------------------------------------------------------
MtSRP-8         ------------------------------------------------------------
SbSRP-7         ------------------------------------------------------------
OsSRP-3         ------------------------------------------------------------
AtSRP-4         ------------------------------------------------------------
AtSRP-7         ------------------------------------------------------------
```

```
AtSRP-1         ------------------------------------------------------------
AtSRP-6         ------------------------------------------------------------
FvSRP-12        ------------------------------------------------------------
FvSRP-7         ------------------------------------------------------------
FvSRP-11        ------------------------------------------------------------
FvSRP-2         ------------------------------------------------------------
FvSRP-1         ------------------------------------------------------------
FvSRP-10        ------------------------------------------------------------
FvSRP-9         ------------------------------------------------------------
FvSRP-14        ------------------------------------------------------------
FvSRP-6         ------------------------------------------------------------
FvSRP-4         YTGTSEPDSNGLGKLESISAMPAYKEKSHEELRWEDYQLGDKAGPSPAGGGGFGMSTVQT
FvSRP-13        ------------------------------------------------------------
FvSRP-5         ------------------------------------------------------------
FvSRP-8         ------------------------------------------------------------
AtSRP-2         ------------------------------------------------------------
AtSRP-3         ------------------------------------------------------------
AtSRP-8         ------------------------------------------------------------
SbSRP-16        ------------------------------------------------------------
OsSRP-6b        ------------------------------------------------------------
OsSRP-5         ------------------------------------------------------------
OsSRP-6a        ------------------------------------------------------------
SbSRP-2         ------------------------------------------------------------
SbSRP-3         ------------------------------------------------------------
AtSRP-5         ------------------------------------------------------------
FvSRP-3         ------------------------------------------------------------
MtSRP-11        ------------------------------------------------------------
MtSRP-9         ------------------------------------------------------------
MtSRP-6         ------------------------------------------------------------
MtSRP-16        ------------------------------------------------------------
MtSRP-3         ------------------------------------------------------------
MtSRP-4         ------------------------------------------------------------
MtSRP-10        ------------------------------------------------------------
MtSRP-12        ------------------------------------------------------------
MtSRP-1         ------------------------------------------------------------
MtSRP-13        ------------------------------------------------------------
MtSRP-14        ------------------------------------------------------------
MtSRP-5         ------------------------------------------------------------
MtSRP-2         ------------------------------------------------------------
MtSRP-7         ------------------------------------------------------------
MtSRP-15        ------------------------------------------------------------
SbSRP-12        ------------------------------------------------------------
SbSRP-1         ------------------------------------------------------------
SbSRP-9         ------------------------------------------------------------
OsSRP-10        ------------------------------------------------------------
OsSRP-8         ------------------------------------------------------------
OsSRP-12        ------------------------------------------------------------
SbSRP-15        ------------------------------------------------------------
OsSRP-9         ------------------------------------------------------------
OsSRP-11        ------------------------------------------------------------
OsSRP-15        ------------------------------------------------------------
SbSRP-10        ------------------------------------------------------------
OsSRP-13        ------------------------------------------------------------
SbSRP-13        ------------------------------------------------------------
SbSRP-14        ------------------------------------------------------------
OsSRP-1         ------------------------------------------------------------
OsSRP-2         ------------------------------------------------------------
SbSRP-5         ------------------------------------------------------------
SbSRP-6         ------------------------------------------------------------
OsSRP-4         ------------------------------------------------------------


CrSRP-1         ------------------------------------------------------------
VcSRP-1         ------------------------------------------------------------
SbSRP-11        ------------------------------------------------------------
OsSRP-14        ------------------------------------------------------------
SmSRP-1         ------------------------------------------------------------
PpSRP-1         ------------------------------------------------------------
PpSRP-2         ------------------------------------------------------------
PpSRP-3         ------------------------------------------------------------
PpSRP-4         ------------------------------------------------------------
SbSRP-8         ------------------------------------------------------------
SbSRP-4         ------------------------------------------------------------
OsSRP-7         ------------------------------------------------------------
MtSRP-8         ------------------------------------------------------------
SbSRP-7         ------------------------------------------------------------
OsSRP-3         ------------------------------------------------------------
AtSRP-4         ------------------------------------------------------------
AtSRP-7         ------------------------------------------------------------
AtSRP-1         ------------------------------------------------------------
AtSRP-6         ------------------------------------------------------------
FvSRP-12        ------------------------------------------------------------
FvSRP-7         ------------------------------------------------------------
```

```
FvSRP-11        ------------------------------------------------------------
FvSRP-2         ------------------------------------------------------------
FvSRP-1         ------------------------------------------------------------
FvSRP-10        ------------------------------------------------------------
FvSRP-9         ------------------------------------------------------------
FvSRP-14        ------------------------------------------------------------
FvSRP-6         ------------------------------------------------------------
FvSRP-4         NPLNPIHATPFAQTCSSPFNHSTSSSLFAPKTSSFPSAGFGVTSSSPFSSSSPFSSSSPF
FvSRP-13        ------------------------------------------------------------
FvSRP-5         ------------------------------------------------------------
FvSRP-8         ------------------------------------------------------------
AtSRP-2         ------------------------------------------------------------
AtSRP-3         ------------------------------------------------------------
AtSRP-8         ------------------------------------------------------------
SbSRP-16        ------------------------------------------------------------
OsSRP-6b        ------------------------------------------------------------
OsSRP-5         ------------------------------------------------------------
OsSRP-6a        ------------------------------------------------------------
SbSRP-2         ------------------------------------------------------------
SbSRP-3         ------------------------------------------------------------
AtSRP-5         ------------------------------------------------------------
FvSRP-3         ------------------------------------------------------------
MtSRP-11        ------------------------------------------------------------
MtSRP-9         ------------------------------------------------------------
MtSRP-6         ------------------------------------------------------------
MtSRP-16        ------------------------------------------------------------
MtSRP-3         ------------------------------------------------------------
MtSRP-4         ------------------------------------------------------------
MtSRP-10        ------------------------------------------------------------
MtSRP-12        ------------------------------------------------------------
MtSRP-1         ------------------------------------------------------------
MtSRP-13        ------------------------------------------------------------
MtSRP-14        ------------------------------------------------------------
MtSRP-5         ------------------------------------------------------------
MtSRP-2         ------------------------------------------------------------
MtSRP-7         ------------------------------------------------------------
MtSRP-15        ------------------------------------------------------------
SbSRP-12        ------------------------------------------------------------
SbSRP-1         ------------------------------------------------------------
SbSRP-9         ------------------------------------------------------------
OsSRP-10        ------------------------------------------------------------
OsSRP-8         ------------------------------------------------------------
OsSRP-12        ------------------------------------------------------------
SbSRP-15        ------------------------------------------------------------
OsSRP-9         ------------------------------------------------------------
OsSRP-11        ------------------------------------------------------------
OsSRP-15        ------------------------------------------------------------
SbSRP-10        ------------------------------------------------------------
OsSRP-13        ------------------------------------------------------------
SbSRP-13        ------------------------------------------------------------
SbSRP-14        ------------------------------------------------------------
OsSRP-1         ------------------------------------------------------------
OsSRP-2         ------------------------------------------------------------
SbSRP-5         ------------------------------------------------------------
SbSRP-6         ------------------------------------------------------------
OsSRP-4         ------------------------------------------------------------


CrSRP-1         ------------------------------------------------------------
VcSRP-1         ------------------------------------------------------------
SbSRP-11        ------------------------------------------------------------
OsSRP-14        ------------------------------------------------------------
SmSRP-1         ------------------------------------------------------------
PpSRP-1         ------------------------------------------------------------
PpSRP-2         ------------------------------------------------------------
PpSRP-3         ------------------------------------------------------------
PpSRP-4         ------------------------------------------------------------
SbSRP-8         ------------------------------------------------------------
SbSRP-4         ------------------------------------------------------------
OsSRP-7         ------------------------------------------------------------
MtSRP-8         ------------------------------------------------------------
SbSRP-7         ------------------------------------------------------------
OsSRP-3         ------------------------------------------------------------
AtSRP-4         ------------------------------------------------------------
AtSRP-7         ------------------------------------------------------------
AtSRP-1         ------------------------------------------------------------
AtSRP-6         ------------------------------------------------------------
FvSRP-12        ------------------------------------------------------------
FvSRP-7         ------------------------------------------------------------
FvSRP-11        ------------------------------------------------------------
FvSRP-2         ------------------------------------------------------------
FvSRP-1         ------------------------------------------------------------
FvSRP-10        ------------------------------------------------------------
```

```
FvSRP-9         ------------------------------------------------------------
FvSRP-14        ------------------------------------------------------------
FvSRP-6         ------------------------------------------------------------
FvSRP-4         SSSWPFSSSTQGNLFTPASSAPAFGSAPAFGQTSSPLPFSSPSSFSFSTAATSSTPSFNF
FvSRP-13        ------------------------------------------------------------
FvSRP-5         ------------------------------------------------------------
FvSRP-8         ------------------------------------------------------------
AtSRP-2         ------------------------------------------------------------
AtSRP-3         ------------------------------------------------------------
AtSRP-8         ------------------------------------------------------------
SbSRP-16        ------------------------------------------------------------
OsSRP-6b        ------------------------------------------------------------
OsSRP-5         ------------------------------------------------------------
OsSRP-6a        ------------------------------------------------------------
SbSRP-2         ------------------------------------------------------------
SbSRP-3         ------------------------------------------------------------
AtSRP-5         ------------------------------------------------------------
FvSRP-3         ------------------------------------------------------------
MtSRP-11        ------------------------------------------------------------
MtSRP-9         ------------------------------------------------------------
MtSRP-6         ------------------------------------------------------------
MtSRP-16        ------------------------------------------------------------
MtSRP-3         ------------------------------------------------------------
MtSRP-4         ------------------------------------------------------------
MtSRP-10        ------------------------------------------------------------
MtSRP-12        ------------------------------------------------------------
MtSRP-1         ------------------------------------------------------------
MtSRP-13        ------------------------------------------------------------
MtSRP-14        ------------------------------------------------------------
MtSRP-5         ------------------------------------------------------------
MtSRP-2         ------------------------------------------------------------
MtSRP-7         ------------------------------------------------------------
MtSRP-15        ------------------------------------------------------------
SbSRP-12        ------------------------------------------------------------
SbSRP-1         ------------------------------------------------------------
SbSRP-9         ------------------------------------------------------------
OsSRP-10        ------------------------------------------------------------
OsSRP-8         ------------------------------------------------------------
OsSRP-12        ------------------------------------------------------------
SbSRP-15        ------------------------------------------------------------
OsSRP-9         ------------------------------------------------------------
OsSRP-11        ------------------------------------------------------------
OsSRP-15        ------------------------------------------------------------
SbSRP-10        ------------------------------------------------------------
OsSRP-13        ------------------------------------------------------------
SbSRP-13        ------------------------------------------------------------
SbSRP-14        ------------------------------------------------------------
OsSRP-1         ------------------------------------------------------------
OsSRP-2         ------------------------------------------------------------
SbSRP-5         ------------------------------------------------------------
SbSRP-6         ------------------------------------------------------------
OsSRP-4         ------------------------------------------------------------


CrSRP-1         ------------------------------------------------------------
VcSRP-1         ------------------------------------------------------------
SbSRP-11        ------------------------------------------------------------
OsSRP-14        ------------------------------------------------------------
SmSRP-1         ------------------------------------------------------------
PpSRP-1         ------------------------------------------------------------
PpSRP-2         ------------------------------------------------------------
PpSRP-3         ------------------------------------------------------------
PpSRP-4         ------------------------------------------------------------
SbSRP-8         ------------------------------------------------------------
SbSRP-4         ------------------------------------------------------------
OsSRP-7         ------------------------------------------------------------
MtSRP-8         ------------------------------------------------------------
SbSRP-7         ------------------------------------------------------------
OsSRP-3         ------------------------------------------------------------
AtSRP-4         ------------------------------------------------------------
AtSRP-7         ------------------------------------------------------------
AtSRP-1         ------------------------------------------------------------
AtSRP-6         ------------------------------------------------------------
FvSRP-12        ------------------------------------------------------------
FvSRP-7         ------------------------------------------------------------
FvSRP-11        ------------------------------------------------------------
FvSRP-2         ------------------------------------------------------------
FvSRP-1         ------------------------------------------------------------
FvSRP-10        ------------------------------------------------------------
FvSRP-9         ------------------------------------------------------------
FvSRP-14        ------------------------------------------------------------
FvSRP-6         ------------------------------------------------------------
FvSRP-4         PTPSNPQTQSAQTGTISFQLNPSTFRSNLFNNTSSLQSSSLGTTSNQLGNNPPAETDSST
```

```
FvSRP-13        ------------------------------------------------------------
FvSRP-5         ------------------------------------------------------------
FvSRP-8         ------------------------------------------------------------
AtSRP-2         ------------------------------------------------------------
AtSRP-3         ------------------------------------------------------------
AtSRP-8         ------------------------------------------------------------
SbSRP-16        ------------------------------------------------------------
OsSRP-6b        ------------------------------------------------------------
OsSRP-5         ------------------------------------------------------------
OsSRP-6a        ------------------------------------------------------------
SbSRP-2         ------------------------------------------------------------
SbSRP-3         ------------------------------------------------------------
AtSRP-5         ------------------------------------------------------------
FvSRP-3         ------------------------------------------------------------
MtSRP-11        ------------------------------------------------------------
MtSRP-9         ------------------------------------------------------------
MtSRP-6         ------------------------------------------------------------
MtSRP-16        ------------------------------------------------------------
MtSRP-3         ------------------------------------------------------------
MtSRP-4         ------------------------------------------------------------
MtSRP-10        ------------------------------------------------------------
MtSRP-12        ------------------------------------------------------------
MtSRP-1         ------------------------------------------------------------
MtSRP-13        ------------------------------------------------------------
MtSRP-14        ------------------------------------------------------------
MtSRP-5         ------------------------------------------------------------
MtSRP-2         ------------------------------------------------------------
MtSRP-7         ------------------------------------------------------------
MtSRP-15        ------------------------------------------------------------
SbSRP-12        ------------------------------------------------------------
SbSRP-1         ------------------------------------------------------------
SbSRP-9         ------------------------------------------------------------
OsSRP-10        ------------------------------------------------------------
OsSRP-8         ------------------------------------------------------------
OsSRP-12        ------------------------------------------------------------
SbSRP-15        ------------------------------------------------------------
OsSRP-9         ------------------------------------------------------------
OsSRP-11        ------------------------------------------------------------
OsSRP-15        ------------------------------------------------------------
SbSRP-10        ------------------------------------------------------------
OsSRP-13        ------------------------------------------------------------
SbSRP-13        ------------------------------------------------------------
SbSRP-14        ------------------------------------------------------------
OsSRP-1         ------------------------------------------------------------
OsSRP-2         ------------------------------------------------------------
SbSRP-5         ------------------------------------------------------------
SbSRP-6         ------------------------------------------------------------
OsSRP-4         ------------------------------------------------------------


CrSRP-1         ------------------------------------------------------------
VcSRP-1         ---------------------------------------------------------MRL
SbSRP-11        ------------------------------------------------------------
OsSRP-14        ------------------------------------------------------------
SmSRP-1         ------------------------------------------------------------
PpSRP-1         ------------------------------------------------------------
PpSRP-2         -------------------------------------MNNLSGLGVVALCGAVCAFVVTL
PpSRP-3         ------------------------------------------------------------
PpSRP-4         ------------------------------------------------------------
SbSRP-8         ------------------------------------------------------------
SbSRP-4         ------------------------------------------------------------
OsSRP-7         ------------------------------------------------------------
MtSRP-8         ------------------------------------------------------------
SbSRP-7         ------------------------------------------------------------
OsSRP-3         ------------------------------------------------------------
AtSRP-4         ------------------------------------------------------------
AtSRP-7         ------------------------------------------------------------
AtSRP-1         ------------------------------------------------------------
AtSRP-6         ----------------------------------------------------MEPKEKKQ
FvSRP-12        ------------------------------------------------------------
FvSRP-7         ------------------------------------------------------------
FvSRP-11        ------------------------------------------------------------
FvSRP-2         ------------------------------------------------------------
FvSRP-1         ------------------------------------------------------------
FvSRP-10        ------------------------------------------------------------
FvSRP-9         ------------------------------------------------------------
FvSRP-14        ------------------------------------------------------------
FvSRP-6         -----------MDNKDDGRRISYTPEQCTPYNPSSPVYTPSSSAYTPSSPGYTPTTPGSP
FvSRP-4         TITINEPSLSNGGSSQPGFGYTSSSTLGTITINSSSGSRITITPASSTTITITPTSGSTT
FvSRP-13        ------------------------------------------------------------
FvSRP-5         ------------------------------------------------------------
FvSRP-8         ------------------------------------------------------------
AtSRP-2         ------------------------------------------------------------
```

```
AtSRP-3         ------------------------------------------------------------
AtSRP-8         ------------------------------------------------------------
SbSRP-16        ------------------------------------------------------------
OsSRP-6b        ------------------------------------------------------------
OsSRP-5         ------------------------------------------------------------
OsSRP-6a        ------------------------------------------------------------
SbSRP-2         ------------------------------------------------------------
SbSRP-3         ---------------------------------------MGPALTPVAFMEAEKAFFAWS
AtSRP-5         ------------------------------------------------------------
FvSRP-3         ------------------------------------------------------------
MtSRP-11        ------------------------------------------------------------
MtSRP-9         ------------------------------------------------------------
MtSRP-6         ------------------------------------------------------------
MtSRP-16        ------------------------------------------------------------
MtSRP-3         ------------------------------------------------------------
MtSRP-4         ------------------------------------------------------------
MtSRP-10        ------------------------------------------------------------
MtSRP-12        ------------------------------------------------------------
MtSRP-1         ------------------------------------------------------------
MtSRP-13        ------------------------------------------------------------
MtSRP-14        ------------------------------------------------------------
MtSRP-5         ------------------------------------------------------------
MtSRP-2         ------------------------------------------------------------
MtSRP-7         ------------------------------------------------------------
MtSRP-15        ------------------------------------------------------------
SbSRP-12        ------------------------------------------------------------
SbSRP-1         ------------------------------------------------------------
SbSRP-9         ------------------------------------------------------------
OsSRP-10        ------------------------------------------------------------
OsSRP-8         -----------------------------------MGSPPPPRRRCCAIQGFVVLFLVYV
OsSRP-12        --------------------------------MATPPAAGAPRRRWCAIQGLVVLFLVYV
SbSRP-15        ---------------------------------------------------------MAR
OsSRP-9         ------------------------------------------------------------
OsSRP-11        ------------------------------------------------------------
OsSRP-15        ------------------------------------------------------------
SbSRP-10        ------------------------------------------------------------
OsSRP-13        ------------------------------------------------------------
SbSRP-13        ------------------------------------------------------------
SbSRP-14        ------------------------------------------------------------
OsSRP-1         ------------------------------------------------------------
OsSRP-2         ------------------------------------------------------------
SbSRP-5         ------------------------------MSSCHPTGSRNTSSSWRQDDHAAPTHDGTD
SbSRP-6         -----------MSTPRRGGQHHGDERAGTRAGAASRDNGGQGAGDLSFGSTAGPWIAFDP
OsSRP-4         ------------------------------------------------------------


CrSRP-1         ------------------------------------------------------MPPVK-
VcSRP-1         FTHKPKVTVTVKQRVPTTCYPTAVG-----------------------STIFH--RQLR-
SbSRP-11        -------------MQAASLRRALHPRRPGAAQLFPFPVGTRPGAFSASAAAAHSPKDAR-
OsSRP-14        ----------------------MQV-----------------------SSYLR--RALR-
SmSRP-1         ------------------------------------------------------------
PpSRP-1         -------------------------------------------------------MDVG-
PpSRP-2         QMGYPNEGFSVRKKILSWFRLGEKP-----------------------STLSK--MDVA-
PpSRP-3         ---------------------------------------------------MG--LDVE-
PpSRP-4         ---------------------------------------------------MG--LDVA-
SbSRP-8         ------------------------------------------------MTGQA--VAAA-
SbSRP-4         ------------------------------------------------------------
OsSRP-7         ------------------------------------------------------------
MtSRP-8         ----------------MKRKYDESS-----------------------SFSSS--YFFS-
SbSRP-7         -------------------------------------------------------MQLA-
OsSRP-3         -------------------------------------------------------MELA-
AtSRP-4         -------------------------------------------------------MDLE-
AtSRP-7         -------------------------------------------------------MDVR-
AtSRP-1         --MDSKRKNQELSTSETADPSLSKT-----------------------NKKQK--IDMQ-
AtSRP-6         KLDTSEVASPSLSKTHLKKKKTKKQKIRKSQEITSPSLSKNTDLVIASPSLSN--IDVG-
FvSRP-12        ------------------------------------------------------------
FvSRP-7         ------------------------------------------------------------
FvSRP-11        -------------------------------------------------------MDTK-
FvSRP-2         ------------------------------------------------------------
FvSRP-1         --------------------MDDLK-----------------------ALQDL--EALQ-
FvSRP-10        --------------------MD---------------------------YKYL--EAFQ-
FvSRP-9         -------------------------------------------------MAYF--KHLK-
FvSRP-14        -------------------------------------------------MADL--QHLK-
FvSRP-6         EYNPSFPQYTPYSPYSTVYTPSSSAYSPTSPGSPEYHPSSPQYTPYPPPYTPY--STAYI
FvSRP-4         VTIAPSSGTTSTSTSGHTPFASPFVVPSQASQPSQTSTSIFYTINSFDQIQPGNRDGFGS
FvSRP-13        ------------------MAPRPIP-----------------------SFKAS--ESLR-
FvSRP-5         --------------MAARFPYQPRP-----------------------SFKAS--ESLR-
FvSRP-8         ---------MENKRLKNCETQKLKAIRETNSRKHGCTIRSIPIPILISTTTFI--QSIR-
AtSRP-2         -------------------------------------------------------MELG-
AtSRP-3         -------------------------------------------------------MELG-
AtSRP-8         -------------------------------------------------------MELG-
SbSRP-16        -------------------------------------------------------MAGT-
OsSRP-6b        ------------------------------------------------------------
```

```
OsSRP-5         --------------------------------------------------MDATATDLR-
OsSRP-6a        -----------------------------------------------------MAADLR-
SbSRP-2         ----------------------------------------------------MATADIR-
SbSRP-3         LLLSSPAKYPENTLQDLEIATPPGQRSAPRFFLVESQGSLSDQVDLPTPTMAT--ADIR-
AtSRP-5         -------------------------------------------------------MDVR-
FvSRP-3         -------------------------------------------------------MDLS-
MtSRP-11        ----MGIDNNILTLDSLCCLLKSDS-----------------------IRRIM--NILS-
MtSRP-9         ----------------------------------------------------M--SYHR-
MtSRP-6         ----------------------------------------------------MMQNLIR-
MtSRP-16        --------------MLPQKRRTSEQ------------------------------NLIG-
MtSRP-3         ------------------------------------------------------------
MtSRP-4         -------------------------------------------------------MNLQ-
MtSRP-10        -------------------MADRES-----------------------KSKQK--RMIL-
MtSRP-12        --------------MAHCESKPEEK------------------------------RMIL-
MtSRP-1         ------------------------------------------------------------
MtSRP-13        ------------------------------------------------------------
MtSRP-14        -------------------------------------------------------MALH-
MtSRP-5         ----------------------------MRFSRKTIAIAKVVLCFFVLDFLLRSESALA-
MtSRP-2         -------------------------------------------------MIPNTQSDIQ-
MtSRP-7         -------------------------------------------------------MDLR-
MtSRP-15        -------------------------------------------------------MDHR-
SbSRP-12        -----------------------MA-----------------------AADAA--IAAR-
SbSRP-1         -------------MQFPTTITSPWS-----------------------MEADT--QRVS-
SbSRP-9         ----------------MTRKRRRSG-----------------------KTVPK--KSNA-
OsSRP-10        --------------------------------------------------MES--CARR-
OsSRP-8         LAVLVLAGGELFRDDHPLDLRFPSS-----------------------PGIGS--SSSSS
OsSRP-12        LAVLVLAGGELFHDDQLQPRFPSSPGIGSSSSSSSARILLSPRSMIRRLGEIARRSGSR-
SbSRP-15        EAGWSRAPRPATRTLMEQRRHGQPP-----------------------VSSCR--PHRR-
OsSRP-9         ----------------------------------------------------M--EDNA-
OsSRP-11        ----------------------------------------------------M--EDDA-
OsSRP-15        -------------MDDGEAARRHRH------------------------------RAIS-
SbSRP-10        -----------------------MA-----------------------SGKGE--KATS-
OsSRP-13        ------------------------------------------------------------
SbSRP-13        ------------------------------------------------------------
SbSRP-14        ------------------------------------------------------------
OsSRP-1         ------------------------------------------------------------
OsSRP-2         ------------------------------------------------------------
SbSRP-5         DGHSPQPFPAPSVTAAIARYLASQP-----------------------TQHIS--QQPSP
SbSRP-6         PHPWAPPEHARAPVFAQQAPVDPFP-----------------------WSTPT--SFANP
OsSRP-4         -------MAALAAGEPFSGRATGGD------------------------------GGVR-


CrSRP-1         ------------------------------------------------------------
VcSRP-1         ---------------------------------------------------TTM------
SbSRP-11        -------------------------------------NAPPPIMPTRPWGEALA------
OsSRP-14        ----------RPPFPAGDANHRRLSSAPAPKPEAPAEAMPPPPMPTRPWGEALA------
SmSRP-1         ----------------------------------------------------MI------
PpSRP-1         ---------------------------------------------------ALA------
PpSRP-2         ---------------------------------------------------ASV------
PpSRP-3         ---------------------------------------------------AMV------
PpSRP-4         ---------------------------------------------------SMV------
SbSRP-8         ---------------------------------------------------AAV------
SbSRP-4         ------------------------------------------------------------
OsSRP-7         ------------------------------------------------------------
MtSRP-8         ---------------------------------------------------SAV------
SbSRP-7         ---------------------------------------------------EAA------
OsSRP-3         ---------------------------------------------------EAV------
AtSRP-4         ---------------------------------------------------ESI------
AtSRP-7         ---------------------------------------------------EAM------
AtSRP-1         ---------------------------------------------------EAM------
AtSRP-6         ---------------------------------------------------EAM------
FvSRP-12        ------------------------------------------------------------
FvSRP-7         ------------------------------------------------------------
FvSRP-11        ---------------------------------------------------DAI------
FvSRP-2         ------------------------------------------------------------
FvSRP-1         ---------------------------------------------------TPI------
FvSRP-10        ---------------------------------------------------TPI------
FvSRP-9         ---------------------------------------------------EPI------
FvSRP-14        ---------------------------------------------------EPI------
FvSRP-6         PQVYHPTQQNGVPSYIPSPSLYSPSRYTAAGHTIMNSTHPPPSFKPSKELRESI------
FvSRP-4         SFERLQNNSGRWLGSACPATNPFNLLHRPGQLPSGHAAPSPSVQNGISGSLTAMRDLIES
FvSRP-13        ---------------------------------------------------KSI------
FvSRP-5         ---------------------------------------------------KSI------
FvSRP-8         --------------------------------------------VSKKIHSKSI------
AtSRP-2         ---------------------------------------------------KSI------
AtSRP-3         ---------------------------------------------------KSI------
AtSRP-8         ---------------------------------------------------KSM------
SbSRP-16        --------------------------------------------------LLSI------
OsSRP-6b        ------------------------------------------------------------
OsSRP-5         ---------------------------------------------------VSI------
OsSRP-6a        ---------------------------------------------------VSI------
SbSRP-2         ---------------------------------------------------RSI------
SbSRP-3         ---------------------------------------------------VSI------
```

```
AtSRP-5         ---------------------------------------------------ESI------
FvSRP-3         ---------------------------------------------------ASI------
MtSRP-11        ---------------------------------------------------KSI------
MtSRP-9         ---------------------------------------------------ESI------
MtSRP-6         ---------------------------------------------------KSL------
MtSRP-16        ---------------------------------------------------KSL------
MtSRP-3         ----------------------------------------------------ML------
MtSRP-4         --------------------------------------------------SESI------
MtSRP-10        ---------------------------------------------------ETI------
MtSRP-12        ---------------------------------------------------ESI------
MtSRP-1         ------------------------------------------------------------
MtSRP-13        ------------------------------------------------------------
MtSRP-14        ---------------------------------------------------ESI------
MtSRP-5         ------------------------------------------------------------
MtSRP-2         ------------------------------IDPTQHQQLLDFLRSESALAN---------
MtSRP-7         ---------------------------------------------------ESI------
MtSRP-15        ---------------------------------------------------ESI------
SbSRP-12        ------------------------------------------------------------
SbSRP-1         ---------------------------------------------------AAS------
SbSRP-9         ------------------------------------------------------------
OsSRP-10        ---------------------------------------------------CAV------
OsSRP-8         ARFLLSPRSLLLRLGEIASRRGRWWRPESDSPTSGGRKDGNSSTTEACSRRCAA------
OsSRP-12        --------------------RWWTGGVRPESGSPRSEGGNSSATDQACSRRCAA------
SbSRP-15        ------------------------------------GGGGGQARPSKRARGTAA------
OsSRP-9         ---------------------------------------------------GDC------
OsSRP-11        ---------------------------------------------------GNC------
OsSRP-15        ------------------------------------------------------------
SbSRP-10        ---------------------------------------------------VCQ------
OsSRP-13        ------------------------------------------------------------
SbSRP-13        ------------------------------------------------------------
SbSRP-14        ------------------------------------------------------------
OsSRP-1         ------------------------------------------------------------
OsSRP-2         ------------------------------------------------------------
SbSRP-5         PRWHKHNYTPLQQWPPHRAAPAGPWQWASSTFAPPNVTVVVPAGETPAPGGACVAG----
SbSRP-6         FHGPSTLNVPWGPPGEVPFAGFPAAGPAAPEPEWRHRRQDVFAGIGRRSVPAVVTDPDLD
OsSRP-4         ---------------------------------------------------SDVMAPPAM


CrSRP-1         -APVADAPASPALSALSKASYALFLEAAAGTGGK------GCFLSPMSIIYALTLALN--
VcSRP-1         -ASCKSMPISPAASAVSKASFSLVLGALTGSTSPQ-----GCFLSPLSIVYALSLALN--
SbSRP-11        -AAQRAFCLPLAGRVLAA------------SATG------NAAVAPVAVHASLALAAA--
OsSRP-14        -AAQRAFCLPLAGRVLAA------------AGTG------NAAVSAPAVHVSLALAAG--
SmSRP-1         -HDQTSFTLDLYKQVWQQQQQESKPQGSSSTDREEQHNEKNVVLSPLSIAMAMAMAAA--
PpSRP-1         -RQQTEFVLDLYRKIANR------------APEE------NTVLSPISISLALSMVAA--
PpSRP-2         -AGQTQFTVDLYKTLVKG------------KESE------NVVLSPLSVDLALAMLTA--
PpSRP-3         -HGQTEFTIDLYKAVVKG------------KETE------NAVLSPVCISLALAMVSA--
PpSRP-4         -HGQMEFTIDLHKAVVKG------------KETK------NVLLSPLSISLALAMVSA--
SbSRP-8         -RDQAALCLLLLRHLGGL---------DNGMPTG------NVAFSPISFHSTLSLLAA--
SbSRP-4         ------------------------------------------------------------
OsSRP-7         ------------------------------------------------------------
MtSRP-8         -NYNTELSFFFAENLFLK---------------K------NMVFSPFSLEMIFGIISA--
SbSRP-7         -QDEAAFSMRVLRHLASR---------DDGSPRA------NLAVSPLSLHAALALLAA--
OsSRP-3         -RDETAMAMRLLGHLARA--------PRGGGGDK------NLAVSPLSLHAALALLGA--
AtSRP-4         -EKQYKAMMDLKESVGNQ-------------NDI------VLRLTAPLINVILSIIAA--
AtSRP-7         -KNQTHVAMILSGHVLSS-----------APKDS------NVIFSPASINSAITMHAA--
AtSRP-1         -KNQNEVSLLLVGKVISA-----------VAKNS------NCVFSPASINAVLTVTAA--
AtSRP-6         -KKQNDVAIFLTGIVISS-----------VAKNS------NFVFSPASINAALTMVAASS
FvSRP-12        -----------------------------------------MVFSPLSLHFALSLIAS--
FvSRP-7         ------------------------------------------------------------
FvSRP-11        -TSQSNVALELTKQLFLT-----------EFKGQ------NMVFSPLSLHFSLSLVAS--
FvSRP-2         -MDLEALDMDLEADM-------------------------DLEALDMDLEADMDLEAP--
FvSRP-1         -SNQTDVAMKITKQLVES-----------EFKDK------NMIYSPLSIYIVLSLIAA--
FvSRP-10        -SHQTDVALKIAKQLLDS-----------EFKGK------NMIYSPLSIHIVLSLIAA--
FvSRP-9         -IKQTNVALQITKKLLET-----------EFHGK------NMIYSPQSIHSSVS------
FvSRP-14        -INQTNVALQITKQLLEQ-----------EFHGK------NMIYSPQSIHIVLSLIAA--
FvSRP-6         -KNQTDVALGITKHLLLT-----------LGKDK------NMVYSPLSIHIGLGMIMS--
FvSRP-4         VRNQSDVALRITKELLLT-----------KGKDK------NMVYSPLSIHVVLSLIAA--
FvSRP-13        -RDQTNVALEITKQLLLT---------E--GKNK------NVVFSPLSIHVVLSMIAV--
FvSRP-5         -QDQTKVALEITKQLLLN---------EKGNKDK------NVVFSPLSIHVVLSLIAA--
FvSRP-8         -QDQTNVALDITKQLLLN----------EKVKDK------NVVFSPLSIHVVLSMIAA--
AtSRP-2         -ENQNNVVARLAKKVIET----------DVANGS------NVVFSPMSINVLLSLIAA--
AtSRP-3         -ENHNDVVVRLTKHVIAT-----------VANGS------NLVFSPISINVLLSLIAA--
AtSRP-8         -ENQTDVMVLLAKHVIPT-----------VANGS------NLVFSPMSINVLLCLIAA--
SbSRP-16        -AQQTRFALCLAAAFSSP--------ALPVSNNT------NAVFSPLSLHVALSLLAA--
OsSRP-6b        ------------------------------------------------------------
OsSRP-5         -AHQTRFAFRLAAALSSP-RAHPAAGGAAGAGGS------NVAFSPLSLHVALSLVAA--
OsSRP-6a        -AHQTSFALRLAAALSSP-------AHPAGGAGR------NVAFSPLSLHVALSLVAA--
SbSRP-2         -AGQTRFALRLTAALSST---------AAAAPAR------NTAFSPLSLHVVLSLLAA--
SbSRP-3         -GHQTRFALRLAAALSSP--------SPSASPAG------NVAFSPLSLHVALSLLAA--
AtSRP-5         -SLQNQVSMNLAKHVITT-----------VSQNS------NVIFSPASINVVLSIIAA--
FvSRP-3         -ANQTDVGLGITKKLLQT-----------EGKDK------NVVYSPLSIHVVLSLIAA--
MtSRP-11        -ANLTKSSMNI--DLLSN----------PEFKER------NVVFSPLSLQTTLSMVTN--
MtSRP-9         -ANLTKNAMNITKHLVSK----------TEFKKK------NVVLSPLSLQTVLSIVAA--
```

```
MtSRP-6         -TNLTNVSMNITKHLLSN----------QKLKEK------NVVFSPLSLNTVLSMIAT--
MtSRP-16        -TNLTNVSLNITKHLLSN----------QKLNEK------NVVFSPLSLNTVLIMITA--
MtSRP-3         -SNVKAWEVS----------------------EK--------------------------
MtSRP-4         -SNLNKFSVTIAKHLFSK----------EEFKEK------NVVFSPLSLHVVLSIIAA--
MtSRP-10        -SNQTKVSLRIAKHLFSK------------ESEK------NIVF----------------
MtSRP-12        -TNQTKVSLRIAKYLFSK------------ESEK------NIVFSPLSLQVALSMIAA--
MtSRP-1         ------------------------------------------------------------
MtSRP-13        ------------------------------------------------------------
MtSRP-14        -TNQTNVSLRIAKHLFLK------------ELDK------NIVFSPLSLQVVLSIIAA--
MtSRP-5         -NNQNNVSLTIAKHLFSK----------ESHQDK------NMVLSPLSLQVVLSIIAT--
MtSRP-2         -NQHSSVSLTIAKHLFSK----------ESHRNK------NIVLSPLSLQVVLSIIAA--
MtSRP-7         -ANQTNVSLSVAKHLFSK------------ESDN------NIVFSPLSLQVVLSIIAS--
MtSRP-15        -TNQTKVSLNMAKHLFSK------------ESDK------NVVFSPLSLQVMLNIVAA--
SbSRP-12        -DGQTALALRLAKHLAPPPPGGGGAGYSATASAAAANNN-NVAFSPVSVHAALALTAA--
SbSRP-1         -AGLTSLALRLAKQLAAN------NKSSDRGNGG------NLVFSPLSIYAALALVAA--
SbSRP-9         -AGLTALALCFTRRLQLQAPPPVVDVAGEPPGGPSTAAAANLVFSPVSVYAALALLAA--
OsSRP-10        -SGLTALSMRLTKQLSAA-------AASKAGAAG------NLVFSPLSIYSVLSVVTA--
OsSRP-8         -SGLMGISLRLAEQFSAE--------EDGGGGGG------NLVFSPLSIYSALSVVTA--
OsSRP-12        -SGLAGMALRLAERLSLE---------EDSVGGG------NLVFSPLSIYSALTVVTA--
SbSRP-15        -SGLTAFALRLAKHLAGA----DKGAGVVGGGGQ------NIVFSPLSIYAALALLAA--
OsSRP-9         -GGMTAFALRLAKRLADV----------GVSSNK------NLVFSPASLYAALALVAA--
OsSRP-11        -GGLTAFALRLAKRLADD----------GDNSNR------NVVFSPVSLYAALALVAS--
OsSRP-15        -GGLTALAVRLADRLGAA------------SPGR------NLAFSPLSVHAALSLAAA--
SbSRP-10        -AGHAALSARLLKRISSE----------AAAVGN------NLVFSPLSIHVALVLMST--
OsSRP-13        -------------------------------MGR--------------------------
SbSRP-13        ----MDYCLQVAWYPGME----------AITNQS------NFIFSPMSLRAGLALLAV--
SbSRP-14        ----MDCCLKVAWYAGMK----------AITEQS------NFIFSPMSLRAGLALLAV--
OsSRP-1         ----MDQCLQVAWIAGSD----------AITEQS------NFIFSPMCLRAGLALLAT--
OsSRP-2         ----MDYCLQVAWIAGTK----------AITEQS------NFMFSPLGLRAGLALLAT--
SbSRP-5         -DDDATGCLRLARCVGRK----------AAGEGR------NFMLSPLSLHAALALVAA--
SbSRP-6         GADGGASCLPLARQAGVR-----------AAARN------NFIVSPLSFHAALALVAA--
OsSRP-4         AEEAKVSCLPLAREVGRR------AAAAGGGQGR------NFIVSPLSFHAALALVAD--


CrSRP-1         -GAGPKT-STHSELLAAIAGGAENAANLGE----GDLNSELGRTMTLLNGAGA-------
VcSRP-1         -GAGPKS-PTHAELLRVIAGD-NGHVSESEL---NSELGRAMAL-MNGQCA---------
SbSRP-11        -GA--RG-DTRRQVLQVLGGGGGGKGAAADA---ANVASRVVKR-VLKDRS---------
OsSRP-14        -GA--RG-ATRRQVLQALGCGGGGRGGAADA---ANVASRVVKR-VLRDRS---------
SmSRP-1         -GA--KG-QTLEQLTSVVRLP-----NGSLM---HDFAQQLNSV-LLGVAR---------
PpSRP-1         -GA--KG-PTLEQIANSIKIP-----HGDLM---HKFSTHLANI-LQSDGE---------
PpSRP-2         -GA--KG-PTREQISKCIKLP-----QGKPL---HDFSSHLRKT-VLSNQQ---------
PpSRP-3         -GA--KG-PTREQIAKCIKLP-----EGEPM---HNFSSQVKIA-LLADGS---------
PpSRP-4         -GA--KG-PTRGEIAKCIKLP-----EGDPM---HNFSSQLKTV-VLADGS---------
SbSRP-8         -GA--SG-ATRDQIATFLGPA-----GAEAH---AALASKVASA-VLAGRD---------
SbSRP-4         ------------------------------------------------------------
OsSRP-7         ------------------------------------------------------------
MtSRP-8         -GT--SG-DTQLELLRFLGLR-----SMKDLVDLVKIRSDLLKH-FSPDHSAL-------
SbSRP-7         -GA--RG-GTLDEIASFLGPA-----GGSAH---AALASYVALR-VFADGDGEGE-----
OsSRP-3         -GA--RG-ETLDQIIAFLGPA-----GGPAH---AALASHVALC-SLADDS------GPG
AtSRP-4         -SSPGDT-DTADKIVSLLQAS-----STDKL---HAVSSEIVTT-VLADST---------
AtSRP-7         -GP--GGDLVSGQILSFLRSS-----SIDEL---KTVFRELASV-VYADRS---------
AtSRP-1         -NT--DNKTLRSFILSFLKSS-----STEET---NAIFHELASV-VFKDGS---------
AtSRP-6         GGE--QGEELRSFILSFLKSS-----STDEL---NAIFREIASV-VLVDGS---------
FvSRP-12        -GT--KG-PCLEQFLSFLKSR-----STHHL---NSLAHDIVTF-VLANAS------PDE
FvSRP-7         ------------------------------------------------------------
FvSRP-11        -GT--KG-PCLDEFLSFLKSR-----STHHL---NSLAHDIVTF-VLASGSPSRPEIEER
FvSRP-2         -AM--DLEALQKPISNQTDVA-------------MKITKQLVES-EFKNKNMVYSQW---
FvSRP-1         -RT--NN-P---QLVYFLNSK-----SIDDL---NSIAFNLVTS-VLADST---------
FvSRP-10        -RT--NN-P---HFVSFLNSK-----SIDDL---NSLAYNLVTS-VLADTP---------
FvSRP-9         ------------------------------------------------------------
FvSRP-14        -RN--NC----PQLLSFLNSK-----SIAEL---NSLAGDLVTS-VLVDLP---------
FvSRP-6         -GT--KG-HIQDQFLSFLKSK-----SINEL---NDLASNVYPL-VFADGE---------
FvSRP-4         -GS-TKGHPLHDEMLKFLKSR-----SIEQL---NELASNLVPL-VFADGT---------
FvSRP-13        -GA--KG-SNQEKVLKFLKSE-----SINEL---NTLASVVAPL-VFADGS---------
FvSRP-5         -GA--KG-SNQKRMLSFLKSK-----SIKEL---NTLASRVVSL-VFADGS---------
FvSRP-8         -GA--KG-SIQKGMLSFLKSK-----SIDAL---NTIASNVVPL-VFADGS---------
AtSRP-2         -GS--NP-VTKEEILSFLMSP-----STDHL---NAVLAKIA-----DGGT---------
AtSRP-3         -GS--CS-VTKEQILSFLMLP-----STDHL---NLVLAQII-----DGGT---------
AtSRP-8         -GS--NC-VTKEQILSFIMLP-----SSDYL---NAVLAKTVSV-ALNDGM---------
SbSRP-16        -GS--GG-ATRDQLLAALAAGAHGPDAADSL---HALADQVARN-VMADGS---------
OsSRP-6b        ------------------------------------------------------------
OsSRP-5         -GA--GG-ATRDQLVSLLGVPGRG--TAEGL---HAFAEQVVQL-VLADSS---------
OsSRP-6a        -GA--GG-ATRDQLASALGGPG----SAEGL---HAFAEQLVQL-VLADAS---------
SbSRP-2         -GA---GHATRDQLLTALGGG-DGPVAAEIL---HALSEQVVQL-VLADGS---------
SbSRP-3         -GA--GG-ATRDQIAATLGGGGD---AAEGL---HALAEQVVQL-VLADGS---------
AtSRP-5         -GS--AG-ATKDQILSFLKFS-----STDQL---NSFSSEIVSA-VLADGS---------
FvSRP-3         -GS--KG-PTQEQILSFLKSK-----FAGDL---NSFASELVAV-IFADGS---------
MtSRP-11        -PS--TI-LTPCPLTSFP----------------MCLKTR--------------------
MtSRP-9         -GS--EG-PTQCQLLSFLGSK-----SIDHL---NSLSTHLFTS-VLDDAA---------
MtSRP-6         -GS--EG-PTQKQLLSFLQSE-----SPGNL---KSLYSRLVSS-VLSDGA---------
MtSRP-16        -GS--EG-PTQNQLLSFLQSE-----STGDL---KSLCSQLVSS-VLSDGA---------
MtSRP-3         ------------------------------------------------------------
MtSRP-4         -GA--EG-PTQDQLLTFLQSR-----STDEL---KSLSSQLVSY-LLADAT---------
```

```
MtSRP-10        ------------------------------------------------------------
MtSRP-12        -GS--DG-PTREQLLDFLLSK-----STDHL---NSFASHLVSA-IISNAA---------
MtSRP-1         ------------------------------------------------------------
MtSRP-13        ------------------------------------------------------------
MtSRP-14        -GS--EG-PTQQQLLDFLLSK-----STDHL---NDLASQLVSV-VLFDAA---------
MtSRP-5         -GS--EG-PTQQQLLNFLQSK-----STYQL---NSFASTLVSV-ILKDAA---------
MtSRP-2         -GS--DG-STQQQLLDFLQSN-----STDQL---NSFASKLVSV-ILKDGA---------
MtSRP-7         -GS--EG-PTQQQLFNFLQSK-----STDHL---NYFASQLVSV-ILSDAS---------
MtSRP-15        -AS--EG-RTQQQLLEFLRSK-----SIDHL---NSFTSHLVSI-ILSDAA---------
SbSRP-12        -GA--RG-ATLAQLLAFLGAP-----SAEEL---ADFGRRVADR-VLADRS---------
SbSRP-1         -GA--PG--------------------------AALDDSWLCS-ALGDGS---------
SbSRP-9         -GA--RG-STLQELLDALGGD-----SRDDL---TAFAGRAGER-ALADRSRSRSRAG--
OsSRP-10        -GA--RG-RTLTELLGALGAE-----SREKL---AANAGEMARALPAPGGGAAQ------
OsSRP-8         -GA--RG-TTLTELLAALGAP-----SRDAL---AKNAAEIAR--ALAGGT---------
OsSRP-12        -GA--RG-TTLAELLAALGAPS----SRDAL---AEDAGEIVRALPGGSGT---------
SbSRP-15        -GA--RG-TTLDEVLATLGAT-----SRDEI---AEFVSAVVER-ALADHSE--------
OsSRP-9         -GA--RG-TTLDELLALLGAA-----SLDDL---EESVRRAVEV-GLADES---------
OsSRP-11        -GA--RG-TTLDELVALLGAA-----SLDDL---EESVRRAVEV-GLADES---------
OsSRP-15        -GA--AG-GTLDEILAVLGAA-----SRDDL---AAFVGRTAET-ALADRG---------
SbSRP-10        -AA--AG-ATLDEILRVAGAP-----SREEL---AAFVRDTVVDGVLDDQS---------
OsSRP-13        -------------------------------------------VLADQA---------
SbSRP-13        -GT--QG-PTLRQLLTFLGSE-----NTHHL---DAATARLLTN----------------
SbSRP-14        -GT--HG-ATLRELLTFLGSE-----NTHHL---DAATARLLS-----------------
OsSRP-1         -GA--DG-ETLRQMLAFLGSE-----HIHQL---NATSAGLLAE----------------
OsSRP-2         -GT--DG-ETLRQLLAFLGSQ-----HIHQL---NAASAGLLAE----------------
SbSRP-5         -GA--NG-ETQAELLRFLGSA-----SLDELRR-AAVTRLVVAA----------------
SbSRP-6         -GA--RG-ETQRELLGFLGSAE----SLSEL---HGAAATALVA-RLNDLP---------
OsSRP-4         -GA--RG-ETQRELLGFLGSP-----SLAELH--RSPTTRLVAR----------------


CrSRP-1         -----GAAEGSASRMVLANSVWTHRGT-TLRKEYVD-AMQSLFDATARE-----------
VcSRP-1         -----SK-NGPSSEMVLANSLWT-RGM-TLKKAYVD-SMKSLFQATASE-----------
SbSRP-11        -----TS-GGP--RLAFAGGIWADTST-NLSPGFVE-AARSVYSSTARTADFNN------
OsSRP-14        -----TS-GGP--RLAFAGGVWADASR-SLSPEFVG-LAGNVYGSAAKKADFKN------
SmSRP-1         -----SDPRAP--ELSLANGVWVEQSL-KLRGEYKE-IIEKNYGASARPVDFKN------
PpSRP-1         --------QGL--ELSCANRIWVDQTI-QLKPTFQK-LLKDSYGAEAASVDFRH------
PpSRP-2         -----GD-GGP--ELALANRLWVEQSV-KLKPAFQK-ILQESYGSEAASVDFIS------
PpSRP-3         -----GA-GGP--QLSLANRIWVEQSV-KLKLEFQK-VLKDSYGSEAASVDFRT------
PpSRP-4         -----GA-GGP--ELALANRVWVDESV-TLKPEFQK-ILKDSYGSEAASVDFHA------
SbSRP-8         -----DG-GEGESKVRSATGVWVDATL-RLSPAFAD-TAAAIHKAEARSVNFRG------
SbSRP-4         -----------------MGVWVDAAL-RLNPAFAD-VAASQFRAAARKVAFSD------
OsSRP-7         -----------------MAVWVDASL-RLNPAFAD-TAASVFKAAVRSAG---------
MtSRP-8         -----HL-DGP--VFSFTNGLWVPKSL-PLKSSFKD-TLTTMFNAAVSDLHTYN------
SbSRP-7         -----GE-GGT--TVRFANGVWVAADL-QLKASFAR-VAAKHYRAEARQAFFKT------
OsSRP-3         D----DR-GGP--KVRFANGVWVDAAL-RLKAAYAR-VVADKYRAEARPVSFRD------
AtSRP-4         -----AS-GGP--TISAANGLWIEKTL-NVEPSFKD-LLLNSYKAAFNRVDFRT------
AtSRP-7         -----AT-GGP--KITAANGLWIDKSL-PTDPKFKD-LFENFFKAVYVPVDFRS------
AtSRP-1         -----ET-GGP--KIAAVNGVWMEQSL-SCNPDWED-LFLNFFKASFAKVDFRH------
AtSRP-6         -----KK-GGP--KIAVVNGMWMDQSL-SVNPLSKD-LFKNFFSAAFAQVDFRSKCNVLN
FvSRP-12        NDIIRG--PGP--RLSFANGVWVDKSL-PFKPDFKH-LVDIAYMAALEEVDFKF------
FvSRP-7         ------------------------------------------MAALEEVDFKS------
FvSRP-11        -----YG-PGP--RLSFANGVWVDKSL-PFKPYFKH-VVDTAYMAALEEVDFKS------
FvSRP-2         -----Q--ADP--RLNFTNGLWVDEST-PLEESYKK-VALDSYKAALN------------
FvSRP-1         -----SK-GGP--LLNFTNGLWVDESM-PLEESYKQ-VVLDSYKAALNEVDFKT------
FvSRP-10        -----SR-GGP--RLNFTNGLWVDEYT-PLEESYKK-VLLDLYKAALNEVDFKT------
FvSRP-9         -----------------------------LEESHKK-VVLDSYKAALNHVDIKT------
FvSRP-14        -----TR-GGP--RLNFTNGVWVDESE-PLDESYKK-VVLDSYKAALNHVDFKT------
FvSRP-6         -----SK-GGP--RISFANGVWVEESL-HVKPCFKE-ALDATYKGALNHVDFRT------
FvSRP-4         -----TS-GGP--SLAFANGVWVETSL-PVKASFKE-VLDNAYKAVLKQVDFRT------
FvSRP-13        -----EH-GGP--LLSIANGVWIDQSL-SIKHSFKN-VLETDYKAALKQVDFKN------
FvSRP-5         -----KL-GGP--LLSFANGVWMDQSL-SIKPSFQN-VLGTDYKAALKQVDFKT------
FvSRP-8         -----KL-GGP--LLSFANG-----------------VLDNDYKAALKQVDFKT------
AtSRP-2         -----ER-SDL--CLSTAHGVWIDKSS-YLKPSFKE-LLENSYKASCSQVDFAT------
AtSRP-3         -----EK-SDL--RLSIANGVWIDKFF-SLKLSFKD-LLENSYKATCSQVDFAS------
AtSRP-8         -----ER-SDL--HLSTAYGVWIDKSL-SFKPSFKD-LLENSYNATCNQVDFAT------
SbSRP-16        -----EA-GGP--RIAFADAVLADASW-KLNPAFQE-LAVGKYKAHTHSVDFQK------
OsSRP-6b        ------------------------------------------------------------
OsSRP-5         -----PA-GGP--RVAFADGVFIDSSL-SLMKSFKD-VAVGKYKAETHSVDFQT------
OsSRP-6a        -----GA-GGP--RVAFADGVFVDASL-SLKKTFGD-VAVGKYKAETHSVDFQT------
SbSRP-2         -----GV-GGP--RVAFADAVFVDASL-KLKSAFEE-VAVGKYKAETHSVDFQE------
SbSRP-3         -----GA-GGP--RVAFADGVFVDASL-KLKPAFQE-VAVGKYRADTQPVDFQK------
AtSRP-5         -----AN-GGP--KLSVANGAWIDKSL-SFKPSFKQ-LLEDSYKAASNQADFQS------
FvSRP-3         -----PT-GGP--RLSFANGIWVDQPL-ALKPSFKE-VVESAYKAAVNQVDFQT------
MtSRP-11        ---------GL--SLSSAYAIWVEKTL-SLYPSFKE-TIAINYKATLQSHDFIN------
MtSRP-9         -----PF-GGP--QLSFVNSVWFEKSL-SLYPSFKE-IVDTNYFATLRSLDFIN------
MtSRP-6         -----PA-GGP--CLSYVNGVWVEQSL-PLQPSFKQ-LMTTDFKATLAEVDFVN------
MtSRP-16        -----PA-GGP--CLSHVNGVWVEQSL-PLQPSFKQ-LMTTDFKATLAAVDFIN------
MtSRP-3         ----------------------------------------------------S------
MtSRP-4         -----PA-GGP--LLSFVNGVWVEQSL-TFQHSFKE-TVATDFKSNTASVDFVN------
MtSRP-10        ------------------------------FPTF--------------------------
MtSRP-12        -----PS-GGP--CLSFLNGVWVDQSR-SLQPSFQQ-IVSNDYKATLSSVDFKN------
MtSRP-1         ------------------------------------------------------------
MtSRP-13        ---------------MFVIGVFSH------------------------------------
```

```
MtSRP-14        -----PT-GGP--RFSFVNGVWVEQTL-SLQPSFKE-IVSSDYKATLLSVDFKT------
MtSRP-5         -----PASNEL--CLMISRTRYV-----SISPLF---SLQNTY-----------------
MtSRP-2         -----PA-GGP--RLSFVDGVWVEKTL-SLQPSFKQ-IVSNDYKANLSSVDFKN------
MtSRP-7         -----PA-GGP--LLSFVDGVWVDQTL-SLQPSFQQ-IVSTHFKAALSSVDFQN------
MtSRP-15        -----PS-GGS--RLSFTQRVWVDQTL-SLQPSFKE-TMVTDYKATLASVDFQN------
SbSRP-12        -----DA-GGP--RVLFGGGVWVDAARGGLTEAFRD-VAAEAYKSEARTVSFTE------
SbSRP-1         -----GS-GGP--LIASACGVWHDDEM-VLKPVFRA-TAVECFKAEVRAVDFQT------
SbSRP-9         -----AV-GGP--AVAFACGAWLDAAW-ALLPAFRDAAAAASYNAEARAVDFGN------
OsSRP-10        -----PG-GGP--RVAHACGVWHERTR-TVRPAFRD-AAAASFNAAALAVDFLN------
OsSRP-8         -----AT-GGP--RVAHACGLWHERTR-SLKLAFRD-AAAASFNAATRAVDFLA------
OsSRP-12        -----AT-GGP--RVAHACGLWHDRRR-NVKPAFRD-AAAASFQATTRAVDFLA------
SbSRP-15        -----TG-SAP--RVAFACALWHEKMM-ALKPAYRA-AAVQSYKAETHAADFVN------
OsSRP-9         -----AS-GGP--RVSDACGVWHDETL-ELKPAYRA-AAAGTYKAVTRAANFQR------
OsSRP-11        -----ES-GGP--RVSYACGVWHDERL-ALKPAYRA-------------ADFQR------
OsSRP-15        -----PESLGP--RVVFAPGVWCDAAR-PFKPAYRA-AVAAEYNAEATVVDFKN------
SbSRP-10        -----GI-GGP--TISYACGTWTDKAW-PLRPANVD-AIVGTFKGNSWALTSKT------
OsSRP-13        -----PT-GGP--CVSFACGSWLDASY-SLKPAYRD-AIVGTYKGAASTVDFKN------
SbSRP-13        -----VS-TWP--QLSFAAGIFVDRTL-FLAPEFVS-SAVSAHYAVARSVDFKN------
SbSRP-14        -------------NLSFAAGIFVDRTL-LLKPETRS---------SCPPPPLLT------
OsSRP-1         -----MQ-AWP--QLVFAAGIFVDRSL-RLRPEFKS-TAAAAHGGIHAICGLPE------
OsSRP-2         -----MR-AWP--QLSFAAGIFVDRSL-RLRPEFQS-TAAAAHGAFPRSVDFQN------
SbSRP-5         -----LR-GIP--QTSFACGVWVDRRC-PLREEFAD-VAGAVYAAVAESVDFVS------
SbSRP-6         -------------QTSFACGVWVDRRR-ALTPEFRD-AAASRYAAVADSVDFAS------
OsSRP-4         -----LR-HLP--NTSFACGVWVDRGR-ALTPEFAD-AAASRYAAVAEPADFAT------


CrSRP-1         ----------------------------------------------------------AV
VcSRP-1         ----------------------------------------------------------AI
SbSRP-11        ---------------------------K------------------------------PE
OsSRP-14        ---------------------------K------------------------------PE
SmSRP-1         ---------------------------K------------------------------AQ
PpSRP-1         ---------------------------K------------------------------ST
PpSRP-2         ---------------------------K------------------------------AA
PpSRP-3         ---------------------------K------------------------------AD
PpSRP-4         ---------------------------K------------------------------AD
SbSRP-8         ---------------------------N------------------------------LR
SbSRP-4         ---------------------------N------------------------------PE
OsSRP-7         ---------------------------N------------------------------PA
MtSRP-8         ---------------------------R------------------------------GV
SbSRP-7         ---------------------------M------------------------------PE
OsSRP-3         ---------------------------K------------------------------LE
AtSRP-4         ---------------------------K------------------------------AD
AtSRP-7         ---------------------------E------------------------------AE
AtSRP-1         ---------------------------K------------------------------AE
AtSRP-6         KLGLAVSLLESIFHISTLFDFKFALIRS------------------------------AE
FvSRP-12        ---------------------------N------------------------------PK
FvSRP-7         ---------------------------N------------------------------PK
FvSRP-11        ---------------------------N------------------------------PK
FvSRP-2         ------------------------------------------------------------
FvSRP-1         ---------------------------D------------------------------PE
FvSRP-10        ---------------------------D------------------------------PE
FvSRP-9         ---------------------------N------------------------------PE
FvSRP-14        ---------------------------N------------------------------PK
FvSRP-6         ---------------------------R------------------------------AE
FvSRP-4         ---------------------------K------------------------------PE
FvSRP-13        ---------------------------E------------------------------PE
FvSRP-5         ---------------------------K------------------------------PE
FvSRP-8         ---------------------------E------------------------------PE
AtSRP-2         ---------------------------K------------------------------PV
AtSRP-3         ---------------------------K------------------------------PS
AtSRP-8         ---------------------------K------------------------------PA
SbSRP-16        ---------------------------K------------------------------AA
OsSRP-6b        ------------------------------------------------------------
OsSRP-5         ---------------------------K------------------------------AA
OsSRP-6a        ---------------------------KWLLL--------------------------PS
SbSRP-2         ---------------------------K------------------------------AA
SbSRP-3         ---------------------------K------------------------------AA
AtSRP-5         ---------------------------K------------------------------AV
FvSRP-3         ---------------------------K------------------------------AA
MtSRP-11        ---------------------------K------------------------------PD
MtSRP-9         ---------------------------K------------------------------AD
MtSRP-6         ---------------------------K------------------------------AD
MtSRP-16        ---------------------------K------------------------------AD
MtSRP-3         ---------------------------K------------------------------AL
MtSRP-4         ---------------------------K------------------------------AF
MtSRP-10        ----------------------------------------------------------PA
MtSRP-12        ---------------------------K------------------------------AT
MtSRP-1         ------------------------------------------------------------
MtSRP-13        ------------------------------------------------------------
MtSRP-14        ---------------------------K------------------------------AV
MtSRP-5         ----------------------------------------------------------AV
MtSRP-2         ---------------------------KICFYICSLF---------------------TT
MtSRP-7         ---------------------------K------------------------------AV
```

```
MtSRP-15        -------KVFSSFAGLAPYPASSDVHENEMLNQFAQWILSIGDGKVGDDTDGEAVVEIPS
SbSRP-12        ---------------------------E------------------------------PE
SbSRP-1         ---------------------------K------------------------------AE
SbSRP-9         ---------------------------Q------------------------------PA
OsSRP-10        ---------------------------N------------------------------PE
OsSRP-8         ---------------------------N------------------------------PE
OsSRP-12        ---------------------------N------------------------------PE
SbSRP-15        ---------------------------K------------------------------PE
OsSRP-9         ---------------------------Q------------------------------PK
OsSRP-11        ---------------------------Q------------------------------PK
OsSRP-15        ---------------------------KVGYSILIDAFILLLDPSMIDQCVLDLVSEKAE
SbSRP-10        ---------------------------SSILSLHHTILLRC-----------------PK
OsSRP-13        ---------------------------H------------------------------PV
SbSRP-13        ---------------------------Q------------------------------PA
SbSRP-14        ---------------------------R------------------------------PS
OsSRP-1         ----------------------------------------------------------PD
OsSRP-2         ---------------------------Q------------------------------AN
SbSRP-5         ---------------------------Q------------------------------AE
SbSRP-6         ---------------------------E------------------------------PE
OsSRP-4         ---------------------------Q------------------------------PE


CrSRP-1         N-----------------------------------------------------------
VcSRP-1         N-----------------------------------------------------------
SbSRP-11        D-----------------------------------------------------------
OsSRP-14        D-----------------------------------------------------------
SmSRP-1         E-----------------------------------------------------------
PpSRP-1         E-----------------------------------------------------------
PpSRP-2         E-----------------------------------------------------------
PpSRP-3         E-----------------------------------------------------------
PpSRP-4         E-----------------------------------------------------------
SbSRP-8         G-----------------------------------------------------------
SbSRP-4         A-----------------------------------------------------------
OsSRP-7         A-----------------------------------------------------------
MtSRP-8         E-----------------------------------------------------------
SbSRP-7         E-----------------------------------------------------------
OsSRP-3         E-----------------------------------------------------------
AtSRP-4         E-----------------------------------------------------------
AtSRP-7         E-----------------------------------------------------------
AtSRP-1         E-----------------------------------------------------------
AtSRP-6         E-----------------------------------------------------------
FvSRP-12        Q-----------------------------------------------------------
FvSRP-7         Q-----------------------------------------------------------
FvSRP-11        Q-----------------------------------------------------------
FvSRP-2         ------------------------------------------------------------
FvSRP-1         Q-----------------------------------------------------------
FvSRP-10        K-----------------------------------------------------------
FvSRP-9         Q-----------------------------------------------------------
FvSRP-14        Q-----------------------------------------------------------
FvSRP-6         E-----------------------------------------------------------
FvSRP-4         E-----------------------------------------------------------
FvSRP-13        E-----------------------------------------------------------
FvSRP-5         E-----------------------------------------------------------
FvSRP-8         E-----------------------------------------------------------
AtSRP-2         E-----------------------------------------------------------
AtSRP-3         E-----------------------------------------------------------
AtSRP-8         E-----------------------------------------------------------
SbSRP-16        E-----------------------------------------------------------
OsSRP-6b        ------------------------------------------------------------
OsSRP-5         E-----------------------------------------------------------
OsSRP-6a        NLANIDWEHYLLLPSLEKKLEKYGKIFLVFTIVGIIYVIMWFPFTAILEIMSLKDDEPIT
SbSRP-2         Q-----------------------------------------------------------
SbSRP-3         E-----------------------------------------------------------
AtSRP-5         E-----------------------------------------------------------
FvSRP-3         E-----------------------------------------------------------
MtSRP-11        E-----------------------------------------------------------
MtSRP-9         E-----------------------------------------------------------
MtSRP-6         E-----------------------------------------------------------
MtSRP-16        E-----------------------------------------------------------
MtSRP-3         E-----------------------------------------------------------
MtSRP-4         E-----------------------------------------------------------
MtSRP-10        E-----------------------------------------------------------
MtSRP-12        E-----------------------------------------------------------
MtSRP-1         ------------------------------------------------------------
MtSRP-13        ------------------------------------------------------------
MtSRP-14        EVTKEGNLWAEKETNGLIKELLPPRSVDSLTRLIFANALYFKGAWSEKFDVLKTKDYDFH
MtSRP-5         E-----------------------------------------------------------
MtSRP-2         KFKCINF-----------------------------------------------------
MtSRP-7         E-----------------------------------------------------------
MtSRP-15        DLLVDHSGDPIGDIVAATYPGVVENLVDATFFQDRAILAPTLELVEKVNDYVMAMIPGDE
SbSRP-12        A-----------------------------------------------------------
SbSRP-1         E-----------------------------------------------------------
SbSRP-9         EK----------------------------------------------------------
```

```
OsSRP-10        E-----------------------------------------------------------
OsSRP-8         E-----------------------------------------------------------
OsSRP-12        E-----------------------------------------------------------
SbSRP-15        K-----------------------------------------------------------
OsSRP-9         R-----------------------------------------------------------
OsSRP-11        S-----------------------------------------------------------
OsSRP-15        E-----------------------------------------------------------
SbSRP-10        E-----------------------------------------------------------
OsSRP-13        E-----------------------------------------------------------
SbSRP-13        A-----------------------------------------------------------
SbSRP-14        HSLSISRTF---------------------------------------------------
OsSRP-1         HEGA--------------------------------------------------------
OsSRP-2         A-----------------------------------------------------------
SbSRP-5         E-----------------------------------------------------------
SbSRP-6         A-----------------------------------------------------------
OsSRP-4         Q-----------------------------------------------------------


CrSRP-1         ------------------------------------------------------------
VcSRP-1         ------------------------------------------------------------
SbSRP-11        ------------------------------------------------------------
OsSRP-14        ------------------------------------------------------------
SmSRP-1         ------------------------------------------------------------
PpSRP-1         ------------------------------------------------------------
PpSRP-2         ------------------------------------------------------------
PpSRP-3         ------------------------------------------------------------
PpSRP-4         ------------------------------------------------------------
SbSRP-8         ------------------------------------------------------------
SbSRP-4         ------------------------------------------------------------
OsSRP-7         ------------------------------------------------------------
MtSRP-8         ------------------------------------------------------------
SbSRP-7         ------------------------------------------------------------
OsSRP-3         ------------------------------------------------------------
AtSRP-4         ------------------------------------------------------------
AtSRP-7         ------------------------------------------------------------
AtSRP-1         ------------------------------------------------------------
AtSRP-6         ------------------------------------------------------------
FvSRP-12        ------------------------------------------------------------
FvSRP-7         ------------------------------------------------------------
FvSRP-11        ------------------------------------------------------------
FvSRP-2         ------------------------------------------------------------
FvSRP-1         ------------------------------------------------------------
FvSRP-10        ------------------------------------------------------------
FvSRP-9         ------------------------------------------------------------
FvSRP-14        ------------------------------------------------------------
FvSRP-6         ------------------------------------------------------------
FvSRP-4         ------------------------------------------------------------
FvSRP-13        ------------------------------------------------------------
FvSRP-5         ------------------------------------------------------------
FvSRP-8         ------------------------------------------------------------
AtSRP-2         ------------------------------------------------------------
AtSRP-3         ------------------------------------------------------------
AtSRP-8         ------------------------------------------------------------
SbSRP-16        ------------------------------------------------------------
OsSRP-6b        ------------------------------------------------------------
OsSRP-5         ------------------------------------------------------------
OsSRP-6a        HWTSGKAAE---------------------------------------------------
SbSRP-2         ------------------------------------------------------------
SbSRP-3         ------------------------------------------------------------
AtSRP-5         ------------------------------------------------------------
FvSRP-3         ------------------------------------------------------------
MtSRP-11        ------------------------------------------------------------
MtSRP-9         ------------------------------------------------------------
MtSRP-6         ------------------------------------------------------------
MtSRP-16        ------------------------------------------------------------
MtSRP-3         ------------------------------------------------------------
MtSRP-4         ------------------------------------------------------------
MtSRP-10        ------------------------------------------------------------
MtSRP-12        ------------------------------------------------------------
MtSRP-1         ------------------------------------------------------------
MtSRP-13        ------------------------------------------------------------
MtSRP-14        LLNGSSVKVPFMTSEENQFIEAYGGFKVLRLPYKKGKDKRRFSMYIFLPNAKDGLPALVE
MtSRP-5         ------------------------------------------------------------
MtSRP-2         ------------------------------------------------------------
MtSRP-7         ------------------------------------------------------------
MtSRP-15        KEYLSCDSVCKCDDDIGVDHRWITTDFLNDIKCSGMPNHRLCLKVGVPVMLLRNVDQASG
SbSRP-12        ------------------------------------------------------------
SbSRP-1         ------------------------------------------------------------
SbSRP-9         ------------------------------------------------------------
OsSRP-10        ------------------------------------------------------------
OsSRP-8         ------------------------------------------------------------
OsSRP-12        ------------------------------------------------------------
SbSRP-15        ------------------------------------------------------------
```

```
OsSRP-9         ------------------------------------------------------------
OsSRP-11        ------------------------------------------------------------
OsSRP-15        ------------------------------------------------------------
SbSRP-10        ------------------------------------------------------------
OsSRP-13        ------------------------------------------------------------
SbSRP-13        ------------------------------------------------------------
SbSRP-14        ------------------------------------------------------------
OsSRP-1         ------------------------------------------------------------
OsSRP-2         ------------------------------------------------------------
SbSRP-5         ------------------------------------------------------------
SbSRP-6         ------------------------------------------------------------
OsSRP-4         ------------------------------------------------------------


CrSRP-1         ------------------------------------------------------------
VcSRP-1         ------------------------------------------------------------
SbSRP-11        ------------------------------------------------------------
OsSRP-14        ------------------------------------------------------------
SmSRP-1         ------------------------------------------------------------
PpSRP-1         ------------------------------------------------------------
PpSRP-2         ------------------------------------------------------------
PpSRP-3         ------------------------------------------------------------
PpSRP-4         ------------------------------------------------------------
SbSRP-8         ------------------------------------------------------------
SbSRP-4         ------------------------------------------------------------
OsSRP-7         ------------------------------------------------------------
MtSRP-8         ------------------------------------------------------------
SbSRP-7         ------------------------------------------------------------
OsSRP-3         ------------------------------------------------------------
AtSRP-4         ------------------------------------------------------------
AtSRP-7         ------------------------------------------------------------
AtSRP-1         ------------------------------------------------------------
AtSRP-6         ------------------------------------------------------------
FvSRP-12        ------------------------------------------------------------
FvSRP-7         ------------------------------------------------------------
FvSRP-11        ------------------------------------------------------------
FvSRP-2         ------------------------------------------------------------
FvSRP-1         ------------------------------------------------------------
FvSRP-10        ------------------------------------------------------------
FvSRP-9         ------------------------------------------------------------
FvSRP-14        ------------------------------------------------------------
FvSRP-6         ------------------------------------------------------------
FvSRP-4         ------------------------------------------------------------
FvSRP-13        ------------------------------------------------------------
FvSRP-5         ------------------------------------------------------------
FvSRP-8         ------------------------------------------------------------
AtSRP-2         ------------------------------------------------------------
AtSRP-3         ------------------------------------------------------------
AtSRP-8         ------------------------------------------------------------
SbSRP-16        ------------------------------------------------------------
OsSRP-6b        ------------------------------------------------------------
OsSRP-5         ------------------------------------------------------------
OsSRP-6a        ------------------------------------------------------------
SbSRP-2         ------------------------------------------------------------
SbSRP-3         ------------------------------------------------------------
AtSRP-5         ------------------------------------------------------------
FvSRP-3         ------------------------------------------------------------
MtSRP-11        ------------------------------------------------------------
MtSRP-9         ------------------------------------------------------------
MtSRP-6         ------------------------------------------------------------
MtSRP-16        ------------------------------------------------------------
MtSRP-3         ------------------------------------------------------------
MtSRP-4         ------------------------------------------------------------
MtSRP-10        ------------------------------------------------------------
MtSRP-12        ------------------------------------------------------------
MtSRP-1         ------------------------------------------------------------
MtSRP-13        ------------------------------------------------------------
MtSRP-14        KLAAKPHLLHYSLHPAEAEVVEFRIPRFKISFELETSDMFKELGVILPFTRGGLTKMVDS
MtSRP-5         ------------------------------------------------------------
MtSRP-2         ------------------------------------------------------------
MtSRP-7         ------------------------------------------------------------
MtSRP-15        LCNGTRLIIVSLGKNVICARVIGGTHAGEVSYIPRMNLIPSGANVSITFERCQFPLVLSF
SbSRP-12        ------------------------------------------------------------
SbSRP-1         ------------------------------------------------------------
SbSRP-9         ------------------------------------------------------------
OsSRP-10        ------------------------------------------------------------
OsSRP-8         ------------------------------------------------------------
OsSRP-12        ------------------------------------------------------------
SbSRP-15        ------------------------------------------------------------
OsSRP-9         ------------------------------------------------------------
OsSRP-11        ------------------------------------------------------------
OsSRP-15        ------------------------------------------------------------
SbSRP-10        ------------------------------------------------------------
```

```
OsSRP-13        ------------------------------------------------------------
SbSRP-13        ------------------------------------------------------------
SbSRP-14        ------------------------------------------------------------
OsSRP-1         ------------------------------------------------------------
OsSRP-2         ------------------------------------------------------------
SbSRP-5         ------------------------------------------------------------
SbSRP-6         ------------------------------------------------------------
OsSRP-4         ------------------------------------------------------------


CrSRP-1         ------------------------------------------------------------
VcSRP-1         ------------------------------------------------------------
SbSRP-11        ------------------------------------------------------------
OsSRP-14        ------------------------------------------------------------
SmSRP-1         ------------------------------------------------------------
PpSRP-1         ------------------------------------------------------------
PpSRP-2         ------------------------------------------------------------
PpSRP-3         ------------------------------------------------------------
PpSRP-4         ------------------------------------------------------------
SbSRP-8         ------------------------------------------------------------
SbSRP-4         ------------------------------------------------------------
OsSRP-7         ------------------------------------------------------------
MtSRP-8         ------------------------------------------------------------
SbSRP-7         ------------------------------------------------------------
OsSRP-3         ------------------------------------------------------------
AtSRP-4         ------------------------------------------------------------
AtSRP-7         ------------------------------------------------------------
AtSRP-1         ------------------------------------------------------------
AtSRP-6         ------------------------------------------------------------
FvSRP-12        ------------------------------------------------------------
FvSRP-7         ------------------------------------------------------------
FvSRP-11        ------------------------------------------------------------
FvSRP-2         ------------------------------------------------------------
FvSRP-1         ------------------------------------------------------------
FvSRP-10        ------------------------------------------------------------
FvSRP-9         ------------------------------------------------------------
FvSRP-14        ------------------------------------------------------------
FvSRP-6         ------------------------------------------------------------
FvSRP-4         ------------------------------------------------------------
FvSRP-13        ------------------------------------------------------------
FvSRP-5         ------------------------------------------------------------
FvSRP-8         ------------------------------------------------------------
AtSRP-2         ------------------------------------------------------------
AtSRP-3         ------------------------------------------------------------
AtSRP-8         ------------------------------------------------------------
SbSRP-16        ------------------------------------------------------------
OsSRP-6b        ------------------------------------------------------------
OsSRP-5         ------------------------------------------------------------
OsSRP-6a        ------------------------------------------------------------
SbSRP-2         ------------------------------------------------------------
SbSRP-3         ------------------------------------------------------------
AtSRP-5         ------------------------------------------------------------
FvSRP-3         ------------------------------------------------------------
MtSRP-11        ------------------------------------------------------------
MtSRP-9         ------------------------------------------------------------
MtSRP-6         ------------------------------------------------------------
MtSRP-16        ------------------------------------------------------------
MtSRP-3         ------------------------------------------------------------
MtSRP-4         ------------------------------------------------------------
MtSRP-10        ------------------------------------------------------------
MtSRP-12        ------------------------------------------------------------
MtSRP-1         ------------------------------------------------------------
MtSRP-13        ------------------------------------------------------------
MtSRP-14        PLVGNSLSVSKIFHKSFIEVNEEGTEAAAASAGCYEEELDSKERIDFVADHPFLFLIREN
MtSRP-5         ------------------------------------------------------------
MtSRP-2         ------------------------------------------------------------
MtSRP-7         ------------------------------------------------------------
MtSRP-15        AMTINKSQGQTLTSVGLYLPRPVFTHGQLYVAVSRVKSRSGLKILITDENGSPSSSTVNV
SbSRP-12        ------------------------------------------------------------
SbSRP-1         ------------------------------------------------------------
SbSRP-9         ------------------------------------------------------------
OsSRP-10        ------------------------------------------------------------
OsSRP-8         ------------------------------------------------------------
OsSRP-12        ------------------------------------------------------------
SbSRP-15        ------------------------------------------------------------
OsSRP-9         ------------------------------------------------------------
OsSRP-11        ------------------------------------------------------------
OsSRP-15        ------------------------------------------------------------
SbSRP-10        ------------------------------------------------------------
OsSRP-13        ------------------------------------------------------------
SbSRP-13        ------------------------------------------------------------
SbSRP-14        ------------------------------------------------------------
OsSRP-1         ------------------------------------------------------------
```

```
OsSRP-2         ------------------------------------------------------------
SbSRP-5         ------------------------------------------------------------
SbSRP-6         ------------------------------------------------------------
OsSRP-4         ------------------------------------------------------------


CrSRP-1         ------------------GAKDVNAW--VEGVTKGMIK----------------------
VcSRP-1         ------------------GAKDINDW--VEGATRGMIK----------------------
SbSRP-11        ------------------AAKQINMW--VKESTKGTAT----------------------
OsSRP-14        ------------------APDQINSW--VKDSTKGTVT----------------------
SmSRP-1         ------------------SRGLVNSW--VAEATKKKIE----------------------
PpSRP-1         ------------------ARETINKW--AENKTHGKIA----------------------
PpSRP-2         ------------------ALAKVNKW--AKDETHGKIE----------------------
PpSRP-3         ------------------ARGKVNEW--AKEETHGKIE----------------------
PpSRP-4         ------------------ALAKVNEW--AKEATHEKIE----------------------
SbSRP-8         ------------------ATAEINEW--YERTTGGLIK----------------------
SbSRP-4         ------------------AREEINGW--FRSETGGLVQPDEREIDSEDEEQLVQFDMDEK
OsSRP-7         ------------------ARAEINEW--FSSQTGGFVK----------------------
MtSRP-8         ------------------VIEEVNSW--VTRKTNGIIK----------------------
SbSRP-7         ------------------ARSQINEW--VASATAGRIK----------------------
OsSRP-3         ------------------ARREINEW--FESATAGRIK----------------------
AtSRP-4         ------------------VNREVNSW--VEKQTNGLIT----------------------
AtSRP-7         ------------------VRKEVNSW--VEHHTNNLIK----------------------
AtSRP-1         ------------------VRLDVNTW--ASRHTNDLIK----------------------
AtSRP-6         ------------------VRTEVNAW--ASSHTNGLIK----------------------
FvSRP-12        ------------------VSVEVNSW--AEKETNGLIK----------------------
FvSRP-7         ------------------VTVEVNSW--VEKETNGLIK----------------------
FvSRP-11        ------------------VSIQVNSW--AEKETNGLIK----------------------
FvSRP-2         ------------------------------------------------------------
FvSRP-1         ------------------VRIQVNSW--VEEQTKGRIP----------------------
FvSRP-10        ------------------VRIQVNSW--VEKETRGLTP----------------------
FvSRP-9         ------------------LRSQVNAW--VGKETKGLIP----------------------
FvSRP-14        ------------------VRSQVNAW--VKKETKGLIP----------------------
FvSRP-6         ------------------VRYEVNSW--VDKKTSGLIK----------------------
FvSRP-4         ------------------ARCEVNSW--VEKVTRGLIK----------------------
FvSRP-13        ------------------VRREVNSW--GEEETKGLIE----------------------
FvSRP-5         ------------------VRREVNSW--GKKETKGLIE----------------------
FvSRP-8         ------------------VRREVNSW--GEKETKGLIK----------------------
AtSRP-2         ------------------VIDEVNIW--ADVHTNGLIK----------------------
AtSRP-3         ------------------VIDEVNTW--AEVHTNGLIK----------------------
AtSRP-8         ------------------VINEVNAW--AEVHTNGLIK----------------------
SbSRP-16        ------------------VAAQVNTW--VEEVTSGTIK----------------------
OsSRP-6b        ------------------VLGQVNSW--VDRVTSGLIK----------------------
OsSRP-5         ------------------VASQVNSW--VDRVTSGLIK----------------------
OsSRP-6a        ------------------VASQVNSW--VEKVTSGLIK----------------------
SbSRP-2         ------------------VAGEVNSW--VEKVTSGLIK----------------------
SbSRP-3         ------------------AAGKVNSW--VEKITSGLIK----------------------
AtSRP-5         ------------------VIAEVNSW--AEKETNGLIT----------------------
FvSRP-3         ------------------VAAGVNLW--AEKETSGLIK----------------------
MtSRP-11        ------------------AVKKVNLW--ATEKTNGLIT----------------------
MtSRP-9         ------------------AVKKVNAW--AKKETNGRIG----------------------
MtSRP-6         ------------------VRKEVNVW--AEKETKGLIK----------------------
MtSRP-16        ------------------VIKEVNLW--ANKETKGFIN----------------------
MtSRP-3         ------------------VRKEVNLW--AEKKDK--------------------------
MtSRP-4         ------------------GRKEVNLW--AEKETNGLIK----------------------
MtSRP-10        ------------------ALKEVNLW--AEKETNGLIK----------------------
MtSRP-12        ------------------VLQEVNLW--AEKETNGLIK----------------------
MtSRP-1         ------------------------------------------------------------
MtSRP-13        ------------------------------------------------------------
MtSRP-14        STGTILFVGQVDEGDAIEVTKEVNLW--AEKETNGLIK----------------------
MtSRP-5         ------------------VNNEVNLW--AERETNGLIK----------------------
MtSRP-2         ------------------VFGQRSEFM-GCKKTNGIIK----------------------
MtSRP-7         ------------------VTNEVNSW--AEKETNGLIK----------------------
MtSRP-15        VYQEC-------------LTNEVNIW--AEKETNGLIK----------------------
SbSRP-12        ------------------AVEMINSW--VKKATDNLIN----------------------
SbSRP-1         ------------------ARQEINSW--VSEATKGLIT----------------------
SbSRP-9         ------------------AVGEINRC--VAAATNNHID----------------------
OsSRP-10        ------------------ARKEINSW--VAAATENLID----------------------
OsSRP-8         ------------------ARKEINSW--VAAATENLID----------------------
OsSRP-12        ------------------ARNEINSW--VAAATENLID----------------------
SbSRP-15        ------------------AREKINRW--VSKATKGLIT----------------------
OsSRP-9         ------------------SRKKINKW--VSKATNKLIP----------------------
OsSRP-11        ------------------SRKKINKW--VSKATNKLIR----------------------
OsSRP-15        ------------------ARKQINAW--ARRATGKLIT----------------------
SbSRP-10        ------------------SRKQINAW--AAKATRNLIT----------------------
OsSRP-13        ------------------ARKEINAW--VARATKNLIT----------------------
SbSRP-13        ------------------ATAEMNAF--VEQATAGRIR----------------------
SbSRP-14        ------------------LTAEMNAF--VEQATAGRIR----------------------
OsSRP-1         ------------------LNQRHPPW--HLEQRHDVRPC---------------------
OsSRP-2         ------------------AAAEVNRF--ISQATNGRLN----------------------
SbSRP-5         ------------------ARQRINDFVNVKDATKGLIG----------------------
SbSRP-6         ------------------ARRRVNAF--VGEATRGLIG----------------------
OsSRP-4         ------------------ARERVNAF--VSDATEGLIR----------------------
```

```
CrSRP-1         ----DL-VQ------TDQWDA---------ILANAIYFKGFWTHA--FKKEDTFPGEFT-
VcSRP-1         ----DL-IQ------TE---------NFHAVLANAIYFKGLWEHA--FKPELTQQGEFTT
SbSRP-11        ----SL-LL------DGLIDQ-----NTGLVIGSALYFRGRWLDR--ADIRSTAVQKFCC
OsSRP-14        ----TL-LP------AGTIDQ-----NTGLVLGSALYFRGRWLDR--DDLRRTTEQKFYC
SmSRP-1         ----EL-LP------EGSVDP-----QTRLILASAIYFKGAWQKQ--LDPSQTRDGIFHL
PpSRP-1         ----NV-LP------PDAVSA-----HTKAILANAIYFNGSWENR--FDSSMTKDDDFHL
PpSRP-2         ----NL-LP------AGSVDH-----DTRVVLANALYFKGAWKKQ--FDDYHTREEDFYL
PpSRP-3         ----DL-LP------PGSVDQ-----GTHIVLANALYFKGAWKKP--FEEKDTKDGEFFL
PpSRP-4         ----EL-LP------TGSVDQ-----MTRVVLANALYFKGAWKKP--FEDEDTKHGEFFL
SbSRP-8         ----NM-LS------EGDCHA-----STAVVVGNSVYFDGYWRDP--FIPKYTEEGPFYV
SbSRP-4         SLFDDI-LA------EGSIDA-----GTAVVLASSLYFNCNWYDP--FYPSGTEEGTFHV
OsSRP-7         ----DI-LSNSIDDDDDGSGGGGGAISASVFLANSLYFNAYWDHP--FFPHLTEEGDFHV
MtSRP-8         ----GI-LQ------PMSITN-----ETQLLFINTVCFKGEWENP--FPTCLTALHDFTL
SbSRP-7         ----DL-LP------TGCLHR-----GTPAVLANALYFKGAWERK--FDACLTRDEAFFL
OsSRP-3         ----DF-LP------KDAVDR-----ATPAVLGNALYFKGDWESK--FDARSTSDDVFYL
AtSRP-4         ----NL-LP------SNPKSA----PLTDHIFANALFFNGRWDSQ--FDPSLTKDSDFHL
AtSRP-7         ----DL-LP------DGSVTS-----LTNKIYANALSFKGAWKRP--FEKYYTRDNDFYL
AtSRP-1         ----EI-LP------RGSVTS-----LTNWIYGNALYFKGAWEKA--FDKSMTRDKPFHL
AtSRP-6         ----DL-LP------RGSVTS-----LTDRVYGSALYFKGTWEEK--YSKSMTKCKPFYL
FvSRP-12        ----DI-LP------ADVVNS-----KTVLILANALYFKAYWAEK--FSPSNTEKNDFYL
FvSRP-7         ----DI-LP------PDAVNN-----LTKLIFANALYFKAYWVKK--FSKFSTRKRNFYL
FvSRP-11        ----DI-LP------ADAVDS-----NTKLIFANALYFKAYWVEK--FSKSATRKRNFYL
FvSRP-2         ----EI-LP------PGSVHT-----VTGLIYANALYFKATWNDDYFHKPPKSKDLKFYL
FvSRP-1         ----KI-LP------PGSVHS-----ATSLIFANALYFKATWEDDYFYKPRKSKDLKFYL
FvSRP-10        ----EI-LP------PGSVHS-----ATGLIFANALYFKATWNDGYFYKPPKSEDLKFYL
FvSRP-9         ----QI-LL------PGSVNT-----WTILIFANALYFRATWDDM-YFEESETKEHEFHL
FvSRP-14        ----QI-LP------PGSIKI-----ETSLICVNALYFKATWSKP-YFTKPKTKEHEFHL
FvSRP-6         ----DI-LS------PGSVSR-----KTDLILASALYFKGAWNEK--FDESMTKEFDFHL
FvSRP-4         ----DL-LP------YETVTC-----ATRIILANALYFKGAWDQK--FIESRTNMFDFHL
FvSRP-13        ----SI-LP------PDSVST-----ETSLILANALYFKGDWDKE--FDASGTRVHDFHL
FvSRP-5         ----SI-LP------PDSVST-----ATRLILANALYFKGEWDRE--FEASDTRVSDFHL
FvSRP-8         ----SI-LP------FGSVSS-----ATRVILANALYFKGEWDKE--FDASYTRVRNFHL
AtSRP-2         ----QI-LS------RDCTDTIKEIRNSTLILANAVYFKAAWSRK--FDAKLTKDNDFHL
AtSRP-3         ----QI-LS------RDSIDT---IRSSTLVLANAVYFKGAWSSK--FDANMTKKNDFHL
AtSRP-8         ----EI-LS------DDSIKT---IRESMLILANAVYFKGAWSKK--FDAKLTKSYDFHL
SbSRP-16        ----EL-LP------PGSVDE-----STRLVLGNALYFKGAWTNK--FDASETRDGEFHL
OsSRP-6b        ----NI-AT------PRSINH-----NTKLVLANALYFKGAWAEK--FDVSKTEDGEFHL
OsSRP-5         ----EI-LP------PGSVDH-----TTRLVLGNALYFKGAWTEK--FDASKTKDGEFRL
OsSRP-6a        ----EI-LP------PGSVDH-----TTRLVLGNALYFKGAWTEK--FDASKTKDGEFHL
SbSRP-2         ----EL-LP------PGSVDQ-----TTRLVLGNALYFKGAWTEK--FNVSETRDSEFHL
SbSRP-3         ----EI-LP------PGSVDH-----TTRLVLGNALYFKGAWTEK--FDASKTKDSEFHL
AtSRP-5         ----EV-LP------EGSADS-----MTKLIFANALYFKGTWNEK--FDESLTQEGEFHL
FvSRP-3         ----EV-LP------VGSVDA-----STRLVFANALYFKGAWSEK--FEASFTKESDFHL
MtSRP-11        ----DV-LS------CGSIDS-----LTRLIFANALYFKGAWHQP--FDASETKDYDFHL
MtSRP-9         ----DV-LS------PGSIDS-----LTGLIFANALYFNGAWHQP--FDASKTKDHDFHL
MtSRP-6         ----NL-LQ------PGSVHS-----LTSLIFANALYFKGVWKQP--FDTSKTKDYDFDL
MtSRP-16        ----DL-LP------RGSVDS-----LTSLIFANALYFKGVWKRP--FDTSKTKDYDFDL
MtSRP-3         --------------------C-----LTNFIFANALYFKGVWLQE--VDTSKTKDYDFNL
MtSRP-4         ----DL-LP------PDSVDS-----LTNLIFANALYFKGAWSEE--FDTSKTKDYDFNL
MtSRP-10        ----NL-LP------PGSVND-----LTVLIGANALYFKGAWEEQ--FDISYTEDNDFHI
MtSRP-12        ----NL-LP------PGSVDD-----LVVLIGANALYFKGTWEEQ--FDIEDTEDYVFHV
MtSRP-1         -----------------------------------MYFKGIWNEK--FDTSVTKDSDCYL
MtSRP-13        ----QI-----------------------------------------HNISKTKDYDFHL
MtSRP-14        ----EL-LP------LRSVDR-----LTRLIFANALYFKGAWSDK--FDVSKTKDYDFHL
MtSRP-5         ----EL-LP------LGSVDY-----FTQLIFANALYFKGEWNDN--FDASETNVYDFHL
MtSRP-2         ----QL-LP------SRSVNS-----LTRLIIANALYFKGVWNDK--FDASKTKDYDFHL
MtSRP-7         ----EL-LP------LGSVNN-----ATRLIFANALYFKGAWNDK--FDASKTEDYEFHL
MtSRP-15        ----EI-LP------QGSINN-----LSRLVFANALYFKGSWNQK--IDASTTKDYDFYL
SbSRP-12        ----SI-IS------TGDIDA-----NTDLVLANAVYFKGAWLEP--FNPYATNWGTFHR
SbSRP-1         ----DV-LS------PGSVDA-----ETGLMLVNAIYFKGKWVRP--FDERCTEVEDFYL
SbSRP-9         ----SI-LD------PSSVDT-----LTTLVLCSAIYFKGRWEAP--FAKAHTVVDKFHR
OsSRP-10        ----TI-LP------PGSVST-----DTGLVVTSAIYFNGQWWTP--FCKEITEKRAFHR
OsSRP-8         ----TI-LP------PGSVST-----DTGLVVTSAIYFNGTWQTP--FRKQDTKKDKFHL
OsSRP-12        ----TI-LP------PGSVST-----DTRLVVASAIYFNATWQTP--FRKQDTKKDKFHI
SbSRP-15        ----SI-LP------QGSVSS-----DTALVLANAIYFKGKWSVP--FPKKDTEIRRFQR
OsSRP-9         ----EI-LP------DGSVHV-----DTALVLVNAIYFKGKWSNP--FPRSSTTTGKFHR
OsSRP-11        ----EI-LP------DGSVHG-----GTALVLVNAIYFKGKWSNP--FPRERTTTGKFHR
OsSRP-15        ----DV-LP------PRSVGP-----ETAVVLGNAIYFKGKWDRP--FNESDTERKPFYR
SbSRP-10        ----QVFIN------PEDDDN---DDTVHVIANNAIYFKGEWRNP--FKKENTVDREFHR
OsSRP-13        ----EV-IK------PESQSV-----DTRHVVGNAIYFKGEWLAP--FDKSDTAEREFRR
SbSRP-13        ----NL-LS------DGAVHG-----DTKVVLANGMHFKATWARR--FDPSNTVRDNFYR
SbSRP-14        ----NL-LS------DGAVHG-----DTKVVLANGMHFKATWARR--FDPSDTVPHYFYR
OsSRP-1         ----ERHAL------QGEVGS-----DVRVV-----------------EHHAGNVPPPRR
OsSRP-2         ----NT-IS------PGTFGS-----STKCVLANAMHFKATWGRK--FESYDTQRRRFHR
SbSRP-5         ----AV-LP------PGSVGP-----STVAVLANALYFKGSWAQP--FDTSRTFDAPFHL
SbSRP-6         ----DV-LP------PGSVNS-----STVLVLANAIYFKGTWARR--FDRSRTFAAPFHL
OsSRP-4         ----DV-LP------PNSVDS-----STVVVLANAVHFKGTWSLP--FHPSATFHAPFHL


CrSRP-1         --TGAKA-----TKEVPFMCKN-FEAQDRITAVRKE---GL------------YDAVAL-
VcSRP-1         H-SG-QP------KMVPMM----------HQLFD-----GPAKTLKGVRKEGLYDAISL-
```

```
SbSRP-11        L-DG-TC-----VE-VPFVEYD-RTRPF--AVHD-----G------------FKVIKL-
OsSRP-14        L-DG-TS-----VE-VPFV-EY-DRTRL-FAVHD-----N------------FKVIKL-
SmSRP-1         P-SG-KT-----KE-VLMM-RS-SKKHF-IKNHG-----S------------FKVLRL-
PpSRP-1         A-DG-TT-----IQ-VPMM-RS-HKNQF-FKSFP-----T------------HKVVRL-
PpSRP-2         L-DG-KT-----IK-VSMM-HT-SQRQY-VKSFP-----T------------FKALRL-
PpSRP-3         L-DG-KS-----IK-VPMM-HT-TKKQY-VKDFS-----T------------FKALRL-
PpSRP-4         L-DG-KS-----IQ-VPML-HT-TKKQY-VKDFS-----T------------FKVLRL-
SbSRP-8         V-DD-ASRDHAVVR-VPFMSGSFRHRFMQIGVHNAAGGGG------------FKVLRM-
SbSRP-4         S-PD-HA-----VR-APFMTGNHLHTQMRIGCHP-----G------------FNVLRM-
OsSRP-7         S-PD-HD-----VR-VPFMAGSHQHAFMDVGCHP-----G------------FNVLRM-
MtSRP-8         S-DG-SS-----VRNIPFM-VS-DETQY-IKSFDLF---G------------FKTLSL-
SbSRP-7         H-DG-GV-----VR-VPFM-SS-TSKQC-IACRP-----G------------YKVLRL-
OsSRP-3         P-DG-GH-----VS-APFM-SS-GKWQY-IACRA-----G------------YKVLRL-
AtSRP-4         L-DG-TK-----VR-VPFM-TG-ASCRY-THVYE-----G------------FKVINL-
AtSRP-7         V-NG-TS-----VS-VPFM-SS-YENQY-VRAYD-----G------------FKVLRL-
AtSRP-1         L-NG-KS-----VS-VPFM-RS-YEKQF-IEAYD-----G------------FKVLRL-
AtSRP-6         L-NG-TS-----VS-VPFM-SS-FEKQY-IAAYD-----G------------FKVLRL-
FvSRP-12        L-NG-GS-----VEAVPFM-RG-YGCWY-IGVFE-----G------------FKVLKL-
FvSRP-7         L-DG-SS-----VEGVLYM-RG-GDYQY-ICAFE-----G------------FKVLKL-
FvSRP-11        L-DG-SS-----VKGVPFM-RD-GGYQS-ICAFE-----G------------FKVLKL-
FvSRP-2         L-NG-DS-----VKGVPYM-TS-RHEHY-IAVFD-----D------------YKVLSL-
FvSRP-1         L-NG-DS-----VKGVPFM-SS-SHEHS-IAVFD-----D------------FKVLSL-
FvSRP-10        L-NG-ED-----CA---------------LEDFD----------------------NG-
FvSRP-9         L-NG-GS-----VKGVPFM-TS-EDCHS-IGAFD-----G------------FKVLRL-
FvSRP-14        M-NG-DS-----VMKVPFM-TS-NDCHY-IGAFD-----G------------FKVLRLF
FvSRP-6         Q-GG-SS-----VK-APFM-TS-SRYQF-ISVFD-----S------------FKVLKL-
FvSRP-4         L-SG-KS-----VK-APFM-TS-WKDQF-ISVFD-----S------------FKVLKL-
FvSRP-13        LRSD-AS-----VK-APFM-TK-SSDQY-VSEFE-----T------------FKVLML-
FvSRP-5         LHNV-GS-----VK-AHFM-TK-WSDQF-VRAFE-----S------------FKVLKL-
FvSRP-8         L-DNAGS-----VK-AHFMRTK-WLYQY-VSEFE-----T------------FKVLKL-
AtSRP-2         L-DG-NT-----VK-VPFM-MS-YKDQY-LRGYD-----G------------FQVLRL-
AtSRP-3         L-DG-TS-----VK-VPFM-TN-YEDQY-LRSYD-----G------------FKVLRL-
AtSRP-8         L-DG-TM-----VK-VPFM-TN-YKKQY-LEYYD-----G------------FKVLRL-
SbSRP-16        L-NG-TS-----VG-APFM-SS-RDDQY-MASYG-----D------------LKVLRL-
OsSRP-6b        L-DG-ES-----VQ-APFM-ST-RKKQY-LSSYD-----S------------LKVLKL-
OsSRP-5         L-DG-KS-----VL-APFM-ST-SKKQY-LSSYD-----S------------LKVLKL-
OsSRP-6a        L-DG-KS-----VQ-APFM-ST-SKKQY-ILSYD-----N------------LKVLKL-
SbSRP-2         L-DG-TS-----VQ-APFM-SS-GKDQC-IACYD-----D------------FKVLKL-
SbSRP-3         L-DG-SS-----VQ-APFM-SS-TKKQY-IVSYN-----N------------LKVLKL-
AtSRP-5         L-DG-NK-----VT-APFM-TS-KKKQY-VSAYD-----G------------FKVLGL-
FvSRP-3         L-DG-SK-----VQ-APFM-TS-KRKQF-LSAYD-----G------------FKVLGL-
MtSRP-11        L-DG-SS-----FK-VPFK-TS-RESQF-ISVFD-----G------------FKVLRL-
MtSRP-9         L-GG-SS-----IK-VPFM-TS-KKKQF-INAFD-----G------------FKILRL-
MtSRP-6         L-NG-KS-----VK-VPFM-TS-KNDQF-ISSFD-----G------------FKVLGL-
MtSRP-16        L-NG-KS-----VK-VPFM-TS-KNNQF-ISSFD-----G------------FKVLGL-
MtSRP-3         L-NG-SS-----VK-VPFM----------------------------------TILRL-
MtSRP-4         L-NG-SQ-----VK-VPFM-TS-MKRQF-ISVFD-----G------------FKVLRL-
MtSRP-10        Q-NC-NS-----VK-VPFM-TS-CEEQF-IGVFD-----D------------FKILRL-
MtSRP-12        Q-NG-NS-----------------------------------------------ILHL-
MtSRP-1         L-NG-SS-----VK-VPFM-TS-YKKRY-IGDFD-----G------------YKVLYL-
MtSRP-13        L-NG-SS-----VK-VPFM-TS-NKKQF-IEVYD-----D------------FKVLHL-
MtSRP-14        L-NG-----------------------F-IEAYD-----D------------FKVLRL-
MtSRP-5         L-NG-NS-----VK-VDFM-TSDEKKQF-IRDFD-----D------------FKVLGL-
MtSRP-2         L-NG-SS-----IK-VPFM-TS-KEEQY-IRAFD-----D------------FKVLGL-
MtSRP-7         L-NG-SP-----VK-VPFM-TS-KKKQF-IRAFD-----G------------FKVLGL-
MtSRP-15        L-NG-SS-----VK-VSFM-TS-KKKQF-IRAFD-----G------------FKVLGL-
SbSRP-12        L-DG-GL-----AE-AEFMRSGIWSLD--VACMD-----G------------FKVLKL-
SbSRP-1         L-DG-TA-----VQ-TPLM-RG--RGSYLVAVHG-----G------------FKVLKL-
SbSRP-9         L-DG-ST-----AD-VPFM-SS-VRSQY-IAIRK-----G------------YKLLKL-
OsSRP-10        L-DG-GD-----VE-ADFM-RS-GEDQY-IAVHD-----G------------FKVLKM-
OsSRP-8         L-DGHGT-----VD-ADFM-RT-GEDQY-IAAHD-----G------------FKVLKM-
OsSRP-12        LGGG-GD-----VD-ADFM-RS-GDDQY-VAAYD-----G------------FKVLKM-
SbSRP-15        L-DG-SH-----VL-TPFM-RS-RKDHA-VAVFD-----G------------FKVLKL-
OsSRP-9         L-DG-SS-----VD-VPFM-SS-REDQY-IGFHD-----G------------FTVLKL-
OsSRP-11        L-DG-SS-----VD-APFM-SS-REDQY-IGFYD-----G------------FKVLKL-
OsSRP-15        H-DG-AAAAAAVAD-VPYM-SS-RSYQR-VAVHD-----G------------FKVLKL-
SbSRP-10        L-DG-SS-----VE-VPFL-QS-WSYQC-IACHS-----G------------FKVLKL-
OsSRP-13        L-DG-SS-----VE-VPFMQRPSGSYHH-VACHD-----G------------FRVLRL-
SbSRP-13        R-NG-EP-----VR-VPFL-SD-AGMHY-AESFDAPGL-G------------FKVLQC-
SbSRP-14        R-DG-EP-----VW-VPFL-SD-AGMHY-AESFDAPGL-E------------FKVLRC-
OsSRP-1         H-DGAGA-----VPVGPRDALRRQGAKFEFHGLE------------------FKVLQL-
OsSRP-2         Q-DG-TR-----VT-VPFL-SD-PRTHY-AARFDGL---G------------FKVLQL-
SbSRP-5         P-GG-AT-----VR-APFMTTSSRFEQHYVAVFP-----G------------FRALKL-
SbSRP-6         P-GG-AT-----VR-APFMTTSPLSEDQQVAVFP-----G------------FKALKL-
OsSRP-4         L-DG-GA-----VR-APFMTTEIPFERH-VAAFP-----G------------FTALKL-


CrSRP-1         --PYKGD----------------------------------------AFSAI--ALLPA
VcSRP-1         --PYK----------------------------------------GDVFSAV--ALLPA
SbSRP-11        --PYQQG-----K--------------------------------NERKFSMY--IFLPD
OsSRP-14        --PYKQG-----K--------------------------------NERKFSMY--IFLPD
SmSRP-1         --PYQRG-----D--------------------------------DPRSFSMY--VLLPA
PpSRP-1         --PYALK-----ET-------------------------------TQRSFAMF--ILLPQ
```

```
PpSRP-2         --PYSAG-----H--------------------------------DERLFSMF--ILLPS
PpSRP-3         --PYSSG-----H--------------------------------DRRSFSMF--ILLPH
PpSRP-4         --PYSAG-----S--------------------------------DRRSFSMF--ILLPH
SbSRP-8         --PYRGD-----GG-------------------------------GGTEFAMY--IYLPD
SbSRP-4         --SY-----------------------------------------TDRNFAMY--IYLPD
OsSRP-7         --MYRTG-----GAAG-----------------------------GDKMFAMY--IYLPD
MtSRP-8         --AYKKG-----NG-------------------------------DDKHYQFYLDIFLPN
SbSRP-7         --RYAQH-----GGG------------------------------EQRLLSMY--IYLPD
OsSRP-3         --PYARGGRGRGRD-------------------------------TGRLFSMY--IYLPD
AtSRP-4         --QYRRG-----RE-------------------------------DSRSFSMQ--IYLPD
AtSRP-7         --PYQRG-----SDD------------------------------TNRKFSMY--FYLPD
AtSRP-1         --PYRQG-----RDD------------------------------TNREFSMY--LYLPD
AtSRP-6         --PYRQG-----RDN------------------------------TNRNFAMY--IYLPD
FvSRP-12        --AYRNQ-----GV-------------------------------HPRCFSMY--FYLPD
FvSRP-7         --PYRNL-----GV-------------------------------SPWSFFMY--FCLPD
FvSRP-11        --PYRNL-----GV-------------------------------SPRCFSMY--FCLPD
FvSRP-2         --RYRKC-----ALEDFDNGTYKF---------------------DPRSFSML--WLLPD
FvSRP-1         --QYSNC-----ALEDSDNGTDKY---------------------DPRSFSML--WLLPD
FvSRP-10        --TYKH---------------------------------------DPRSFSML--WLLPD
FvSRP-9         --PFNNY-----DYDDLQIGLVNYTTPGRIQTSKDWSHRGTRKYDDPRQFSMF--MLLPD
FvSRP-14        SRKYEDGELFEDLSHLSRTGNRKYD--------------------DYRKFSIF--WLLPD
FvSRP-6         --PYKHG-----ED-------------------------------YDRRFCMC--VFLPN
FvSRP-4         --PYKQG-----ED-------------------------------HNRRFSMY--MFLPN
FvSRP-13        --PYKQG-----KD-------------------------------YERRFSMY--IFLPN
FvSRP-5         --LYKQG-----QD-------------------------------YEREFSMY--IFLPN
FvSRP-8         --PYKQG-----KD-------------------------------YEREFSMY--IFLPN
AtSRP-2         --PYVE---------------------------------------DKRHFSMY--IYLPN
AtSRP-3         --PYIE---------------------------------------DQRQFSMY--IYLPN
AtSRP-8         --PYVE---------------------------------------DQRQFAMY--IYLPN
SbSRP-16        --PYEQG-----G--------------------------------DKRQFSMY--ILLPE
OsSRP-6b        --PYLQG-----G--------------------------------DKRQFSMY--ILLPE
OsSRP-5         --PYQKG-----R--------------------------------DLRQFSMY--ILLPE
OsSRP-6a        --PYQQG-----G--------------------------------DKRQFSMY--ILLPE
SbSRP-2         --AYQQG-----G--------------------------------DKRKFSMY--ILLPE
SbSRP-3         --PYQQG-----G--------------------------------DKRQFSMY--ILLPD
AtSRP-5         --PYLQG-----Q--------------------------------DKRQFSMY--FYLPD
FvSRP-3         --PYRQG-----E--------------------------------DKRRFSMH--IYLPD
MtSRP-11        --PYEHG-----TI-------------------------------DDRHFSMY--FLLPD
MtSRP-9         --PYKQG-----N--------------------------------DIRQFSMY--FFLPD
MtSRP-6         --PYKQG-----N--------------------------------YGRAFSIY--FFLPD
MtSRP-16        --PYKQG-----N--------------------------------DKRAFSIY--FFLPD
MtSRP-3         --FYKHD-----AFRQS----------------------------DKRYFSMY--FFLPD
MtSRP-4         --FYKHEYSYKQS--------------------------------DKRYFSIY--FFLPD
MtSRP-10        --PFEQG-----G--------------------------------GKRRFSFY--LFLPD
MtSRP-12        --PFEQG-----G--------------------------------GKRRFSFY--LFLPD
MtSRP-1         --PYNQG-----K--------------------------------DECQFSMY--IFLPD
MtSRP-13        --PYKKG-----E--------------------------------DKRQFSMY--FFLPN
MtSRP-14        --PYKKG-----K--------------------------------DKRPFSMY--IFLPN
MtSRP-5         --PYKQG-----E--------------------------------DKRQFTMY--FFLPN
MtSRP-2         --PYKQG-----E--------------------------------DKREFTMY--FFLPN
MtSRP-7         --PYKQG-----E--------------------------------DKRQFTMY--FFLPN
MtSRP-15        --PYKQG-----E--------------------------------DKRRFTMY--FFLPN
SbSRP-12        --PYERGAAGTGSCQLKRRRGQQVAGKSKATAPSTEAN-------EGTQYSML--FFLPD
SbSRP-1         --PYQAPLAFPRFGGGMRMAKVARRGG------------------VGAMYSLC--VFLPD
SbSRP-9         --PYRSPAAPAPAPRRKGATPSESNRPTGDGGDDDDDD-------PAPKYSMC--IFLPD
OsSRP-10        --PYAACVSARTT--------------------------------TTPRYSMY--VFLPD
OsSRP-8         --PYAHDHAAPQP--------------------------------SPRYYSMY--ILLPD
OsSRP-12        --PYNTR-----ASRTH----------------------------TQPQYSLC--VFLPD
SbSRP-15        --AYETHRRKADRHLSGRNSKQQDGHNSD----------------EHPRFSMC--VFLPD
OsSRP-9         --PYHHRTMKNHGDGGDTITNSSITRAILEHYGG-----------ENVGLSMY--IFLPD
OsSRP-11        --PYHRT-----MKNHGDGGDITPAILKHYG--------------ENVGLSMY--IFLPD
OsSRP-15        --RYRSPRLLRDKRKRGGGGDVGG---------------------EFTRYAMA--IFLPD
SbSRP-10        --PYELMNESNWKLYD-----------------------------SLPRFSMC--VFLLD
OsSRP-13        --PYKAT-----SDTYNLKLRY-----------------------SLPSFAML--VFLPD
SbSRP-13        --FYKMV-----GRDGKLDP-------------------------KAPCFCML--IFLPH
SbSRP-14        --FYKMV-----GRDGRLDS-------------------------RAPCFCML--IFLPH
OsSRP-1         --FYKMV-----GRDGQVDFGF-----------------------GAPCFCML--VFLPI
OsSRP-2         --FYKMV-----GHDGQVHF-------------------------GAPCFCML--VFLPI
SbSRP-5         --PYSCKSGDQWH--------------------------------QAAYFYML--LLLPD
SbSRP-6         --PYKND-----G--------------------------------GAAWFYML--LLLPD
OsSRP-4         --PYKNV-----GGGGGGDGV------------------------PRAAFYML--LLLPD
                   :                                                     *

CrSRP-1         VGVA---MEAALKDWGS------GP----------------Q-ELRPVGKCFVKLPKFKV
VcSRP-1         VGVD---MATAFEDWAR------GPQEF------QP-----------IRRVKIVMPKFKV
SbSRP-11        AHDG---LFELTKKIFA------EP-SFLEHH--LP-------TEKRHVDIRV--PKFTV
OsSRP-14        DHDG---LFELTQKIFS------EP-MFLEQH--LP-----T-EKCHVG-ISV--PNFKI
SmSRP-1         ELQG---LPALVSSLH-------DA-ESMGNA--LA-----GIHEVEVGDFQL--PKFKI
PpSRP-1         ENIT---LLDLESSL--------TPQSLTSDL--TN-----FGNKVPLKRFLL--PKFKV
PpSRP-2         EKYG---LVELEKALDA------KTLAEDLQH--VK-------QLLPVSKFEL--PKFKI
PpSRP-3         EKNG---ITEFEKSL-D------FK-TLAEDLSKVN-------QEAPMNQFAL--PKFKI
PpSRP-4         EKDG---ITELEKSLDL---------KILAEDLRTV-----N-QEVPVTEFAL--PKFKI
SbSRP-8         DRDG---LPALLRALSA------SP-DTLLGRSVVP-----EEPAVKVGELKI--PKFEV
```

```
SbSRP-4         ELDG---LPGLVRQIGS------DPAAFLRKT-IVP-----E-KPVTVGKLRI--PRFLV
OsSRP-7         DRDG---LPELARKLAS------NPAAFLRRT-IVP-----A-QPVAVGELKI--PKFEV
MtSRP-8         PGFD---LPTMLQSMAA------NNFDLIMSR--LV-----Q-DKVRVGEFRI--PKFKI
SbSRP-7         AHDG---LPALLHKLSA------DPAAPLESSRTLT-------SEVPVRAFRV--PRFTV
OsSRP-3         ERHG---LPDMLRKLCS------DPAALIESSAALT-------EKVPVGAFMV--PRFTL
AtSRP-4         EKDG---LPSMLERLAS------TR-GFLKDNEVLP-----S-HSAVIKELKI--PRFKF
AtSRP-7         KKDG---LDDLLEKMAS------TP-GFLDSH--IP-----T-YRDELEKFRI--PKFKI
AtSRP-1         KKGE---LDNLLERITS------NP-GFLDSH--IP-----E-YRVDVGDFRI--PKFKI
AtSRP-6         KKGE---LDDLLERMTS------TP-GFLDSH--NP-----E-RRVKVGKFRI--PKFKI
FvSRP-12        ARDG---LPALVEKVCS------GS-GFLDSH--VP-----H-SKLRVGKMLI--PKFKI
FvSRP-7         ARDG---LPALVEQVCS------GS-RFLEGC--VP-----D-EELYVGKMLV--PKFKI
FvSRP-11        ARDG---LPALVEDFCS------GS-EFLEGC--IP-----D-EELYVGEMLI--PKFKI
FvSRP-2         ARDG---LPALAERVCS------KP-GFFDRH--LN--CESF-TSVKVKKFLI--PKFKI
FvSRP-1         ARDG---LPALAERVCS------ES-RFFDRH--LN--FESF-TSVKVKKFLI--PKFKM
FvSRP-10        ARDG---LPALAERVCS------ES-GFLDRH--LNLKFESF-TWVRVKKFLI--PKFKI
FvSRP-9         ARDG---LPALVERVCS------ES-EFLDRH--TR-----T-RSARVGKFFI--PKFKM
FvSRP-14        ARDG---LPALVERVCT------ES-GFLDRH--TD-----L-RYVRVGEFLI--PKFKM
FvSRP-6         ETDG---LQALVERVCS------ES---IDGY--IP-----R-TSVPVGKFLI--PKFKI
FvSRP-4         ASNG---LPSLVERFCS------EA-GFLDRY--LP-----C-RKVEVDKFLI--PKFKI
FvSRP-13        ARKG---LRALVKKFCS------DF-EFMDRH--LP-----D-ERVPVDEFLI--PKFKI
FvSRP-5         TRNG---LPSLIEQFCS------NV-EFIDSH--LP-----N-ERVPVRRFLI--PKFKI
FvSRP-8         ARNG---LRALIEQFCS------NV-KFIDSH--LP-----D-HKVPVGRFLI--PKFKI
AtSRP-2         DKDG---LAALLEKIST------EP-GFLDSH--IP-----L-HRTPVDALRI--PKLNF
AtSRP-3         DKEG---LAPLLEKIGS------EP-SFFDNH--IP-----L-HCISVGAFRI--PKFKF
AtSRP-8         DRDG---LPTLLEEISS------KP-RFLDNH--IP-----R-QRILTEAFKI--PKFKF
SbSRP-16        AQDG---LWSLAEKLSS------EP-EFLDRH--IP-----T-RKIPVGQIKV--PKFKI
OsSRP-6b        AQDG---LWSLAEKLNS------EP-EFMENH--IP-----M-RPVHVGQFKL--PKFKI
OsSRP-5         AQDG---LWSLAAKLNS------EP-EFLEKR--IP-----T-RQVTVGKFKL--PKFKI
OsSRP-6a        AQDG---LWSLAEKLNS------EP-EFLEKH--IP-----T-RQVTVGQFKL--PKFKI
SbSRP-2         ARDG---LWSLAEKLSS------EP-EFMEEH--IP-----T-RKVPVGQFKV--PKFKV
SbSRP-3         AKDG---IWSLSEKLSS------EP-EFLDKY--IP-----M-QKVPVGQFKV--PKFKI
AtSRP-5         ANNG---LSDLLDKIVS------TP-GFLDNH--IP-----R-RQVKVREFKI--PKFKF
FvSRP-3         AKDG---LPALVEKVGT------GS-GFLQRH--LP-----Y-EQVSVSDFKL--PKFKI
MtSRP-11        AKDG---LSALIEKVAS------EY-ETLEHI--LP-----D-SIVDVGDFRI--PSFEI
MtSRP-9         AQDG---LLALIEKVAS------KP-EILKHK--LP-----R-KEVIVGDFRI--PRFKI
MtSRP-6         AKDG---LSALIDNVTS------DY-EFLEHN--LP-----R-RKVEVGKFRI--PRFNI
MtSRP-16        EKDG---LSALIDKVAS------DS-EFLEQK--LP-----R-NQVKVGKFMI--PRFNI
MtSRP-3         AKDG---LLALTEKVAS------ES-EFLERT--CP-----K-QTVRVGDFRI--PRFKI
MtSRP-4         AKDG---LLALTEKVAS------ES-EFLEHT--RP-----R-ETVIVGDLRI--PKFNI
MtSRP-10        AEDG---LLDLIEKLAS------EF-EFLQHK--LP-----S-RKVKVGTFRI--PRLNI
MtSRP-12        AEDG---LLDLIEKLAS------EF-EYLQHK--LP-----S-RKVKVGAFRI--PRFNI
MtSRP-1         AKDG---LSTLVEKLAS------EF-ELPEHN--LP-----LIKKVAVGEFKI--PRFNI
MtSRP-13        AKDG---LSALVEKVSS------TS-EFLHRS--LC-----L-SQKELGNFKI--PKFNI
MtSRP-14        AKDG---LSTLVKKVAS------ES-ELLHHRFHLP--------EEEVGEFRI--PRFKI
MtSRP-5         AKDG---LPTLLEKLAS------ES-ELLQHK--LP-----L-DKVEVGDFRI--PRFNI
MtSRP-2         AKDG---LPTLLEKLAS------ES-ESLKHK--LP-----Y-DRVEVGDFRI--PRFNI
MtSRP-7         AKDG---LAALVEKVAS------ES-ELLQHK--LP-----F-GKVEVGDFRI--PKFNI
MtSRP-15        AKDG---LPSLVEKVAS------ES-DLLQHK--LP-----F-DKVEVGDFRI--PKFNI
SbSRP-12        ARDG---LSTMVDLVTA------SP-SFLYGI--LA-----EMKERPV-VMEL--PKFAI
SbSRP-1         ARDG---LWSLVDELAA------SGPAFLHDH--LP-----W-SKVCVHKLRL--PRFKM
SbSRP-9         ERDG---LPGLVEKIAS------GA-GFWHYR--LP-----T-SQVPVGAFRL--PKFKV
OsSRP-10        ERDG---LWSLEDRMAA------GGEGFLREH--TP-----E-RRVEVGEFRI--PRFKL
OsSRP-8         ERDG---LSSLEDRMAAAGGGGGGE-GFLSEH--MP-----V-RRVEVGEFRI--PRFKL
OsSRP-12        KRNG---LWTLADRMEA-----GGGEVFLREH--MP-----E-KRVKVGEFRI--PRFKL
SbSRP-15        AHDG---LQNLMDMVAS------HP-SFLWDH--MP-----R-RRVKVGELRL--PKFKL
OsSRP-9         ERDG---LPALVDKMAA----SSSSSSFLRDH--RP-----T-RRREVGDLRV--PRFKV
OsSRP-11        ARDG---LPALVDKMAVASSGTASS-SFLRDH--RP-----GRRRIKVGDLRV--PRFKV
OsSRP-15        ARDG---LRGLVERMAS------RP-GFLHEH--MP-----AAWPVPVGEFRV--PKFKV
SbSRP-10        GKKG---LRDIMEKIAS------SLPAFLHDH--LP-----K-EYVPIGQFRL--PKFKL
OsSRP-13        DRDG---LPGLLDRITA------SP-EFVDEH--LP-----P-GCVPVGRFRV--PKFKL
SbSRP-13        RDDG---LRDLLRLAVT------EP-DFVMRC--AP-----R-REQAVCPCKV--PKFKF
SbSRP-14        SDDG---LLDLLRLAVT------EP-DFVMRC--AP-----R-REQEVCPCKV--PKFKF
OsSRP-1         KRDG---LRHLLRMAVT------EP-DFVTRC--VP-----R-SRQIVTPCKV--PKFKF
OsSRP-2         KRDG---LRHLLRMAVT------EP-DFVMRC--VP-----R-SEQEVSPCMV--PKFKF
SbSRP-5         DDHG---LGDVYDKAVS------TP-GFIRKH--TP-----V-GKVPVGRLMV--PKFKF
SbSRP-6         GEALT--LSDLYDKAVS------TP-GFIRRH--TP-----V-DGVPVRRFMV--PKFKF
OsSRP-4         G-DGALKLADLYDMAVT------TP-EFIKKH--TP-----A-AEAPVRRLMV--PKFKF
                       :                                           :  * : .

CrSRP-1         ES-QLSLK-TVLH-KLGVKQAFGGS-----AD-FSRL---S--DT---------------
VcSRP-1         SS-QLSLK-PVLK-EMGVEVAFTVA-----AD-FTRM---A--DG---------------
SbSRP-11        SF-QVDMK-EFLK-EMGLELPFLRD-----AD-FTDM---VKEDES-----------RS-
OsSRP-14        SF-QIDVK-DFLK-DMGLELPFLRE-----AE-FSDM---IKEDDS-----------SG-
SmSRP-1         SL-GVQAP-ELLK-RMGLDLAFSPPH----AD-FSGM---V--EGS-------P---GD-
PpSRP-1         TY-GLEVS-EPLK-AMGMELPFTPE-----GD-FSDM---TSDDG---------------
PpSRP-2         SS-GFEVP-KALE-SMGLTLPFGRE-----AD-LTEM---L--DSPV----------SD-
PpSRP-3         SF-SFEVP-EALQ-TLGLSLPFGEE-----AD-LTEM---V--DSSL----------AD-
PpSRP-4         SH-GFEAP-EALK-SLGLSLPFGEE-----AD-LTDM---V--DSPT----------AD-
SbSRP-8         SL-RVEAS-QMLQ-SLGLDLRSS-------GDSFSEM---LSPPAP--------------
SbSRP-4         SL-KVEAS-RLLR-DLGLDLPFDPAM----AD-FSAM---L--MPDS----------PQ-
OsSRP-7         SL-KVEAS-RLLR-EFGLDLPFLPAA----DNSFSGM---L--LDP-----------PQ-
MtSRP-8         LS-RLDDTLDVLK-ERGVSKAFEK------GA-LKDI---L--QHDA------A---GN-
SbSRP-7         AY-RTNAR-EMLL-DLGLLLPFDRVA----AD-FGDM---G--EEAG------A---PE-
```

```
OsSRP-3         SY-KTNAA-ETLR-QLGLRLPFEYPG----AD-LSEM---V--ESSP------E---AE-
AtSRP-4         DF-AFEAS-EALK-GFGLVVPL--------------------------------------
AtSRP-7         EF-GFSVT-SVLD-RLG----------------LRSM-----------------------
AtSRP-1         EF-GFEAS-SVFN-DFELNVSLH-------------------------------------
AtSRP-6         EF-GFEAS-SAFS-DFELDVSF--------------------------------------
FvSRP-12        SS-GFEAS-KVLA-GLGLVLPFVYDV--KGGN-VTEM---V--ESP-------A---GE-
FvSRP-7         SS-GFEAS-KVLK-ASGLVLPFSFDPVNKGGN-VSEM---V--ESP-------S---GD-
FvSRP-11        SS-GFEAS-KVLK-TSGLVLPFIFDPV-KGGN-VTEM---V--ESP-------A---GE-
FvSRP-2         SS-RFEAA-GVLQ-KLGL------------GG-ACDS---M--V----------------
FvSRP-1         SS-RFEAS-SVLQ-KLGL------------GD-GCDS-----------------------
FvSRP-10        SS-GFEAS-DVLK-KIGM------------GD-GCDS---M--A----------------
FvSRP-9         SS-RFEAS-GVLK-QMGLRCVLDS------------------------------------
FvSRP-14        SS-RFEAS-GVLK-KMGL------------GD-VLDL---I--E----------------
FvSRP-6         SA-EFDPL-PVLK-PLGFSFEN--------GH-LTEM---V--EG---------------
FvSRP-4         TF-GIEVS-QVLE-TLGLKLPY-----------LSET---VVGDEP--------------
FvSRP-13        SC-DFEAS-KLLE-TLGFTP----------GG-LTEM---V--DS------------AE-
FvSRP-5         SC-GFEAS-KLLE-TLGFSP----------GG-LTEM---V--DSP------------E-
FvSRP-8         SC-GFEAS-KLLQ-TLGFSL----------GG-LTEM---V--DPP------------E-
AtSRP-2         SF-EFKAS-EVLK-DMGLTSPFTSK-----GN-LTEM---V--DSPS------N---GD-
AtSRP-3         SF-EFNAS-EVLK-DMGLTSPFNNG-----GG-LTEM---V--DSPS------N---GD-
AtSRP-8         SF-EFKAS-DVLK-EMGLTLPFTH------GS-LTEM---V--ESPSIPENLCV---AE-
SbSRP-16        SF-GFEAS-ELLK-GLGIQLPFSSE-----AD-LSEF---F--DSP-------V---PL-
OsSRP-6b        SF-GFGAS-GLLK-GLGLPLLFGSE-----VD-LIEM---V--DSP-------G---AQ-
OsSRP-5         SF-GFEAS-DLLK-ILGLQLPFSSK-----AD-LTGM---V--GSP-------E---RH-
OsSRP-6a        SF-GFEAS-DLLK-SLGLHLPFSSE-----AD-LTEM---V--DSP-------E---GK-
SbSRP-2         SF-GFEAS-DLLK-GLGLELPFSSQ-----AD-LSEL---V--HLP-------A---GQ-
SbSRP-3         SF-GFEAS-KLLK-GLGLQLPFSAQ-----AD-LSEL---V--DSP-------E---GQ-
AtSRP-5         SF-GFDAS-NVLK-GLGLTSPFSGE-----EG-LTEM---V--ESPE------M---GK-
FvSRP-3         SF-GFEAT-NLLK-GLGVVLPFSGE-----GG-LTEM---V--DST-------V---GQ-
MtSRP-11        SF-GFELS-NMLK-ELGVILPFSN------GG-LTKI---V--DSP--------------
MtSRP-9         YS-GLELS-NVSK-ELGVVLPFSG------GG-LTKM---A--DSP--------------
MtSRP-6         SF-EIEAP-ELLK-KLGLTLPFSM------GG-LTKM---V--DSP-------I---SQ-
MtSRP-16        SF-EIEAS-ELLN-KLGLTLPFSK------GG-LTKM---V--DSP-------I---SQ-
MtSRP-3         SF-ELETS-NMLK-ELGMVLPFSQ------GG-LTKM---V--DSP-------T---SQ-
MtSRP-4         AF-GLETS-GVLK-ELGVVLPFST------GG-LTNM---V--DSP-------M---DQ-
MtSRP-10        SF-ELETS-SMLK-ELGVVLPFSDI-----GG-VAKM---V--D-------------NE-
MtSRP-12        SF-ELETS-SVLK-ELGVVLPFSDI-----GG-VAKT---V--A-------------GE-
MtSRP-1         SF-GIETT-NTMK-ELGVILPFSA------GG-FTKI---V--DSSF------E---GE-
MtSRP-13        SF-ELEAT-RMLK-KLGVVLPFSP------GG-FTKM---V--DSSL------M---GK-
MtSRP-14        SF-ELETS-DMLK-ELGVTLPFTR------EG-LTKM---V--DSSL------V---GD-
MtSRP-5         SF-ELETS-DMLK-ELGVVLPFTN------GG-LTKM---V--NSA-------V---SQ-
MtSRP-2         SF-GLETS-DMLK-ELGVVLPFTN------GG-LTKM---V--NS------------SQ-
MtSRP-7         SF-GLETS-DMLK-ELGVVLPFSG------GG-LTKM---V--NSS-------V---SQ-
MtSRP-15        SF-GLETS-DMLK-ELGVVLPFSG------EG-LTKM---V--DS------------NQ-
SbSRP-12        TF-SWGDLKGDLR-RLGLSLPFSPEA----AD-LRGM---CKGDDEV------VDGAGAA
SbSRP-1         SF-HSDLT-DALR-EMGLEATLDPRDGD--TD-LTDM---A--ERK-------GYA-GE-
SbSRP-9         SA-SGSVR-EVLRDGMGIKSAFVAGE----AD-LADMAAKR--DDDE----------DAA
OsSRP-10        SF-DDSVV-GALQ-RLGVRDVFKPFV----AD-LADV---LEAENS-----------GDD
OsSRP-8         SF-SRSVV-RALR-GVGVNAVFDR------AE-LPDM---I--E-------------GE-
OsSRP-12        SF-DGSIK-TALQ-GVGVRAVFDPAA----AD-LSDV---L--EEG-----------NSG
SbSRP-15        SF-SSRIN-GVLE-DMGIKAAFGT------AD-LSEM---L----------------EQR
OsSRP-9         SF-YSQIN-GVLQ-GMGVTAAFDAGE----AD-LSGMAEGV--DQR-----------GG-
OsSRP-11        SF-YSEMN-EVLK-GMGIGAAFDVGK----VD-LSGM---I--DGE--------------
OsSRP-15        SC-GGSVV-GALE-QLGLRLPFSPEL----AD-LSDM---VEDDGS-----------GW-
SbSRP-10        SF-EREIQ-DDLI-HLGLELPFDKKK----AN-MGDLLHEE--D-------------TR-
OsSRP-13        AFCHYGIA-DVLR-GLGLRLPFDMFA----AE-MSGIAVEG--DGE-----------DA-
SbSRP-13        SF-TFDAV-TALW-QLGLSAPFAD------GADLSRM---VS-NMP-----------RE-
SbSRP-14        SF-AFDAG-NALR-QLGLSEPFTYA-----AD-LSGM---V--SSMP----------AE-
OsSRP-1         SS-QLDAG-GALA-QLGLGAPFDPDA----AD-LSRMA--V--NTP-----------PA-
OsSRP-2         SS-ELDAR-GALA-KLGLGAPFDPLA----AD-LSRMAVSV--NTP-----------PE-
SbSRP-5         TF-DFEAR-EEMQ-RLGVIRAFGG------GD-FSGM---F--AGG-----------GG-
SbSRP-6         TF-EFEAS-GDIQ-KLGVMRAFEG------GD-FSGM---V--SG------------GN-
OsSRP-4         SF-KFEAK-SDMR-KLGVTRAFAG------GD-FSGM---V--TG------------GD-


CrSRP-1         --KMFVSDVVHKA-VVEVDE----------------------------------------
VcSRP-1         --GLFISDVVHKA-VVEVDE----------------------------------------
SbSRP-11        --PLYLSDILHKA-ILEVND----------------------------------------
OsSRP-14        --PLFLSDVLHKA-VLEVDQ----------------------------------------
SmSRP-1         --DLFISDVFHKA-FVEVNE----------------------------------------
PpSRP-1         --PLGISSVRHKA-FVDVNE----------------------------------------
PpSRP-2         --KLYVSNIYHKT-FVEVNE----------------------------------------
PpSRP-3         --KLFVSNLYHKT-FVDVNE----------------------------------------
PpSRP-4         --KLYVSNLYHKT-FVEVNE----------------------------------------
SbSRP-8         --PVGLSSVVHQC-VVKVTE----------------------------------------
SbSRP-4         --QVAVSAMLHQC-FVSVNE----------------------------------------
OsSRP-7         --GTAVSSLLHQC-FVNVNE----------------------------------------
MtSRP-8         --KLLVSNIFHKS-FIEVNE----------------------------------------
SbSRP-7         --PLVVSDVYHES-FVEVNE----------------------------------------
OsSRP-3         --KIVVSAVYHES-FVEVNE----------------------------------------
AtSRP-4         ------SMIMHKS-CIEVDE----------------------------------------
AtSRP-7         -------SMYHKA-CVEIDE----------------------------------------
AtSRP-1         ----------QKA-LIEIDE----------------------------------------
```

```
AtSRP-6         ---------YQKT-LIEIDE----------------------------------------
FvSRP-12        --DPYVKAIFHKS-CIEIDE----------------------------------------
FvSRP-7         --DPYVSAMFHKS-CIEIDE----------------------------------------
FvSRP-11        --DPYVSAMFHKS-CIEIDE----------------------------------------
FvSRP-2         --------IFHES-VIEVDE----------------------------------------
FvSRP-1         --------IFHEC-VVEVDE----------------------------------------
FvSRP-10        --------IFHES-VIDVDE----------------------------------------
FvSRP-9         ------REIFHES-VIEVDE----------------------------------------
FvSRP-14        --------IVHESAVIEVDE----------------------------------------
FvSRP-6         --RAVALNMFQKS-FIEVNE----------------------------------------
FvSRP-4         ----FVNLIIHKS-FIEVNE----------------------------------------
FvSRP-13        --PLYASNIFHKA-FIEVNE----------------------------------------
FvSRP-5         --PLYVSQIFHKA-FIEVNE----------------------------------------
FvSRP-8         --PLHVSLIFHKA-FTEVNE----------------------------------------
AtSRP-2         --KLHVSSIIHKA-CIEVDE----------------------------------------
AtSRP-3         --DLYVSSILHKA-CIEVDE----------------------------------------
AtSRP-8         --NLFVSNVFHKA-CIEVDE----------------------------------------
SbSRP-16        --GLSVSSILHRS-FVEVNE----------------------------------------
OsSRP-6b        --NLFVSSVFHKS-FIEVNE----------------------------------------
OsSRP-5         --NLFVSSLFHKS-FVQVDE----------------------------------------
OsSRP-6a        --NLFVSSVFHKS-FVEVNE----------------------------------------
SbSRP-2         --NLCVSSIFHKS-FVEVNE----------------------------------------
SbSRP-3         --NLSVSSVYHKS-FVEVNE----------------------------------------
AtSRP-5         --NLCVSNIFHKA-CIEVNE----------------------------------------
FvSRP-3         --NLYVSSIYHKS-FIEVNE----------------------------------------
MtSRP-11        ---LWISNITQKS-IIKVNE----------------------------------------
MtSRP-9         ---IWVSNIFQNS-FIEVNE----------------------------------------
MtSRP-6         --EIFVSDILQKS-CIEVNE----------------------------------------
MtSRP-16        --ELSVTSIFQKS-FIELNEEGTIAAATARGSTGGAAPFRLPPPPPIDFVADHPFLFLIR
MtSRP-3         --NLYVSDVFHKS-FIEVNE----------------------------------------
MtSRP-4         --NLHISNIFHKS-FIEVNE----------------------------------------
MtSRP-10        --SLVVSKIFHKS-FIEVNE----------------------------------------
MtSRP-12        --SLVVSKIFHKS-FIEVNE----------------------------------------
MtSRP-1         --NLYVSNIFHKS-FIEVNE----------------------------------------
MtSRP-13        --ILSVSNIFHKS-FIEVNE----------------------------------------
MtSRP-14        --SLSVSKIFHKS-FIEVNE----------------------------------------
MtSRP-5         --NLYVSKIFHKS-FIEVNE----------------------------------------
MtSRP-2         --NLCISKIFHKS-FIDVNE----------------------------------------
MtSRP-7         --NLCVSNIFHKS-FIEVNE----------------------------------------
MtSRP-15        --KLFMSNILHKY-FIEVNE----------------------------------------
SbSRP-12        RRPTFLSKVAHTA-VVKVNE----------------------------------------
SbSRP-1         --SPKIDKVCHKA-VIELNE----------------------------------------
SbSRP-9         RTPLCVSDVCHKA-VLEVNE----------------------------------------
OsSRP-10        P-PLFVSDVKHKA-VIEVNE----------------------------------------
OsSRP-8         --PLRVSDVLHKA-VIEVNE----------------------------------------
OsSRP-12        DPPLFVSDVLHGA-AIEVNE----------------------------------------
SbSRP-15        ENGLVLEHVFHKA-VIEVNE----------------------------------------
OsSRP-9         --GLVVEEVFHRA-VVEVNE----------------------------------------
OsSRP-11        --LVVVEKVMHRA-VVEVNE----------------------------------------
OsSRP-15        --PLFVGDIQHKA-VIEVNE----------------------------------------
SbSRP-10        --RMRVNRVIHKA-VIEMNE----------------------------------------
OsSRP-13        --AMFVSSVIHKV-VVEVNE----------------------------------------
SbSRP-13        --GIYVSAVRQTC-AVEVDE----------------------------------------
SbSRP-14        --GLYVSAMRQTC-AVEVDE----------------------------------------
OsSRP-1         --GLYVSTMRQKC-AVEVDE----------------------------------------
OsSRP-2         --RLYVSAMRQKC-AVEVDE----------------------------------------
SbSRP-5         --GAFVAGVYHKA-TVEVDE----------------------------------------
SbSRP-6         --GLFISGVYHKA-TVEVDE----------------------------------------
OsSRP-4         --GLFIAEVYHQA-TIEVDE----------------------------------------
                          :     .: :

CrSRP-1         ------------------EGTVAAAAT------AVMMLRCALP----MP---TPEFIFNR
VcSRP-1         ------------------EGTVAAAAT------AVMMLRCVPA----P----PEEIIFNR
SbSRP-11        ------------------NGIKDTSVT--------MGIGKPRP---------GEHFVADH
OsSRP-14        ------------------KGIEETSVS--------MGLGKPLP---------AQHFKADH
SmSRP-1         ------------------EGTEAAAAS---AA-VVTLRALNVQ----ME---PEDFVADH
PpSRP-1         ------------------VGTEAAAVT---TV-EISLMSMIMY----TRE--PDTFVADR
PpSRP-2         ------------------KGTEAAAAT---AL-TVTAKSLQMY----TDD--PVEFVCDH
PpSRP-3         ------------------KGTEAAAAT---AA-TITLKGISMF----QD---PIDFICDH
PpSRP-4         ------------------KGTEAAAAT---AA-TITLRSLPMF----HE---PTDFVCDH
SbSRP-8         ------------------RGTMAAAGT---VA-MMYGSSMSRD----P----TVDFVADH
SbSRP-4         ------------------KGTVAAAGT------VGNMMGFAMP----DDL--IVDFVADH
OsSRP-7         ------------------EGTVAAAGT------VGEIMGFAMP----DDQ--IVDFVADH
MtSRP-8         ------------------RETVAASVS------TSVEVKCYTP---------SVDFVADH
SbSRP-7         ------------------EGTKAASAT---A--VAMGFGCAHV----EA---PVDFVADH
OsSRP-3         ------------------EGTEAAAAT---A--VVMTLGCAAP----SAPVHVVDFVADH
AtSRP-4         ------------------VGSKAAAAA------AFRGIGCRRP----PPE--KHDFVADH
AtSRP-7         ------------------EGAEAAAAT---AD-GDCGCSLDFV----EPPK-KIDFVADH
AtSRP-1         ------------------EGTEAAAAT---TV-VVVTGSCLWE----PKK--KIDFVADH
AtSRP-6         ------------------KGTEAVTFT---AF-RSAYLGCALV----K----PIDFVADH
FvSRP-12        ------------------DGTEASAVT---VS-VLYGSGGGPP----PPP--EVDFVADH
FvSRP-7         ------------------EGTEASAVT-------AQISGGRAP----PV---EIDFVADH
FvSRP-11        ------------------EGTEASAVT------VTQMCGGGAP----PV---EIDFVADH
```

```
FvSRP-2         ------------------NGTTAAAAT---CA-QEEESMGSPT----KPKV-IKEFLADH
FvSRP-1         ------------------NGTTAATAT---CA-ELVWSMGEPP----KPKV-IEEFVADH
FvSRP-10        ------------------NGTTAAAAT---CA-QVYCSMGSPP----KPKE-IEEFVADH
FvSRP-9         ------------------NGTTAAAAT---CS-VTS-SAGKVS----EPDV-KLDFVADH
FvSRP-14        ------------------NGTTAAAAT---YA-VISCSAGKAS----EPDE-KLDFVADH
FvSRP-6         ------------------EGTEAAAVY---IT-RGPGYSSGID----PPKPIPVDFVADH
FvSRP-4         ------------------EGTEAAAAT---A--AVAAFGCAAP----CPVIEKIDFVADH
FvSRP-13        ------------------KGTEAAAVT---SW-GGWMMCDEAP----PP---PIDFVADH
FvSRP-5         ------------------KGTEAAAVT---AA-VVRMYCAREP----PR---PIDFVADH
FvSRP-8         ------------------KGTEAAAVT---ATGTMCSAGKPVP----PP---PIDFVADH
AtSRP-2         ------------------EGTEAAAVS---VA-IMMPQCLMRN----P------DFVADH
AtSRP-3         ------------------EGTEAAAVS---VG-VVSCTSFRRN----P------DFVADR
AtSRP-8         ------------------EGTEAAAVS------VASMTKDMLL---------MGDFVADH
SbSRP-16        ------------------EGTEAAAAS------AIVTRGTSLL----RRH--PDDFITDH
OsSRP-6b        ------------------EGTEATAAV------MVSMEHSRPR---------RLNFVADH
OsSRP-5         ------------------EGTEAAAAS---AA-VVSFRSAPV----------TVDFVADH
OsSRP-6a        ------------------EGTEAAAAT---AA-VITLRSAPI----------AEDFVADH
SbSRP-2         ------------------EGTEAAAAS---AA-VVMLCSFRM----------PTDFVADH
SbSRP-3         ------------------EGTEAAAAS---AA-TVVLRSFAM----------PQDFVADH
AtSRP-5         ------------------EGTEAAAAS---AG-VIKLRGLLME----ED---EIDFVADH
FvSRP-3         ------------------EGTEAAAAS---AG-VIKLRGL-------PQ---TMDFVADH
MtSRP-11        ------------------VGTEAAAVT------VTGIAGCSQF----TSIPTPIDFVADH
MtSRP-9         ------------------KGTEAAAVT------RTGLLGCARP----TSIPTPIDFVADH
MtSRP-6         ------------------EGTKAAAVT---VS-MLCGCSRYSP----PPPP-PIDFVADH
MtSRP-16        EEFSGTILFVGKVVNPLDEGTKAAAVT---VS-VLCGCSRYSP----PPPP-PIDFVADH
MtSRP-3         ------------------QGTKAAALLALYTP-MTCGSGLISP----PPI--PIDFVADH
MtSRP-4         ------------------KGTEAAAVTSMGQK-VTCGRRTGPP----PT---PIDFVADH
MtSRP-10        ------------------AGTEAAAAT---FE-CMGFGLCLDD----TP---RIEFVADH
MtSRP-12        ------------------AGTEAAAAT---AF-IEAEYGMSEV----EDDTSKIEFVADH
MtSRP-1         ------------------EGTEVAKCI--------------------H---REDFVADH
MtSRP-13        ------------------EGVEAAAAT------AAILSKGFSF----PS---QLDFVADH
MtSRP-14        ------------------EGAEAVAVS---AA-FICS-EGIRF----PT---QLDFVADH
MtSRP-5         ------------------EGTKAAAAT---AG-LLCG-SSLSL----PT---DINFVADH
MtSRP-2         ------------------EGTEAVAAT---AT-EVFTSSGMGF----PT---RLDFVAGH
MtSRP-7         ------------------EGTEAAAAT---AA-TILLRSAMSI----PP---RLDFVADH
MtSRP-15        ------------------EGTEAAAIS----V-LFMQAQCMRR----PT---RMDFVVDH
SbSRP-12        ------------------VGTEAAAVT------VHLRGGGGPP----PD---LVEFNADH
SbSRP-1         ------------------EGTEAVAVT---YV-GVFAPSCAPP----GYRPETVDFVADH
SbSRP-9         ------------------GGTVANGAT---ASYMLCGASAIME----QPA--TVDFVADH
OsSRP-10        ------------------EGTEAAAAT---AV-CLTFASAAPS----SRRPARVDFVADH
OsSRP-8         ------------------EGTEAAAAT---AV-LMEGAARYAP----PPPP-REDFVADH
OsSRP-12        ------------------EGTEVAAAT---VV-IMKGRARRPS----PAPA-PVDFVADH
SbSRP-15        ------------------EGTEAAAST---AC-VMKKLCRSSR----L----PVNFVADH
OsSRP-9         ------------------EGTEAAAST---AC-TIRLLSMSY----------PEDFVADH
OsSRP-11        ------------------EGTEAAAAT---AC-TMKFLCLTLT----S----PVDFVADH
OsSRP-15        ------------------EGTVAAAAT------MTRMLPSGVP----PP---PVDFVAEH
SbSRP-10        ------------------EGSEAAAVTVESDD-DMGYSMFDDY----PPPPKPVNFVADH
OsSRP-13        ------------------EGSEAAAYT---EE-SDDDLGCSLYDDDYTPPPKLVDFVADH
SbSRP-13        ------------------EGTTAVAAI---------PPVRPP----PP---PMRFVADH
SbSRP-14        ------------------EARQRLRRR---APFLAQPVPAGHP----RRSPQPMSFVADH
OsSRP-1         ------------------EGTTAVEAM---YSPSSPGYSPGYQ----PPRPPPMSFVAEH
OsSRP-2         ------------------EGTTAVEAT---YS-CCSPTYSGPE----SPKPRPMSFVAEH
SbSRP-5         ------------------EGTVAAAAT---AV-SFCLSGSAIP---------PVDFVADR
SbSRP-6         ------------------AGTVAAAAT---AV-CMQQCARMGP----P----PVDFVADR
OsSRP-4         ------------------LGTVAAAST---AV-VMMQKGSSLP---------PVDFVADR
                                                                   :   .

CrSRP-1         PFAFIIMHTPTGLPAFVGT-----------------------------------------
VcSRP-1         PFAFMIYHNPTNLPAFVGV-----------------------------------------
SbSRP-11        PFFFVIKEEVSGSVIFMGH-----------------------------------------
OsSRP-14        PFFFMIREEVSGTVIFMGH-----------------------------------------
SmSRP-1         PFMFLVREDATGVVLFVGH-----------------------------------------
PpSRP-1         PFMFLIMEEVSNAIVFSGR-----------------------------------------
PpSRP-2         PFMFVIKEEHSNVVIFTGR-----------------------------------------
PpSRP-3         PFLFVIKEEVTNVIIFTGR-----------------------------------------
PpSRP-4         PYLFIIKEELTNVIIFTGR-----------------------------------------
SbSRP-8         PFAFFLMEDVSGVVVFAGH-----------------------------------------
SbSRP-4         PFLFFIMEENIGLIVFAGQ-----------------------------------------
OsSRP-7         PFLFFIVEEVSGLVVFAGQ-----------------------------------------
MtSRP-8         PFVFLVRELNSKTILFMGQ-----------------------------------------
SbSRP-7         PFVFLIKEELSGVVVFAGQ-----------------------------------------
OsSRP-3         PFMFLIKEDLTGVVVFAGQ-----------------------------------------
AtSRP-4         PFLFIVKEYRSGLVLFLGQ-----------------------------------------
AtSRP-7         PFLFLIREEKTGTVLFVGQ-----------------------------------------
AtSRP-1         PFLFLIREDKTGTLLFAGQ-----------------------------------------
AtSRP-6         PFLFLIREEQTGTVLFAGQ-----------------------------------------
FvSRP-12        PFMFLIKEDSTGAVLFMGH-----------------------------------------
FvSRP-7         PFMFFNREDSTGTVLFMGT-----------------------------------------
FvSRP-11        PFVFFIREDRTGTVLFMGT-----------------------------------------
FvSRP-2         PFMFLVRENCTGTVLFIGQKSPDETAATSLESLFSIRLLSKSPKKTTARTLTLSPDRFLQ
FvSRP-1         PFMFLIRENRTRTVMFMGQ-----------------------------------------
FvSRP-10        PSMFLIRENRTGTVLFMGQ-----------------------------------------
FvSRP-9         PLHVCDKGRHDGNSALHGA-----------------------------------------
```

```
FvSRP-14        PFMFLIREDMTGTVLFSLH-----------------------------------------
FvSRP-6         PFLYLIREDVTGTVMFVGH-----------------------------------------
FvSRP-4         PFLFLIREEVTGAVMFVGH-----------------------------------------
FvSRP-13        PFLYLIREEVTKTVMFVGQ-----------------------------------------
FvSRP-5         PFLYLIREEVTKTVMFVGQ-----------------------------------------
FvSRP-8         PFLYLIREEATKTVMFVGQ-----------------------------------------
AtSRP-2         PFLFTVREDNSGVILFIGQ-----------------------------------------
AtSRP-3         PFLFTVREDKSGVILFMGQ-----------------------------------------
AtSRP-8         PFLFTVREEKSGVILFMGQ-----------------------------------------
SbSRP-16        PFLFLIREDTTGVVLFVGH-----------------------------------------
OsSRP-6b        PFMFLIREDVTGVILFIGH-----------------------------------------
OsSRP-5         PFLFLIREDMTGVVLFIGH-----------------------------------------
OsSRP-6a        PFLFLIQEDMTGVVLFVGH-----------------------------------------
SbSRP-2         PFLFLIREDTTGVLLFVGH-----------------------------------------
SbSRP-3         PFLFLIREDLTGVVLFVGH-----------------------------------------
AtSRP-5         PFLLVVTENITGVVLFIGQ-----------------------------------------
FvSRP-3         PFLFLIKEEMTGTVLFIGH-----------------------------------------
MtSRP-11        PFLFFIREDLSGTILFVGQ-----------------------------------------
MtSRP-9         PFMFLIRDDLSGTILFVGQ-----------------------------------------
MtSRP-6         PFLFLIREEFSGTILFVGK-----------------------------------------
MtSRP-16        PFLFLIREEFSGTILFVGK-----------------------------------------
MtSRP-3         PFLFLIREDLSGTVLSIGQ-----------------------------------------
MtSRP-4         PFLFLIREDLSGTVLFIGQ-----------------------------------------
MtSRP-10        PFLFLIRDDLSGTVLFIGQ-----------------------------------------
MtSRP-12        PFLFLIREDLSGTVLFIGQ-----------------------------------------
MtSRP-1         PFLFLIREVSTGTILFAGQ-----------------------------------------
MtSRP-13        PFLFLIREDLTGTIIFVGQ-----------------------------------------
MtSRP-14        PFLFLIREDWTGTILFVGQ-----------------------------------------
MtSRP-5         PFLFIIREDLTGTIVFVGQ-----------------------------------------
MtSRP-2         PFLFMIREELTGTIIFVGQ-----------------------------------------
MtSRP-7         PFLFMIREDLTGTIIFVGQ-----------------------------------------
MtSRP-15        PFLYLIRDDLAGTIIFVGQ-----------------------------------------
SbSRP-12        PFTFFIMEERSGVIVFAGH-----------------------------------------
SbSRP-1         PFAFFVMEEVSGAVVFAGC-----------------------------------------
SbSRP-9         PFVFFVIEEVSRAIIFVGR-----------------------------------------
OsSRP-10        PFAFLVLEESSGAVLFAGH-----------------------------------------
OsSRP-8         PFAFFVVEESSGAVLFAGH-----------------------------------------
OsSRP-12        PFAFFVVEESSGAVLFAGH-----------------------------------------
SbSRP-15        PFVFFVVEEVSRVIVFMGH-----------------------------------------
OsSRP-9         PFAFFVVEETSGAVLFAGH-----------------------------------------
OsSRP-11        PFAFFVVEEKSDAVLFAGH-----------------------------------------
OsSRP-15        PFAYFIVEEMSSAVVFAGH-----------------------------------------
SbSRP-10        PFAFFIIQETSGAVVFAGH-----------------------------------------
OsSRP-13        PFAFFIVEERSQAIVFAGH-----------------------------------------
SbSRP-13        PFMFAIVEYEKAEVLFLGH-----------------------------------------
SbSRP-14        PFMFAIVEYEKAELLFLSH-----------------------------------------
OsSRP-1         PFMFAIVEYKKAQVLFLGH-----------------------------------------
OsSRP-2         PFMFAIVEYEKAQVLFLGH-----------------------------------------
SbSRP-5         PFLFAVVEERSSAVLFFGH-----------------------------------------
SbSRP-6         PFLFAIVEERSGAVLFLGH-----------------------------------------
OsSRP-4         PFLFAVVEELTGAVLFLGH-----------------------------------------
                *

CrSRP-1         -------------------V-NDPSQ----------------------------------
VcSRP-1         -------------------V-HDPVAA---------------------------------
SbSRP-11        -------------------I-LDPSSQS--------------------------------
OsSRP-14        -------------------V-LDPSSRT--------------------------------
SmSRP-1         -------------------V-NDPSV----------------------------------
PpSRP-1         -------------------V-TNPSKQA--------------------------------
PpSRP-2         -------------------V-TDPSLAQ--------------------------------
PpSRP-3         -------------------I-TDPSVEK--------------------------------
PpSRP-4         -------------------I-TDPSVGN--------------------------------
SbSRP-8         -------------------V-INPLLAPLP------------------------------
SbSRP-4         -------------------V-VNPLLH---------------------------------
OsSRP-7         -------------------V-VNPLLH---------------------------------
MtSRP-8         -------------------V-LNPTEGYDDEQVS--------------------------
SbSRP-7         -------------------V-IDPSISQ--------------------------------
OsSRP-3         -------------------V-TNPSSST--------------------------------
AtSRP-4         -------------------V-MDPSKH---------------------------------
AtSRP-7         -------------------I-FDPSGPCSGSNSDSDDY----------------------
AtSRP-1         -------------------I-FDPSELSSALDRA--------------------------
AtSRP-6         -------------------I-FDPSA----------------------------------
FvSRP-12        -------------------V-LNPLDG---------------------------------
FvSRP-7         -------------------V-LNPLAG---------------------------------
FvSRP-11        -------------------V-LNPLAG---------------------------------
FvSRP-2         ASMSTKNRLNVVNLACLEKVRLDSLNDTTRRSRCFICEERFDHYVGVKECITRLPCRHLF
FvSRP-1         -------------------V-LNPLAGKYHLCGKMGQMETATSTSGTNGTDEAKERERKE
FvSRP-10        -------------------V-LNPLAG---------------------------------
FvSRP-9         -------------------G-AQSSCGLIFIIVGRYSCTSFSL-----------------
FvSRP-14        -------------------G-AGPQSSSGLITIILLEDVLCKQVNFLGFHYASYFCSLDY
FvSRP-6         -------------------V-LNPIKEK--------------------------------
FvSRP-4         -------------------V-LDPIAE---------------------------------
FvSRP-13        -------------------V-LNPIED---------------------------------
```

```
FvSRP-5         -------------------V-LNPIED---------------------------------
FvSRP-8         -------------------V-LNPIEDSTDFK----------------------------
AtSRP-2         -------------------V-LDPSKH---------------------------------
AtSRP-3         -------------------V-LDPSKH---------------------------------
AtSRP-8         -------------------V-LDPSIH---------------------------------
SbSRP-16        -------------------V-VDPLLA---------------------------------
OsSRP-6b        -------------------V-VNPLLAV--------------------------------
OsSRP-5         -------------------V-VNPLL----------------------------------
OsSRP-6a        -------------------V-AAE------------------------------------
SbSRP-2         -------------------V-VNPLLAP--------------------------------
SbSRP-3         -------------------V-VNPLLAP--------------------------------
AtSRP-5         -------------------V-VDPLH----------------------------------
FvSRP-3         -------------------LNMIRLISLEIV-----------------------------
MtSRP-11        -------------------V-LNPLLK---------------------------------
MtSRP-9         -------------------V-LNPLVGRS-------------------------------
MtSRP-6         -------------------V-VNPLDG---------------------------------
MtSRP-16        -------------------V-VNPLDG---------------------------------
MtSRP-3         -------------------V-LNPLVM---------------------------------
MtSRP-4         -------------------V-LNPLLK---------------------------------
MtSRP-10        -------------------V-LNPLDM---------------------------------
MtSRP-12        -------------------V-LNPLDM---------------------------------
MtSRP-1         -------------------V-LNPVVG---------------------------------
MtSRP-13        -------------------V-LNPLTG---------------------------------
MtSRP-14        -------------------V-LNPLDE---------------------------------
MtSRP-5         -------------------V-LNPLISNIALLPSNPNISSCWVVFGTTTGFWSSASFTHQ
MtSRP-2         -------------------V-FNPLAG---------------------------------
MtSRP-7         -------------------V-LNPLAG---------------------------------
MtSRP-15        -------------------V-LNPLDG---------------------------------
SbSRP-12        -------------------V-LDPTK----------------------------------
SbSRP-1         -------------------V-LDPSQ----------------------------------
SbSRP-9         -------------------I-VDPSVSG--------------------------------
OsSRP-10        -------------------V-VDPTDE---------------------------------
OsSRP-8         -------------------V-VDPTKS---------------------------------
OsSRP-12        -------------------V-VDPTNPSQL------------------------------
SbSRP-15        -------------------V-LDPTKSEHEK-----------------------------
OsSRP-9         -------------------V-LDPTSSSE-------------------------------
OsSRP-11        -------------------V-LDPTSLE--------------------------------
OsSRP-15        -------------------I-VDPSME---------------------------------
SbSRP-10        -------------------V-LDPSEEV--------------------------------
OsSRP-13        -------------------V-LDPSEEE--------------------------------
SbSRP-13        -------------------V-MDPSKDD--------------------------------
SbSRP-14        -------------------V-IDPSNED--------------------------------
OsSRP-1         -------------------V-MDPSKEDQ-------------------------------
OsSRP-2         -------------------V-MDPSNEE--------------------------------
SbSRP-5         -------------------V-VNPIAE---------------------------------
SbSRP-6         -------------------V-VNPLVG---------------------------------
OsSRP-4         -------------------V-VNPLAE---------------------------------


CrSRP-1         ------------------------------------------------------------
VcSRP-1         ------------------------------------------------------------
SbSRP-11        ------------------------------------------------------------
OsSRP-14        ------------------------------------------------------------
SmSRP-1         ------------------------------------------------------------
PpSRP-1         ------------------------------------------------------------
PpSRP-2         ------------------------------------------------------------
PpSRP-3         ------------------------------------------------------------
PpSRP-4         ------------------------------------------------------------
SbSRP-8         ------------------------------------------------------------
SbSRP-4         ------------------------------------------------------------
OsSRP-7         ------------------------------------------------------------
MtSRP-8         ------------------------------------------------------------
SbSRP-7         ------------------------------------------------------------
OsSRP-3         ------------------------------------------------------------
AtSRP-4         ------------------------------------------------------------
AtSRP-7         ------------------------------------------------------------
AtSRP-1         ------------------------------------------------------------
AtSRP-6         ------------------------------------------------------------
FvSRP-12        ------------------------------------------------------------
FvSRP-7         ------------------------------------------------------------
FvSRP-11        ------------------------------------------------------------
FvSRP-2         HGKCILKRSMTSQSCPIPSCLYPMPTIKEEEVAQSSKPPGSRRLNWHGVLAVATGFILTA
FvSRP-1         EEQSKIKFKRKSKLKSYRQGWEQSFLRRSAISVAILKATHTGGTKAYRCGHVHSRVKTGY
FvSRP-10        ------------------------------------------------------------
FvSRP-9         ------------------------------------------------------------
FvSRP-14        SFSLLIVLS---------------------------------------------------
FvSRP-6         ------------------------------------------------------------
FvSRP-4         ------------------------------------------------------------
FvSRP-13        ------------------------------------------------------------
FvSRP-5         ------------------------------------------------------------
FvSRP-8         ------------------------------------------------------------
AtSRP-2         ------------------------------------------------------------
AtSRP-3         ------------------------------------------------------------
```

```
AtSRP-8         ------------------------------------------------------------
SbSRP-16        ------------------------------------------------------------
OsSRP-6b        ------------------------------------------------------------
OsSRP-5         ------------------------------------------------------------
OsSRP-6a        ------------------------------------------------------------
SbSRP-2         ------------------------------------------------------------
SbSRP-3         ------------------------------------------------------------
AtSRP-5         ------------------------------------------------------------
FvSRP-3         ------------------------------------------------------------
MtSRP-11        ------------------------------------------------------------
MtSRP-9         ------------------------------------------------------------
MtSRP-6         ------------------------------------------------------------
MtSRP-16        ------------------------------------------------------------
MtSRP-3         ------------------------------------------------------------
MtSRP-4         ------------------------------------------------------------
MtSRP-10        ------------------------------------------------------------
MtSRP-12        ------------------------------------------------------------
MtSRP-1         ------------------------------------------------------------
MtSRP-13        ------------------------------------------------------------
MtSRP-14        ------------------------------------------------------------
MtSRP-5         VCGGAGAQRLVVVFGDPLGWMVHRGRSRFRSLSGWFSVLHHCSGFAFSVVLVNYV-----
MtSRP-2         ------------------------------------------------------------
MtSRP-7         ------------------------------------------------------------
MtSRP-15        ------------------------------------------------------------
SbSRP-12        ------------------------------------------------------------
SbSRP-1         ------------------------------------------------------------
SbSRP-9         ------------------------------------------------------------
OsSRP-10        ------------------------------------------------------------
OsSRP-8         ------------------------------------------------------------
OsSRP-12        ------------------------------------------------------------
SbSRP-15        ------------------------------------------------------------
OsSRP-9         ------------------------------------------------------------
OsSRP-11        ------------------------------------------------------------
OsSRP-15        ------------------------------------------------------------
SbSRP-10        ------------------------------------------------------------
OsSRP-13        ------------------------------------------------------------
SbSRP-13        ------------------------------------------------------------
SbSRP-14        ------------------------------------------------------------
OsSRP-1         ------------------------------------------------------------
OsSRP-2         ------------------------------------------------------------
SbSRP-5         ------------------------------------------------------------
SbSRP-6         ------------------------------------------------------------
OsSRP-4         ------------------------------------------------------------


CrSRP-1         --------------------
VcSRP-1         --------------------
SbSRP-11        --------------------
OsSRP-14        --------------------
SmSRP-1         --------------------
PpSRP-1         --------------------
PpSRP-2         --------------------
PpSRP-3         --------------------
PpSRP-4         --------------------
SbSRP-8         --------------------
SbSRP-4         --------------------
OsSRP-7         --------------------
MtSRP-8         --------------------
SbSRP-7         --------------------
OsSRP-3         --------------------
AtSRP-4         --------------------
AtSRP-7         --------------------
AtSRP-1         --------------------
AtSRP-6         --------------------
FvSRP-12        --------------------
FvSRP-7         --------------------
FvSRP-11        --------------------
FvSRP-2         MLASRFWDLCVSYI------
FvSRP-1         FIHIVDWCRIRCSKTEYLVM
FvSRP-10        --------------------
FvSRP-9         --------------------
FvSRP-14        --------------------
FvSRP-6         --------------------
FvSRP-4         --------------------
FvSRP-13        --------------------
FvSRP-5         --------------------
FvSRP-8         --------------------
AtSRP-2         --------------------
AtSRP-3         --------------------
AtSRP-8         --------------------
SbSRP-16        --------------------
OsSRP-6b        --------------------
OsSRP-5         --------------------
```

```
OsSRP-6a        --------------------
SbSRP-2         --------------------
SbSRP-3         --------------------
AtSRP-5         --------------------
FvSRP-3         --------------------
MtSRP-11        --------------------
MtSRP-9         --------------------
MtSRP-6         --------------------
MtSRP-16        --------------------
MtSRP-3         --------------------
MtSRP-4         --------------------
MtSRP-10        --------------------
MtSRP-12        --------------------
MtSRP-1         --------------------
MtSRP-13        --------------------
MtSRP-14        --------------------
MtSRP-5         --------------------
MtSRP-2         --------------------
MtSRP-7         --------------------
MtSRP-15        --------------------
SbSRP-12        --------------------
SbSRP-1         --------------------
SbSRP-9         --------------------
OsSRP-10        --------------------
OsSRP-8         --------------------
OsSRP-12        --------------------
SbSRP-15        --------------------
OsSRP-9         --------------------
OsSRP-11        --------------------
OsSRP-15        --------------------
SbSRP-10        --------------------
OsSRP-13        --------------------
SbSRP-13        --------------------
SbSRP-14        --------------------
OsSRP-1         --------------------
OsSRP-2         --------------------
SbSRP-5         --------------------
SbSRP-6         --------------------
OsSRP-4         --------------------
```

D. I6 Cereal.

```
VvATI-1         -----------------------------------MAKLSIFAATLLLLLAISNAT--IY
RcATI-5         -----------------------------------MAKLIPAVALISVLLFIIANASFAY
RcATI-1a        -----------------------------------MAKLIPTIALVSVLLFIIANASFAY
RcATI-2a        -----------------------------------MAKLIPTIALVSVLLFIIANASFAY
RcATI-6a        -----------------------------------MAKLIPTVTLISVLLVIIANASFAY
MgATI-1         ----------------------------------MANKATLAAALLVALVTLTSATSYTT
RcATI-6b        ------------------------------------------------------------
RcATI-4         MQKLYHTINNLHHLHSNTHRSNSYLFLSTARSQASSTMAKFAILLASFIALLFLVDASIY
RcATI-1b        ------------------------------------------------------------
RcATI-2b        ------------------------------------------------------------
RcATI-3         -----------------------------------MAKLAILLASFIALLFLVDAS--IY
OsATI-9         -------------------------------------MASKVVFFAAALMAAMVAI----
SbATI-8         -----------------------------------MAKIAAVAATAALCLAALVAVAVGQ
OsATI-10        -------------------------------MAPNKGDLCLPVENEISNYTMVLSVALDS
BdATI-2         -----------------------------------MAYNKVVAFSVLLVLSMLAAATNSM
OsATI-6         --------------------------------------MAFIKVVFSVLLPVVVSMLVAT
OsATI-8         -----------------MGELLSLSYKLTIKNFCKQKKMASNKVVISALLVVVVSVLAAT
OsATI-11        -------------------------------------MASNKVVFSVLLLVVLSVLAAAM
OsATI-7         -------------------------------------MASNKVVFSVLLLAVVSVLAATA
OsATI-1         ----------------MKTLNLSLSYKFRLKIFCKQKAMASNKVVFSALLLIIVSVLAAT
OsATI-5         -----------------------------MASKNSKLSVSSVAVALVALMVVVVGAVVAA
BdATI-3         -----------------------------------MTTSKKKLVSSVVLLAAVILLAATA
OsATI-3         -----------------------------------MALASDKFVLSAIVLAVLTVAAAAA
OsATI-4         -----------------------------------MASASDKLVLSAIVLAVLAAVVAAA
ZmATI-1         -------------------------------MACCTTTSRLVLSAAAALLAVAGGA----
SbATI-5         ------------------------------MACCTSRLVLAVSVAASVLLAAVLSAAAA-
SbATI-6         ------------------------------MACFTNHLVLVLSSSAAVLLAVVLGTAAS-
SbATI-2         ------------------------------MASTTRSSYIILLSAAAVLLSVVGGAM---
SbATI-3         --------------------------------MASTRSSSYILLSAAVLLSVLAAT----
BdATI-1         ---------------------------------MASNQKLLFLCSAAVLLSALITAAAAD
SbATI-1         -----------------------------------TA-----------------------
SbATI-4         ------------------------------------MVSGHLLLSAGVLLSVLAACAATV
OsATI-2         ------------------------------------MASKNLLLSAAVLLSVLAIAAAAA
SbATI-7         ----------------------------------MASSSSSNLLAAAVLLSVLAAAGASA
ZmATI-2         MPLTWCSSIFQVTNANCTKKSIERPSTGELMASSSSSSHRRLILAAAVLLSVLAAASASA


VvATI-1         QTTVITRDDG----------------SEFGQ--FQGSQSQRCRQQAEQQ-QGGQ--GDVL
RcATI-5         RTTITTVEVDD------TNTQER---CFRD---LRGKEFRACQMYLSQS-SSRRSTDGEV
RcATI-1a        RTTITTIEIDESKGEREGSSSQQ---CRQE---VQRKDLSSCERYLRQS-SSRRSPGEEV
RcATI-2a        RTTITTIEIDESKGEREGSSSQQ---CRQE---VQRKDLSSCERYLRQS-SSRRSPGEEV
RcATI-6a        GTTIEIDDTKAGGE---GSRSQQ---CHQE---FQRKDLSSCEQYIRQS-SSRRSPGEEL
MgATI-1         TVTTTTIDDEAT-----RGEQQE---CRQH---VQGRLYLSCQRYLSQR-SQYGGDEEEV
RcATI-6b        -----------------PNQEG----CRQHI--IGQQKLRQCQEYITQQ-YGV-------
RcATI-4         RTTVIVDEEDAN-----PSYQS----CREQV--VMRQYLSPCQEYIRQQ-VAGLGLSHG-
RcATI-1b        -----------------PSQQG----CRGQI--QEQQNLRQCQEYIKQQ-VSG-------
RcATI-2b        -----------------PSQQG----CRGQI--QEQQNLRQCQEYIKQQ-VSG-------
RcATI-3         RTTVIVDAEDANNILRQDIHQQQRGSCSAEI--EKQQNLWRCQQYIKKE-VTG-------
OsATI-9         --SGAQLSESEM-----RFRDRQ---CQRE---VQDSPLDACRQVLDRQ-LTGR---ER-
SbATI-8         GVVERQRLKDLQ--------------CWQE---VQENPLGACRQVLDRQLTGGMRYGIGP
OsATI-10        CLHACGNDNNGRPPLLGGVYPQPALPAKNRL--LDVYPLPRCRTMVKGQ-CIGGGAAGNV
BdATI-2         AAAAAARYGAVA-----NTPGEW---CWAGMG-FPVYPFPRCRALVKSQ-CLGAQAAQS-
OsATI-6         TTMADHRGQVV------YTPGQL---CAAGRG-YPMYPLPRCRALAKRQ-CAGGAVDE--
OsATI-8         TTMADHHQEQVV-----YTPGQL---CQPGIG-YPTYPLPRCRAFVKRQ-CVAPGTVDE-
OsATI-11        ATMADHHQV--------YSPGEQ---CRPGIS-YPTYSLPQCRTLVRRQ-CVGRGAASAA
OsATI-7         TMAEYHHQDQVV-----YTPGPL---CQPGMG-YPMYPLPRCRALVKRQ-CVGRGTAAAA
OsATI-1         ATMADHHKDQVV-----YSLGER---CQPGMG-YPMYSLPRCRAVVKRQ-CVGHGAPGGA
OsATI-5         AAGGEEYYGGVA-----RAADG----CEPGQGVVPKDPLPGCRAYLLRR-CGGGDPP---
BdATI-3         AAEGADDYGE----------------CRVGKK-IPYNPLPGCREYITRW-CAVRNDPKKQ
OsATI-3         GYGGY------------GDVGEY---CRVGKA-VSRNPVPSCRNYIARW-CAVAGGRLDS
OsATI-4         SGY--------------GDVGEY---CRVGKA-VSRNPVPSCRNYIARW-CAAAGGRMDS
ZmATI-1         -----------------AEAPSY---CAPGQA-IPYRPLSGCTWYVASR-SCDVIEA---
SbATI-5         -----------------SAPPSY---CAVGKA-IPHSPLSGCVWYVATR-SCDMAVS---
SbATI-6         -----------------GAPPSY---CAVGKA-IPHSPLSGCAWYVATR-SCDMAVS---
SbATI-2         -----------------AARTNW---CEPGLV-IPLNPLPSCRTYLVRR-TCGLG-----
SbATI-3         -----------------AAAANW---CEPGLV-IPLNPLPSCRTYMVRR-ACGVS-----
BdATI-1         GVQED------------DQNGEWW--CYPGKA-FPHNPLGSCRTYVISR-ACHRGPGLPM
SbATI-1         --------------------------CAPGMA-IPIPPVPSCRIYAVSR-TCGLGGP---
SbATI-4         DVTA----------------------CAPGLA-IPAPPVPTCRIYTVSR-TCGLGGP---
OsATI-2         TASAATTS------------------CQPGMA-IPHDPLRGCRRYVLRR-ACGLAAGGRL
SbATI-7         GTT-----------------------CVPGWG-IPHNPLPSCRWYVASR-TCG-------
ZmATI-2         GTS-----------------------CVPGWA-IPHNPLPSCRWYVTSR-TCG-------
                                          .              *
```

```
VvATI-1         ILRGIRNQQQQEQQWLRQCCQALQNM-D--QQCQCEGLRQIVQRV---------------
RcATI-5         LEMPGEKDQQER-HQLQECCNELKQV-R--DECQCEALQVAVEKQIESEQ----------
RcATI-1a        LRMPGDENQQQESQQLQQCCNQVKQV-R--DECQCEAIKYIAEDQIQQGQ----------
RcATI-2a        LRMPGDENQQQESQQLQQCCNQVKQV-R--DECQCEAIKYIAEDQIQQGQ----------
RcATI-6a        LRMPRAEDQQQETQQLQQCCNQVEQL-R--HDCQCEAIKSIAEYQIQQGH----------
MgATI-1         VEMTTTGNPKERSQDLRSCCQQLHYV-K--QQCRCEAIKLAAEEVQQEGG----------
RcATI-6b        --QRSRRSDSNQQQSLRRCCDQIKQMYPETTQCRCEALYYAVEEKYLQGQ----------
RcATI-4         --YNPR---------LRDCCERIQSM-Q--TQCRCEGLRMAIDQQKSKGQ----------
RcATI-1b        --QGPRRSDNQE-RSLRGCCDHLKQM-Q--SQCRCEGLRQAIEQQQSQGQ----------
RcATI-2b        --QGPRRSDNQE-RSLRGCCDHLKQM-Q--SQCRCEGLRQAIEQQQSQGQ----------
RcATI-3         --WGPRM------DYYPLCCDHLEQM-T--SQCRCEGLRQAIQKQQSEGQ----------
OsATI-9         --FQPMFRRPGALGLRMQCCQQLQDV-S--RECRCAAIRRMVRSYEESMP----MPLEQG
SbATI-8         FRWGT--------GLRMRCCQQLQDV-S--RECRCAAIRSMVRGYEETMPPLEKGWWGQQ
OsATI-10        DE-----------QVWRDCCRQLATINN--NLCRCPVLSHKLVGMYKELG----------
BdATI-2         -------------SVREDCCRQLAAIPD--DFCKCPALGAMRDSMYKELG----------
OsATI-6         -------------QVRQDCCRQLAAIDD--SFCRCPALSHMLVGMYKELG----------
OsATI-8         -------------QVRRGCCRQLAAIDS--SWCRCDALNHMLRIIYRESG----------
OsATI-11        DE-----------QVWQDCCRQLAAVDD--GWCRCGALDHMLSGIYRELG----------
OsATI-7         E------------QVRRDCCRQLAAVDD--SWCRCEAISHMLGGIYRELG----------
OsATI-1         VDE----------QLRQDCCRQLAAVDD--SWCRCSALNHMVGGIYRELG----------
OsATI-5         -------------GVRARCCHQLREV-A--ARCRCDALRAMVEVLVEEEE----------
BdATI-3         --LVPD-------EVKRRCCGEVSEL-P--KGCRCDALGILANGVITEEG----------
OsATI-3         GKQPPR-------QLLEPCCRELAAV-P--MQCRCDALSVLVRGVVTEEG----------
OsATI-4         RKQPPR-------EFLEPCCRELAAV-P--MQCRCDALSVLVRGVVTEEG----------
ZmATI-1         --MLPNRA-----VLKETCCSQLRDI-P--AECRCRALRVMMETPLVVGA----------
SbATI-5         --MLP--------VLKETCCGQLEQI-P--AECRCRALRVMMEAPLVVGADTGAQRRCRA
SbATI-6         --MLP--------VLKETCCGQLEHI-P--AECRCRALRVMMEAPLVVGA----------
SbATI-2         --RGPFVPLP---VLKGRCCTELEKIVP---YCRCGALRIMMDGM--PGG----------
SbATI-3         --IGPVVPLP---VLKERCCSELEKIVP---YCRCSALRTALDSMMTG------------
BdATI-1         -------------LVKERCCRELGAVHP--DRCRREALRVLMDGVVVEGG----------
SbATI-1         --YGPVADPSP--VLKERCCQELAAV-P--SRCRCDALYYMMDTEF--GR----------
SbATI-4         --YGPVDPSP---VLKQRCCRELAAV-P--SRCRCAALGFMMDGVDAPLQ----------
OsATI-2         YDW----------SLKERCCQELAAV-P--AYCRCAALAYFMDGA-SEGR----------
SbATI-7         --IGPLLPLP---ELRRRCCRELADI-A--AYCRCTALSILIDGVIPPGP----------
ZmATI-2         --IGPRLPWP---ELKRRCCRELADI-P--AYCRCTALSILMDGAIPPGP----------
                                  **  :  :      *.   :

VvATI-1         ------------------------------------------------------------
RcATI-5         --------------------QMQ-------------------------------------
RcATI-1a        ---------------------LH-------------------------------------
RcATI-2a        ---------------------LH-------------------------------------
RcATI-6a        ---------------------LQ-------------------------------------
MgATI-1         --------------------QWQ-------------------------------------
RcATI-6b        ---------------------VE-------------------------------------
RcATI-4         ---------------------IL-------------------------------------
RcATI-1b        ---------------------LQ-------------------------------------
RcATI-2b        ---------------------LQ-------------------------------------
RcATI-3         ---------------------IE-------------------------------------
OsATI-9         ------------------WSSSS-------------------------------------
SbATI-8         PQPGYDYPCSQAGEGYGYGESGQ-------------------------------------
OsATI-10        ---------------TAAHGQPM-------------------------------------
BdATI-2         --------VVMKGEGVGDGTVEA-------------------------------------
OsATI-6         -----APA----------KGQPM-------------------------------------
OsATI-8         ---------------AADAGHPM-------------------------------------
OsATI-11        -----ATE----------AGHPM-------------------------------------
OsATI-7         ---------------APDVGHPM-------------------------------------
OsATI-1         -----ATD----------VGHPM-------------------------------------
OsATI-5         ------------------------------------------------------------
BdATI-3         ------------------VKVGR-------------------------------------
OsATI-3         ---------------DRVAGMIS-------------------------------------
OsATI-4         ---------------DRVSGMIS-------------------------------------
ZmATI-1         ----------------------E-------------------------------------
SbATI-5         AQARFAPAVVAAGECGLRTVHGRPFCNKLDADRRPVLVVLSATAVLLAVVFFGAATTTAA
SbATI-6         ----------------------D-------------------------------------
SbATI-2         ------------------------------------------------------------
SbATI-3         ------------------------------------------------------------
BdATI-1         -----------------RVVEGR-------------------------------------
SbATI-1         ------------------------------------------------------------
SbATI-4         ------------------------------------------------------------
OsATI-2         ----------------------L-------------------------------------
SbATI-7         ----------------DAQLEGR-------------------------------------
ZmATI-2         ----------------DAQLEGR-------------------------------------
```

```
VvATI-1      ------------------------------------------------------------
RcATI-5      --------------REQY------------------------------------------
RcATI-1a     --------------GEES------------------------------------------
RcATI-2a     --------------GEES------------------------------------------
RcATI-6a     --------------GEES------------------------------------------
MgATI-1      --------------TGEL------------------------------------------
RcATI-6b     --------------EEYL------------------------------------------
RcATI-4      --------------GQDS------------------------------------------
RcATI-1b     --------------GQDV------------------------------------------
RcATI-2b     --------------GQDV------------------------------------------
RcATI-3      --------------GEDV------------------------------------------
OsATI-9      --------------SEYYGGEGS-------------------------------------
SbATI-8      --------------QQMYPPCRP-------------------------------------
OsATI-10     --------------DEVFPGCRR-------------------------------------
BdATI-2      --------------AEIFPGCRT-------------------------------------
OsATI-6      --------------DEVFPGCRR-------------------------------------
OsATI-8      --------------AEVFRGCRR-------------------------------------
OsATI-11     --------------AEVFPGCRR-------------------------------------
OsATI-7      --------------SEVFRGCRR-------------------------------------
OsATI-1      --------------AEVFPGCRR-------------------------------------
OsATI-5      ----------------APLACKK-------------------------------------
BdATI-3      --------------MEAVPGCDR-------------------------------------
OsATI-3      --------------QHAAPGCDA-------------------------------------
OsATI-4      --------------QHAAPGCDA-------------------------------------
ZmATI-1      --------------PGAQQRCRV-------------------------------------
SbATI-5      SAAAASAAPSYCAVGKAIPGCVWYVASRSCDVVAAMLPNRASLRATCCGQLQNVPAECRC
SbATI-6      --------------TGAQRRCRA-------------------------------------
SbATI-2      --------------GEDTPPCSW-------------------------------------
SbATI-3      --------------YEMRPTCSW-------------------------------------
BdATI-1      --------------LGDLRGCPR-------------------------------------
SbATI-1      --------------LQDFRGCTR-------------------------------------
SbATI-4      ----------------DFRGCTR-------------------------------------
OsATI-2      --------------LEDLPGCPR-------------------------------------
SbATI-7      --------------LEDLPGCPR-------------------------------------
ZmATI-2      --------------LEDLPGCPR-------------------------------------


VvATI-1      ------------------------------------------------------------
RcATI-5      ---------------------------QEVM-----------------------------
RcATI-1a     ---------------------------ERVA-----------------------------
RcATI-2a     ---------------------------ERVA-----------------------------
RcATI-6a     ---------------------------ERVG-----------------------------
MgATI-1      ---------------------------QQVY-----------------------------
RcATI-6b     ---------------------------FDAF-----------------------------
RcATI-4      ---------------------------RQAY-----------------------------
RcATI-1b     ---------------------------FEAF-----------------------------
RcATI-2b     ---------------------------FEAF-----------------------------
RcATI-3      ---------------------------REAF-----------------------------
OsATI-9      -------------------------SSEQGYYGEGSSEEGYYGEQQQQPGMTRVRLTRAR
SbATI-8      -------------------------GTGQKI--------------------ARVKLTKAR
OsATI-10     -------------------------DNMKCM-----------------------------
BdATI-2      ---------------------------EVMD-----------------------------
OsATI-6      ---------------------------GDMK-----------------------------
OsATI-8      ---------------------------GDIE-----------------------------
OsATI-11     ---------------------------GDLE-----------------------------
OsATI-7      ---------------------------GDLE-----------------------------
OsATI-1      ---------------------------GDLE-----------------------------
OsATI-5      -------------------------GAMAAI-----------------------------
BdATI-3      -------------------------ETIAFL-----------------------------
OsATI-3      -------------------------ATIAGM-----------------------------
OsATI-4      -------------------------ATIAGM-----------------------------
ZmATI-1      -------------------------A-QARF-----------------------------
SbATI-5      RALRVMMETTPLVVGADPGAQRRCRAAQARF-----------------------------
SbATI-6      -------------------------A-QPRF-----------------------------
SbATI-2      -------------------------GGQLEL-----------------------------
SbATI-3      -------------------------GGLLEF-----------------------------
BdATI-1      -------------------------QIQRGF-----------------------------
SbATI-1      -------------------------EMQRDL-----------------------------
SbATI-4      -------------------------EMQRTF-----------------------------
OsATI-2      -------------------------ETQRGL-----------------------------
SbATI-7      -------------------------AVQRRF-----------------------------
ZmATI-2      -------------------------EVQRGF-----------------------------
```

```
VvATI-1         RDRVRVRESSSR--R------------------------
RcATI-5         QKARSIPSSCGL----PEQ----CQIRTFFF---------
RcATI-1a        QRAGEIVSSCGV--R--------CMRQTRTN---------
RcATI-2a        QRAGEIVSSCGV--R--------CMRQTRTN---------
RcATI-6a        QRASDIVSSCGL--R--------CLRQIQKN---------
MgATI-1         ERARYLPRQCNF--RSPQQ----CQFRDLFL---------
RcATI-6b        KTARYLPLQCGV--E-PRE----CQ---------------
RcATI-4         NIAQDLPYTCGV--S-PQK----CRFGTRWGF--------
RcATI-1b        RTAANLPSMCGV--S-PTE----CRF--------------
RcATI-2b        RTAANLPSMCGV--S-PTE----CRF--------------
RcATI-3         RIAQDLPSRCGV--S-PSP----CQFEPGLGF--------
OsATI-9         QYAAQLPSMCRV--E-PQQ----CSIFAAGQY--------
SbATI-8         QYAAGMPMMCRL--SEPQE----CSVFSGGDQYY------
OsATI-10        -VVASLLALCGV--DIHIGIGGVC----------------
BdATI-2         RAIASIPAFCNQ--RIPIGTDGVCYWLSYYQHPKQVTSA-
OsATI-6         RVAASLPAFCNV--DIPIGIGGVCYWLSYPMNPATGH---
OsATI-8         RAAASLPAFCNV--DIPNGVGGVCYWLPGTGY--------
OsATI-11        RAAASLPAFCNV--DIPNGPGGVCYWLGYPRTPRTGH---
OsATI-7         RAAASLPAFCNV--DIPNGGGGVCYWLARSGY--------
OsATI-1         RAAASLPAFCNV--DIPNGTGGVCYWLGYPRTPRTGH---
OsATI-5         AEGLPGRDECDLDTRADDGGSRRCHLVIN-----------
BdATI-3         ASDLMEIRHCYI--------GYSCPLFGGGMD--------
OsATI-3         ASALTDYGRCNL--Q-HTG-FFGCPMFGGGMD--------
OsATI-4         ASALTDYGRCNL--QHTAG-SFACLMFGGGMD--------
ZmATI-1         APALVAAAECGL--LTAHG-RRFCNALDAE----------
SbATI-5         APAVVAAGECSL--RTVHG-RPVCNALDAE----------
SbATI-6         APAVVAAGECGL--RTVHG-WPFCNALDAE----------
SbATI-2         APTLVSEAECNL--MTIHG-RPFCYALGAEGTTTD-----
SbATI-3         APTLVSEAECNL--RTIHG-RPFCYALGAEGTTTSD----
BdATI-1         AATLLTPGECGL--RA------------------------
SbATI-1         ARRLTRAEECNL--PTVDG-G-------------------
SbATI-4         ARRLTRAAECNL--PTIDG--GMCHSLNSPRPVLESVSTY
OsATI-2         AAMLTTPGECNL--ETIHG-GPYCLELTDREMPKY-----
SbATI-7         AATLITEAECNL--PTITG-VAECPWILGGETMPSK----
ZmATI-2         AATLVTEAECNL--ATISG-VAECPWILGGGTMPSK----
                         .
```

E. I12 Bowman-Birk.

```
MtBBI-9         ------------------------------------------------------------
GmBBI-2         -----------------------------------------------------------M
GmBBI-4         -----------------------------------------------------------M
GmBBI-9         -----------------------------------------------------------M
GmBBI-5         -----------------------------------------------------------M
GmBBI-1         -----------------------------------------------------------M
GmBBI-3         -----------------------------------------------------------M
MtBBI-12        -------------------------------------------MYCYCYLPLHKENKCIC
MtBBI-1         -------------------------------------------MVAHTLARAKDIKRVIM
GmBBI-8         ------------------------------------------------------------
MtBBI-5         ------------------------------------------------------------
MtBBI-7         ------------------------------------------------------------
MtBBI-4         -----------------------------------------------------------M
MtBBI-2         ------------------------------------------------------------
GmBBI-7         ------------------------------------------------------------
MtBBI-6         ------------------------------------------------------------
GmBBI-6         ------------------------------------------------------------
MtBBI-8         ------------------------------------------------------------
GmBBI-12        ------------------------------------------------------------
MtBBI-11b       ---------------------------------------------------------MEL
GmBBI-10        ------------------------------------------------------------
GmBBI-11        ------------------------------------------------------------
MtBBI-3         ----------------------------------------------------------MD
MtBBI-11a       ---------------------------------------------------------MVL
MtBBI-10        ------------------------------------------------------------
OsBBI-9         ------------------------------------------------------------
SbBBI-1         ------------------------------------------------------------
ZmBBI-4         MRTQALFFLALALIGAVLAQSSNRHHHHYHHAHVQGKGRCPSAPTPTQLLPKSRNLGVFF
SbBBI-3         ------------------------------------------------------------
SbBBI-2         ------------------------------------------------------------
SbBBI-4         ------------------------------------------------------------
SbBBI-7         ------------------------------------------------------------
SbBBI-5         ------------------------------------------------------------
SbBBI-6         ------------------------------------------------------------
ZmBBI-2a        ----------------------MDMELEHGEAEDDRGSYSSSSKRSFGASSDATASSTPS
ZmBBI-3a        ---------------------------------------MRLRMNNHQQQLQAWRKKKGI
ZmBBI-1a        ----------------------------------------------------------MA
OsBBI-1a        ------------------------------------------------------------
OsBBI-2a        ------------------------------------------------------------
OsBBI-7b        ------------------------------------------------------------
OsBBI-8b        ------------------------------------------------------------
OsBBI-3a        ------------------------------------------------------------
OsBBI-6b        ------------------------------------------------------------
OsBBI-5a        ---------------------------------------------------------MTM
OsBBI-8a        ------------------------------------------------------------
OsBBI-6a        ------------------------------------------------------------
OsBBI-7a        ------------------------------------------------------------
BdBBI-3         ------------------------------------------------------------
BdBBI-5         ------------------------------------------------------------
ZmBBI-1b        ------------------------------------------------------------
BdBBI-1         ------------------------------------------------------------
ZmBBI-2b        ------------------------------------------------------------
ZmBBI-3b        ------------------------------------------------------------
BdBBI-2a        ------------------------------------------------------------
BdBBI-4a        ------------------------------------------------------------
BdBBI-2b        ------------------------------------------------------------
OsBBI-4b        ------------------------------------------------------------
BdBBI-4b        ------------------------------------------------------------
OsBBI-4a        ------------------------------------------------------------
BdBBI-6b        ------------------------------------------------------------
BdBBI-6a        -------------------------------------------------------MTKRG
OsBBI-3b        ------------------------------------------------------------
OsBBI-8c        ------------------------------------------------------------
OsBBI-2b        ------------------------------------------------------------
OsBBI-6c        ------------------------------------------------------------
OsBBI-7c        ------------------------------------------------------------
OsBBI-1b        ------------------------------------------------------------
OsBBI-5b        ------------------------------------------------------------


MtBBI-9         ---------MKLSFLLFLLGF----------------------------------IAIVV
GmBBI-2         GLKNNMVVLKVCLVLLFLVGG----TTSASL---------------------RLSELGLL
GmBBI-4         GLKNNMVVLKVCLVLLFLVGG----TTSANL---------------------RLSKLGLL
GmBBI-9         SLKNNMVVLKVCLLLLFLVGV----TAA--------------------------RMELSF
GmBBI-5         GLKNNMVVLKVCFLVLFLVGV--------------------------------TNARMEL
GmBBI-1         GLKNNMVVLKVCFLVLFLVGV--------------------------------TNAHMEL
GmBBI-3         GLKNNMVVLKVCFLVLFLVGV--------------------------------TNARMEL
MtBBI-12        KVMESNKNTFVKLSVLLLLLC----FTATIVD--------------ARFDSTPFTTRVLS
MtBBI-1         GLMNKKTMIKLAM-LIFLLSF----IATIDAR------------------------FDSK
GmBBI-8         ------MELKKVGLVLFLLG-----FTATTVDA-------------TGFNPNSLITQLLP
```

```
MtBBI-5         -----MDLKMKVVLLLFFLGLAT-------------------------------------
MtBBI-7         -----MGLKMKIALLLFFLG-----LTTTIID--------------ARFEPNSLITQVIS
MtBBI-4         ELMKKKATMISLAFLVFLLS-----FTATVDA---------------RFDPTSFIAQVVS
MtBBI-2         --MELNKALLNVASLLFLLN-----FTATVDA---------------RFDPISLNTQVLS
GmBBI-7         --MEMKKLVLVKVALLFLFIC----FTASNVD-------------------ARSRSINPG
MtBBI-6         ---------MKVVLLLFILG-----FATTIDA---------------HFDPSSLVTQVLP
GmBBI-6         ----MGLSMKVIASILLLLLG----FTAITVS----------------------------
MtBBI-8         --------MMKLALLVFLLG-----FTSTVVDA--------------RFDPTSFITQVIS
GmBBI-12        MMELKKVALVKVALLLFLLG-----FSATTAD-------------ATRFDPRSFITQVLP
MtBBI-11b       MNKNKKAMVMKLALLVFLLG-----FTSTVVDA--------------RFDSTSFITQVIS
GmBBI-10        -MELSMKVLVKVASLLFLLG-----FTATVVD--------------ARFDPSSFITQFLP
GmBBI-11        -MELSMKVLVKVASLLFLLE-----FTATVVD----------------ARFDPSSFITQF
MtBBI-3         LMMNKKAMVMKLALLVFLLG-----FTSTVVD--------------ARFDGSSFITQLLS
MtBBI-11a       M-MNKKAMMMKLALLVFLLS-----FTSTMVDA--------------RFDRASFITQLLS
MtBBI-10        -MELNKKTMMKLALLVFLLS-----FTSTMV-----------------------------
OsBBI-9         MRSSSALFLAFVLLAVFLAAL----PFAESSGRHH----------HHHHSHLHGRGEGER
SbBBI-1         ------------------------------------------------MEDGPGQGGG
ZmBBI-4         ADPQSIQDLFSVFSALVLVGK----FKICSFLPCYQHAWVVQFGSWVPNRPLVSGQGAGG
SbBBI-3         --MRPQVVLLVSLAVFVVLVA----LPLGKAN---------------------------E
SbBBI-2         ---MRLQVLLVTLGVLAVLAA----LP--------------------------------L
SbBBI-4         ----MRSQVILVVVTLALLGV----LAALPLG----------------------------
SbBBI-7         ----MRPLVILAAVTLVALGV----LATLPLG----------------------------
SbBBI-5         -----MRPQLILVVTLALLGV----LAALPLG----------------------------
SbBBI-6         -----MRPQLILVVTLALLGV----LAALPLG----------------------------
ZmBBI-2a        KLQALSKMISAKAAGVQSTGA----AEASTAGAAVTGFLDKVVLFTVCFGATATDTATDG
ZmBBI-3a        AAGSGMGTSPVIPATMLFVAL----LVAVCFGA-------------TAARSSHARHTDAD
ZmBBI-1a        MGAASILLMMVSLETLLLAAG-------AGAGGTIRLSSDVGVYILRAGVAADLVTAMAR
OsBBI-1a        --MNSGNMATSSILFFFLLGG----LTVAVAA----------------HGTANDDTNTIR
OsBBI-2a        --MKMKRTMAATSILFFFLAG----LAAAHGS--------------TADDTTTTTNTIR
OsBBI-7b        ------------------------------------------------------------
OsBBI-8b        ------------------------------------------------------------
OsBBI-3a        ---MKTAMTTSTL-LFLLLAG----LTAAALG--------------TADDDTTTNTIRLP
OsBBI-6b        ------------------------------------------------------------
OsBBI-5a        KVKSTMMAASMLLFLLVLAGI----VTATTTT-------------------TDSNIRLPS
OsBBI-8a        -MSNNSMATSTILLFLLAVGG----LAAAHGDTIR-------------------------
OsBBI-6a        ---MSNTTMATSTILLFLLAG----LAAAHGD---------------------GDTTIRL
OsBBI-7a        ---MSNTTMAISTILLFLLAG----LVAAHGD----------------------GDTMIR
BdBBI-3         ---MKRGIAFILVLTLSLAGG-------------------------RLSAAAVADVAII
BdBBI-5         ---MKRGIAFILVLTLSLAGGRLSAAAVADVA------------------IIRLPAHAGG
ZmBBI-1b        ---------------------------------------------------------MAD
BdBBI-1         MKGSCINTIQVVLGAMLLAGAGAPSMAVPVDTAGVPVTQLPTGYRGKGSAVIQDQTTTLR
ZmBBI-2b        ------------------------------------------------------------
ZmBBI-3b        ------------------------------------------------------------
BdBBI-2a        MEGVRVATVLLMLSLVAAACC----LSAAAAD---------------------VDTIRLP
BdBBI-4a        ------------------------------------------------------------
BdBBI-2b        ------------------------------------------------DKVFADEATIVA
OsBBI-4b        ------------------------------------------------------------
BdBBI-4b        ---------------------------------------------------------DDN
OsBBI-4a        --MKSGSLLTLTLGALLLAGI----PISSAAV-----------------------TPNSN
BdBBI-6b        -----------------------------------------------------EEEEEEA
BdBBI-6a        STAAAIGLLMLSLPAALLVAG----MAADKED---------------TIRLPSDSVVEAE
OsBBI-3b        ------------------------------------------------------------
OsBBI-8c        ------------------------------------------------------------
OsBBI-2b        ------------------------------------------------------------
OsBBI-6c        ------------------------------------------------------------
OsBBI-7c        ------------------------------------------------------------
OsBBI-1b        ------------------------------------------------------------
OsBBI-5b        ------------------------------------------------------------


MtBBI-9         DAGLDPTTFIT--QEQNNEGCCNDC--EC--SS--LT-FPP-CRCNDATG-RSCHPGCDN
GmBBI-2         MKSDHHQHSND--DDSSK-PCCDQC--AC---T--KS-NPPQCRCSDMRL-NSCHSACKS
GmBBI-4         MKSDHHQHSND--DESSK-PCCDQC--AC---T--KS-NPPQCRCSDMRL-NSCHSACKS
GmBBI-9         FKSDQSSSYDD--DEYSK-PCCDLC--MC---T--RS-MPPQCSCEDIRL-NSCHSDCKS
GmBBI-5         NLFKSDNSSSD--DESSK-PCCDLC--MC---T--AS-MPPQCHCADIRL-NSCHSACDR
GmBBI-1         NLFKSDHSSSD--DESSK-PCCDLC--MC---T--AS-MPPQCHCADIRL-NSCHSACDR
GmBBI-3         NLFKSDHSSSD--DESSK-PCCDLC--MC---T--AS-MPPQCHCADIRL-NSCHSACDR
MtBBI-12        NGEDTYDVIYHS-------ACCDKC--ECYVKE--GH-FFPQCICRDDG----CHSACKK
MtBBI-1         SFITQVISSND--VNSTTNRCCKNVEWFY---V--EW-IPIMCQCLDIVE-T-CESTCKY
GmBBI-8         QIEGDSDDNYV--KSNTE-PCCDNC--LC---T--NS-IPPKCQCTDWAE--DCHSACKG
MtBBI-5         ------------TTKST-GCCDNC--TC---T--RS-LPPDCTCDDVKP--FCHSDCKS
MtBBI-7         NGV----------KSTCK-PCCNNC--SC---T--FS-IPPQCRCKDVKP--TCHSACKT
MtBBI-4         NGDATNYTV----KFTAI-TCCNKC--IC---T--KT-IPSMCRCVDVVE--TCRSGCRS
MtBBI-2         NVKLITT------------SCCDNC--HC---T--TT-IPPLCRCADIVK-NKCNSACKL
GmBBI-7         SFIIAGDNYNL--KSTTS-ACCDAC--AC---T--KS-IPPICHCHDFGE--TCHSACNL
MtBBI-6         NGDASYYVKKS--TTTAT-ACCDKC--YC---T--KS-RPPQCHCADLNK--TCNSACKL
GmBBI-6         ---GDANNYYI--KSTTK-ACCDKC--YC---S--KS-IPPKCYCADVGI--TCHSACKV
MtBBI-8         NGEA---AYDV--KSTTT-GCCNSC--QC---T--RS-FPQAC-CLD------------
GmBBI-12        NNYYVKI------KSTAT-ACCDLC--LC---T--KS-YPPQCNCVDESE-TGCHSCCKN
MtBBI-11b       NGDSTTNNYDA--KSTAT-ACCNTC--LC---S--VTPELTQCRCADVGK--TCHSACKN
GmBBI-10        NAEANNYYV----KSTTN-GCCDNC--RC---T--IS-ISPMCKCADIGE--TCHPSCKS
GmBBI-11        LPNAEANNYYV--KSTTK-ACCNSC--PC---T--KS-IPPQCRCSDIGE--TCHSACKT
MtBBI-3         NGEATYEV-----KSTTT-ACCNSC--PC---T--KS-IPPQCHCADIGE--KCHSACKR
```

```
MtBBI-11a       NGEA-----NT--KSTTT-ACCDFC--PC---T--RS-IPPQCQCTDVKE--KCHSACKS
MtBBI-10        -------------DATTT-ACCDSC--PC---T--KS-IPPQCHCTDIGE--TCHSACKS
OsBBI-9         GGEARSLAAKG--AAAAW-PCCDNC-GGC---T--KS-IPPQCQCMDARP-AGCHPACKS
SbBBI-1         GGGELASRGKA--AARAW-PCCDSC-GGC---T--KS-EPRRCQCLDAVP-RGCHPACRD
ZmBBI-4         GGGELASRAKA--AARAW-PCCDSC-GGC---T--RS-EPPRCQCLDAAP-RGCHPACRD
SbBBI-3         EKEAAAEGVDA--RRWRW-PCCDEC-GVC---T--RS-QPPICQCLDTST-SGCNPGCKA
SbBBI-2         SKGEEEGGAAR--AKGSW-PCCDKC-GFC---Y--RS-FPPRCQCLDFSQ-RGCHPACRS
SbBBI-4         -------------EGSSW-PCCDNC-GIC---N--RM-IPPKCRCNDISP-HGCHPKCNK
SbBBI-7         -------------EGSSW-PCCDNC-GVC---N--RK-LPPDCKCNDVSV-QGCHPECKN
SbBBI-5         -------------KGSSW-PCCDNC-GFC---N--RK-LPPDCQCNDVSV-DGCHPECKN
SbBBI-6         -------------KGSSW-PCCDNC-GFC---D--RQ-LPPDCQCDDVSV-DGCHPECKN
ZmBBI-2a        AIRLLPGSSGA--VGRPW-ECCDYV--TK---DPIIS-RPPRWRCNDVVD--KCSADCQE
ZmBBI-3a        DAIRLPSSFGA--GGRPW-NCCDSA--IK---D--AS-LPA-WRCNDVVD--KCSADCQD
ZmBBI-1a        ARARAKHQLRD--EERPWGECCDLA--VC---V--KT-YPLTCSCFDRVE--RCSDACKE
OsBBI-1a        LPSDGAKSPKMPTEKRPW-KCCDDI--EE---QPASI-FPPFWRCNDELEPSQCAAQCEV
OsBBI-2a        LPIDGAVAARR--RTRPW-KCCDNIVRLP---E--RI-NPPFWQCDDELEPGQCFRQCEA
OsBBI-7b        --------QPT--TKRPW-KCCDSIVQLP---Q--RI-FPPFWRCDDELEPGKCTAACKS
OsBBI-8b        ----EWPERTT--KKRPW-KCCDNIRRLP---P--RI-HPPFWRCDDELKPGQCFAACKA
OsBBI-3a        TDGGSAQQAPT--KKKPW-KCCDNIERLP---T--KT-NPPQWRCNDELEPSKCVAQCEV
OsBBI-6b        -------DGLS--MERPW-KCCDNIKRLP---T--KP-DPPQWRCNDELEPSQCTAACKS
OsBBI-5a        NGAAGETDGNKQAKSRPW-ECCDNI--EM---SVLKI-YPPRWRCNDEVK--QCAAACEN
OsBBI-8a        -LPSEGDAPPQ--PAKPW-DCCDDI--EM---SPLKI-FPPLYRCNDEVK--QCSAACKE
OsBBI-6a        PSDGAKASRPR--AAKPW-DCCDNI--EI---SRLMI-YPPLYRCNDEVK--QCAAACKE
OsBBI-7a        LPSDGAEAPPR--PPKPW-DCCDNI--EM---SPLEI-FPPLYRCNDEVK--QCSAACKE
BdBBI-3         RLPAHAGVASEGEEEKPW-KCCDLT--IC---A--RS-FPPTCRCADVVK--QCADGCKS
BdBBI-5         MDAAGVASEGE--EEKPW-KCCDLT--IC---A--RS-FPPTCRCADVVK--QCADGCKS
ZmBBI-1b        DKKAGGGDDDK--KKRPW-KCCDVP--IC---T--RS-WPPVCRCADTVE--RCASTCQH
BdBBI-1         ASSGEEDEYRE--SSRPW-SCCDRV--VN---P--RGVYPPILRCSDEVE--RCAGACAE
ZmBBI-2b        -----------------W-DCCDFA--VC---T--RE-YIPNCRCGDVGE--SCPSNCKV
ZmBBI-3b        -----------------W-DCCDSV--AC---T--RD-YIPNCMCLDKVE--SCSSNCER
BdBBI-2a        SQDEVANQAGA--EKRPW-KCCDRA--MC---T--RS-IPPFCKCLDLLD--RC-PGCKD
BdBBI-4a        -----MAKASA--EKRPW-KCCNRA--RC---T--RS-IPPFCWCHDVVE--RCSGACKD
BdBBI-2b        AEEKKAAAAEA--EKRPW-KCCDDT--LC---N--RA-APPTCRCLDTVD--RCAASCRE
OsBBI-4b        ------GEEAV--RSRPW-KCCDDA--VC---T--RS-MPPTCSCQDKVK--SCSGGCGK
BdBBI-4b        KAVAADETAAK--KKRPW-KCCDRT--LC---T--KS-APPTCSCLDKVE--RCSGRCKR
OsBBI-4a        IRLPTDGGDEW--PSPPW-DCCDKL--KQ---SPLRI-WPPKYKCLDEVD--HCAAACED
BdBBI-6b        AIPIITEPPPV--PERPW-ECCDAT--VC---T--KS-FPPTCRCLDVVD--RCAAACDR
BdBBI-6a        GLVIGQTAMAA--ATRPW-DCCDTT--LC---T--KS-FPPICRCLDVVP--RCAAACES
OsBBI-3b        -----------------WGDCCTNT--TC---T--RS-IPPICRCNDKVK--KCAAACKD
OsBBI-8c        -----------------WGDCCDNT--TC---T--KS-IPPICSCGDKVA--ACDGACKD
OsBBI-2b        --------------EWPWGPCCDIA--VC---T--KS-LPPICHCSDEVE--SCAAACGQ
OsBBI-6c        -----------------WGDCCDKA--FC---N--KM-NPPTCRCMDEVK--ECADACKD
OsBBI-7c        -----------------WGKCCDKA--FC---N--KM-NPPTCRCMDEVN--KCAAACKD
OsBBI-1b        -----------------WGDCCDMD--IC---S--RS-LPPICRCADEVE--SCAAACKD
OsBBI-5b        -----------------WGDCCDKA--FC---T--RS-LPPICHCADEVA--SCAAACKE
                                    **                    * *

MtBBI-9         CVC--D--------SGLP----PTCRCED-----------YIYS----------------
GmBBI-2         CIC--A--------LSYP----AQCFCVD-----------I--TDFCYEP----------
GmBBI-4         CIC--A--------LSYP----AQCFCVD-----------I--TDFCYEP----------
GmBBI-9         CMC--T--------RSQP----GQCRCLD-----------T--NDFCYKP----------
GmBBI-5         CAC--T--------RSMP----GQCRCLD-----------T--TDFCYKP----------
GmBBI-1         CAC--T--------RSMP----GQCRCLD-----------T--TDFCYKP----------
GmBBI-3         CAC--T--------RSMP----GQCRCLD-----------T--TDFCYKP----------
MtBBI-12        CDCIRD-------SESET----PSCYCAD-----------VMFF-TCYDL----------
MtBBI-1         CTC------------KQP----KRCTCDD-----------YEQD----------------
GmBBI-8         CIC--Q--------RIWP----PRCRCFD-----------E--TDTCYDK----------
MtBBI-5         CRC--F--------ESLP----LKCTCLD-----------I--TEFCYEP----------
MtBBI-7         CRC--F--------ESFP----LKCDCLD-----------I--TDFCYEP----------
MtBBI-4         CIC--P--------GSSP----PQCYCDD-----------LFQSRFCHKK----------
MtBBI-2         CVC--K--------STIP----PQCRCMD-----------H--TNFCYEP----------
GmBBI-7         CIC--T--------ASYP----PQCRCLD-----------Q--TTFCYDK----------
MtBBI-6         CAC--L--------PSPP----VLCRCVD-----------I--TNFCYPP----------
GmBBI-6         CLC------------IH----PQCRCVD-----------T--TDFCYEP----------
MtBBI-8         CVC--T--------KFIP----PQCRCHDVTTFWQRGTIGWFRFGFVTEKSRHDPGGTWH
GmBBI-12        CIC--N--------KKFP----RTCYCSD-----------I--TNFCYDK----------
MtBBI-11b       CEC-----------GWSS----PLCTCYD-----------I--TDFCYKP----------
GmBBI-10        CFC--D-------IPTFP----GLCQCID-----------V--TNFCYEL----------
GmBBI-11        CIC--T--------RSIP----PQCHCSD-----------I--TNFCYEP----------
MtBBI-3         CLC--T--------RSFP----PQCRCTD-----------T--TDFCYEP----------
MtBBI-11a       CLC--T--------RSFP----PQCRCYD-----------I--TNFCYPS----------
MtBBI-10        CLC--T--------KSIP----PQCHCAD-----------I--TDFCYPK----------
OsBBI-9         CVKSSL--------SVSP----PVYQCMD-----------RIPN-LCQRR----------
SbBBI-1         CVKSSL--------SADP----PVYQCMD-----------RVPN-FCQRR----------
ZmBBI-4         CVKSSL--------SADP----PVYQCMD-----------RVPN-FCQRR----------
SbBBI-3         CVK-----------SISD----GLYECKD-----------RIVN-FCKRR----------
SbBBI-2         CLKFTT------GGIDEP----PIFRCAD-----------ILVN-FCDRS----------
SbBBI-4         CVT-NTLTAAGDDDGTGP---VRAYYCAD-----------MITN-FCKRR----------
SbBBI-7         CVK--VGAGIRPGGGHGP---VVTYRCDD-----------ILTN-FCEHR----------
SbBBI-5         CVK--VGAGIRPGGGHGP---VVTYRCDD-----------ILTN-FCEQR----------
SbBBI-6         CVM----------VGSGP---VVTYRCDD-----------VLTN-FCEQR----------
ZmBBI-2a        CEE-----------SLAG----DGFVCDD-----------WIFSLLEPPV----------
ZmBBI-3a        CEA-----------SPAG----DGFVCGD-----------FILSLTEPPV----------
```

```
ZmBBI-1a        CVE------------TED----SRHVCVD-----------RYRG-DPGPR----------
OsBBI-1a        CQD--Q--------EASP----GRLICGD-----------VYWGADPGPF----------
OsBBI-2a        CRDPPG--------RPFP---GRPLICDD-----------VFWGDDPGTS----------
OsBBI-7b        CRE-AP--------GPFP----GPLICED-----------VYWGADPGPL----------
OsBBI-8b        CRE-AP--------GPFP----GPLICDD-----------VYWGADPGPF----------
OsBBI-3a        CQE-AP--------GPFP----GPLICSD-----------VYWGADPGPF----------
OsBBI-6b        CRE-AP--------GPFP----GKLICED-----------IYWGADPGPF----------
OsBBI-5a        CLQLVP------GAGGED----VVFVCDD-----------WYPTTNPGPV----------
OsBBI-8a        CVAAPA-------AGDSPCGGGAALVCRD-----------WYSTEDPGKP----------
OsBBI-6a        CVE-----------APGGDFNGGAFVCSD-----------WFSTVDPGPK----------
OsBBI-7a        CVE--A-------PGDFP---RGAFVCRD-----------WYSTVDPGHM----------
BdBBI-3         CMG-------------VP----FGHICND-----------WYTG-DPAQG----------
BdBBI-5         CMG-------------VP----FGHICND-----------WYTG-DPAQG----------
ZmBBI-1b        CEQ--V-------EEGGP----RRYRCLD-----------THRG-DPGPR----------
BdBBI-1         CEE--V-------EESEP----RRYMCVD-----------WYRGDDPGPR----------
ZmBBI-2b        CEF--V--------ESDP----PGYRCLD-----------VFHG-YPGPR----------
ZmBBI-3b        CEF--L-------YHSDP----PRFQCRD-----------IFHG-YPGPK----------
BdBBI-2a        CRAERE-------HEADP----ESFVCHD-----------VYRG-APGPN----------
BdBBI-4a        CRE--A----EREDESDP----EGYVCHD-----------MYHG-SPGPA----------
BdBBI-2b        CVA----------AGSDP----SRRVCKD-----------RFHG-WPGPK----------
OsBBI-4b        CVQ----------VESQP----PRFRCLD-----------RYHG-FPGPK----------
BdBBI-4b        CGP----------SESDP----SGLFCLD-----------RYHG-WPGPK----------
OsBBI-4a        CKR------------ADG----GGYVCRD-----------WYWGVNPGPK----------
BdBBI-6b        CEP----------AEDDP----ARRVCKD-----------QYFG-DPGPT----------
BdBBI-6a        CDP----------SESDP----SGCVCND-----------WHRG-DPGPR----------
OsBBI-3b        CKR--V-------KSSKP----PRYVCQD-----------QFTG-QPGPK----------
OsBBI-8c        CQP--V------ASSSEP----PRFVCKD-----------QFTG-QPGPK----------
OsBBI-2b        CEM--V------DSWSWR----PLFVCRD-----------SFTG-DPGPR----------
OsBBI-6c        CQR--V-------ESSEP----PRYVCKD-----------RFTG-HPGPV----------
OsBBI-7c        CQR--V-------ESSEP----PRYVCKD-----------RFTG-QPGPM----------
OsBBI-1b        CQQ--LE---SSSSSSEP----PRYVCHD-----------WFRG-EPGPS----------
OsBBI-5b        CDM--V------NSSSEP----PRYICRD-----------HFTG-EPGPM----------
                *                         * *

MtBBI-9         ----------CNKCTNSNQ--------------------------------
GmBBI-2         ----------CKPSEDDKENY------------------------------
GmBBI-4         ----------CKPSEDDKENY------------------------------
GmBBI-9         ----------CKSRDD-----------------------------------
GmBBI-5         ----------CKSSDEDDD--------------------------------
GmBBI-1         ----------CKSSDEDDD--------------------------------
GmBBI-3         ----------CKSSDEDDD--------------------------------
MtBBI-12        ----------CKFKL------------------------------------
MtBBI-1         ----------CNEC-------------------------------------
GmBBI-8         ----------CTSTSQETAKAHDRYN-------------------------
MtBBI-5         ----------CNNSTIIAN--------------------------------
MtBBI-7         ----------CNSTIAKSGCTCTHILRDGNRAVDALAKNG-----------
MtBBI-4         ----------CN---------------------------------------
MtBBI-2         ----------CNVIPMKSQLEGH----------------------------
GmBBI-7         ----------CDSSEDKAHSE------------------------------
MtBBI-6         ----------CN---------------------------------------
GmBBI-6         ----------CNH--------------------------------------
MtBBI-8         DVVDKVYYCTIPAPSWHDWVETWHDAAY-----------------------
GmBBI-12        ----------CNSTEAN----------------------------------
MtBBI-11b       ----------CN---------------------------------------
GmBBI-10        ----------CNSSETKAH--------------------------------
GmBBI-11        ----------CNSSETEAH--------------------------------
MtBBI-3         ----------CSYSSNGGSH-------------------------------
MtBBI-11a       ----------CTMSPHKEIKERYKEIVT-----------------------
MtBBI-10        ----------CN---------------------------------------
OsBBI-9         ----------CTAAAR-----------------------------------
SbBBI-1         ----------CTAPAAAH---------------------------------
ZmBBI-4         ----------CTAAAAAAAH-------------------------------
SbBBI-3         ----------CTRPPAAAAAADA----------------------------
SbBBI-2         ----------CTPPEAL----------------------------------
SbBBI-4         ----------CSPAPAAAFLGEVF---------------------------
SbBBI-7         ----------CTAAPPPEAAAFLGDGF------------------------
SbBBI-5         ----------CTPAPAPVPEAGFLGGGF-----------------------
SbBBI-6         ----------CTPAPAPAPAPEAAFLGRGF---------------------
ZmBBI-2a        ----------CTPRP------------------------------------
ZmBBI-3a        ----------CTPRP------------------------------------
ZmBBI-1a        ----------CHDEDGRSGGPADDDAGGQATPTAV----------------
OsBBI-1a        ----------CTPRP------------------------------------
OsBBI-2a        ----------CAPSS------------------------------------
OsBBI-7b        ----------CTPRP------------------------------------
OsBBI-8b        ----------CTPRP------------------------------------
OsBBI-3a        ----------CTPRP------------------------------------
OsBBI-6b        ----------CTPRP------------------------------------
OsBBI-5a        ----------CTPRP------------------------------------
OsBBI-8a        ----------CTPER------------------------------------
OsBBI-6a        ----------CTAAL------------------------------------
OsBBI-7a        ----------CTAPD------------------------------------
BdBBI-3         ----------ATNGN------------------------------------
BdBBI-5         ----------ATNGN------------------------------------
```

```
ZmBBI-1b        ----------CGDGEDDGGEWSPTPRLARGHRF------------------
BdBBI-1         ----------CTGVHHL----------------------------------
ZmBBI-2b        ----------CTPFGSVKAISSAQLLAST----------------------
ZmBBI-3b        ----------CRTWISTSN--------------------------------
BdBBI-2a        ----------CSTNG------------------------------------
BdBBI-4a        ----------CSSGT------------------------------------
BdBBI-2b        ----------CSNNQ------------------------------------
OsBBI-4b        ----------CHNQPA-----------------------------------
BdBBI-4b        ----------CTNKNDGL---------------------------------
OsBBI-4a        ----------CTGGG------------------------------------
BdBBI-6b        ----------CKSKHHDGPPAGGGSPSLAAVGGATTGLLMAFSTVLLFTQT
BdBBI-6a        ----------CPHHD------------------------------------
OsBBI-3b        ----------CKHSCEN----------------------------------
OsBBI-8c        ----------CTPCTQN----------------------------------
OsBBI-2b        ----------CTPEMHN----------------------------------
OsBBI-6c        ----------CKPRAEN----------------------------------
OsBBI-7c        ----------CKPRAEN----------------------------------
OsBBI-1b        ----------CTPDEHK----------------------------------
OsBBI-5b        ----------CA---------------------------------------
```

F. I13 Pin-I.

```
PpPINI-1        ---------MSRAALIGQSYVWYLNRLANSMHTAQEKMRARLSLTCECVQRRTEWPELLH
CsPINI-1        MPGAPQQANAGLDTTTPSAGTGQSGLLALDGTGSGGGVYSGGDSISGQGASPVGGGSSAG
PpPINI-2        ------------------------------------------------------------
AtPINI-2        ------------------------------------------------------------
OsPINI-2        ------------------------------------------------------------
OsPINI-14       ------------------------------------------------------------
OsPINI-1        ------------------------------------------------------------
SbPINI-22       ------------------------------------------------------------
SbPINI-21       ------------------------------------------------------------
SbPINI-23       ------------------------------------------------------------
SbPINI-3        ------------------------------------------------------------
VvPINI-6        ------------------------------------------------------MADENQ
OsPINI-7        ------------------------------------------------------------
SbPINI-17       ------------------------------------------------------------
OsPINI-13       ------------------------------------------------------------
OsPINI-8        ------------------------------------------------------------
SbPINI-18       ------------------------------------------------------------
SbPINI-19       ------------------------------------------------------------
SbPINI-20       ------------------------------------------------------------
OsPINI-3        ------------------------------------------------------------
OsPINI-4        ------------------------------------------------------------
SbPINI-1        ------------------------------------------------------------
SbPINI-2        ------------------------------------------------------------
SbPINI-5        ------------------------------------------------------------
SbPINI-4        ------------------------------------------------------------
OsPINI-6        ------------------------------------------------------------
OsPINI-5        ------------------------------------------------------------
OsPINI-15       ------------------------------------------------------------
SbPINI-6        ------------------------------------------------------------
SbPINI-15       ------------------------------------------------------------
SbPINI-16       ------------------------------------------------------------
OsPINI-9        ------------------------------------------------------------
OsPINI-11       ------------------------------------------------------------
OsPINI-12       ------------------------------------------------------------
OsPINI-10       ------------------------------------------------------------
SbPINI-11       ------------------------------------------------------------
SbPINI-12       ------------------------------------------------------------
SbPINI-10       ------------------------------------------------------------
SbPINI-9        ------------------------------------------------------------
SbPINI-7        ------------------------------------------------------------
SbPINI-8        ------------------------------------------------------------
SbPINI-14       ------------------------------------------------------------
SbPINI-13       ------------------------------------------------------------
SmPINI-1        ------------------------------------------------------------
MtPINI-1        ------------------------------------------------------------
MtPINI-2        ------------------------------------------------------------
MtPINI-3        ------------------------------------------------------------
MtPINI-4        ------------------------------------------------------------
AtPINI-1        ------------------------------------------------------------
VvPINI-5        ------------------------------------------------------------
AtPINI-4        ------------------------------------------------------------
AtPINI-6        ------------------------------------------------------------
AtPINI-5        ------------------------------------------------------------
AtPINI-3        ------------------------------------------------------------
FvPINI-1        ------------------------------------------------------------
FvPINI-2        ------MRCAGKDFWPELLGAKAREARSKIESENDLVKVVIVKEGTFVTHDYRCDRVRVW
MtPINI-6        ------------------------------------------------------------
VvPINI-3        ------------------------------------------------------------
FvPINI-3        ------------------------------------------------------------
FvPINI-4        ------------------------------------------------------------
FvPINI-5        ------------------------------------------------------------
MtPINI-8        ------------------------------------------------------------
MtPINI-9        ------------------------------------------------------------
MtPINI-5        ------------------------------------------------------------
MtPINI-7        ------------------------------------------------------------
VvPINI-4        ------------------------------------------------------------
VvPINI-1        ----------------------------------------------MRVTSLVTIDLLYS
VvPINI-2        ------------------------------------------------------------


PpPINI-1        KNMSAVWALFWNTLDEADPDFSMFLQRPLPSEQNRPQHDE--ESDV--------WFYLTR
CsPINI-1        VGDVSGSTSFGNPGGLVFDGAGNIVSGVQSAFGDVPSTYT--GRQE--------W-----
PpPINI-2        ----------------MRTVACLIVVVVLMASLSGGNANH--GRIGIHSALDGRW-----
AtPINI-2        ------------MNRNCPLYSPPCQRCSCAGITCQPLFPG--IKVD--------W-----
OsPINI-2        ---------------------------MSWACRSLPNCPN--GKSS--------W-----
OsPINI-14       ----------------------MPAAGGDPQVAAAEQLRR--LKSS--------W-----
OsPINI-1        ----------------------------MGRAAAAGDGDS--LKTA--------W-----
SbPINI-22       ----------------------MGRFLTPATTAAANDDGD--LKTS--------W-----
SbPINI-21       ------------------------------MPAAGGGEDG--QKSS--------W-----
SbPINI-23       ------------------------MGRAAAAASAGGGGDE--QKTS--------W-----
```

```
SbPINI-3        ----------------------------------MAGADP--KRTS--------W-----
VvPINI-6        RTELPQERPQQSTTIPQEQPHQSTILLPGSVGHPSGVEVA--PKTT--------W-----
OsPINI-7        -------------------------MGRAAAAVGGGEIVS--IKTA--------W-----
SbPINI-17       ------------------------------------MSQN--TKTS--------W-----
OsPINI-13       -------------------------------------MSS--DKSS--------W-----
OsPINI-8        -------------------------------------MEK--KKVR--------W-----
SbPINI-18       ---------------------------MTKTFAPPKDHGE--KKES--------W-----
SbPINI-19       --------------------------MTKTFAPPKDHGEK--TKES--------W-----
SbPINI-20       ---------LELTSDSRSSSSRPAKAKTMTKTFAPPKDHG--EKES--------W-----
OsPINI-3        --------------------------------------MS--QKSS--------W-----
OsPINI-4        --------------------------------------MS--QKSS--------W-----
SbPINI-1        --------------------------------------IT--GKSS--------W-----
SbPINI-2        ----------------------------------MSVPIP--GKSS--------W-----
SbPINI-5        -------------------------------------------RMS--------W-----
SbPINI-4        -------------------------------------MSS--QKTS--------W-----
OsPINI-6        ------------------------------------MSSE--KKAS--------W-----
OsPINI-5        -----------------------------MSSSDSKPCGG--LKTE--------W-----
OsPINI-15       -----------------------MSSGGKQDGAAAAGEEE--RKTS--------W-----
SbPINI-6        -----------------------MATSGPNADGGAAAAAA--PKNS--------W-----
SbPINI-15       -----------------------------QKKSCSFEETQ--KKTC--------W-----
SbPINI-16       ------------------------------------------GVWS--------W-----
OsPINI-9        ------------------------MAGVVRSAACSGGEGE--RKTS--------W-----
OsPINI-11       ----------------MTNLVFDRVRRCEVVGEMSSGGEE--GKTS--------W-----
OsPINI-12       -------------------------------MGGGVRSAA--AKRS--------W-----
OsPINI-10       ------------MNSTSHFVAISFFHHQLDQTRRGKMSSS--AKTS--------W-----
SbPINI-11       -----------------------------------MAGGT--EKTS--------W-----
SbPINI-12       ----------------------------MSSSAEAERGGA--KKQS--------W-----
SbPINI-10       -------------------------MSSTAAVTTAPGSGG--AKTS--------W-----
SbPINI-9        ----------------------------MSSTATAADCGG--AKTS--------W-----
SbPINI-7        ----------------------------MSSTAAAPESGG--AKTS--------W-----
SbPINI-8        ---------------------------MSSTATAAPECGG--AKTS--------W-----
SbPINI-14       ----------------------------MSSIVMGATGDK--NKTS--------W-----
SbPINI-13       ----------------------------MSSIVMDVTSGE--KKTS--------W-----
SmPINI-1        -------------------------------------------KTA--------W-----
MtPINI-1        ------MTEEKQGQGTNPPHEQPNESLLRTYNQRLRRNNP--TKTS--------W-----
MtPINI-2        ----------MAESFKFICVIIIFLCSFIAAKNIDGRNNP--TRRN--------W-----
MtPINI-3        ------MAEEQQGQGTNPPQEQPNEPLPRTYNQLLGTNNP--TKTS--------W-----
MtPINI-4        ------------------MAEEKSGQGTNPPQEQPRRNNP--TRRN--------W-----
AtPINI-1        ---------------------MSRYPPCWSGSCEDPECCAIGKKYR--------W-----
VvPINI-5        ---------------------------------MSGPHCF--GKQA--------W-----
AtPINI-4        ----------------------------MSDVCQNTAGEG--MKSS--------W-----
AtPINI-6        ----------------MATEWCSYIGSSFILNFYFSFFDI--GKNS--------W-----
AtPINI-5        ----------------------------------MSTECP--RKNS--------W-----
AtPINI-3        -----MFVIVEKNPIQNSNHKNLQINTFIQEKMASICEDP--GKSS--------W-----
FvPINI-1        -------------------------------MATSSTSTE--TKCS--------W-----
FvPINI-2        VDKHGTVTRVPTLSQTYYKRFITESKTQDLKNAKMAGQCV--GKED--------W-----
MtPINI-6        -----------------------------------MSSCK--GKKT--------W-----
VvPINI-3        ----------------------------------MTTCPD--GKSS--------W-----
FvPINI-3        ----------------------------------MSDQCQSEGKSS--------W-----
FvPINI-4        ----------------------------------MSDQCE--GKDS--------W-----
FvPINI-5        ----------------------------------MAGQSV--GKES--------W-----
MtPINI-8        ---------------------------------MSDDECK--GKSS--------W-----
MtPINI-9        --------------------------------MSYDDECK--GKSS--------W-----
MtPINI-5        ----------------------------------MSDECK--GKNS--------W-----
MtPINI-7        ----------------------------------MSDECQ--GKSS--------W-----
VvPINI-4        ----------------------------------MAFECR--GKTS--------W-----
VvPINI-1        TVHMLDSDFGSDFHMYLEYQEALVDRYLCQTRKRMASECE--GKSS--------W-----
VvPINI-2        ----------------------------------MASECE--GKSS--------W-----
                                                                      *

PpPINI-1        DDIIYEIPRHGRWHPNRVCPGWPELKGVHYRDAVKIIKDDMPGVLVD-YG--PLRRR-K-
CsPINI-1        ----------------------PELVSTNALVAKAKLQGE-TGLNVL-LV--PKGSV-V-
PpPINI-2        ----------------------PDLVGRDAEEAKTHILSERPYLNVR-IV--PTDMM-V-
AtPINI-2        ----------------------PELNGVKGLEAKRIIEHDNPKVVVV-II--PDDVA-V-
OsPINI-2        ----------------------SELVGKKGSEAMAVILRERPDITRAILV--PQDAV-I-
OsPINI-14       ----------------------PELVGWPEFYAALKIIDERPDVTVY-MF--RDGDD-LP
OsPINI-1        ----------------------PELVGWVELNAAFQINRDRPDVHVA-FY--MVGTQ-L-
SbPINI-22       ----------------------PEVVGWVTLNASFKITADRPDVSTA-FY--SDTTP-L-
SbPINI-21       ----------------------PEVVGWVMLNASDKITRDRPDVSIA-FY--SQTTT-L-
SbPINI-23       ----------------------PEVVGWVTLNAAFKINSDRPDVSTA-FY--SETTP-L-
SbPINI-3        ----------------------PELVGIPALAAVMRINHDRPELVVE-VL--PLDMK-L-
VvPINI-6        ----------------------PEVVGMTVEEAERKIREDMPRVQFQ-VV--PPNCF-V-
OsPINI-7        ----------------------PEVVGWDGMSAVMKIHRDRADVRVE-VH--TVGES-V-
SbPINI-17       ----------------------PEVKGLPAEVAKHKIQDDRPDVEVI-LV--RVGSV-V-
OsPINI-13       ----------------------PEVVGLPAEAAKHIILNDRPDVHVV-VL--RVGSV-V-
OsPINI-8        ----------------------AEVLGELAPLAVTQISNDRPDVAVE-VLLRDAAAV-V-
SbPINI-18       ----------------------PEVKGWPATQAAQTIAHERPDVAVE-VL--PPGSY-V-
SbPINI-19       ----------------------PEVKGWPATQAAQTIAHERPDVAVE-VL--PPGSY-V-
SbPINI-20       ----------------------PEVKGWPATQAAQTIAHERPDVAVE-VL--PPGSY-V-
OsPINI-3        ----------------------PELVGVLATLAATQIGKDRPDVAVE-VL--PPGAP-L-
OsPINI-4        ----------------------PELVGVLATLAATQIGKDRPDVAVE-VL--PPGAP-L-
SbPINI-1        ----------------------PELVGVQATLAATAIAHDRPDVSVE-VL--PPGSP-V-
SbPINI-2        ----------------------PELVGVNATLAATAIAHDRPDVAVE-VL--PPGAP-V-
```

```
SbPINI-5        ----------------------PEVVGWSAADAKAQIKSDRPDVTIE-VL--PWSTY-A-
SbPINI-4        ----------------------PEVVGWPATAAVTQISSDRPDVAIE-VV--QVGTN-V-
OsPINI-6        ----------------------PEVVGWPATAAVTQINGDRPDVSIE-VI--PDGAT-V-
OsPINI-5        ----------------------PELVGLTIEQAKAKIKADRPDLQVE-VL--PVGTI-I-
OsPINI-15       ----------------------PEVVGLPVEEAKKVILKDMPDADIV-VL--PAGSP-AG
SbPINI-6        ----------------------PELVGKSSEEAKKKIKEDKPGADVQ-VV--PADAF-V-
SbPINI-15       ----------------------PEFVGLSITQAVPSILKDMPNAEIE-VL--ALGSP-M-
SbPINI-16       ----------------------PNIIGFTMEDAMIIILRDKPDADIV-FL--PVGSP-VT
OsPINI-9        ----------------------PEVVGLSAEEAKKVILKDMPDADIV-VL--PAGSP-V-
OsPINI-11       ----------------------PEVVGLRAEEAKKVILKDMPDADIV-VV--PVGTP-V-
OsPINI-12       ----------------------PEVVGMTMEEAKAAILKDKPDADIV-VL--PVGAP-M-
OsPINI-10       ----------------------PEVVGLSIEEAKKVILKDKPDADIV-VL--PFGTA-V-
SbPINI-11       ----------------------PEVVGLSVEKAKKTILKDKPDADII-VL--PVGTP-V-
SbPINI-12       ----------------------PEVVGLSVEEAKKVILKDKPDADIF-VL--PVGSP-V-
SbPINI-10       ----------------------PEVVGLSIEEAKKVILKDKPDADIV-VL--PVGSK-V-
SbPINI-9        ----------------------PEVVGKSVEEAKKVILKDKPDADIV-VL--PVGTI-V-
SbPINI-7        ----------------------PELVGLNVEEAKKVILKDKPDADIV-VL--PVGSP-V-
SbPINI-8        ----------------------PEVVGKSVEEAKKVILKDKPDADIV-VL--PVGSP-V-
SbPINI-14       ----------------------PEVVGMSINEATDIILKDMLNAHIE-IL--PIGSI-V-
SbPINI-13       ----------------------PEVLGMSIKEATEIILKDMPNAYIQ-VL--PVGSP-V-
SmPINI-1        ----------------------PELVGKDAIYAREQILADNPKLEIH-IV--FYEACLV-
MtPINI-1        ----------------------PELVGVTVEEAKRKIKEEMSEVKIE-VV--SPGSC-V-
MtPINI-2        ----------------------SELVGVTAEEAERKIKEEMNGVEIR-VV--PPGYF-V-
MtPINI-3        ----------------------PELVGVTAEEAERKIKEDISGVEIQ-VV--PPDSF-V-
MtPINI-4        ----------------------SELVGVTAEEAERKIKEEMHGVEIR-VV--PPGY----
AtPINI-1        ----------------------PELVGKNGQLAKMTIERENPNVLAI-VL--RYGEK-R-
VvPINI-5        ----------------------PELLGEKAEVAKETIERENPSVRAR-FI--KQGHY-R-
AtPINI-4        ----------------------PELVGRRGEEVKEIIDRENTKVTAK-II--SENAV-V-
AtPINI-6        ----------------------PELLGTNGDYAASVIKGENSSLNVV-VV--SDGNY-V-
AtPINI-5        ----------------------PELTGTNGDYAAVVIERENPTVNAA-VI--LDGSP-V-
AtPINI-3        ----------------------PELLGAKGEDAKEVIERENPKMKAV-II--LDGTV-V-
FvPINI-1        ----------------------PELVGTKGEEAAATIVKENPSVKAH-TV--NEGSF-V-
FvPINI-2        ----------------------PELLGAEGTVAKETIERENSTVKAE-IV--LEGTI-V-
MtPINI-6        ----------------------PELVGVNGEAAAQIIMSENSLVTAS-TL--PEDSV-F-
VvPINI-3        ----------------------PELVGVHGEAAAAIIERENLNVKAG-VV--KEGTL-V-
FvPINI-3        ----------------------PELLGAQGTDAKATIESENSSVTAV-IV--QEGSI-V-
FvPINI-4        ----------------------PELLGAQGTVAKATIESENASVKAV-IV--LEGTS-V-
FvPINI-5        ----------------------PELLGAEGTVAKEIIESENASVTAV-IV--LEGTP-V-
MtPINI-8        ----------------------PELVGVEGKVAEATIQRENASVKAI-IV--LEGSS-V-
MtPINI-9        ----------------------PELVGVEGKVAEATIERENPLVNAI-IV--PEGSA-V-
MtPINI-5        ----------------------PELVGVEGKVAEATIQSENPLVNAI-IV--PEGSF-V-
MtPINI-7        ----------------------PELVGVEGKVAEATIQRENPLVDAI-IV--PEGSS-V-
VvPINI-4        ----------------------PELLGVQKAVAKATVERENPYVTDVEIV--LEGTI-V-
VvPINI-1        ----------------------PELVGVQGEVAAETIKRENPHITTVDIL--LEGTI-V-
VvPINI-2        ----------------------PELVGVQGEVAAETIKRENPHITTVDIV--LEGTS-V-
                                      .:. .     .   :  :

PpPINI-1        PKGT--QIN-RVVLYVDE--E---E-----KVARI-PHVG--------------------
CsPINI-1        TTDY--RAD-RIRIYFDPATY---------LVVQPRPSVG--------------------
PpPINI-2        TMDY--NEN-RVRLFVDD--E---R-----KVVKC-PTIG--------------------
AtPINI-2        LAVN--CCN-RVIVRVPV------NNCPNGPVSNF-PHIG--------------------
OsPINI-2        TDDY--CCN-RVRILVDC--GDGGGDCGDASVTAV-PMIG--------------------
OsPINI-14       PPEH--DPA-RVAIFVDG--D---I-----RVSRT-PVVG--------------------
OsPINI-1        PTDH--DDL-RVIIVSDA--G---T-----VVVRT-PVVG--------------------
SbPINI-22       PTDY--NPK-RVIIIFDS--G---N-----VVVRT-PVVGETSTCTNGIGFLSESLTTSG
SbPINI-21       PTNY--DPN-RVIIVGDN--G---S-----VVVRT-PVIG--------------------
SbPINI-23       PTDY--DPN-RVIIVADG--G---S-----VVVRT-PVIG--------------------
SbPINI-3        SKGF--NPR-RVRVFYDR-PGGLAG-----PRRQG-PRRR--------------------
VvPINI-6        TMDF--NT--RVRLHVDS--E---G-----KVSRA-PRIG--------------------
OsPINI-7        PPGF--DGE-RVLVFLND--DNA-A-----TVAQT-PVVG--------------------
SbPINI-17       TDDF--NTM-RVRVFFDK--V---G-----NVAEV-PKIG--------------------
OsPINI-13       TTEV--DPK-RVRVFVNN--S---A-----TVAQV-PKI---------------------
OsPINI-8        SPEF--NPK-RVRVFVDN--N---F-----IVVKV-PVIS--------------------
SbPINI-18       IPGY--NAK-RVRVHIDD--N---G-----NVSEV-PVIG--------------------
SbPINI-19       IPGY--NGK-RVRVHIDD--S---G-----NVSEI-PKIG--------------------
SbPINI-20       IPGY--NGK-RVRVHIDD--S---G-----NVSEI-PKIG--------------------
OsPINI-3        TPDF--NDK-RVRVFMDD--N---G-----IVFKI-PVIG--------------------
OsPINI-4        TPDF--NDK-RVRVFMDD--N---G-----IVFKI-PVIG--------------------
SbPINI-1        IPDF--NPT-RVRVFINN--G---G-----FVNQV-PVIG--------------------
SbPINI-2        IPDY--NPL-RVRVFIDN--N---N-----LVSQV-PVIG--------------------
SbPINI-5        PPKY--FNNLRVRVYVDTSYA---G-----SPVYV-PVVG--------------------
SbPINI-4        APGY--NAL-RVRVYFDA--GNATG-----PVVYV-PVVGY-------------------
OsPINI-6        PPGF--DAN-RVRVFFNA--GDAVG-----PVMRT-PFVG--------------------
OsPINI-5        LGVV--VPN-RVILWVDT-------------VAEI-PKIG--------------------
OsPINI-15       DQGLPPQPRPHLRRHRRR--HPHHRLACLICLPSA-SCLS--------------------
SbPINI-6        TLDY--RTG-RVRVFVDS--D---D-----KVARA-PQIG--------------------
SbPINI-15       THDF--RPN-RVRIFVDT-------------VAQT-PMVG--------------------
SbPINI-16       TNDF--RPN-RVRIFLDT-------------IVSP-PRVG--------------------
OsPINI-9        TLDF--RSN-RVRIFVDT-------------VAGT-PTIG--------------------
OsPINI-11       TMDF--RPN-RVRIFVDT-------------VAGT-PTIG--------------------
OsPINI-12       TRDL--RPN-RVRIFGSA------------TVAET-PRVG--------------------
OsPINI-10       PEDF--RFN-RVRIFVDT-------------VAET-PRVG--------------------
SbPINI-11       PKDF--QPN-RVRIFVDI-------------VVET-PHVG--------------------
```

```
SbPINI-12       TRDF--RPN-RVRIFVDT-------------VAET-PRVG--------------------
SbPINI-10       TGDY--LPD-RVRVFVDT-------------VAET-PHVG--------------------
SbPINI-9        TADF--VPS-RVRIFVDT-------------VAQT-PHIG--------------------
SbPINI-7        TRDY--RPN-RVRIFVDI-------------VAEA-PHVG--------------------
SbPINI-8        TMDY--RTN-RVRIFVDT-------------VAQT-PHVG--------------------
SbPINI-14       TQDF--RLD-RVRIFVDI-------------VAET-PIVG--------------------
SbPINI-13       TLDI--RPD-RVRIFVDT-------------VAKT-PTVG--------------------
SmPINI-1        TADF--DKN-RVRIYTDC--S---N-----RVVRT-PSIG--------------------
MtPINI-1        TFDL--RYD-RVRLYVDE--F---N-----NVFST-PKIG--------------------
MtPINI-2        TADY--NTQ-RVRLYVDQ--S---N-----KLIKT-PTIG--------------------
MtPINI-3        TADF--RFK-RVRLYVDE--S---N-----KVIRT-PIIG--------------------
MtPINI-4        ---F--RFK-RVRLYVDQ--S---N-----KLIKT-PTIG--------------------
AtPINI-1        IENF--CCN-RVFVYLGS--N---G-----QVADA-PMIG--------------------
VvPINI-5        TMDY--RCD-RVWVWTTEGQT---G-----VVVEV-PKVG--------------------
AtPINI-4        LAVV--ICD-RVYVRVND--Q---G-----IVTRT-PISLANLIVIYIYIYIYICVCVCE
AtPINI-6        TEDL--SCY-RVRVWVDE--I---R-----IVVRN-PTAG--------------------
AtPINI-5        TADF--RCD-RVRVFVDG--N---R-----IVVKT-PKSG--------------------
AtPINI-3        PEIF--ICS-RVYVWVND--C---G-----IVVQI-PIIG--------------------
FvPINI-1        TFDM--RRD-RVRVWIDE--R---G-----IVTKA-PKIG--------------------
FvPINI-2        PADLVRVCD-RVRVWVDT--C---G-----IVTRV-PTIG--------------------
MtPINI-6        TADF--RCD-RVRVFVDK--Q---D-----VVTRV-PKIG--------------------
VvPINI-3        TTDF--RCD-RVRVWVDS--Y---G-----IVSMA-PKIG--------------------
FvPINI-3        TQDI--RCD-RVRVWVDS--Y---G-----KLEFD-GLAGEGKWSGKLLESAGVEARPHQ
FvPINI-4        TDDF--RLD-RVRVWVNT--E---G-----IVTSV-PKIG--------------------
FvPINI-5        TRDF--RLD-RVRVWVNT--D---G-----IVISI-PKIGHQRFAVHFAANSSTSSVMLI
MtPINI-8        TDDL--RFD-RVRVWVDK--E---G-----IVTQV-PTIG--------------------
MtPINI-9        ILDF--RCD-RVWVWVDK--D---G-----IVFKV-PTIG--------------------
MtPINI-5        TADF--RCD-RVRVWVDK--D---G-----IVYQV-PIIG--------------------
MtPINI-7        PFDF--RCD-RVWVWINK--D---E-----IVYQV-PTIG--------------------
VvPINI-4        PADLVPVCT-RVRIWVDE--S---G-----IVTRV-PVVG--------------------
VvPINI-1        TQDF--YCT-RVRVWVDE--N---G-----IVISV-PTIG--------------------
VvPINI-2        TKDF--YCT-RVRVWVDE--N---G-----IVISV-PTIG--------------------
                          .:

PpPINI-1        ----------------------------------------------------------
CsPINI-1        ----------------------------------------------------------
PpPINI-2        ----------------------------------------------------------
AtPINI-2        ----------------------------------------------------------
OsPINI-2        ----------------------------------------------------------
OsPINI-14       ----------------------------------------------------------
OsPINI-1        ----------------------------------------------------------
SbPINI-22       AAASLSGPPPTNGDAALSGPTADPVASDPSSSQQHAAAQPDSTMESGTPISGLGGASS
SbPINI-21       ----------------------------------------------------------
SbPINI-23       ----------------------------------------------------------
SbPINI-3        ----------------------------------------------------------
VvPINI-6        ----------------------------------------------------------
OsPINI-7        ----------------------------------------------------------
SbPINI-17       ----------------------------------------------------------
OsPINI-13       ----------------------------------------------------------
OsPINI-8        ----------------------------------------------------------
SbPINI-18       ----------------------------------------------------------
SbPINI-19       ----------------------------------------------------------
SbPINI-20       ----------------------------------------------------------
OsPINI-3        ----------------------------------------------------------
OsPINI-4        ----------------------------------------------------------
SbPINI-1        ----------------------------------------------------------
SbPINI-2        ----------------------------------------------------------
SbPINI-5        ----------------------------------------------------------
SbPINI-4        ----------------------------------------------------------
OsPINI-6        ----------------------------------------------------------
OsPINI-5        ----------------------------------------------------------
OsPINI-15       ----------------------------------------------------------
SbPINI-6        ----------------------------------------------------------
SbPINI-15       ----------------------------------------------------------
SbPINI-16       ----------------------------------------------------------
OsPINI-9        ----------------------------------------------------------
OsPINI-11       ----------------------------------------------------------
OsPINI-12       ----------------------------------------------------------
OsPINI-10       ----------------------------------------------------------
SbPINI-11       ----------------------------------------------------------
SbPINI-12       ----------------------------------------------------------
SbPINI-10       ----------------------------------------------------------
SbPINI-9        ----------------------------------------------------------
SbPINI-7        ----------------------------------------------------------
SbPINI-8        ----------------------------------------------------------
SbPINI-14       ----------------------------------------------------------
SbPINI-13       ----------------------------------------------------------
SmPINI-1        ----------------------------------------------------------
MtPINI-1        ----------------------------------------------------------
MtPINI-2        ----------------------------------------------------------
MtPINI-3        ----------------------------------------------------------
MtPINI-4        ----------------------------------------------------------
AtPINI-1        ----------------------------------------------------------
```

```
VvPINI-5        ----------------------------------------------------------
AtPINI-4        SIMDLNM---------------------------------------------------
AtPINI-6        ----------------------------------------------------------
AtPINI-5        ----------------------------------------------------------
AtPINI-3        ----------------------------------------------------------
FvPINI-1        ----------------------------------------------------------
FvPINI-2        ----------------------------------------------------------
MtPINI-6        ----------------------------------------------------------
VvPINI-3        ----------------------------------------------------------
FvPINI-3        RRTVANRRKSAP----------------------------------------------
FvPINI-4        ----------------------------------------------------------
FvPINI-5        ERNVSLKIEHYPWRPAIIG---------------------------------------
MtPINI-8        ----------------------------------------------------------
MtPINI-9        ----------------------------------------------------------
MtPINI-5        ----------------------------------------------------------
MtPINI-7        ----------------------------------------------------------
VvPINI-4        ----------------------------------------------------------
VvPINI-1        ----------------------------------------------------------
VvPINI-2        ----------------------------------------------------------
```

G. I20 Pin-II.

```
SmPinII-2b      KDAVEICGGTVHPWKPGSC------------------------ATCCSIPQGCLDL--GH
SmPinII-4b      KDAVEICGGTVHPWKPGSC------------------------ATCCTIPQGCMDL--GH
SmPinII-1b      ---VEICGGTVHPWTPGSC------------------------ATCCTIPQGCLDL--GH
SmPinII-3b      ---VEICGGTVHPWTPGSC------------------------ATCCTIPQGCLDL--GH
SmPinII-2a      MAASFLFLTILSFQLLLISADDFAASGHRLQVCSDAVVTLDECSNCCTLEPGCIYLEPGT
SmPinII-4a      MAASFLFLTILSFQLLLISADDFAASSHRLQVCSDAVVTLDGCSSCCALEAGCIYLEPGT
SmPinII-1a      MAASFLFLTILSFQLLLIS----AAGSHRLQVCSDAVVTLDECSNCCALKAGCIYLEPGT
SmPinII-3a      MAASFLFLTILSFQLLLIS----AASSHRLQVCSDAVVTLEECSNCCALKAGCIYLEPGT
SbPinII-1       MATIKACSSRLVPVALLLC--------------------------------GLIVM--GS
OsPinII-1       MASIKLAL----PMALLLC--------------------------------GLMVI--GS
AtPinII-1       MVTYKIW-----VMSFIIA--------------------------------GAILG--GI
BdPinII-1       ETWPSL------PALFLIE-----YRTTTVQRQTSDMAASRFHIACALLLFGAVLL--GQ
SbPinII-2       MAASRFY----VTCALLLI--------------------------------GVVLL--GQ
ZmPinII-1       MAASKFY---VASCALLLI--------------------------------GVVLL--GQ
RcPinII-1       MATYKVA-----LALLLLY--------------------------------GAISL--QA
VvPinII-1       MGNMKFS----VFVLLLVC--------------------------------GVVLL--GE
PtPinII-1       MGGARLA---ASAVVLLVL--------------------------------GVVLL--GA
MgPinII-1       ---------------------------------------------------ILLVL--ES
PpPinII-1       MAADKNI---GAMVLLLVC--------------------------------GSILL--LA
FvPinII-1       MASDKSI---TAILLLLVC--------------------------------GSVIL--GV
PpPinII-2       MTTRYFA--YLQPSKIHQK--------------------------------GSILL--GI
                                                                     :

SmPinII-2b      GKSICVSGPD--KTCTFKC-GSAS-Y-VCPGPKSVALDFNSCHTCRKPLKE-GCKLYNR-
SmPinII-4b      GKSVCVSGPG--KACTLEC-GFAS-Y-VCPGPKSVALDFNSCHTCKKPLRE-GCKLYNR-
SmPinII-1b      GKSICVSGPG--KVCTREC-GFVS-Y-VCPGPKSVALDFNSCHTCKKPLRE-GCKLYNR-
SmPinII-3b      GKSICVSGPG--KVCTREC-GFVS-Y-VCPGPKSVALDFNSCHTCKKPLKE-GCKLYNR-
SmPinII-2a      GSPVCLGGTSPARACLPIC-LPPK-IKVCPSRWTNQIPISVLPPGKEP--SNGCHLYNLK
SmPinII-4a      GSPVCLGGTSPFRPCPLIC-HPPK-IKVCPSRGTNRIPISVLPPDKEP--SNGCHLYNLK
SmPinII-1a      GSPVCLGGTSASRLCPLIC-EPPK-IKVCPSRGTNQIPISVLPPDKEP--SNGCHLYNLK
SmPinII-3a      GSPVCLGGTSPFRPCPLIC-QPPK-IKVCPSPGTNQIPISVLPPDKEP--SNGCHLYNLK
SbPinII-1       ISGLEAKDKDG-KVCIETC-QEAY-YMTCPSTGNAKLN-PACNCCLASLKEDGCTIYLK-
OsPinII-1       IQSAEAQGG---KFCPQFCYDGLE-YMTCPSTGSQHLK-PACNCCIAG--EKGCVLYLN-
AtPinII-1       IPGVTTTKTA--IACPLYC-LQVE-YMTCPSSGADKLP-PRCNCCLAP--K-NCTLHLS-
BdPinII-1       DGKA---GMEA-VACPQYC-LEVE-YTTCPSSGSEKLP-ARCNCCMAP--K-GCTLHLS-
SbPinII-2       Q------GIEGAVACPQYC-LEVD-YVTCPSSGSEKLP-ARCNCCLAP--K-GCTLHLS-
ZmPinII-1       Q------GIDGAVACPQFC-LDVD-YVTCPSSGSEKLP-ARCNCCMTP--K-GCTLHLS-
RcPinII-1       TSG---------KACPLYC-LDVE-YMTCQSSGDEKLN-PSCNCCLAP--K-NCTLHLS-
VvPinII-1       TSKS----FGA-KACPLYC-LDVD-YMTCVSSGEEKLT-APCNCCLAP--K-QCTLHLV-
PtPinII-1       SGGN----LIA-KACPLYC-LDVD-YMTCESSGDKKLN-SACNCCLAP--K-NCTLHLA-
MgPinII-1       NAQY----ASA-KVCPLYCIINQGIYMTCPSSGSQKLE-PVCNCCLAK--T-GCKLYRD-
PpPinII-1       INPT-----EA-KVCNKICYGAAA-YMTCPSSGSTQLD-PVCNCCLAP--ALGCTLYES-
FvPinII-1       VPTEA-------KVCPFICYDAAA-YMTCPSSGNEQLS-PPCNCCLAP--APGCALYRA-
PpPinII-2       NPSTA-------KICPQYCTDQAG-YMTCPSSGNTQLS-PSCNCCLAP--A-GCTLYRA-
                              *   *        .* .     :                * ::

SmPinII-2b      -------QGHQL-CVKEDDLLQLV
SmPinII-4b      -------RGHQL-CVKEDDLLQLA
SmPinII-1b      -------QGHQL-CVKEDDLLQLA
SmPinII-3b      -------QGHQL-CVKEDDLLQLA
SmPinII-2a      TGAPLP-NGDDNSCL---------
SmPinII-4a      TGAPLP-NGDDNSCL---------
SmPinII-1a      TGAPLPVNGDDKSCLKDA------
SmPinII-3a      TGAPLPVNGDDKSCLKDA------
SbPinII-1       -------DGTVEKCPRT-------
OsPinII-1       -------NGQVINCT---------
AtPinII-1       -------DSTTIHCSK--------
BdPinII-1       -------DGTKQTCR---------
SbPinII-2       -------DGTQQTCS---------
ZmPinII-1       -------DGTQQTCS---------
RcPinII-1       -------DGTSLFCKPN-------
VvPinII-1       -------DGSEVQCD---------
PtPinII-1       -------DGRMVQC----------
MgPinII-1       -------NGTLI-CTAT-------
PpPinII-1       -------DGTPI-CTST-------
FvPinII-1       -------DGTRL-CTTT-------
PpPinII-2       -------DGTSI-CTGT-------
                        .    *
```

H. I25 Cystatin.

```
VcCPI-1         ----------------------------------------M---KHYF-LIFCTLILFSS
CrCPI-1         ---------------------------------------MA---KRVL-IALAAFVMLNA
SbCPI-17        ---------------------------------------MKKQSAAIVAVLALVAFLATA
OsCPI-18        ---------------------------------------MV---AIAL-FLLLVVAGAGA
SbCPI-6         --------------------------------------------MRSL-IALAVVLVVVA
OsCPI-17        --------------------------------------------MKSL-AAVLIVLAAAA
OsCPI-14        ---------------------------------------MRQSSRLSIIAVVISVTLVAI
SbCPI-8         --------------------------------------MARPSVLVVVLPIVLATLLATA
OsCPI-13        -------------------------------------MRPSSLSTVVLSVIFTAALLAVA
SbCPI-18        ---------------------------------------MRSLLALLVTALVAVVVAFPD
OsCPI-15        ---------------------------------------MRTSSSLLVAAALVFVVVVAE
OsCPI-16        --------------------------------------MARSSPLFLLGAALAVVAVAAA
SbCPI-7         ---------------------------------------MR---SSPV-VLPLLAAALLV
VvCPI-3         MNSTLGLLEKVDLSVHWKDKRMKPLFTFHCPWIRGGGARHL---LSSS-LVFYHVDPTEV
FvCPI-11        ---------------------------------------MH---NPLY-LLVLFALLLIG
FvCPI-5         -------------------------MAAAIRSMIGKVGNTRSVRNDVTSFPISNPHLHLG
AtCPI-2         ---------------------------------------MATMLKVSLVLSLLGFLVIAV
FvCPI-4         --------------------------------------MDHSGDSNPV------------
FvCPI-10        ----------------------------------------M---SPHY-LIAIFALLFPL
SbCPI-12        ---------------------------------------MRPGQKLLFKWHLVIFLIGHY
SbCPI-16        ------------------------------------MASST---MRTG-LLFVVVVAAIH
SbCPI-19        ---------------------------------------MR---TSFLLLAAAICAVVAS
OsCPI-11        --------------------------------------MAR---HPGLLLILLAAVAAVA
SbCPI-5         ---------------------------------------MR---TSHLVVMAATAIFIAA
SbCPI-1         ---------------------------------------MMRALISMLIVAAAVVGLCSV
SbCPI-2         ---------------------------------------MRTLISMLVVAAAAVVGLCSV
OsCPI-9         ---------------------------------------MRTSSLVLFAAVAVFGAACTA
SbCPI-4         --------------------------------------MATPGHHLPLLLLVLATLAAAA
OsCPI-7         ---------------------------------------MT---MRTSSLLLAAVAVVAI
OsCPI-6         -------------------------------------------------MAMTTRTLLLA
OsCPI-8         --------------------------------------------MARIPLLLALLLAVSA
SbCPI-3         ------------------------------------MATTR---ALLL-TTTAAALLLLL
FvCPI-7         ----------------------------------------M---HPQY-IFAVLVLLVPL
FvCPI-9         ----------------------------------------M---LRPH-CLLVLLALLFP
OsCPI-10        ---------------------------------------MATSPMLFLVSLLLVLVAAAT
OsCPI-5         --------------------------------------MASKLYYAVAPLVLVLLLLAPL
OsCPI-4         -------------------------------------MAAR---CPVG-VASVLLLIVLV
SbCPI-11        -------------------------------------MDPR---ALGAGMLLAVLLMAAT
MtCPI-2         --------------------------------------------MAMTLTIVITTLLCIL
FvCPI-3         ------------------------------------MALIKSPAARIAAATVLMSLLFTM
OsCPI-3         ------------------------------MLRRRGFCCCS---GAPA-AAAAALL-LLA
SbCPI-13        --------------------------------------MPR---CAVVVLFAAVLLAASA
SmCPI-2         ------------------------------------------------------------
SmCPI-3         ------------------------------------------------------------
PpCPI-5         ---------------------------------------MAPNHKVSV-LLAAVCLFGIY
SmCPI-1         ------------------------------------------------------------
SbCPI-15        ------------------MSLLRGVQRLALGVAAAAAARGR---LPLA-ALLRFRFSSAY
SbCPI-20        ------------------------------------------------------------
SbCPI-14        ----------------------MRIWLRGATTIRRCKCYSC---SFPL-AVVPVVSPPPP
AtCPI-1         ------------------------------------------------------------
OsCPI-2         -------------------MRASSLFAESVFTTSAAAGRRR---CPRL-AAVPVTLFFST
PpCPI-4         ------------------------------------------------------------
PpCPI-1         ------------------------------------------------------------
PpCPI-2         ------------------------------------------------------------
PpCPI-3         ------------------------------------------------------------
MtCPI-1         ------------------------------------------------------------
OsCPI-1         ---------------------------------------MRKYRVAGL-VAALLVLHSLA
SbCPI-10        -----------------------------------MRKHRI---VPLV-AALLVLLALAV
AtCPI-7         --------------------------------MDMRRASMC---MMLI-CVSLVLLSGFG
MtCPI-12        ------------------------------------------------------------
FvCPI-1         MNSIQRPKQAEHSPTQFNSLSKLQNPNSSISKSIKISHNPVKPKTHKATSSFAFIHIHRF
VvCPI-5         ------------------------------------------------------------
AtCPI-3         ---------------------------------------------MES-KTFWIVTLLLC
VvCPI-2         ------------------------------------------------------------
VvCPI-4         ---------------------------------------RH---FPPS-AIHFRRKYTLL
AtCPI-6         ---------------------------------------MM---RSRF-LLFIVFFSLSL
OsCPI-12        ---------------------------MRVAATTRPASSSA---AAPL-PL-FLLLAVAA
SbCPI-9         ---------------------------------MRVAATRLAAAPPPIRFLFLLVLLGSA
MtCPI-19        ---------------------------MRA--------TIY---ILPF-LVFFFFFFALF
FvCPI-2         --------------------------------------------MATL------------
FvCPI-12        ------------------------------------MMRTQ---CFLL-ALFAFLVPLVA
FvCPI-8         ---------------------------------------MKKHVFSLDLFAFLLVAGLFA
AtCPI-4         ---------------------------------------MM---MKSL-ICLSLILLPLV
MtCPI-20        ---------------------------------------MR---LESM-VLVLVVLLAFT
MtCPI-14        ---------------------------------------MT---LQSP-VFILIVLLVLS
MtCPI-16        ---------------------------------------MK---LQSL-VFILIVLLALS
MtCPI-11        ---------------------------------------MR---FQSL-VLILAVLFASF
MtCPI-7         ---------------------------------------MR---FQSL-ILILIVLFALA
MtCPI-4         ---------------------------------------MR---FQSL-VLILVVLLASA
MtCPI-9         ---------------------------------------MR---FQSL-VFILVVLLASV
MtCPI-18        ---------------------------------------MR---FQFI-FLILVVLFVSL
```

```
MtCPI-10        --MEGVFNKCNQQDHHARRRHPTMWWISMRLVCPPLVGTCQ---LNSVPILKLDNLVRKP
MtCPI-17        ---------------------------------------MR---FQSI-ILILFILFSSL
FvCPI-6         --------------------------------------------MRPFCLLAILAVLLPL
MtCPI-15        --------------------------------------------MKLQLFVLFVVLMSYA
MtCPI-21        ---------------------------------------MR---LQSV-VFLLLGFLATM
MtCPI-13        ---------------------------------------MR---LQWL-VLLVLVLMVST
MtCPI-8         ---------------------------------------MKLYNVAIFVLFLSVVSVFTV
AtCPI-5         ---------------------------------------MTSKVVFLL-LLSLVVVLLPL
VvCPI-1         -----------MQPTTRLSLLFTALPDISIRENCEKSTTMAWGQNPLFLFLTAALLLLNG
MtCPI-3         ---------------------------------------MR---FQYL-VIFLLVLLASA
MtCPI-6         ---------------------------------------MR---FQYL-VIFLLVLLASA
MtCPI-22        ---------------------------------------MRNPTLLLV-LLFVVVLFSYA
MtCPI-5         ---------------------------------------MR---IQLL-VLFVVVLMASA


VcCPI-1         AWV---------------------------KAT-----------------------ADTG
CrCPI-1         ATA---------------------------TI----------------------------
SbCPI-17        LTV---------------------------VVA---------------------------
OsCPI-18        GAQ---------------------------GGDA----------------------GSLP
SbCPI-6         GAA---------------------------SAK---------------------------
OsCPI-17        AVA---------------------------ATA---------------------------
OsCPI-14        TNN---------------------------ADV-------------------ALASSLPP
SbCPI-8         NAQDYTAPTPSPLPDTPTPPPPPPPPESSSTAPPPASQPPPAASSSPPPPSPPPPAYSSP
OsCPI-13        KAD---------------------------GGS-----------------------VTAA
SbCPI-18        GAD---------------------------AVS---------------------------
OsCPI-15        TLP---------------------------------------------------------
OsCPI-16        TLP---------------------------AAT---------------------------
SbCPI-7         ASS---------------------------SAQ---------------------------
VvCPI-3         RNQ---------------------------TGSYGKAFMEYEKDVSKENREKIEKWRAAL
FvCPI-11        GNT---------------------------QPI------------NLDG-----------
FvCPI-5         YSS---------------------------RHPLDLFPVVHHPRNQRLPKKSEPDSRPSI
AtCPI-2         VTP---------------------------SAA------------------------NPF
FvCPI-4         ------------------------------------------------------------
FvCPI-10        LTT---------------------------AVD------------NIGDYT---------
SbCPI-12        STA-----------------NQNQLLLHGLATTKPAMRAAVLLGVLGIAIVAIHIVAMAT
SbCPI-16        AFA---------------------------TST---------------------------
SbCPI-19        VVS---------------------------------------------------------
OsCPI-11        TTS---------------------------RAQ---------------------------
SbCPI-5         TTP---------------------------AMA---------------------------
SbCPI-1         APA---------------------------ASA------------------------REA
SbCPI-2         APA---------------------------ASA-----------------------RGEP
OsCPI-9         AAG---------------------------DES---------------------------
SbCPI-4         FAV---------------------------APV--------------------PGPAALG
OsCPI-7         VAG--------------------------ATAA---------------------------
OsCPI-6         AVC--------------------------AAAA---------------------------
OsCPI-8         AAA---------------------------AQV-----------------------GGNR
SbCPI-3         AGG---------------------------GGA-------------------------AA
FvCPI-7         VVI---------------------------VSV---------------------------
FvCPI-9         ILT---------------------------AAA---------------------------
OsCPI-10        GDE---------------------------ASP---------------------SNAAAP
OsCPI-5         SSA---------------------------RLA------------AAAAADDDGQWPAGG
OsCPI-4         TVA---------------------------SAASGARS-------GGGGGGGIRELRGGG
SbCPI-11        AAA---------------------------SAR------------PDDDDVAGSGAGAGG
MtCPI-2         STA---------------------------SCG---------------------------
FvCPI-3         SHG---------------------------YGG---------------------------
OsCPI-3         VAA---------------------------AAP------------RAAGFHLGGDES---
SbCPI-13        AAV---------------------------SGF-----------------------HLGG
SmCPI-2         ------------------------------MTT---------------------------
SmCPI-3         ------------------------------MTT---------------------------
PpCPI-5         LVG---------------------------AQA---------------------------
SmCPI-1         ------------------------------------------------------------
SbCPI-15        YYP---------------------------TSA------------AAARSRFLATGSRML
SbCPI-20        ------------------------------------------------------------
SbCPI-14        LPP---------------------------GSR------------RRTASSAMAEAHSGR
AtCPI-1         --------------------------------------------------MADQQAGT--
OsCPI-2         GRG---------------------------SPA------------MAEEAQQPRGVKV--
PpCPI-4         ------------------------------------------------------------
PpCPI-1         ------------------------------------------------------------
PpCPI-2         ------------------------------------------------------------
PpCPI-3         ------------------------------------------------------------
MtCPI-1         --------------------------------------------------------MA--
OsCPI-1         TPS---------------------------AQA------------EAHRAGGEGEEKMSS
SbCPI-10        SST---------------------------R---------------NRNAQEGEESMA--
AtCPI-7         QFV---------------------------ICS------------EEKGTYNDNVVKM--
MtCPI-12        --------------------------------------------------------MA--
FvCPI-1         QSL---------------------------SLS------------IQLPSSTLSLYFSPE
VvCPI-5         --------------------------------------------------------MA--
AtCPI-3         GTI---------------------------QLA------------ICRSEEKSTEKTM--
VvCPI-2         --------------------------------------------------------MN--
VvCPI-4         LEG---------------------------ECR------------SPKVLRTVPRSIA--
AtCPI-6         FIS---------------------------SLI------------ASDLGFCNEEMA---
OsCPI-12        AAA---------------------------ALF------------LVGSA-SLAMAGH--
SbCPI-9         IGA---------------------------AMA---------------------------
```

```
MtCPI-19        IVL---------------------------ESS------------GDCSDFDHAQMAT--
FvCPI-2         ------------------------------------------------------------
FvCPI-12        SAF---------------------------RGP------------S-ED-----------
FvCPI-8         SLS---------------------------PLL-----------------------LATP
AtCPI-4         SVV---------------------------EGL---------------------------
MtCPI-20        ATK---------------------------QAI---------------------------
MtCPI-14        ATN---------------------------QAL---------------------------
MtCPI-16        IIN---------------------------QAI---------------------------
MtCPI-11        ATN---------------------------QAI---------------------------
MtCPI-7         TTN---------------------------QAT---------------------------
MtCPI-4         STN---------------------------QAD---------------------------
MtCPI-9         ATN---------------------------QAD---------------------------
MtCPI-18        ASN---------------------------QAK---------------------------
MtCPI-10        RPN---------------------------HEI---------------------------
MtCPI-17        ATN---------------------------QAL---------------------------
FvCPI-6         AAA---------------------------------------------------------
MtCPI-15        DAR---------------------------KQP---------------------------
MtCPI-21        SVR---------------------------NQA---------------------------
MtCPI-13        TAT---------------------------KQL---------------------------
MtCPI-8         SDG---------------------------KDA---------------------------
AtCPI-5         YAS---------------------------AAA---------------------------
VvCPI-1         GFA---------------------------ART---------------------------
MtCPI-3         -AR---------------------------NQA---------------------------
MtCPI-6         -AR---------------------------NQA---------------------------
MtCPI-22        AAR---------------------------NQF---------------------------
MtCPI-5         IAR---------------------------MET---------------------------


VcCPI-1         DQHHGLGA------------VV--------------------------------------
CrCPI-1         -----VGG------------SS--------------------------------------
SbCPI-17        -DPAPPPS-Y----------TK--------------------------------------
OsCPI-18        PAPAATGE-W----------VP--------------------------------------
SbCPI-6         ---------F--------VDPP--------------------------------------
OsCPI-17        ----SASE-F--------KQTP--------------------------------------
OsCPI-14        APPAAAAG-W----------TA--------------------------------------
SbCPI-8         PPPPPPASNW----------TP--------------------------------------
OsCPI-13        AAAPPPAA-W----------TA--------------------------------------
SbCPI-18        ---------W----------TP--------------------------------------
OsCPI-15        ----AAEATY----------RP--------------------------------------
OsCPI-16        ----EAAG-W----------AP--------------------------------------
SbCPI-7         ------AA-W----------VP--------------------------------------
VvCPI-3         KEAINLSG-WHLHNQRGSIRIPVSFQSINPTHHHHHHHTKTHPKPTSSTSLMAHTQAIRW
FvCPI-11        ---PDTGG-Y----------QR--------------------------------------
FvCPI-5         PEMPYLGT------------GP--------------------------------------
AtCPI-2         RKSVVLGG-K----------SG--------------------------------------
FvCPI-4         ----NDSA-W----------FP--------------------------------------
FvCPI-10        ----LIGA-W----------KP--------------------------------------
SbCPI-12        PAMAQTFGVW----------YP--------------------------------------
SbCPI-16        ----MAET------------TP--------------------------------------
SbCPI-19        ---ADSQG-W----------FP--------------------------------------
OsCPI-11        ----WVGG-W----------NV--------------------------------------
SbCPI-5         EIGRLQGGFY----------QP--------------------------------------
SbCPI-1         PLPENVGR-W----------TP--------------------------------------
SbCPI-2         PVPQAVGG-W----------KP--------------------------------------
OsCPI-9         ---------W----------KT--------------------------------------
SbCPI-4         GGGPLLGG-W----------NP--------------------------------------
OsCPI-7         ----TVGS-W----------EP--------------------------------------
OsCPI-6         ----LPRG-W----------SP--------------------------------------
OsCPI-8         GHGPLVGG-W----------SP--------------------------------------
SbCPI-3         ARGPLAGG-W----------SP--------------------------------------
FvCPI-7         ---AAEGG-W----------IP--------------------------------------
FvCPI-9         ---DGPTF-W----------VP--------------------------------------
OsCPI-10        AAPVLVGG-R----------TE--------------------------------------
OsCPI-5         GRGRKVGG-R----------TD--------------------------------------
OsCPI-4         -AGRRVGG-R----------TE--------------------------------------
SbCPI-11        IRQPASDG-Y--RGRKVGARTE--------------------------------------
MtCPI-2         --RVIVGA-R----------TE--------------------------------------
FvCPI-3         ----LVGG-R----------RP--------------------------------------
OsCPI-3         ---VLVRG-M----------LA--------------------------------------
SbCPI-13        DESGLVRGVL----------AA--------------------------------------
SmCPI-2         ----LVGA-P----------KP--------------------------------------
SmCPI-3         ----LVGA-P----------KP--------------------------------------
PpCPI-5         ---MSVGG-P----------KD--------------------------------------
SmCPI-1         --SSLVGG-W----------SA--------------------------------------
SbCPI-15        APDLLCGG-V--------VDAP--------------------------------------
SbCPI-20        ----MVDD-V--------QDTP--------------------------------------
SbCPI-14        RVGMV-GD-V--------RDAP--------------------------------------
AtCPI-1         ---IV-GG-V--------RDID--------------------------------------
OsCPI-2         ------GG-I--------HDAP--------------------------------------
PpCPI-4         ---MLSGG-K--------QEVD--------------------------------------
PpCPI-1         ---ML-GG-K--------KEVD--------------------------------------
PpCPI-2         ----MLGG-K--------KEVD--------------------------------------
PpCPI-3         ----MLGG-K--------KEVD--------------------------------------
```

```
MtCPI-1         ---TV-GG-V--------RDVS--------------------------------------
OsCPI-1         DGGPVLGG-V----------EP--------------------------------------
SbCPI-10        ---LA-GG-I--------KDVP--------------------------------------
AtCPI-7         ---KL-GG-F--------SDSK--------------------------------------
MtCPI-12        ---AL-GG-S--------TEVE--------------------------------------
FvCPI-1         KMAAVLGGVH----------ES--------------------------------------
VvCPI-5         ---TV-GG-I--------HDSD--------------------------------------
AtCPI-3         ---ML-GG-V--------HDLR--------------------------------------
VvCPI-2         ---LL-GG-V--------RDCG--------------------------------------
VvCPI-4         ---LL-DL-L--------LKNT--------------------------------------
AtCPI-6         ---LV-GG-V--------GDVP--------------------------------------
OsCPI-12        ---VL-GG-A--------HDAP--------------------------------------
SbCPI-9         --GHVLGG-V--------KENP--------------------------------------
MtCPI-19        ---PL-GG-I--------QDSP--------------------------------------
FvCPI-2         ------GG-I--------GDSP--------------------------------------
FvCPI-12        ---QIVGA-W----------EI--------------------------------------
FvCPI-8         GDHPNYGA-Y----------RP--------------------------------------
AtCPI-4         ----GGGG-G------LGSRKP--------------------------------------
MtCPI-20        ----PIGN-L----------SP--------------------------------------
MtCPI-14        ----TFID-W----------SP--------------------------------------
MtCPI-16        ----TSSD-W----------GP--------------------------------------
MtCPI-11        ----PPNN-L----------RR--------------------------------------
MtCPI-7         ---------------------F--------------------------------------
MtCPI-4         ---------------------F--------------------------------------
MtCPI-9         ---------------------F--------------------------------------
MtCPI-18        ----RPIQ-S----------SP--------------------------------------
MtCPI-10        -----SIH-CFYSLCFICLIDR--------------------------------------
MtCPI-17        -----GIS-T-----------R--------------------------------------
FvCPI-6         ----VPGG-W----------SP--------------------------------------
MtCPI-15        ----LSDG-W----------SR--------------------------------------
MtCPI-21        ----IAGG-W----------EP--------------------------------------
MtCPI-13        ----IVRG-W----------DP--------------------------------------
MtCPI-8         ----LVGG-W----------TP--------------------------------------
AtCPI-5         ----RVGG-W----------SP--------------------------------------
VvCPI-1         --EALAGG-W----------RP--------------------------------------
MtCPI-3         ----KPGG-Y----------SP--------------------------------------
MtCPI-6         ----IPGG-Y----------SP--------------------------------------
MtCPI-22        ----APGG-W----------SP--------------------------------------
MtCPI-5         ----SAGG-W----------SP--------------------------------------


VcCPI-1         --EADV---D-N-PAIRDAADYVTRTANTNN--------CNGLCASLKRTGKLKLLEILS
CrCPI-1         --KAAV---S-D-PDVVHAANFVVSSANTNA--------CSGLCAGLQKEGELKLVKVLS
SbCPI-17        --QEDV---S-S-DFIKQVGKFAVNVYR-----------LAHM-------IPMNYLSTSK
OsCPI-18        --IGGDVIRG-D-GLYRQVARFALVVRMLAF--------GAAE-------GELTLVEVVA
SbCPI-6         --DSDV---PAG-TRPPQIGRFAVLVYN-----------LNRG-------AKLKYAGVSN
OsCPI-17        --VADM---PVD-PRGPLLGRFAVLVYS-----------LNRN-------RRLTYAGVSL
OsCPI-14        --VANV---N-D-KSIQQAGQFALWIYR-----------QITR------LYFLRYVNVVS
SbCPI-8         --VANV---K-D-PKIQQVAQFAVRIHA-----------LSST------ELNMQLQIVVS
OsCPI-13        --VANV---N-D-KSIQQVGQSAVRIYG-----------LSTN------KTYLRFVNVVS
SbCPI-18        --IANP---G-T-TLVKQVGNFCVIVYSNSD--------RRRH-------LPLQLVIVVR
OsCPI-15        --IGNT---S-N-LVILQVGRFSVLVYD-----------LSHR-------KSLVFVSVVS
OsCPI-16        --VADV---Q-E-LVIQQVGRFAVLVYS-----------LAHH-------TDLAYVGVAR
SbCPI-7         --VLDV---N-E-LVIKQVAQFAVLVYG-----------LAHH-------RDLAYVGVVR
VvCPI-3         TKVQDV---E-E-KHIQELGEYAVEEQN-----------KKEN-------WNLEFEKVSM
FvCPI-11        --IPNP---ATD-PDVKEIVEFAISEIN-----------FQSL-------KNLVLTGIVR
FvCPI-5         --INDS---SRDCPRVVINAHFALEEFN-----------KQKN-------AQLQFVRVVK
AtCPI-2         --VPNI---RTN-REIQQLGRYCVEQFN-QQAQNEQGNIGSIAKTDTAISNPLQFSRVVS
FvCPI-4         --LKNV---S-D-PRVTEIANYAVDVYT-----------RQNQ-------KHLIFQKVIK
FvCPI-10        --LKSI---S-D-PHVTEIANYAVDAYT-----------RQNQ-------KHLIFQKVIK
SbCPI-12        --V-DL---S-E-GHVQYIGRWAVAEHV-----------KQAK-------DGLKFDKVVG
SbCPI-16        --I-NV---N-D-PVYQKIARWAVTEHV-----------MLAN-------DGLKFNKLVS
SbCPI-19        --LPDI---D-A-PKVQQLGRWAVTEHD-----------KKAN-------DKVKFNRVVS
OsCPI-11        --IEDV---AGN-NQIQRVGAWAVGKHN-----------QLGT------NDRLQFVRVVA
SbCPI-5         --IVNI---N-D-PHVQEVGRWAVSEHV-----------KKAN-------DGLKFSRVVS
SbCPI-1         --ITDV---N-D-PEIQEIGRWAVSEHD-----------AAAS-------DSLVFSKVVR
SbCPI-2         --I-NV---N-D-PHIQELGRWAVSEHG-----------KQAS-------DRLVFGKVVS
OsCPI-9         --IDAN-----D-RHVQDVALWAVAETD----------WASAT-------GGLTLNTVDG
SbCPI-4         --IPDV---G-D-KHIQELGGWALGQAK-----------YQKL-----AANALRFRRVVR
OsCPI-7         --V-DI---N-D-PHVQELGRWAVAEED--R--------GVAA-------GGLTFERVTD
OsCPI-6         --IKNI---D-D-PHIQELGRWAITENN--R--------VSPS-------DELTFHRVTG
OsCPI-8         --ITDV---G-D-PHIQELGGWAVERHA-----------SLSS-------DGLRFRRVTS
SbCPI-3         --IRNV---S-E-PHIQELGGWAVTEHV-----------RVAN-------DGLRFGEVTS
FvCPI-7         --L-SP---S-D-PRAITVAQFAITEYN-----------KNTT-------QKLVYQSLVS
FvCPI-9         --IKNT---S-D-PVVVEVGQFAVSEYD-----------KNTT-------KKLIFEKVVS
OsCPI-10        --IRDV---GSN-KAVQSLGRFAVAEHNRRLRHGGSGGPADPV------PVKLAFARVVE
OsCPI-5         --VEDV---EGN-REVQELGLFCVVEHN-----------RRGG--SATRGRGLVFSRVVA
OsCPI-4         --VRDV---EGD-REVQELGRFSVEEHN--R----RRRSRDCG------DVRLEFGRVVA
SbCPI-11        --VRDV---ESD-GEVQELGRFSVAEYNRQLR-------GDGG-------GRLEFGRVVA
MtCPI-2         --ISDV---GTN-KEVQELGKFAVKEYN-----------YKQGLNNGGGGEGLKFVEVVE
FvCPI-3         --VEDV---KTN-KEVQELGRFSVAEYNRMQ--------RQSL--RSNGGGELQFREVVE
OsCPI-3         --AIRR---E-Q-AEAEDAARFAVAEYN-----------KNQG-------AELEFARIVK
SbCPI-13        --LRER-------AEAEDAARFAVAHHN-----------KNQG-------AALEFTRVLK
```

```
SmCPI-2         --LKEA---N-S-LEAEEHAKFAVEEHN--R--------QNPE-------ANLCFKRVVS
SmCPI-3         --LKEA---N-S-LEAEEHAKFAVEEHN-----------RQVQ-------ANLCFKRVVS
PpCPI-5         --ISNF---PNS-VEIDELANFAVDQYK-SR--------QNSI-------AVITFSKVLS
SmCPI-1         --IDPK-----D-PNVVTLAKFAVHEHN-KK--------LSGH-------GTLLYSKLVE
SbCPI-15        --G-HE---M-H-PDAIELARFAVAEHN-SK--------TNAA---------LEFVRLVK
SbCPI-20        --AGCE---K-G-LEAIKLVCFTNAEHN-----------GKTN-------AILKFVRSVK
SbCPI-14        --EGHE---N-D-LEAIELARFAVDEHN-SK--------TNAM---------LEFERLVK
AtCPI-1         --A-NA---N-D-LQVESLARFAVDEHN-KN--------ENLT---------LEYKRLLG
OsCPI-2         --AGRE---N-D-LTTVELARFAVAEHN-SK--------ANAM---------LELERVVK
PpCPI-4         --LQNS---N-N-LEIDEAAKFAVAEHN-DR--------ENSL-------EKLTFSKVVS
PpCPI-1         --VQDT---N-S-LEIDELANFAVAEHL-KS--------QNSL-------EGMTFSKVVS
PpCPI-2         --VQDT---N-S-LEIDELANFAVSEHN-AR--------QNSL-------EKMNFSKVVS
PpCPI-3         --VQDT---N-S-LEIDELANFAVSEHN-NR--------QNSL-------EKMNLSKVIS
MtCPI-1         --G-NQ---N-S-LAIDGLARFAVEEHN-KK--------QNAL---------LEFSRVIS
OsCPI-1         --VGNE---N-D-LHLVDLARFAVTEHN-----------KKAN-------SLLEFEKLVS
SbCPI-10        --A-NE---N-D-LHLQELARFAVEEHN-KK--------ANAL---------LGYEKLVK
AtCPI-7         --NDWN---G-G-KEIDDIALFAVQEHN-RR--------ENAV---------LELARVLK
MtCPI-12        --Y-TQ---N-S-VEIDNLAIFAVQEHN-IK--------QNAV---------LEFVRVLN
FvCPI-1         --QGSQ---N-S-LETDELARFAVQEHN-----------KKEN-------ALLEFVRVVK
VvCPI-5         --G-SQ---N-A-LEIENLARFAVDEHN-KK--------QNTL---------LEFNGVVK
AtCPI-3         --G-NQ---N-S-GEIESLARFAIQEHN-KQ--------QNKI---------LEFKKIVK
VvCPI-2         --G-IQ---N-S-AEIESIARFAVQEHN-KN--------QNAL---------LEFAKVLK
VvCPI-4         --TRRS---K-K-CKVEDMRRIMILQNQ-IAFVEGWTYWENAL---------LEFARVVK
AtCPI-6         --A-NQ---N-S-GEVESLARFAVDEHN-KK--------ENAL---------LEFARVVK
OsCPI-12        --S-AA---N-S-VETDALARFAVDEHN-KR--------ENAL---------LEFVRVVE
SbCPI-9         --AAAN-----S-AESDGLGRFAVDEHN-----------KREN-------ALLEFVRVVE
MtCPI-19        --S-SE---N-S-LEIESLARFAVDQHN-AK--------QNSL---------LEFARVVK
FvCPI-2         --AGSE---N-S-LETEALGRFAVDDHN-----------QKQN-------GMLEFVRVVK
FvCPI-12        --IKDI---G-D-PHVQEIAKWGVSEYN--R--------QSHK-------GL-VFQRVIS
FvCPI-8         --IEDI---H-N-PHVVEIAEFAVSELN-----------KQFQ-------KKLVFQSVVR
AtCPI-4         --IKNV---S-D-PDVVAVAKYAIEEHN--K--------ESKE-------KL-VFVKVVE
MtCPI-20        --INNI---N-D-PKVIDVANFAVKEYNNRR--------RKPE-------EKLRLWKVIK
MtCPI-14        --V-DI---N-D-PHVVEIAKFAVTEFN--K--------RITI-------EKLTFENVIH
MtCPI-16        --V-DI---N-D-PHVVDIAKFAVTAYN--K--------RNTV-------GKLAFEKVIS
MtCPI-11        --I-NI---N-D-PRVIEMAGFAIIEHN--N--------QITG-------AKLRFEKLVD
MtCPI-7         --V-DI---N-D-SEVIKVATFAVTEYN--K--------QHTK-------AKLKFEKVIN
MtCPI-4         --I-DV---N-D-PHVMKVTT---------------------------------FEKVIS
MtCPI-9         --V-DV---N-D-PHVIEIATFAVTEYN--N--------QHTE-------AKLVFEKVMS
MtCPI-18        --V-NI---N-D-PYVINITTFAVIEYN--K--------QNTK-------AKLVFEKLLN
MtCPI-10        --V-DI---N-D-PHVIDTGNFAVTKHN--K--------QNNE-------TKLIFEKVID
MtCPI-17        --V-NI---N-D-PHVIDTGNF------------------------------------KVIN
FvCPI-6         --IKDI---N-D-PHVKEIAEFAVSEYN-----------KESG-------KKLELQRVVK
MtCPI-15        --IKDI---N-N-PHVIDIANFAVIEFN-----------KQTG-------AKLKFEKVIK
MtCPI-21        --IKNI---N-D-PHVIDIANYAVTEHD--K--------QAGL-------NL-KLEKVIS
MtCPI-13        --I-NI---Y-E-PYMIDLCNF------------------------------------KIIK
MtCPI-8         --IKNV---K-D-PQVVEIAKFAVTEYG-----------KQSG-------SKLSFVKVIK
AtCPI-5         --ISNV---T-D-PQVVEIGEFAVSEYN-----------KRSE-------SGLKFETVVS
VvCPI-1         --IKNI---S-D-PRVQEIGEFAVTEHN-----------KQAT-------ESLKFQSVVS
MtCPI-3         --IKNL---N-D-PHVIEIANFAVTEYG--K--------QQRF-------YS-KLDKIIK
MtCPI-6         --IKNL---N-D-HHLIEIANFAVTEYG--K--------QQGI-------KQIKLEKITK
MtCPI-22        --IDDI---N-D-PHVTEIANFAVTEYD-----------RRSG-------AKLKFEKVIN
MtCPI-5         --IKDI---N-D-PHVIVIANFAVTEYN-----------KHTG-------ANLKLDKLIK


VcCPI-1         AKTQ--VV--AGI--LYKMELLL-------E--D--EK---GQQV-LFTCSVWNR-----
CrCPI-1         ASTQ--VV--AGV--NVHLELLM-------A--D--DT---GKQT-VVTSTVWSR-----
SbCPI-17        CWSS--PA--GGGANNYWMVLSA-------T--N--YT---GTAG-SYVSTVWGI-----
OsCPI-18        GSVQAAG---AGN--NYRLLLRA--------------A---GGVG-TYEAVVWGV-----
SbCPI-6         SERHP-HR--GGV--RYKMVVTA-------A--D--AS---GATA-QYQVLAWGI-----
OsCPI-17        VDQHPDK---GGV--RYQMVVTA-------A--DAGG----GAAS-PYRAVVWGI-----
OsCPI-14        GQTQP-YN--GGY--NYRLVVTV-------Y--G--GP---NWKTTLYDADVWGI-----
SbCPI-8         GETQP-CN--GGY--NYRLLITV-------S-----G----GKNT-QYEAFVWGI-----
OsCPI-13        GQTQP-CN--GGY--NYRLLVTV-------A-----GP---GATTARYDALMWGI-----
SbCPI-18        GETQPAAV--GNGISDYRLVLNV----------KNTDT---GSTA-LYQCVVRGK-----
OsCPI-15        GETEAAVG--GGT--NYRLVILA-------E--TTPG----GSKA-KFQCVVWGV-----
OsCPI-16        GETEAAAGGAGGT--NYRLAVAV-------TKPD-------GSAA-QYECLVWGV-----
SbCPI-7         GQTEQAVG--GGT--NFRLVVVA-------ARPD--DG---GSTA-QYDCLVWGV-----
VvCPI-3         GWFQ--HL--AGN--IYKLHIKA----------D--LA---GTSG-DYEAIVWKK-----
FvCPI-11        AELQ--MV--EGL--NYHLVIEV-------R--GPERH---NTTE-EYEFTVFTR-----
FvCPI-5         AYNQ--EC--GGAFNHLMLTLEA-------I--D--AN---NVIQ-IYQAIVKQF-----
AtCPI-2         AQKQ--VV--AGL--KYYLRIEV-------T--Q--PN---GSTR-MFDSVVVIQ-----
FvCPI-4         GYYQ--IE--SGT--LYKLVIRV-------K--DENVH---FPTA-EYLAIVLSKGEQWR
FvCPI-10        GYYQ--IE--SGI--LYKLVIRV-------K--DENVH---FPTA-EYQAVVLSN-----
SbCPI-12        GEMM--MS--IGI--DFRLVIDA----------SSTSD---GKHA-NYEARVHER-----
SbCPI-16        GEVY--VS--LGY--LYHLIIDA-------S--D--RD---GKDA-RYTAVVRQSTLGGE
SbCPI-19        GEETLDPE-LVGI--KYHLVIDA-------S--D--GS---GQHR-KYEAVLGEE-----
OsCPI-11        AEEQ--VV--QGS--NYLVVIDA-------A--S--SR---KKTRELYVAVVADL-----
SbCPI-5         GQYQ--VV--EGF--NYRLIIDA-------T--D--SH---GKVA-KYEAVVWEK-----
SbCPI-1         GEEQ--VV--AGT--NYKLVIEA-------T--KGAG----GKIA-TYGAVVYEK-----
SbCPI-2         GEEQ--IV--AGT--KYKLVIQA----------TRAGA---GGNSATYGAVVYEK-----
OsCPI-9         AEKR--FE--AGV-NYYRLTLEA-------S--S--RV---VAKYLRFQAVVYEE-----
SbCPI-4         GEEQ--VV--SGM--RYRLYVDA-------A--D--RA---GRSV-TYVAVIYEQ-----
OsCPI-7         GEKQ--VV--AGV--NYRLTLEA-------S-----SS---GAKDGRYEAVVYEQ-----
```

```
OsCPI-6         GEQQ--VV--SGM--NYRLEIEA-------A--S--GG---GDVTGSYGAVVFEQ-----
OsCPI-8         GEQQ--VV--SGM--NYRLVVSA-------S--D--PA---GATA-SYVAVVYEQ-----
SbCPI-3         GEQQ--VV--SGM--NYRLVLHA-------T--D--AD---GDVA-AYGALVYEQ-----
FvCPI-7         AEIA--QI--LGT--LYHLTIAA-------K--NETSP---TSVA-NYEAGVLVR-----
FvCPI-9         GLAA--NN--SGV--MYLLQIAA-------K--NESLP---IPAQ-NYTTGVWAR-----
OsCPI-10        AQKQ--VV--SGV--AYYLKVAA-------SARDPRGGAAAGGDR-VFDAVVVVK-----
OsCPI-5         AQTQ--VV--SGI--KYYLRIAA-------Q--E-------ADDELVFDAVVVVK-----
OsCPI-4         AQRQ--VV--SGL--KYYLRVAAAEEGAAGQ--N--G----GEPR-VFDAVVVVK-----
SbCPI-11        AQRQ--VV--SGL--KYYLRVAA-------VEEGAQNA---GGER-AFDAVVVVK-----
MtCPI-2         AEQQ--VV--SGM--KYYLNISA-------V--D--HN---GVQR-MFNSVVVVK-----
FvCPI-3         AETQ--VV--SGI--KYYLKIST-------VRNGLNG----GTPL-LFDSQVVVK-----
OsCPI-3         AKRQ--VV--TGT--LHDLMLEV-------V--D--S----GKKS-LYSAKVWVK-----
SbCPI-13        SKRQ--VV--TGT--LHDLILEA-------A--D--A----GKKS-LYRAKVWVK-----
SmCPI-2         AQTQ--VV--SGT--MFHLSIEA-------H--S--EQ---HGTG-VYDAKVWTK-----
SmCPI-3         AQTQ--VV--SGT--MFHLSIEA-------H--S--EQ---HGTG-VYDAKVWTK-----
PpCPI-5         AKEQ--VV--QGK--MYYFTIEV-------M-----EN---GVPK-NYDAKVWVK-----
SmCPI-1         AKSQ--VV--SGV--MYLLTVEA-------T--D--SK---NSVC-SYEAKVWVK-----
SbCPI-15        VRTQ--LV--AGR--MYYFTVEV-------R--EVDG----GASK-LYEAKVWLR-----
SbCPI-20        VRYQ--VV--AGT--MHQFTVKV-------K--E--A----GSTKKLYKAKVWAM-----
SbCPI-14        VRHQ--VV--AGT--MHHFTVQV-------K--EA-G----GGKK-LYEAKVWEK-----
AtCPI-1         AKTQ--VV--AGT--MHHLTVEV-------A--D--G----ETNK-VYEAKVLEK-----
OsCPI-2         VRQQ--VV--GGF--MHYLTVEV-------K--EP-G----GANK-LYEAKVWER-----
PpCPI-4         CHMQ--VV--AGS--MYYLVIEV-------E--E--G----SSIK-LYEAKVWVK-----
PpCPI-1         CRKQ--VV--QGT--MYHLVIEV-------E--E--S----GKLS-QYEAKVWVK-----
PpCPI-2         CHKQ--VV--SGL--MYHFVIEV-------E--E--G----SQLK-QYEAKVWVK-----
PpCPI-3         CHKQ--VV--SGL--MYHFVLEV-------E--Q--G----SQPK-QYEAKVWVK-----
MtCPI-1         AKEQ--VV--AGT--IHHITLEV-------K--D--G----VNKK-VYEAKGLGK-----
OsCPI-1         VKQQ--VV--AGT--LYYFTIEV-------K--E-------GDAKKLYEAKVWEK-----
SbCPI-10        AKTQ--VV--AGT--MYYLTVEV-------K--D--G----EVKK-LYEAKVWEK-----
AtCPI-7         ATEQ--VV--AGK--LYRLTLEV-------I--E--A----GEKK-IYEAKVWVK-----
MtCPI-12        AKKK--VV--SGT--LYDITLET-------K--D--G----GKQK-VYEAKILEK-----
FvCPI-1         TKEQ--VV--SGT--VYYLTIEA-------T--D--G----GQKK-LFEAKVWVK-----
VvCPI-5         VRQQ--VV--AGT--IYYITLDA-------T--D--G----GKKN-KYEAKIWVK-----
AtCPI-3         AREQ--VV--AGT--MYHLTLEA-------K--E--G----DQTK-NFEAKVWVK-----
VvCPI-2         AKEQ--VV--AGK--IYYLTLEA-------I--D--A----GKKK-IYEAKVWVK-----
VvCPI-4         AEEQ--VV--AGT--LHHLTLEV-------I--D--A----GRKK-LYEAKVWVK-----
AtCPI-6         AKEQ--VV--AGT--LHHLTLEI-------L--E--A----GQKK-LYEAKVWVK-----
OsCPI-12        AKEQ--VV--AGT--LHHLTLEA-------L--E--A----GRKK-VYEAKVWVK-----
SbCPI-9         AKEQ--VV--AGT--LHHLTLEA-------I--E--A----GKKK-LYEAKVWVK-----
MtCPI-19        AREQ--VV--AGT--LHHLTLEA-------I--D--A----GEKK-IYEAKVWVK-----
FvCPI-2         AKEQ--VV--AGT--LHHLVVEA-------I--D--G----GKKK-LYEAKVWVK-----
FvCPI-12        GQEQ--SV--AGW--NYKLITSI-------K--D--G----SSIV-NYEVYVYER-----
FvCPI-8         GEKQ--VV--AGE--NYKLVITV-------K--D-------ESSSVNYECVVWEK-----
AtCPI-4         GTTQ--VV--SGT--KYDLKIAA-------K--D--GG---GKIK-NYEAVVVEK-----
MtCPI-20        GESQI-VA--DGV--NYRLTLSA-------T--K--V----YTSN-TYEAIVLE------
MtCPI-14        GESKN-VV--DGT--VYRLTFSA-------H--G--L----STSY-KYRAIVFEK-----
MtCPI-16        GESQN-VI--NGT--NYRLTLSA-------R--Q--I----ITVQ-KYRAIVLEK-----
MtCPI-11        GYIQK-VG--LET--DYHLTISA-------K--N--G----STIN-NYEAVVFYS-----
MtCPI-7         GISNL-GN--NVTIINYRLTLSA-------N--N--G----SASN-NYPAIVLDKSSEIF
MtCPI-4         GVSNV-VD--NGT--RYSLTLSA-------N--N--G----SASN-NYDTIVLEK-----
MtCPI-9         GVSNV-VD--NET--RYSLTLSA-------K--N--G----SASN-NYDTIVLEK-----
MtCPI-18        GVIDT-LN--DGI--NFRLTLSA-------N--N--G----STSN-KYGAIVLEK-----
MtCPI-10        DVVDI-VN--DKT--KYRLTLSA-------N--N--G----STSN-NYEIIVLDK-----
MtCPI-17        GVIDI-MN--NET--KYRLTLSA-------N--N--G----STSN-NYGAIVLEK-----
FvCPI-6         GETQ--VV--AGQ--NFRLLLAV-------K--D-------DTTAAKYVGVVYER-----
MtCPI-15        GESRLALA--EDA--IYRLIIST-------S--N-------SVPN-IFQAVVIEN-----
MtCPI-21        GETK--VV--DGI--IYCLNITA-------S--D--G----SASN-KYNLAVLEK-----
MtCPI-13        AESM--VV--EWI--NYRLILSA-------N--D--G----SHSK-TYKAVVWET-----
MtCPI-8         GETQ--VV--SGT--NYRLVLAA-------K-----VV---SATK-NYEALVWEK-----
AtCPI-5         GETQ--VV--SGT--NYRLKVAA-------N--D--GD---GVSK-NYLAIVWDK-----
VvCPI-1         GETQ--VV--SGT--NYRLVVVA-------E--D--G----GVSN-KYEAVVWEK-----
MtCPI-3         GESQ--AV--DGT--NYRFILYV-------I--I--G----SESY-PYKAFVHEN-----
MtCPI-6         GETQ--VV--DGT--NYRLLLSA-------I--I--E----SMSY-PYQAIVYEN-----
MtCPI-22        GESQ--VV--AGT--NYRLTLSA-------S--D-------GSYSKNYEAVVWEK-----
MtCPI-5         GESQ--VT--SGI--YYDLILSA-------G--D-------GSHSNIYKALVWEK-----
                                  .

VcCPI-1         --P-------------------------W-NTGQNGGDDHHNHITKFH------------
CrCPI-1         --P-------------------------W-LASKNDAAQPATQITALT------------
SbCPI-17        --P------------------------GS-----------ESKTWKLL--------T---
OsCPI-18        --P---------------------GSTGW--------------TWKVL--------S---
SbCPI-6         --P-------------------------G------------TYQWMLL--------E---
OsCPI-17        --P-------------------------E------------THAWMLL--------E---
OsCPI-14        --P------------------------GT------------TTHWWFR--------S---
SbCPI-8         --L-------------------------G------------TTSWKLL--------S---
OsCPI-13        --L-------------------------G------------TTNWKLL--------S---
SbCPI-18        --P------------------------GS-----------RSTTWVLL--------S---
OsCPI-15        --P------------------------GS-----------RANTWKLL--------S---
OsCPI-16        --P------------------------GS-----------RLDTWKLR--------R---
SbCPI-7         --P------------------------GS-----------RSDTWKLR--------R---
VvCPI-3         ----------------------------H------------NQCNELI--------N---
FvCPI-11        --Y-------------------------W------------DKIQSRSAMVDSGGRD---
FvCPI-5         ----------------------------W------------IAKRPLLLLLFGLVADNGN
```

```
AtCPI-2         --P-------------------------W------------LHSKQLL--------G---
FvCPI-4         PPPDFCCGLGTGGNLRENFILVAAGCSSW------------PLMQRII--------GDIW
FvCPI-10        ----------------------------G------------SISEKLI--------S---
SbCPI-12        --D-------------------------W------------MDGISLL--------S---
SbCPI-16        LSL-------------------------W---------------------------S---
SbCPI-19        ----------------------------W------------IGRIILI--------S---
OsCPI-11        --V-------------------------G------------ATTYQLS--------S---
SbCPI-5         --E-------------------------W------------ENFLQLT--------S---
SbCPI-1         --V--------------------------------------DNTRQLL--------S---
SbCPI-2         --V--------------------------------------DKTRQLL--------S---
OsCPI-9         -----------------------------------------GDEHKLV--------S---
SbCPI-4         --V-------------------------W------------TNTRKLA--------S---
OsCPI-7         -DP-------------------------R------------SNARKLV--------S---
OsCPI-6         --E-------------------------W------------SNTRKLI--------S---
OsCPI-8         --S-------------------------W------------TNTRQLT--------S---
SbCPI-3         --S-------------------------W------------TNTRELM--------S---
FvCPI-7         ----------------------------H------------GQDPKLT--------A---
FvCPI-9         ----------------------------Y------------REPLMLL--------A---
OsCPI-10        --A-------------------------W------------LKSKELV--------S---
OsCPI-5         --A-------------------------W------------VPSREMV--------S---
OsCPI-4         --P-------------------------W------------LESRTLL--------T---
SbCPI-11        --P-------------------------W------------LESRTLL--------T---
MtCPI-2         --P-------------------------W-----------LHQYKKVI--------Q---
FvCPI-3         --P-------------------------W------------LRSKRLL--------N---
OsCPI-3         --P-------------------------W------------LDFKAVV--------E---
SbCPI-13        --P-------------------------W------------EDFKSVV--------E---
SmCPI-2         --P-------------------------W------------ESFKKLE--------E---
SmCPI-3         --P-------------------------W------------ESFKKLE--------D---
PpCPI-5         --P-------------------------W------------EGYKELE--------S---
SmCPI-1         --E-------------------------W------------ENFRRLM--------S---
SbCPI-15        --P-------------------------W------------ENFKGLH--------A---
SbCPI-20        ----------------------------W------------KNFKQLQ------------
SbCPI-14        --V-------------------------W------------ENFKQLQ--------S---
AtCPI-1         --A-------------------------W------------ENLKQLE--------S---
OsCPI-2         --A-------------------------W------------ENFKQLQ--------D---
PpCPI-4         --P-------------------------W------------QNFKKLE--------E---
PpCPI-1         --P-------------------------W------------ENFKKLE--------D---
PpCPI-2         --P-------------------------G------------GSSRKLE--------E---
PpCPI-3         --P-------------------------G------------GASKKLE--------E---
MtCPI-1         --V----------------------------------------LDELQ--------G---
OsCPI-1         --P-------------------------W------------MDFKELQ--------E---
SbCPI-10        --P-------------------------W------------ENFKELQ--------E---
AtCPI-7         --P-------------------------W------------MNFKQLQ--------E---
MtCPI-12        --P-------------------------W------------LNFKEVQ--------E---
FvCPI-1         --P-------------------------W------------LNFKEVQ--------E---
VvCPI-5         --P-------------------------W------------LNFKELQ--------E---
AtCPI-3         --P-------------------------W------------MNFKQLQ--------E---
VvCPI-2         --P-------------------------W------------MNFKELQ--------E---
VvCPI-4         --P-------------------------W------------MNFKELQ--------E---
AtCPI-6         --P-------------------------W------------LNFKELQ--------E---
OsCPI-12        --P-------------------------W------------LDFKELQ--------E---
SbCPI-9         --P-------------------------W------------LNFKELQ--------D---
MtCPI-19        --P-------------------------W------------MNFKELT--------E---
FvCPI-2         --P-------------------------W------------LNFKQVQ--------E---
FvCPI-12        ----------------------------YVLAALESSQEDLSVDGALV--------EKAK
FvCPI-8         --E-------------------------QHGRSYTNNEEKVTLFAILE--------E---
AtCPI-4         --L-------------------------W------------LHSKSLE--------S---
MtCPI-20        ----------------------------W----------SLQHLRNLT--------S---
MtCPI-14        --P-------------------------S------------EHYRKLA--------S---
MtCPI-16        --P-------------------------L------------VHFRNLV--------S---
MtCPI-11        --P--------------------------------------IFELSLK--------S---
MtCPI-7         SVP------------------------VYQKTCLDTFGEHETYCKELS--------N---
MtCPI-4         --S-------------------------S------------ENFSLIA------------
MtCPI-9         --S-------------------------S------------ENFSLIA------------
MtCPI-18        --A-------------------------Y------------KHFRKLT--------A---
MtCPI-10        --P-------------------------L------------EHLRNLT--------A---
MtCPI-17        --P-------------------------L------------EHLRNLT--------A---
FvCPI-6         --V-------------------------W------------EHTRKLL--------S---
MtCPI-15        --K-------------------------L------------NHDRNLT--------S---
MtCPI-21        --L-------------------------E------------QHFRNLT--------S---
MtCPI-13        --T-------------------------D------------RDAKNLT--------S---
MtCPI-8         --P-------------------------W------------LHLKNLT--------S---
AtCPI-5         --P-------------------------W------------MKFRNLT--------S---
VvCPI-1         --P-------------------------W------------MGFRNLT--------S---
MtCPI-3         --A-------------------------S------------KSFKKLI--------S---
MtCPI-6         --R-------------------------L------------KNFKKLI--------S---
MtCPI-22        --I-------------------------W------------QHFRNLT--------S---
MtCPI-5         --T-------------------------W--------------QHNLI--------S---


VcCPI-1         Y--QYIDP----------------------------------------------------
CrCPI-1         F--KPLDG------TLE-------------------------------------------
SbCPI-17        F--EGTM-----------------------------------------------------
```

```
OsCPI-18        F--RRVAGDQN-------------------------------------------------
SbCPI-6         F--KKIN-----------------------------------------------------
OsCPI-17        F--NRIN-----------------------------------------------------
OsCPI-14        F--TPKRS----------------------------------------------------
SbCPI-8         F--TPKY-----------------------------------------------------
OsCPI-13        F--TLAAN----------------------------------------------------
SbCPI-18        F--VPYTQ------AP--------------------------------------------
OsCPI-15        F--KAI------------------------------------------------------
OsCPI-16        F--RRIQL------P---------------------------------------------
SbCPI-7         F--RKIVQ------G---------------------------------------------
VvCPI-3         Y--HKILL------TKL-------------------------------------------
FvCPI-11        F--RLYSG----------------------------------------------GTGRCL
FvCPI-5         W--DLIKL---------------------------------------------------I
AtCPI-2         F--TPVVS------PVY-------------------------------------------
FvCPI-4         W--SRLVA------AAAPRADRPSCGR-----RLI-------------------------
FvCPI-10        F--TQITN----------------------------------------------------
SbCPI-12        F--KPAK-----------------------------------------------------
SbCPI-16        F--KPAN-----------------------------------------------------
SbCPI-19        F--NPAR-----------------------------------------------------
OsCPI-11        F--KLATK----------------------------------------------------
SbCPI-5         F--KPAN-----------------------------------------------------
SbCPI-1         F--APEN-----------------------------------------------------
SbCPI-2         F--SPAN-----------------------------------------------------
OsCPI-9         F--VPIH-----------------------------------------------------
SbCPI-4         F--TLASR------AH--------------------------------------------
OsCPI-7         F--EPIH-----------------------------------------------------
OsCPI-6         F--DKNHN------F---------------------------------------------
OsCPI-8         F--KPAAA------H---------------------------------------------
SbCPI-3         F--ASAN-----------------------------------------------------
FvCPI-7         F--HIAKC----------------------------------------------------
FvCPI-9         F--HKTNE------TTVE------------------------------------------
OsCPI-10        F--TPASS------TK--------------------------------------------
OsCPI-5         F--VPAAE------LPGY------------------------------------------
OsCPI-4         F--APAAD------SPNES-----------------------------------------
SbCPI-11        F--APAAA------K---------------------------------------------
MtCPI-2         T--RHIMG------ILVP------------------------------------------
FvCPI-3         F--APHSP------TENPKGVCNKPRKMSRRNDDVAGSPVINDITDAFLWSATIGATKAA
OsCPI-3         F--RHVGD--------------------------------------------------SQ
SbCPI-13        F--RLVGD------SESESESSVTS-----------------------------------
SmCPI-2         F--KPSKQ-------------------------------------------------ETA
SmCPI-3         F--KPSKQ------GSDFCFSCCSPEA--------------------------------A
PpCPI-5         F--LPSAP----------------------------------------------------
SmCPI-1         F--D--------------------------------------------------------
SbCPI-15        F--DPVAD------AA--------------------------------------------
SbCPI-20        ---MPQSP------DLRYKLFHRCSTK-----NRRE------------------------
SbCPI-14        F--QPVGD------------AAVA------------------------------------
AtCPI-1         F--NHLHD------V---------------------------------------------
OsCPI-2         F--KPLDD------ATA-------------------------------------------
PpCPI-4         F--KLKDAGVTSADLGVRTGGPHSTG--------------------------RGISAPPS
PpCPI-1         F--KPKEQ-------EVFTSADLGVR------------------PGGPFMVLGGNSAPPT
PpCPI-2         F--KSVDGGLTSADTGVKTEEICHNAG-------------------VPLTKVSQHSCVPV
PpCPI-3         F--KPKDA----------------------------------------------------
MtCPI-1         G--SRVQA------------RRRCACA---------------------------------
OsCPI-1         F--KPVDA------SANA------------------------------------------
SbCPI-10        F--KPVEE------------GASA------------------------------------
AtCPI-7         F--KNIIP------SFTISDLGFKP------------------------------DGNGF
MtCPI-12        F--KLISQ------NDDAPSVSST------------------------------------
FvCPI-1         F--KPVAD------V---------------------------------------------
VvCPI-5         F--KPIGD------------EPATASA---------------------------------
AtCPI-3         F--KESSS----------------------------------------------------
VvCPI-2         F--KHSQE------TKSFAPSDLGVKQ----------------------------DGHGS
VvCPI-4         F--KHAGD------VPTLTPSDLGVKK----------------------------DGHGP
AtCPI-6         F--KPASD------APAITSSDLGCKQ----------------------------GEHES
OsCPI-12        F--RNTGD------ATTFTNADLGAKK----------------------------GGHEP
SbCPI-9         F--SHKGE------ATTFTNADLGAKK----------------------------GEHEP
MtCPI-19        F--KHAGD----GHAPSFTTSDLGVIK----------------------------DGHKP
FvCPI-2         F--KHAGEPETVSGTPSFTSSDLGVKQ----------------------------GGHAP
FvCPI-12        FLMRCFESCRWSYVPREADKVAHKV-----------------------------------
FvCPI-8         F--DDIEN------FNSQEKHSFTLGL-----KAFNYEIEDSPERWDWNTVGHKRSCEPV
AtCPI-4         F--KAL------------------------------------------------------
MtCPI-20        F--KLIQP--------------------------------------------------IP
MtCPI-14        F--ELVHA----------------------------------------------------
MtCPI-16        F--ELINA----------------------------------------------------
MtCPI-11        F--VLIHA----------------------------------------------------
MtCPI-7         F--KYMHNFVSDTFFDVFKQVGASMKK----------------------------ERHVN
MtCPI-4         FALNPHA-----------------------------------------------------
MtCPI-9         FALNPHA-----------------------------------------------------
MtCPI-18        F--APVQH------A---------------------------------------------
MtCPI-10        F--ALISR------A---------------------------------------------
MtCPI-17        F--LHLFLMFKLSNVVFYIII---------------------------------------
FvCPI-6         F--DQVKK----------------------------------------------------
MtCPI-15        F--ILTH-----------------------------------------------------
MtCPI-21        F--VPLQN----------------------------------------------------
```

```
MtCPI-13        F--APVVN------Y---------------------------------------------
MtCPI-8         F--KPVA-----------------------------------------------------
AtCPI-5         F--EPANN------GRFL------------------------------------------
VvCPI-1         F--TRV------------------------------------------------------
MtCPI-3         F--VSID-----------------------------------------------------
MtCPI-6         F--VPIN-----------------------------------------------------
MtCPI-22        F--VPVHA----------------------------------------------------
MtCPI-5         F--VPANN----------------------------------------------------


VcCPI-1         ------------------------------------------------------------
CrCPI-1         ------------------------------------------------------------
SbCPI-17        ------------------------------------------------------------
OsCPI-18        ------------------------------------------------------------
SbCPI-6         ------------------------------------------------------------
OsCPI-17        ------------------------------------------------------------
OsCPI-14        ------------------------------------------------------------
SbCPI-8         ------------------------------------------------------------
OsCPI-13        ------------------------------------------------------------
SbCPI-18        ------------------------------------------------------------
OsCPI-15        ------------------------------------------------------------
OsCPI-16        ------------------------------------------------------------
SbCPI-7         ------------------------------------------------------------
VvCPI-3         ------------------------------------------------------------
FvCPI-11        LWRMWNVLDHGVRMAHQILVSEK-------------------------------------
FvCPI-5         DNRGVFGPGPTDNRCFGRRIKIQKTQRCSLEEEILSTLHLPLK-----------------
AtCPI-2         ------------------------------------------------------------
FvCPI-4         ------------------------------------------------------------
FvCPI-10        ------------------------------------------------------------
SbCPI-12        ------------------------------------------------------------
SbCPI-16        ------------------------------------------------------------
SbCPI-19        ------------------------------------------------------------
OsCPI-11        ------------------------------------------------------------
SbCPI-5         ------------------------------------------------------------
SbCPI-1         ------------------------------------------------------------
SbCPI-2         ------------------------------------------------------------
OsCPI-9         ------------------------------------------------------------
SbCPI-4         ------------------------------------------------------------
OsCPI-7         ------------------------------------------------------------
OsCPI-6         ------------------------------------------------------------
OsCPI-8         ------------------------------------------------------------
SbCPI-3         ------------------------------------------------------------
FvCPI-7         ------------------------------------------------------------
FvCPI-9         ------------------------------------------------------------
OsCPI-10        ------------------------------------------------------------
OsCPI-5         ------------------------------------------------------------
OsCPI-4         ------------------------------------------------------------
SbCPI-11        ------------------------------------------------------------
MtCPI-2         ------------------------------------------------------------
FvCPI-3         AEGTYYAIQRGSVSSHDFECTLKRMGEEGVYWGTTAGLFVTTEYGLESVRGRSDWKNTLI
OsCPI-3         SQSATAADDNAGQDTADPTVASRNDLHNTENNKVSVVLSTFSQTYSV-------------
SbCPI-13        ---DVSSGQAIAKLSLEADIAQEEAHLHTIENDGLSGDFASSS-----------------
SmCPI-2         GAAIALDDPVVINEAAEHALKGLQQRSNSLIPYELDHVVKAQAEEVEIDTHRPSDKGTNA
SmCPI-3         GAAIALDDPVVINEAAEHALKGLQQRSNSLIPYELDHVVKAQAEE-----------GTNA
PpCPI-5         ----------------SHYDSAVDSDAKSGDCYEKSEIAT--------------------
SmCPI-1         ------------------------------------------------------------
SbCPI-15        ------------------------------------------------------------
SbCPI-20        ------------------------------------------------------------
SbCPI-14        ------------------------------------------------------------
AtCPI-1         ------------------------------------------------------------
OsCPI-2         ------------------------------------------------------------
PpCPI-4         GKQSWPTDDLVVQEAAEHAMKMLQQGSNSLASYELSEIVSADAELSD---------ESAD
PpCPI-1         DKQSVPTDDPVVQEAAEHVIKTLQMGSNSLSTYELNEILSAEAELND---------ETAQ
PpCPI-2         GKQIVPTEAPIVQEAAEHAIKMLQQGSNSLSSYELREIVSAEAQLKE---------GSAV
PpCPI-3         ------------------------------------------------------------
MtCPI-1         ------------------------------------------------------------
OsCPI-1         ------------------------------------------------------------
SbCPI-10        ------------------------------------------------------------
AtCPI-7         DWRSVSTNNPEVQEAAKHAMKSLQQKSNSLFPYKLIDIILARAKVVE---------ERVK
MtCPI-12        ------------------------------------------------------------
FvCPI-1         ------------------------------------------------------------
VvCPI-5         ------------------------------------------------------------
AtCPI-3         ------------------------------------------------------------
VvCPI-2         EWQVVPTNDPEVQDAANHVVKSIQMRSNSIFRYELLEILLAKAKVIE---------GSAK
VvCPI-4         GWQEVPAHDPEVQNAANHAIKTLQQRSNSIFPHELQEILLAKAEVSQ---------NLVK
AtCPI-6         GWREVPGDDPEVKHVAEQAVKTIQQRSNSLFPYELLEVVHAKAEVTG---------EAAK
OsCPI-12        GWRDVPVHDPVVKDAADHAVKSIQQRSNSLFPYELLEIVRAKAEVVE---------DFAK
SbCPI-9         GWREVPIEDPVVKDAAHHAVKSIQERSNSLFPYELLEIVRAKAQVVE---------DFAK
MtCPI-19        GWQSVPAHDPQVQDAANHAIKTIQQRSNSLVPYELHEVTDAKAEVID---------DTAK
FvCPI-2         GWQDVHPHDPQVQDAANHAVKSLQQKSNSLFPYELQEVVHAKAEVME---------EHAK
FvCPI-12        ------------------------------------------------------------
FvCPI-8         TNIKNQGNCVRNTTEAASRVEAPSPAVSKLAK----------------------------
AtCPI-4         ------------------------------------------------------------
```

```
MtCPI-20        AHEGLRHSKGDANSKFFHGCISQGRRSNNISSFMVNGAACGRCVSAPSSLYSEPFNGKQL
MtCPI-14        ------------------------------------------------------------
MtCPI-16        ------------------------------------------------------------
MtCPI-11        ------------------------------------------------------------
MtCPI-7         FLSDLPDGRSTLRPTNVMVYEWVGGKHAFVDLTGVSPLV---------------------
MtCPI-4         ------------------------------------------------------------
MtCPI-9         ------------------------------------------------------------
MtCPI-18        ------------------------------------------------------------
MtCPI-10        ------------------------------------------------------------
MtCPI-17        ------------------------------------------------------------
FvCPI-6         ------------------------------------------------------------
MtCPI-15        ------------------------------------------------------------
MtCPI-21        ------------------------------------------------------------
MtCPI-13        ------------------------------------------------------------
MtCPI-8         ------------------------------------------------------------
AtCPI-5         ------------------------------------------------------------
VvCPI-1         ------------------------------------------------------------
MtCPI-3         ------------------------------------------------------------
MtCPI-6         ------------------------------------------------------------
MtCPI-22        ------------------------------------------------------------
MtCPI-5         ------------------------------------------------------------


VcCPI-1         --------------------------------------------
CrCPI-1         --------------------------------------------
SbCPI-17        --------------------------------------------
OsCPI-18        --------------------------------------------
SbCPI-6         --------------------------------------------
OsCPI-17        --------------------------------------------
OsCPI-14        --------------------------------------------
SbCPI-8         --------------------------------------------
OsCPI-13        --------------------------------------------
SbCPI-18        --------------------------------------------
OsCPI-15        --------------------------------------------
OsCPI-16        --------------------------------------------
SbCPI-7         --------------------------------------------
VvCPI-3         --------------------------------------------
FvCPI-11        --------------------------------------------
FvCPI-5         --------------------------------------------
AtCPI-2         --------------------------------------------
FvCPI-4         --------------------------------------------
FvCPI-10        --------------------------------------------
SbCPI-12        --------------------------------------------
SbCPI-16        --------------------------------------------
SbCPI-19        --------------------------------------------
OsCPI-11        --------------------------------------------
SbCPI-5         --------------------------------------------
SbCPI-1         --------------------------------------------
SbCPI-2         --------------------------------------------
OsCPI-9         --------------------------------------------
SbCPI-4         --------------------------------------------
OsCPI-7         --------------------------------------------
OsCPI-6         --------------------------------------------
OsCPI-8         --------------------------------------------
SbCPI-3         --------------------------------------------
FvCPI-7         --------------------------------------------
FvCPI-9         --------------------------------------------
OsCPI-10        --------------------------------------------
OsCPI-5         --------------------------------------------
OsCPI-4         --------------------------------------------
SbCPI-11        --------------------------------------------
MtCPI-2         --------------------------------------------
FvCPI-3         AGAVTGAVMSAVNKNNKDKILTDAIVGGAVATAASFVRNFRTGK
OsCPI-3         --------------------------------------------
SbCPI-13        --------------------------------------------
SmCPI-2         LDLVLKVKRGSREEHVNAKLHRGDTGWTLTSAHVL---------
SmCPI-3         LDLVLKVKRGSREEHVNAKLHRGDTGWTLTSAHVL---------
PpCPI-5         --------------------------------------------
SmCPI-1         --------------------------------------------
SbCPI-15        --------------------------------------------
SbCPI-20        --------------------------------------------
SbCPI-14        --------------------------------------------
AtCPI-1         --------------------------------------------
OsCPI-2         --------------------------------------------
PpCPI-4         FELLLKIKRGAKEEHFKSEIHRTGDGDWSVKHVTLQ--------
PpCPI-1         FDLLLKTKLGAKEQVFKAEVSRTGDGDWTVKHATIQ--------
PpCPI-2         FDLLLKTKRGSKEENFKSEVHRAEDGSWSVKHATLE--------
PpCPI-3         --------------------------------------------
MtCPI-1         ---------VKRLTRYFITLHNSSCLLF----------------
OsCPI-1         --------------------------------------------
SbCPI-10        --------------------------------------------
AtCPI-7         FELLLKLERGNKLEKFMVEVMKDQTGKYE---------------
MtCPI-12        --------------------------------------------
```

```
FvCPI-1         --------------------------------------------
VvCPI-5         --------------------------------------------
AtCPI-3         --------------------------------------------
VvCPI-2         FDLLLKLKWGSKDVKFKAEVNKNIEGKFCSTQWKEDHL------
VvCPI-4         FDMLLKVKRGDKEEKYKVEVHKNNEGAYQLNQMAP-DHS-----
AtCPI-6         YNMLLKLKRGEKEEKFKVEVHKNHEGALHLNHAEQ-HHD-----
OsCPI-12        FDILMKLKRGNKEEKFKAEVHKNLEGAFVLNQMQQ-EHDESSSQ
SbCPI-9         FDILMKLKRGSKEEKIKAEVHKSLEGAFVLNKHQPAEHEESSSQ
MtCPI-19        FNLLLKVKRGQKEEKFKVEVHKNSEGNFHLNQMEA-DNS-----
FvCPI-2         FNMLLKLKRGDKEEKYKVEVHKNNEGAYNLNQMEV-EH------
FvCPI-12        --------------------------------------------
FvCPI-8         --------------------------------------------
AtCPI-4         --------------------------------------------
MtCPI-20        RPCLANFEDQLLLADFDL--------------------------
MtCPI-14        --------------------------------------------
MtCPI-16        --------------------------------------------
MtCPI-11        --------------------------------------------
MtCPI-7         --------------------------------------------
MtCPI-4         --------------------------------------------
MtCPI-9         --------------------------------------------
MtCPI-18        --------------------------------------------
MtCPI-10        --------------------------------------------
MtCPI-17        --------------------------------------------
FvCPI-6         --------------------------------------------
MtCPI-15        --------------------------------------------
MtCPI-21        --------------------------------------------
MtCPI-13        --------------------------------------------
MtCPI-8         --------------------------------------------
AtCPI-5         --------------------------------------------
VvCPI-1         --------------------------------------------
MtCPI-3         --------------------------------------------
MtCPI-6         --------------------------------------------
MtCPI-22        --------------------------------------------
MtCPI-5         --------------------------------------------
```

I. I51 Serine Carboxypeptidase Y Inhibitors (SCPYInh).

```
SmSCPYI-11      ------------------------------------------------------------
PpSCPYI-3       ------------------------------------------------------------
MpSCPYI-1       ------------------------------------------------MRRGGPEELPLL
SmSCPYI-15      ------------------------------------------------------------
SmSCPYI-14      ------------------------------------------------------------
SmSCPYI-16      ------------------------------------------------------------
SmSCPYI-17      ------------------------------------------------------------
SmSCPYI-18      ------------------------------------------------------------
SmSCPYI-1       ------------------------------------------------------------
SmSCPYI-6       ------------------------------------------------------------
SmSCPYI-2       ------------------------------------------------------------
SmSCPYI-10      ------------------------------------------------------------
SmSCPYI-8       ------------------------------------------------------------
SmSCPYI-9       ------------------------------------------------------------
SmSCPYI-5       ------------------------------------------------------------
SmSCPYI-7       ------------------------------------------------------------
PpSCPYI-2       -------------------------------------------------------MYVSV
MpSCPYI-2       ------------------------------------------------------------
CsSCPYI-1       ------------------------------------------------------------
SmSCPYI-12      ------------------------------------------------------------
SmSCPYI-13      ------------------------------------------------------------
SmSCPYI-4       ------------------------------------------------------------
AtSCPYI-5       ------------------------------------------------------------
VvSCPYI-1       ------------------------------------------------------------
FvSCPYI-1       ------------------------------------------------------------
MtSCPYI-8       ------------------------------------------------------------
AtSCPYI-6       ------------------------------------------------------------
MtSCPYI-1       ------------------------------------------------------------
AtSCPYI-1       ------------------------------------------------------------
FvSCPYI-3       ------------------------------------------------------------
VvSCPYI-6       ------------------------------------------------------------
MtSCPYI-5       ------------------------------------------------------------
VvSCPYI-5       ------------------------------------------------------------
OsSCPYI-13      ------------------------------------------------------------
OsSCPYI-15      ------------------------------------------------------------
SbSCPYI-9       ------------------------------------------------------------
SbSCPYI-13      ------------------------------------------------------------
SbSCPYI-11      ------------------------------------------------------------
OsSCPYI-5       ------------------------------------------------------------
SbSCPYI-7       ------------------------------------------------------------
OsSCPYI-4       ------------------------------------------------------------
OsSCPYI-12      ------------------------------------------------------------
SbSCPYI-5       ------------------------------------------------------------
OsSCPYI-10      ------------------------------------------------------------
SbSCPYI-17      ------------------------------------------------------------
OsSCPYI-3       ------------------------------------------------------------
SbSCPYI-6       ------------------------------------------------------------
SbSCPYI-10      ------------------------------------------------------------
OsSCPYI-11      ------------------------------------------------------------
SbSCPYI-2       ------------------------------------------------------------
MtSCPYI-2       ------------------------------------------------------------
OsSCPYI-17      ------------------------------------------------------------
SbSCPYI-8       ------------------------------------------------------------
OsSCPYI-6       ------------------------------------------------------------
SbSCPYI-12      ------------------------------------------------------------
OsSCPYI-16      ------------------------------------------------------------
SbSCPYI-1       ------------------------------------------------------------
OsSCPYI-14      ------------------------------------------------------------
SbSCPYI-14      ------------------------------------------------------------
MtSCPYI-4       ------------------------------------------------------------
MtSCPYI-3       ------------------------------------------------------------
MtSCPYI-6       ------------------------------------------------------------
FvSCPYI-5       ------------------------------------------------------------
AtSCPYI-2       ------------------------------------------------------------
AtSCPYI-4       ------------------------------------------------------------
SbSCPYI-3       MKFHRLTTARSGSAACIGLSTPTPTPTGMDAGRFPTPLAAHGCARISSTTTDDDNSITQA
FvSCPYI-2       ------------------------------------------------------------
VvSCPYI-2       ------------------------------------------------------------
OsSCPYI-2       -----------------------MTGSSCPADSSQLLYPRRGAPTTTTTSRPPLHPLIAH
SbSCPYI-15      ------------------------------------------------------------
OsSCPYI-7       ------------------------------------------------------------
OsSCPYI-8       ------------------------------------------------------------
VvSCPYI-3       ------------------------------------------------------------
PpSCPYI-1       ------------------------------------------------------------
PpSCPYI-4       ------------------------------------------------------------
PpSCPYI-5       ------------------------------------------------------------
PpSCPYI-6       ------------------------------------------------------------
SmSCPYI-3       ------------------------------------------------------------
MtSCPYI-7       ------------------------------------------------------------
AtSCPYI-3       ------------------------------------------------------------
FvSCPYI-4       -------------MRCRPLISQPGAPELLSSRVFSYEDGGRVLVQMDLHTLKTLRDGSCV
```

```
VvSCPYI-4       ------------------------------------------------------------
OsSCPYI-1       ------------------------------------------------------------
SbSCPYI-4       ------------------------------------------------------------
OsSCPYI-9       ------------------------------------------------------------
SbSCPYI-16      ------------------------------------------------------------


SmSCPYI-11      ------------------------------------------------------------
PpSCPYI-3       ------------------------------------------------------------
MpSCPYI-1       NKPANGKYGAKTLDDTTSTASNAVNAGDAILTALHGQRNKVLSARVNNESMQSDMDVSER
SmSCPYI-15      ------------------------------------------------------------
SmSCPYI-14      --------------------------------------------------MWSQDLSIWS
SmSCPYI-16      ------------------------------------------------------------
SmSCPYI-17      ------------------------------------------------------------
SmSCPYI-18      ------------------------------------------------------------
SmSCPYI-1       ------------------------------------------------------------
SmSCPYI-6       ------------------------------------------------------------
SmSCPYI-2       ------------------------------------------------------------
SmSCPYI-10      ------------------------------------------------------------
SmSCPYI-8       ------------------------------------------------------------
SmSCPYI-9       ------------------------------------------------------------
SmSCPYI-5       ------------------------------------------------------------
SmSCPYI-7       ------------------------------------------------------------
PpSCPYI-2       LLLLLLTMAAGIARYGSDALLSVEADNVLMREPSNDPVYREDSRELLHGNAFLNEEESPY
MpSCPYI-2       ------------------------------------------MADPGGVINPNSVQLCKL
CsSCPYI-1       ---------------------------------------MRFISAGLLVLLVALSASTAS
SmSCPYI-12      ------------------------------------------------------------
SmSCPYI-13      ------------------------------------------------------------
SmSCPYI-4       ------------------------------------------------------------
AtSCPYI-5       ------------------------------------------------------------
VvSCPYI-1       ------------------------------------------------------------
FvSCPYI-1       ------------------------------------------------------------
MtSCPYI-8       ------------------------------------------------------------
AtSCPYI-6       ------------------------------------------------------------
MtSCPYI-1       ------------------------------------------------------------
AtSCPYI-1       ------------------------------------------------------------
FvSCPYI-3       ------------------------------------------------------------
VvSCPYI-6       ------------------------------------------------------------
MtSCPYI-5       ------------------------------------------------------------
VvSCPYI-5       ------------------------------------------------------------
OsSCPYI-13      ------------------------------------------------------------
OsSCPYI-15      ------------------------------------------------------------
SbSCPYI-9       ------------------------------------------------------------
SbSCPYI-13      ------------------------------------------------------------
SbSCPYI-11      ------------------------------------------------------------
OsSCPYI-5       ------------------------------------------------------------
SbSCPYI-7       ------------------------------------------------------------
OsSCPYI-4       ------------------------------------------------------------
OsSCPYI-12      ------------------------------------------------------------
SbSCPYI-5       ------------------------------------------------------------
OsSCPYI-10      ------------------------------------------------------------
SbSCPYI-17      ------------------------------------------------------------
OsSCPYI-3       ------------------------------------------------------------
SbSCPYI-6       ------------------------------------------------------------
SbSCPYI-10      ------------------------------------------------------------
OsSCPYI-11      ------------------------------------------------------------
SbSCPYI-2       ------------------------------------------------------------
MtSCPYI-2       ------------------------------------------------------------
OsSCPYI-17      ------------------------------------------------------------
SbSCPYI-8       ------------------------------------------------------------
OsSCPYI-6       ------------------------------------------------------------
SbSCPYI-12      ------------------------------------------------------------
OsSCPYI-16      ------------------------------------------------------------
SbSCPYI-1       ------------------------------------------------------------
OsSCPYI-14      ------------------------------------------------------------
SbSCPYI-14      ------------------------------------------------------------
MtSCPYI-4       ------------------------------------------------------------
MtSCPYI-3       ------------------------------------------------------------
MtSCPYI-6       ------------------------------------------------------------
FvSCPYI-5       ------------------------------------------------------------
AtSCPYI-2       ------------------------------------------------------------
AtSCPYI-4       ------------------------------------------------------------
SbSCPYI-3       CPITTSRGTSTPPPPAGPPGPHLPPRPLCLLARSRSSISRKHPHRQESLCSGLERRRTTT
FvSCPYI-2       ------------------------------------------------------------
VvSCPYI-2       ------------------------------------------------------------
OsSCPYI-2       APYDVVLHRSVVLSISSARDQAELASFWCYIRLGGGASAKRAAGEDDRSRSSLTAARGGG
SbSCPYI-15      ------------------------------------------------------------
OsSCPYI-7       ------------------------------------------------------------
OsSCPYI-8       ------------------------------------------------------------
VvSCPYI-3       ------------------------------------------------------------
PpSCPYI-1       ------------------------------------------------------------
PpSCPYI-4       ------------------------------------------------------------
PpSCPYI-5       ------------------------------------------------------------
```

```
PpSCPYI-6       ------------------------------------------------------------
SmSCPYI-3       ------------------------------------------------------------
MtSCPYI-7       ------------------------------------------------------------
AtSCPYI-3       ------------------------------------------------------------
FvSCPYI-4       FLQGSQSVTQVLPLECFMVLIAKKLWGENDSHIGSFVDRCLAVTDGCDRAIWMEETEGWW
VvSCPYI-4       ------------------------------------------------------------
OsSCPYI-1       ------------------------------------------------------------
SbSCPYI-4       ------------------------------------------------------------
OsSCPYI-9       ------------------------------------------------------------
SbSCPYI-16      ------------------------------------------------------------


SmSCPYI-11      ------MADQ----------------------------------------FRLV------
PpSCPYI-3       ------MDE-----------------------------------------SAMY------
MpSCPYI-1       RMSRMACERCVQRYICLICLLLVIGGASFYLYWRYELRDDGAAPAPAPTAATLG------
SmSCPYI-15      ------MRKLVVVAFL----------------------------------AIFF------
SmSCPYI-14      KLEKLNPERL--------------------------LKQDINMKGIDLCRTPYR------
SmSCPYI-16      ------MRKL--------------------------VL------------AAFL------
SmSCPYI-17      ------MRKLVAVAFL----------------------------------AIFF------
SmSCPYI-18      ------MAASLV--------------------------------------SCFL------
SmSCPYI-1       ------MAIS--------------------------VV------------FCFV------
SmSCPYI-6       ------MKRY--------------------------VE------------SCQV------
SmSCPYI-2       ------MATS--------------------------VV------------SSLV------
SmSCPYI-10      ------M-------------------------------------------SCLI------
SmSCPYI-8       ------MKRY--------------------------VE------------SCQV------
SmSCPYI-9       ------MDSK--------------------------AL------------QLLI------
SmSCPYI-5       ------MAAS--------------------------VV------------SSLI------
SmSCPYI-7       ------MATS--------------------------VV------------FCFV------
PpSCPYI-2       FAAGYPNGGNYELTKDSRATANATVGNVTIETALVAERIAEVEGEDQPGGPPVL------
MpSCPYI-2       VGWAFIKNRQ--------------------------ARADREWWRHTYGGEPWRGTPYRR
CsSCPYI-1       LATLISQEQT----------------------------------------EDLT------
SmSCPYI-12      ------------------------------------------------------------
SmSCPYI-13      ------------------------------------------------------------
SmSCPYI-4       --MEQPESRHREDVNAL---------------------------------DPLI------
AtSCPYI-5       ------MSREI---------------------------------------EPLI------
VvSCPYI-1       ------MSRNM---------------------------------------EPLS------
FvSCPYI-1       ------M-------------------------------------------ETLT------
MtSCPYI-8       ------MSRP--------------------------L-------------EPLS------
AtSCPYI-6       --MENMGTRVI---------------------------------------EPLI------
MtSCPYI-1       ------MSIVT---------------------------------------DPLA------
AtSCPYI-1       ------MARISS--------------------------------------DPLM------
FvSCPYI-3       ------MAKIS---------------------------------------DPLV------
VvSCPYI-6       ------MARM--------------------------S-------------DPLI------
MtSCPYI-5       ------MARM--------------------------SQ------------EPLI------
VvSCPYI-5       ------MARI--------------------------L-------------EPLI------
OsSCPYI-13      ------MSRS--------------------------V-------------EPLV------
OsSCPYI-15      ------MSRS--------------------------V-------------EPLV------
SbSCPYI-9       ------MSRS--------------------------V-------------ESLI------
SbSCPYI-13      ------MSRS--------------------------V-------------EPLI------
SbSCPYI-11      ------MSRV--------------------------L-------------EPLV------
OsSCPYI-5       ------MSRV--------------------------L-------------EPLV------
SbSCPYI-7       ------MSRV--------------------------L-------------EPLI------
OsSCPYI-4       ------MSRVL---------------------------------------EPLI------
OsSCPYI-12      ------M-------------------------------------------DPLY------
SbSCPYI-5       ------MTDV----------------------------------------EPLV------
OsSCPYI-10      ------MAN-----------------------------------------DSLT------
SbSCPYI-17      ------MAN-----------------------------------------DSLT------
OsSCPYI-3       ------MAN-----------------------------------------DSLA------
SbSCPYI-6       ------MAN-----------------------------------------DSLV------
SbSCPYI-10      ------MAAN----------------------------------------DSLV------
OsSCPYI-11      ------MSRG--------------------------R-------------DPLA------
SbSCPYI-2       ------MSRG--------------------------R-------------DPLA------
MtSCPYI-2       ------MPQN--------------------------LV------------DPL-------
OsSCPYI-17      ------MSR-----------------------------------------DPLV------
SbSCPYI-8       ------MSR-----------------------------------------DPLV------
OsSCPYI-6       ------MSR-----------------------------------------DPLV------
SbSCPYI-12      ------MSR-----------------------------------------DPLV------
OsSCPYI-16      ------MSR-----------------------------------------DPLV------
SbSCPYI-1       ------MSR-----------------------------------------DPLV------
OsSCPYI-14      ----MSMSR-----------------------------------------DPLV------
SbSCPYI-14      -----MSSR-----------------------------------------DPLV------
MtSCPYI-4       ---MAGSSR-----------------------------------------NPLA------
MtSCPYI-3       ------MRIK--------------------------ST------------NPLV------
MtSCPYI-6       ------M-------------------------------------------NPLV------
FvSCPYI-5       ----MAKARD--------------------------Q-------------DALV------
AtSCPYI-2       ----MSLSRR----------------------------------------DPLV------
AtSCPYI-4       ------MSIN--------------------------IR------------DPLI------
SbSCPYI-3       TTTRQRGSRE--------------------------RRRSSDRAATMQRGDPLV------
FvSCPYI-2       ------MPRD--------------------------R-------------DPLV------
VvSCPYI-2       ------MPRE--------------------------R-------------DPLV------
OsSCPYI-2       GETMSGRGRG----------------------------------------DPLV------
SbSCPYI-15      ---MAGSRE---------------------------R-------------ETLV------
OsSCPYI-7       ---MAGSGRD----------------------------------------DPLV------
```

```
OsSCPYI-8       ---MAGSGRD--------------------------R-------------DPLV------
VvSCPYI-3       ------MAGS--------------------------L-------------ESLV------
PpSCPYI-1       ------MSRS--------------------------V-------------DPLV------
PpSCPYI-4       ------MARS--------------------------I-------------DPLV------
PpSCPYI-5       ------MPRS--------------------------I-------------DPLI------
PpSCPYI-6       ------MARS--------------------------I-------------DPLV------
SmSCPYI-3       ------MGRS--------------------------M-------------DPLV------
MtSCPYI-7       ------MAAS--------------------------V-------------DPLV------
AtSCPYI-3       ------MAAS--------------------------V-------------DPLV------
FvSCPYI-4       FYLIDGGPRVESFACLLSCFLLFLFFFFFFFFFFFFSLFSFFPITMAVSVDPLV------
VvSCPYI-4       ------MAAS--------------------------V-------------DPLV------
OsSCPYI-1       ------MARF--------------------------V-------------DPLV------
SbSCPYI-4       ------MARF--------------------------V-------------DPLV------
OsSCPYI-9       ------MASH--------------------------V-------------DPLV------
SbSCPYI-16      ------MAAH--------------------------V-------------DPLV------


SmSCPYI-11      --------------------------SPGG------------------------------
PpSCPYI-3       ----------------------------------------------------ITSRA---
MpSCPYI-1       ---------------------------AGAAAW-------------------LGGEVIPI
SmSCPYI-15      ---------------------------AGA----------------------TSSRLLAS
SmSCPYI-14      ---------------------------AGRLP--------------------NLGNV---
SmSCPYI-16      -----------------------AIFFAGA----------------------TSSRLLAS
SmSCPYI-17      ---------------------------AGA----------------------TSSRLLAS
SmSCPYI-18      ---------------------------VCL------------------------------
SmSCPYI-1       ---------------------------LCF----------------------FLVQV---
SmSCPYI-6       ----------------------------------------------------VLPED---
SmSCPYI-2       ---------------------------FFS----------------------LLAQV---
SmSCPYI-10      ---------------------------LCS----------------------LLAQA---
SmSCPYI-8       ----------------------------------------------------VLPED---
SmSCPYI-9       ----------------------LSLVSIGHFPNCGTSQCDRAHLLKECVPCVASGTV---
SmSCPYI-5       ---------------------------LCS----------------------FLVQV---
SmSCPYI-7       ---------------------------LCF----------------------FLIQV---
PpSCPYI-2       -----GNGFLSGDIVTTVPTPDLVKGVVGHLEQTG-----------------IIPEV---
MpSCPYI-2       PEAEEKRAPREATTATTTTTTTTREDDEDD----------------------VMDDAEIY
CsSCPYI-1       ---------------------------AAK----------------------VIPDVISG
SmSCPYI-12      ----------------------------------------------------IIPAW---
SmSCPYI-13      ----------------------------------------------------VIPDW---
SmSCPYI-4       ---------------------------LGG----------------------IIPDV---
AtSCPYI-5       ---------------------------VGR----------------------VIGDV---
VvSCPYI-1       ---------------------------VGR----------------------VVGDV---
FvSCPYI-1       ---------------------------LGR----------------------VVGEV---
MtSCPYI-8       ---------------------------VGR----------------------VIGEV---
AtSCPYI-6       ---------------------------MGR----------------------VVGDV---
MtSCPYI-1       ---------------------------IGR----------------------VIGDV---
AtSCPYI-1       ---------------------------VGR----------------------VIGDV---
FvSCPYI-3       ---------------------------VGR----------------------VIGDV---
VvSCPYI-6       ---------------------------VGR----------------------VIGDV---
MtSCPYI-5       ---------------------------VGR----------------------VIGEV---
VvSCPYI-5       ---------------------------VGR----------------------VIGDV---
OsSCPYI-13      ---------------------------VGR----------------------VIGEV---
OsSCPYI-15      ---------------------------VGR----------------------VIGEV---
SbSCPYI-9       ---------------------------VGR----------------------VIGEV---
SbSCPYI-13      ---------------------------VGR----------------------VIGEV---
SbSCPYI-11      ---------------------------VGK----------------------VIGEV---
OsSCPYI-5       ---------------------------VGK----------------------VIGEV---
SbSCPYI-7       ---------------------------VGK----------------------VIGEV---
OsSCPYI-4       ---------------------------VGK----------------------VIGEV---
OsSCPYI-12      ---------------------------LSQ----------------------IIPDV---
SbSCPYI-5       ---------------------------LAH----------------------VIRDV---
OsSCPYI-10      ---------------------------RSH----------------------IVGDV---
SbSCPYI-17      ---------------------------RGH----------------------IIGDV---
OsSCPYI-3       ---------------------------TGR----------------------VIGDV---
SbSCPYI-6       ---------------------------TAR----------------------VIGDV---
SbSCPYI-10      ---------------------------TAH----------------------VIGDV---
OsSCPYI-11      ---------------------------LSQ----------------------VIGDV---
SbSCPYI-2       ---------------------------LSQ----------------------VIGDV---
MtSCPYI-2       -----------------------------G----------------------VIGDV---
OsSCPYI-17      ---------------------------VGN----------------------VVGDI---
SbSCPYI-8       ---------------------------VGN----------------------VVGDI---
OsSCPYI-6       ---------------------------VGH----------------------VVGDI---
SbSCPYI-12      ---------------------------VGH----------------------VVGDI---
OsSCPYI-16      ---------------------------VGH----------------------VVGDI---
SbSCPYI-1       ---------------------------VGH----------------------VVGDI---
OsSCPYI-14      ---------------------------VGS----------------------IVGDV---
SbSCPYI-14      ---------------------------VGS----------------------IVGDI---
MtSCPYI-4       ---------------------------VGR----------------------VIGDV---
MtSCPYI-3       ---------------------------VGG----------------------VIGEV---
MtSCPYI-6       ---------------------------VCG----------------------VIGDV---
FvSCPYI-5       ---------------------------VSR----------------------VIGDI---
AtSCPYI-2       ---------------------------VGS----------------------VVGDV---
AtSCPYI-4       ---------------------------VSR----------------------VVGDV---
SbSCPYI-3       ---------------------------VGR----------------------IIGDV---
```

```
FvSCPYI-2      ---------------------------VGR----------------------VIGDV---
VvSCPYI-2      ---------------------------VGR----------------------VVGDV---
OsSCPYI-2      ---------------------------LGR----------------------VVGDV---
SbSCPYI-15     ---------------------------VGR----------------------VVGDV---
OsSCPYI-7      ---------------------------VGR----------------------IVGDV---
OsSCPYI-8      ---------------------------VGR----------------------VVGDV---
VvSCPYI-3      ---------------------------VGK----------------------VIGDV---
PpSCPYI-1      ---------------------------VGR----------------------VIGDV---
PpSCPYI-4      ---------------------------VGK----------------------VIGDV---
PpSCPYI-5      ---------------------------VGK----------------------VIGDV---
PpSCPYI-6      ---------------------------VGK----------------------VIGDV---
SmSCPYI-3      ---------------------------LGR----------------------VIGDV---
MtSCPYI-7      ---------------------------VGR----------------------VIGDV---
AtSCPYI-3      ---------------------------VGR----------------------VIGDV---
FvSCPYI-4      ---------------------------VGR----------------------VIGDV---
VvSCPYI-4      ---------------------------VGR----------------------VIGDV---
OsSCPYI-1      ---------------------------VGR----------------------VIGEV---
SbSCPYI-4      ---------------------------VGR----------------------VIGEV---
OsSCPYI-9      ---------------------------VGR----------------------VIGDV---
SbSCPYI-16     ---------------------------VGR----------------------VIGDV---


SmSCPYI-11     -------------------------------------------FH-HE----------GK
PpSCPYI-3      -------------------------------------------FK-NG----------ER
MpSCPYI-1      VDPTKWGEVPSPPAPAAATTTTTTEAATTPAPRPASSAATVAKFL-EVNAGVQNAAALPT
SmSCPYI-15     TPP----------------------------------------RS-PLPGSLAVEKS-IS
SmSCPYI-14     KHLRRSNQTKSCLQGIKAITTQQRINEEACGATSSRLLASTPPRS-PSPRSLAVEKS-IS
SmSCPYI-16     TPP----------------------------------------RS-PSPGSLAVEKS-IS
SmSCPYI-17     TPP----------------------------------------RS-PSPGSLAVEKS-IS
SmSCPYI-18     LSA----------------------------------------YA--A----------MG
SmSCPYI-1      -----------------------------------------------S----------VG
SmSCPYI-6      YQC----------------------------------------EG-II----------VG
SmSCPYI-2      -----------------------------------------------S----------VG
SmSCPYI-10     -----------------------------------------------T----------VG
SmSCPYI-8      YQC----------------------------------------EG-II----------VG
SmSCPYI-9      LEHPDVNCCKAVNALGHNCLCSLIVSSTGVLAKTTMKRYVESCQV-VPPEDYQCEGIIVG
SmSCPYI-5      -----------------------------------------------S----------VG
SmSCPYI-7      ----------------------------------------------S-----------VG
PpSCPYI-2      IDR----------------------------------------FT-PT----------TT
MpSCPYI-2      LET----------------------------------------LG-IPRGL-------PT
CsSCPYI-1      IDP-----------------------------------------A-PG----------VA
SmSCPYI-12     VDS----------------------------------------YDSPY----------VS
SmSCPYI-13     VDS----------------------------------------FHFPR----------PS
SmSCPYI-4      VDD----------------------------------------FV-PC----------CE
AtSCPYI-5      LEM----------------------------------------FN-PS----------VT
VvSCPYI-1      VDG----------------------------------------FT-PS----------VK
FvSCPYI-1      VDM----------------------------------------FT-PS----------VK
MtSCPYI-8      VDI----------------------------------------FN-PS----------VR
AtSCPYI-6      LDF----------------------------------------FT-PT----------TK
MtSCPYI-1      VDY----------------------------------------FT-ST----------MK
AtSCPYI-1      VDN----------------------------------------CL-QA----------VK
FvSCPYI-3      IDC----------------------------------------FT-PS----------VK
VvSCPYI-6      VDS----------------------------------------FC-ST----------VK
MtSCPYI-5      LDS----------------------------------------FT-TS----------MK
VvSCPYI-5      LDP----------------------------------------FP-PT----------IK
OsSCPYI-13     IDS----------------------------------------FN-PC----------TK
OsSCPYI-15     LDT----------------------------------------FN-PC----------MK
SbSCPYI-9      LDS----------------------------------------FS-PC----------VK
SbSCPYI-13     LDS----------------------------------------FN-PC----------VK
SbSCPYI-11     IDN----------------------------------------FN-PT----------VK
OsSCPYI-5      IDN----------------------------------------FN-PT----------VK
SbSCPYI-7      LDH----------------------------------------FN-PT----------VK
OsSCPYI-4      LDN----------------------------------------FN-PT----------VK
OsSCPYI-12     LDP----------------------------------------FI-ST----------IS
SbSCPYI-5      LDS----------------------------------------FT-PT----------IP
OsSCPYI-10     LDQ----------------------------------------FS-NS----------VP
SbSCPYI-17     LDP----------------------------------------FT-SS----------VP
OsSCPYI-3      LDP----------------------------------------FI-ST----------VD
SbSCPYI-6      LDP----------------------------------------FY-SS----------ID
SbSCPYI-10     LDP----------------------------------------FY-TT----------VD
OsSCPYI-11     LDP----------------------------------------FI-KS----------AA
SbSCPYI-2      LDP----------------------------------------FI-KS----------AT
MtSCPYI-2      LSP----------------------------------------FT-NS----------VS
OsSCPYI-17     LDP----------------------------------------FI-KS----------AS
SbSCPYI-8      LDP----------------------------------------FI-KS----------AS
OsSCPYI-6      LDP----------------------------------------FN-KS----------AS
SbSCPYI-12     LDP----------------------------------------FI-KT----------AS
OsSCPYI-16     VDP----------------------------------------FV-TT----------AS
SbSCPYI-1      VDP----------------------------------------FI-TT----------AS
OsSCPYI-14     VDH----------------------------------------FG-AS----------AL
SbSCPYI-14     VDY----------------------------------------FS-AS----------AL
MtSCPYI-4      IDS----------------------------------------FE-NS----------IP
MtSCPYI-3      LDP----------------------------------------FT-SS----------VS
```

```
MtSCPYI-6       LDP----------------------------------------FT-NS----------VS
FvSCPYI-5       IEP----------------------------------------FT-KS----------VS
AtSCPYI-2       LDP----------------------------------------FT-RL----------VS
AtSCPYI-4       LDP----------------------------------------FN-RS----------IT
SbSCPYI-3       VDP----------------------------------------FV-RR----------VP
FvSCPYI-2       LDP----------------------------------------FT-KS----------VS
VvSCPYI-2       LDP----------------------------------------FL-RS----------IT
OsSCPYI-2       VDP----------------------------------------FV-RR----------VA
SbSCPYI-15      LDP----------------------------------------FV-RT----------TN
OsSCPYI-7       LDP----------------------------------------FV-RI----------TN
OsSCPYI-8       LDA----------------------------------------FV-RS----------TN
VvSCPYI-3       VNM----------------------------------------FT-PA----------AE
PpSCPYI-1       IDM----------------------------------------FA-PS----------VD
PpSCPYI-4       IDT----------------------------------------FV-PS----------VD
PpSCPYI-5       IDT----------------------------------------FV-PR----------VD
PpSCPYI-6       IDT----------------------------------------FV-PS----------VD
SmSCPYI-3       LDM----------------------------------------FV-PA----------VD
MtSCPYI-7       VDM----------------------------------------FI-PS----------VG
AtSCPYI-3       LDM----------------------------------------FI-PT----------AN
FvSCPYI-4       VDM----------------------------------------FV-PT----------VN
VvSCPYI-4       VDM----------------------------------------FV-PT----------IN
OsSCPYI-1       VDL----------------------------------------FV-PS----------IS
SbSCPYI-4       VDL----------------------------------------FV-PS----------IS
OsSCPYI-9       VDL----------------------------------------FV-PT----------TA
SbSCPYI-16      VDL----------------------------------------FV-PT----------VA


SmSCPYI-11      IPRKH-----------T--GDGQ-GDQKDISPALEWYNVPEGTVS---------------
PpSCPYI-3       IPPQY-----------T--QDGQ-GARSNISPPLEWYNIPDETVC---------------
MpSCPYI-1       LTVTY-----------A--NHDV-VPGEHFQSSRDAFVLNPPPA--------VSW--SAI
SmSCPYI-15      IAVSY-----------T--GKDTFADGTLLTC---PQTAIHPNVM-------VPG--DVD
SmSCPYI-14      IAVSY-----------T--GKDTFADGTLLTC---PQTAIHPNVM-------VPG--DVD
SmSCPYI-16      IAVSY-----------T--GKDTFADGTLLTC---PQTAIHPNVM-------VPG--DVD
SmSCPYI-17      IAVSY-----------T--GKDTFADGTLLTC---PQTAIHPNVM-------VPG--DVD
SmSCPYI-18      LLVQY-----------G--STTV-GNGNFIPL---SSTNSPPTVD-------IEGFRGNG
SmSCPYI-1       LLVQY-----------G--STTV-GNGNFVSL---SSTDSAPTVD-------IEGFGKNG
SmSCPYI-6       LLVQY-----------G--STTV-GNGNLVSL---SSTDSAPTVD-------IEGFGKNG
SmSCPYI-2       LLVQY-----------G--STTV-GNGNFIPL---SSTDAAPTVD-------IQGFGKNG
SmSCPYI-10      LLVQY-----------G--SVTV-GNGNFIPL---SSTDSAPTVD-------IEGFGKNG
SmSCPYI-8       LLVQY-----------G--STTV-GNGNFVSL---SSTDSAPTVD-------IEGFGKNG
SmSCPYI-9       LLVQY-----------G--STTV-GNGNFVSL---SSTGSAPTVD-------IEGFGKNG
SmSCPYI-5       LLVQY-----------G--STTV-GNGNFISL---SSTGSAPTVD-------IEGYGKNG
SmSCPYI-7       LLVQY-----------G--STTV-GNGNFVSL---SSTDSAPTVD-------IEGFGKNG
PpSCPYI-2       VRLIY----------ND--NLEV-LDGTKVSE---NDVSKQPKVE-------IEG-AFVM
MpSCPYI-2       LRVVYEKRTLGEREGNT--PRPI-APGERFSSRHDAFLQAAPVVSWRDLDAAVDG--EER
CsSCPYI-1       LKIQY-----------G--DTPITTKGGRLPR---PETLNAPSVQ-------VTDLVGNV
SmSCPYI-12      VSATF-----------G--SRAVTTTGQMFKQ---ADTQKPPVVS-------ISD-IHAK
SmSCPYI-13      LRVAY-----------G--SQNV-TIERQFSP---AEVLLQPKVS-------ITN--AGN
SmSCPYI-4       MAVYY-----------G--KDQV-TNGCELAP---FATSSPPNVQ-------IAGNFDDG
AtSCPYI-5       MRVTF-----------N--SNTIVSNGHELAP---SLLLSKPRVE-------IGG--QDL
VvSCPYI-1       MSVTY----------NS--NKQV-ANGHELMP---SVVTAKPRVE-------VGG--EDL
FvSCPYI-1       MNVVY-----------SSCNKQV-SNGHEIMP---SVIAAKPRVD-------IGG--EDM
MtSCPYI-8       MNVTY-----------S--TKQV-ANGHELMP---SIVMNKPRVD-------IGG--EDM
AtSCPYI-6       MNVSY-----------N--KKQV-SNGHELFP---SSVSSKPRVE-------IHG--GDL
MtSCPYI-1       MSVTY-----------N--TKQV-YNGHEFFP---SSVTTKPKVQ-------IHG--GDM
AtSCPYI-1       MTVTY----------NS--DKQV-YNGHELFP---SVVTYKPKVE-------VHG--GDM
FvSCPYI-3       MTVTY----------NS--SKKV-YNGHELYP---SSVTVKPKVE-------VHG--GDM
VvSCPYI-6       MTVTY----------NS--NKQV-YNGHELFP---SSVTIKPKIE-------VEG--GDM
MtSCPYI-5       MTVSY-----------N--KKQV-FNGHEFFP---STINTKPKVE-------IDG--GDM
VvSCPYI-5       MTVTY-----------H--NKQI-CNGYELYP---SSITVKPRVE-------VQG--GDL
OsSCPYI-13      MIVTY-----------N--SNKLVFNGHEFYP---SAVVSKPRVE-------VQG--GDM
OsSCPYI-15      MIVTY-----------N--SNKLVFNGHELYP---SAVVSKPRVE-------VQG--GDL
SbSCPYI-9       MVVTY-----------N--SNKLVFNGHEIYP---SAVVSKPRVE-------VQG--GDL
SbSCPYI-13      MIVTY-----------N--SNKLVFNGHEIYP---SAVVSKPRVE-------VQG--GDL
SbSCPYI-11      MTVTY-----------GS-NNQV-FNGHEFFP---SAVLSKPRVE-------VQG--DDM
OsSCPYI-5       MTATY----------SS--NKQV-FNGHELFP---SAVVSKPRVE-------VQG--GDL
SbSCPYI-7       MVVTY----------NS--NKQV-FNGHEFFP---SAVTAKPRVE-------VQG--GDL
OsSCPYI-4       MTATY-----------GA-NKQV-FNGHEFFP---SAVAGKPRVE-------VQG--GDL
OsSCPYI-12      LRVTY-----------N--SRLL-LAGAALKP---SAVVSKPQVD-------VGG--NDM
SbSCPYI-5       LRIAY-----------N--NRLL-LAGVELKP---SAVVNNPRVD-------VGG--TDL
OsSCPYI-10      LTVMY-----------D--GRPV-FNGKEFRS---SAVSMKPRVE-------IGG--DDF
SbSCPYI-17      LTVMY-----------D--GRPV-FDGMEFRA---SAVSVKPRVE-------IGG--DDF
OsSCPYI-3       LTVMY-----------GDDGMPV-ISGVELRA---PAVAEKPVVE-------VGG--DDL
SbSCPYI-6       LMVLF-----------N--GMPI-VSGMELRA---PTVSERPRVE-------IGG--DDY
SbSCPYI-10      MMILF-----------D--GTPI-ISGMELRA---PAVSDRPRVE-------IGG--DDY
OsSCPYI-11      MRINY-----------G--EKEI-TNGTGVRS---SAVFTAPHVE-------IEG--RDQ
SbSCPYI-2       MRINY-----------G--DKEI-TNGTGLRA---SAVFNAPHVE-------IEG--HDQ
MtSCPYI-2       LSALI-----------N--NREI-SNGCIMKP---SQLVNRPRVN-------VGG--DDL
OsSCPYI-17      LRVLY-----------S--NREL-TNGSELKP---SQVANEPRIE-------IAG--RDM
SbSCPYI-8       LRVLY-----------N--NREL-TNGSELKP---SQVANEPRIE-------IAG--HDM
OsSCPYI-6       LKVLY-----------N--NKEL-TNGSELKP---SQVANEPRIE-------IAG--RDI
SbSCPYI-12      LKVLY-----------N--NKEL-TNGSELKP---SQVANEPRVE-------IGG--RDM
OsSCPYI-16      LRVFY-----------N--SKEM-TNGSELKP---SQVLNQPRIY-------IEG--RDM
```

```
SbSCPYI-1       LRVFY-----------N--NKEM-TNGSDLKP---SQVMNEPRVH-------ISG--RDM
OsSCPYI-14      LRLFY-----------N--HREM-TSGSELRP---SQVAGEPAVQ-------ITG-GRDG
SbSCPYI-14      LRVMY-----------G--GREI-TCGSELRP---SQVAGEPTVH-------ITGGRRDG
MtSCPYI-4       LRVTY-----------G--NRDV-NNGCELKP---SQIGNQPRVS-------VGG--NDL
MtSCPYI-3       LRVVY----------DN--NKEV-INSGELKP---SQIINSPRVQ-------VGG--NDL
MtSCPYI-6       LRVVY----------EN--NKEV-SNSGELKP---SQIVNPPRVQ-------VGG--NDL
FvSCPYI-5       LRMTY----------IN--NREF-TNGSELKP---SHVVHRPRVD-------IGG--DDL
AtSCPYI-2       LKVTY-----------G--HREV-TNGLDLRP---SQVLNKPIVE-------IGG--DDF
AtSCPYI-4       LKVTY-----------G--QREV-TNGLDLRP---SQVQNKPRVE-------IGG--EDL
SbSCPYI-3       LRVAY-----------A--AREI-SNGCELRP---SAIADQPRVE-------VGG--PDM
FvSCPYI-2       LRVTY-----------T--SKEV-NNGCELKP---SQVVSQPRVD-------IGG--EDL
VvSCPYI-2       LRVTY-----------N--NREV-ANGCEFRP---SQLVSQPRVD-------IGG--DDL
OsSCPYI-2       LRVAY-----------G--AREV-ANGCELRP---SAVADQPRVA-------VGG--PDM
SbSCPYI-15      LRVSY-----------G--TRTV-SNGCELKP---SMVVNQPRVE-------VGG--PDM
OsSCPYI-7       LSVSY-----------G--ARIV-SNGCELKP---SMVTQQPRVV-------VGG--NDM
OsSCPYI-8       LKVTY-----------G--SKTV-SNGCELKP---SMVTHQPRVE-------VGG--NDM
VvSCPYI-3       FTVHF-----------G--SRQV-ANGRMIPP---SAAVDKPKVQ-------IHG--HRL
PpSCPYI-1       MAVVY-----------T--SRKV-SNGCQMKP---SATNEAPTVH-------VTG--NNG
PpSCPYI-4       MAIHY-----------S--TRQV-TNGCQMMP---SATAQAPEIH-------LSD--KSG
PpSCPYI-5       MAIHY-----------S--TRQV-TNGCQLKP---SATAQAPEIQ-------LSD--KSG
PpSCPYI-6       MAIHY-----------S--SRQV-TNGCQMKP---SATAQAPEIQ-------LSD--NSE
SmSCPYI-3       MSVCY-----------G--SKQV-NNGCELKP---SATQARPIVQ-------VGS--PHE
MtSCPYI-7       MSVYF-----------G--PKHV-TNGCDIKP---SMAINPPKVT-------LTG---NM
AtSCPYI-3       MSVYF-----------G--PKHI-TNGCEIKP---STAVNPPKVN-------ISG--HSD
FvSCPYI-4       MSVYF-----------G--SKHV-TNGCDIKP---SIAVSPPKVT-------FSG--HPG
VvSCPYI-4       MSVYY-----------G--AKHV-TNGCDVKP---SLTVNPPKVT-------LSG--HPD
OsSCPYI-1       MTAAY-----------G--DRDI-SNGCLVRP---SAADYPPLVR-------ISG--RRN
SbSCPYI-4       MTVAY-----------G--PKDI-SNGCLLKP---SATAAPPLVR-------ISG--RRN
OsSCPYI-9       MSVRF-----------G--TKDL-TNGCEIKP---SVAAAPPAVQ-------IAG--RVN
SbSCPYI-16      MSVRF-----------G--TKDV-TNGCEIKP---SLTAAAPVVQ-------IAG--RAN
                .                            .

SmSCPYI-11      --------------LALVVDDPDAPDPKDPVVP--------WVHWVLINIPPTLKGIPAG
PpSCPYI-3       --------------LVLIVEDPA-PQ-MERSPRS-------FCHWVVLNIPPTLKGLPEH
MpSCPYI-1       RPPLDPTKH-----RAVIMFDPDAPEPA-PGDGATPGANAPYLHAVWTTHGGNA------
SmSCPYI-15      PAQL----------YSIVMIDRQASQSMFTLADQFT-----YVHYWIANIPGS-------
SmSCPYI-14      PAQL----------YSIVMIDRRASQSMFTLADQFT-----YVHYWIANIPGS-------
SmSCPYI-16      PAQL----------YSIVMIDRRASRSMFTLADQFT-----YVHYWIANIPGS-------
SmSCPYI-17      PAQL----------YSIVMIDRRASRSMFTLADQFT-----YVHYWITNIPGS-------
SmSCPYI-18      SVRL----------FSVVMADQKALPSPNAVYP--------YINYWIANIPAGS------
SmSCPYI-1       TTRL----------FSVVMADQKALVASNKVYP--------YINFWVANIPAGS------
SmSCPYI-6       TTRL----------FSVVMADQKALVASNKVYP--------YINFWIANIPAGS------
SmSCPYI-2       TARL----------FSVVMADQKALVAPNKVYP--------YINFWIANIPAGS------
SmSCPYI-10      TARL----------FSVVMADQKALSAPTKVYP--------YINFWIANIPAGS------
SmSCPYI-8       TTRL----------FSVVMADQKALVASNKVYP--------YINFWIANIPAGS------
SmSCPYI-9       TTRL----------FSVVMADQKALVASNKVYP--------YINFWIANIPAGS------
SmSCPYI-5       TVSL----------FSVVMADQKALSAQNKVYP--------YINFWIANIPAGS------
SmSCPYI-7       TTRL----------FSVVMADQKALVASNKVYP--------YINFWIANIPAGS------
PpSCPYI-2       PGSL----------YTIMLVSSSAIGEG----KE-------RVHWIHVNYQGPQ------
MpSCPYI-2       RPRPRPRP------HVVVMVDPDAPAPRTAGRRHLPGAAGPWLHALWKDCDGD-------
CsSCPYI-1       LSKLDPLKLQADTKYTLVLSDPDAPSPAMPTSRE-------FLHWIVTNAPFG-------
SmSCPYI-12      KGDL----------FTLLMVDPDAVSPEKPIYRN-------VLHWIVTNIPTG-------
SmSCPYI-13      R-DL----------FTLVMVDPDPPGPQIPILRN-------ILHWIVVNIPAQ-------
SmSCPYI-4       --SL----------FTLVMTDPDAPSPAEPSLGE-------YLHWLVTDIPGG-------
AtSCPYI-5       R-SF----------FTLIMMDPDAPSPSNPYMRE-------YLHWMVTDIPGT-------
VvSCPYI-1       R-AA----------YTLIMTDPDAPSPSDPYLKE-------HLHWIVADIPGT-------
FvSCPYI-1       R-AA----------YTLIMTDPDYPSPSDPCLRE-------HLHWMVTDIPGT-------
MtSCPYI-8       R-SA----------YTLIMTDPDAPSPSDPHLRE-------HLHWMVTDIPGT-------
AtSCPYI-6       R-SF----------FTLVMIDPDVPGPSDPFLKE-------HLHWIVTNIPGT-------
MtSCPYI-1       R-SF----------FTLVMTDPDVPGPSDPYLKE-------HLHWIVTDIPGT-------
AtSCPYI-1       R-SF----------FTLVMTDPDVPGPSDPYLRE-------HLHWIVTDIPGT-------
FvSCPYI-3       R-SF----------FTLVMTDPDVPGPSDPYLKE-------HLHWIVTDIPGT-------
VvSCPYI-6       R-SF----------FTLIMTDPDVPGPSDPYLRE-------HLHWIVTDIPGT-------
MtSCPYI-5       R-SF----------YTLVMTDPDVPGPSDPYLRE-------HLHWIVTDIPGT-------
VvSCPYI-5       R-SF----------FTLVMTDPDVPGPSDPYLRE-------HLHWIVTDIPGT-------
OsSCPYI-13      R-SF----------FTLVMTDPDVPGPSDPYLRE-------HLHWIVTDIPGT-------
OsSCPYI-15      R-SF----------FTLVMTDPDVPGPSDPYLRE-------HLHWIVTDIPGT-------
SbSCPYI-9       R-SF----------FTLVMTDPDVPGPSDPYLRE-------HLHWIVTDIPGT-------
SbSCPYI-13      R-SF----------FTLVMTDPDVPGPSDPYLRE-------HLHWIVTDIPGT-------
SbSCPYI-11      R-SF----------FTLVMTDPDVPGPSDPYLRE-------HLHWIVTDIPGT-------
OsSCPYI-5       R-SF----------FTLVMTDPDVPGPSDPYLRE-------HLHWIVTDIPGT-------
SbSCPYI-7       R-SF----------FTLVMTDPDVPGPSDPYLRE-------HLHWIVTDIPGT-------
OsSCPYI-4       R-SF----------FTLVMTDPDVPGPSDPYLRE-------HLHWIVTDIPGT-------
OsSCPYI-12      R-VS----------YTLVLVDPDAPSPSDPSLRE-------YLHWMVTDIPET-------
SbSCPYI-5       R-VF----------YTLVLVDPDAPSPSNPSLRE-------YLHWMVIDIPGT-------
OsSCPYI-10      R-FA----------YTLVMVDPDAPNPSNPTLRE-------YLHWMVTDIPSS-------
SbSCPYI-17      R-VA----------YTLVMVDPDAPNPSNPTLRE-------YLHWMVTDIPAS-------
OsSCPYI-3       R-VA----------YTLVMVDPDAPNPSNPTLRE-------YLHWMVTDIPAS-------
SbSCPYI-6       R-VA----------YTLVMVDPDAPNPSNPTLRE-------YLHWMVTDIPAS-------
SbSCPYI-10      R-VA----------YTLVMVDPDAPNPSNPTLRE-------YLHWMVTDIPAS-------
OsSCPYI-11      T-KL----------YTLVMVDPDAPSPSKPEYRE-------YLHWLVTDIPEA-------
SbSCPYI-2       T-KL----------YTLVMVDPDAPSPSKPEYRE-------YLHWLVTDIPEA-------
MtSCPYI-2       R-TF----------YTMVMVDADAPSPSNPFLKE-------YLHWMVTDIPAT-------
```

```
OsSCPYI-17      R-TL----------YTLVMVDPDSPSPSNPTKRE-------YLHWLVTDIPET-------
SbSCPYI-8       R-TL----------YTLVMVDPDSPSPSNPTKRE-------YLHWLVTDIPES-------
OsSCPYI-6       R-NL----------YTLVMVDPDSPSPSNPTKRE-------YLHWLVTDIPES-------
SbSCPYI-12      R-NL----------YTLVMVDPDSPSPSNPTKRE-------YLHWLVTDIPES-------
OsSCPYI-16      R-TL----------YTLVMVDPDAPSPSNPTKRE-------YLHWMVTDIPET-------
SbSCPYI-1       R-TL----------YTLVMVDPDAPSPSNPTKRE-------NLHWLVTDIPET-------
OsSCPYI-14      R-AL----------YTLVMVDPDAPSPSNPSKRE-------YLHWLVTDVPEG-------
SbSCPYI-14      TPAF----------YTLLMLDPDAPSPSNPTKRE-------YLHWLVTDIPEG-------
MtSCPYI-4       R-NL----------YTLVMVDPDSPSPSNPTFKE-------YLHWLVTDIPGT-------
MtSCPYI-3       R-TL----------YTLVMVNPDAPSPSDPNMRE-------YLYWMVTNIPAT-------
MtSCPYI-6       R-TL----------YTLVMVDPDGPSPSNPNMRE-------YLHWMVTNIPAT-------
FvSCPYI-5       R-NF----------YTLIMVDPDAPNPSEPNLKE-------YLHWLVTDIPAT-------
AtSCPYI-2       R-NF----------YTLVMVDPDVPSPSNPHQRE-------YLHWLVTDIPAT-------
AtSCPYI-4       R-NF----------YTLVMVDPDVPSPSNPHLRE-------YLHWLVTDIPAT-------
SbSCPYI-3       R-TF----------YTLVMVDPDAPSPSDPNLRE-------YLHWLVTDIPAT-------
FvSCPYI-2       R-TF----------YTLVMVDPDAPSPSDPNLKE-------YLHWLVTDIPAT-------
VvSCPYI-2       R-TF----------YTLVMVDPDAPSPSNPNLRE-------YLHWLVTDIPAT-------
OsSCPYI-2       R-TF----------YTLVMVDPDAPSPSDPNLRE-------YLHWLVTDIPAT-------
SbSCPYI-15      R-TF----------YTLVMVDPDAPSPSDPNLRE-------YLHWLVTDIPGT-------
OsSCPYI-7       R-TF----------YTLVMVDPDAPSPSNPNLRE-------YLHWLVTDIPGT-------
OsSCPYI-8       R-TF----------YTLVMVDPDAPSPSDPNLRE-------YLHWLVTDIPGT-------
VvSCPYI-3       SSNL----------YTLVMVDPDAPSPSEPTFRE-------WLHWIVVDIPEG-------
PpSCPYI-1       DNNF----------FTLIMTDPDAPSPSEPSLRE-------WVHWIVTDIPGN-------
PpSCPYI-4       GNNL----------YTLIMIDPDAPSPSEPTLRE-------WLHWIVTDIPGN-------
PpSCPYI-5       DNNY----------YTLVMTDPDAPSPSEPSLRE-------WLHWIVTDIPGN-------
PpSCPYI-6       GNNY----------YTLIMTDPDAPSPSEPSLRE-------WLHWIVTDIPGN-------
SmSCPYI-3       EGAL----------YTLVMVDPDAPSPSEPSMRE-------WVHWIVADIPGG-------
MtSCPYI-7       D-NL----------YTLVMTDPDAPSPSEPSMRE-------LIHWIVVDIPGG-------
AtSCPYI-3       E--L----------YTLVMTDPDAPSPSEPNMRE-------WVHWIVVDIPGG-------
FvSCPYI-4       E--L----------YTLVMTDPDAPSPSEPSMRE-------WVHWILADIPGG-------
VvSCPYI-4       E--F----------YTLVMTDPDAPSPSEPSMRE-------WVHWIVADIPGG-------
OsSCPYI-1       D--L----------YTLIMTDPDAPSPSDPSMRE-------FLHWIVVNIPGG-------
SbSCPYI-4       D--L----------YTLIMTDPDAPSPSDPTMRE-------YLHWIVTNIPGG-------
OsSCPYI-9       E--L----------FALVMTDPDAPSPSEPTMRE-------WLHWLVVNIPGG-------
SbSCPYI-16      D--L----------FTLVMTDPDAPSPSEPTMRE-------LIHWLVVNIPGG-------
                                :::  .

SmSCPYI-11      FTTKGL-DEKSEFA----------------------------------------------
PpSCPYI-3       FTTKEHDDDRDELT----------------------------------------------
MpSCPYI-1       -------------A----------------------------------------------
SmSCPYI-15      ------------------------------------------------------------
SmSCPYI-14      ------------------------------------------------------------
SmSCPYI-16      ------------------------------------------------------------
SmSCPYI-17      ------------------------------------------------------------
SmSCPYI-18      -------------S----------------------------------------------
SmSCPYI-1       -------------T----------------------------------------------
SmSCPYI-6       -------------T----------------------------------------------
SmSCPYI-2       -------------T----------------------------------------------
SmSCPYI-10      -------------T----------------------------------------------
SmSCPYI-8       -------------T----------------------------------------------
SmSCPYI-9       -------------T----------------------------------------------
SmSCPYI-5       -------------T----------------------------------------------
SmSCPYI-7       -------------T----------------------------------------------
PpSCPYI-2       -----------DIG----------------------------------------------
MpSCPYI-2       -------------T---------------------SAAATAPGEARPRARRFPSRTFFLS
CsSCPYI-1       ------------------------------------------------------------
SmSCPYI-12      -------------T----------------------------------------------
SmSCPYI-13      -------------S----------------------------------------------
SmSCPYI-4       -------------T----------------------------------------------
AtSCPYI-5       -------------T----------------------------------------------
VvSCPYI-1       -------------T----------------------------------------------
FvSCPYI-1       -------------T----------------------------------------------
MtSCPYI-8       -------------T----------------------------------------------
AtSCPYI-6       -------------T----------------------------------------------
MtSCPYI-1       -------------T----------------------------------------------
AtSCPYI-1       -------------T----------------------------------------------
FvSCPYI-3       -------------T----------------------------------------------
VvSCPYI-6       -------------T----------------------------------------------
MtSCPYI-5       -------------T----------------------------------------------
VvSCPYI-5       -------------T----------------------------------------------
OsSCPYI-13      -------------T----------------------------------------------
OsSCPYI-15      -------------T----------------------------------------------
SbSCPYI-9       -------------T----------------------------------------------
SbSCPYI-13      -------------T----------------------------------------------
SbSCPYI-11      -------------T----------------------------------------------
OsSCPYI-5       -------------T----------------------------------------------
SbSCPYI-7       -------------T----------------------------------------------
OsSCPYI-4       -------------T----------------------------------------------
OsSCPYI-12      -------------T----------------------------------------------
SbSCPYI-5       -------------T----------------------------------------------
OsSCPYI-10      -------------T----------------------------------------------
SbSCPYI-17      -------------T----------------------------------------------
OsSCPYI-3       -------------T----------------------------------------------
```

```
SbSCPYI-6       -------------T----------------------------------------------
SbSCPYI-10      -------------T----------------------------------------------
OsSCPYI-11      -------------I----------------------------------------------
SbSCPYI-2       -------------R----------------------------------------------
MtSCPYI-2       -------------T----------------------------------------------
OsSCPYI-17      -------------T----------------------------------------------
SbSCPYI-8       -------------T----------------------------------------------
OsSCPYI-6       -------------A----------------------------------------------
SbSCPYI-12      -------------A----------------------------------------------
OsSCPYI-16      -------------T----------------------------------------------
SbSCPYI-1       -------------T----------------------------------------------
OsSCPYI-14      -------------G----------------------------------------------
SbSCPYI-14      -------------A----------------------------------------------
MtSCPYI-4       -------------T----------------------------------------------
MtSCPYI-3       -------------T----------------------------------------------
MtSCPYI-6       -------------T----------------------------------------------
FvSCPYI-5       -------------T----------------------------------------------
AtSCPYI-2       -------------T----------------------------------------------
AtSCPYI-4       -------------T----------------------------------------------
SbSCPYI-3       -------------T----------------------------------------------
FvSCPYI-2       -------------A----------------------------------------------
VvSCPYI-2       -------------T----------------------------------------------
OsSCPYI-2       -------------T----------------------------------------------
SbSCPYI-15      -------------T----------------------------------------------
OsSCPYI-7       -------------T----------------------------------------------
OsSCPYI-8       -------------T----------------------------------------------
VvSCPYI-3       -------------C----------------------------------------------
PpSCPYI-1       -------------SSTTTSVIRQSVAGQGSKRAREPASSAKQPNVERKKKGPAASTTDKE
PpSCPYI-4       -------------S--------------------------------------GGSEMTSG
PpSCPYI-5       -------------S--------------------------------------GGSETNTG
PpSCPYI-6       -------------S--------------------------------------GGSETTSG
SmSCPYI-3       -------------A----------------------------------------------
MtSCPYI-7       -------------T----------------------------------------------
AtSCPYI-3       -------------T----------------------------------------------
FvSCPYI-4       -------------T----------------------------------------------
VvSCPYI-4       -------------T----------------------------------------------
OsSCPYI-1       -------------T----------------------------------------------
SbSCPYI-4       -------------T----------------------------------------------
OsSCPYI-9       -------------T----------------------------------------------
SbSCPYI-16      -------------A----------------------------------------------


SmSCPYI-11      -----------EIQE--GYNDWKLPGYRGPNPP-----------VGTHRYVFKLYAL---
PpSCPYI-3       -----------QIRE--GINDFKVPSYFGPKPP-----------VGEHNYEFRLYAL---
MpSCPYI-1       -----------TAPLAVP-RL--AVPYEPPVPP-----------KGTHRYVFVLFEQ---
SmSCPYI-15      -----------KVDA--G-TI--LEEYMRPAPH----------DFMLHKYEFILYKQ---
SmSCPYI-14      -----------KVDA--G-TI--LEEYMRPAPH----------DFMQHKYEFILYKQ---
SmSCPYI-16      -----------KVDA--G-TI--LEEYMRPAPH----------DFMQHKYEFILYKQ---
SmSCPYI-17      -----------KVDA--G-TI--LEEYMRPAPH----------DFMQHKYEFILYKQ---
SmSCPYI-18      -----------GTVN--G-SI--LEPYVAPSNTSNNIHYPNPFSAKNHTYDFVLVPQ---
SmSCPYI-1       -----------GAVK--G-SI--LEPYVSPSNTSKNIPYPNPFNARNHTYDFVLVPQ---
SmSCPYI-6       -----------GAVK--G-SI--LEPYVSPSNTSKNIPYPNPFNARNHTYDFVLVPQ---
SmSCPYI-2       -----------GAVK--S-SI--LEPYVAPSNTSKNIPYQNPFSAKNHTYDFVLVPQ---
SmSCPYI-10      -----------GAVK--G-SI--LEPYVSPSNTSKNIPYPNPFNARNHTYDFVLVPQ---
SmSCPYI-8       -----------GAVK--G-SI--LEPYVSPSNTSKNIPYPNPFNARNHTYDFVLVPQ---
SmSCPYI-9       -----------GAVK--G-SI--LEPYVSPSNTSKNIPYPNPFNARNHTYDFVLVPQ---
SmSCPYI-5       -----------GAVK--G-SI--LEPYVSPSNTSKNIPYPNPFNAKNHTYDFVLVPQ---
SmSCPYI-7       -----------GAVK--G-SI--LEPYVSPSNTSKNIPYPNPFNARNHTYDFVLVLQ---
PpSCPYI-2       -----------SAVIRAG-AD--VLHCEAPKAG-----------SNSNQLVFLLFRQ---
MpSCPYI-2       PPARGEDAHEEDLSRVPG--L--LVPYNGPSPP-----------SGHHRYVFVLFEQTDD
CsSCPYI-1       -----------DITK--G-EV--AVPYAPPSPP-----------AGVHRYVFSLFQQ---
SmSCPYI-12      -----------KDVFKHG-TN--AVSYAGPSPP-----------MGVHRYYILVFKQ---
SmSCPYI-13      ---------TNASEQ--G-DH--LAPYLSPTPV-----------QGVHRYYFLLFRQ---
SmSCPYI-4       -----------DPSK--G-KG--VLPYERPKPP-----------AGTHRYTFCLFKQ---
AtSCPYI-5       -----------DASF--G-RE--IVRYETPKPV-----------AGIHRYVFALFKQ---
VvSCPYI-1       -----------DASF--G-KE--IVSYEPPKPV-----------IGIHRYAFILFKQ---
FvSCPYI-1       -----------DVSF--G-RE--VVEYETPKPV-----------VGIHRYVFLLFQQ---
MtSCPYI-8       -----------DVSF--G-NE--IVEYENPKPV-----------IGIHRYVFILFKQ---
AtSCPYI-6       -----------DATF--G-KE--VVSYELPRPS-----------IGIHRFVFVLFRQ---
MtSCPYI-1       -----------DATF--G-KE--VMKYEMPRPN-----------IGIHRFVFLLYKQ---
AtSCPYI-1       -----------DVSF--G-KE--IIGYEMPRPN-----------IGIHRFVYLLFKQ---
FvSCPYI-3       -----------DNTF--G-KE--VVKYEMPRPN-----------IGIHRFVFLLFKQ---
VvSCPYI-6       -----------DSTF--G-KE--IVNYEMPRPN-----------IGIHRFVFLLFKQ---
MtSCPYI-5       -----------DATF--G-KE--VVSYEIPKPN-----------IGIHRFVFVLFKQ---
VvSCPYI-5       -----------DATF--G-RE--VLSYEIPKPN-----------IGIHRFVFVLFKQ---
OsSCPYI-13      -----------DASF--G-RE--IISYESPKPS-----------IGIHRFVFVLFKQ---
OsSCPYI-15      -----------DASF--G-RE--VISYESPKPN-----------IGIHRFIFVLFKQ---
SbSCPYI-9       -----------DASF--G-RE--VISYESPRPS-----------IGIHRFIFVLFKQ---
SbSCPYI-13      -----------DASF--G-RE--VISYESPRPN-----------IGIHRFIFVLFKQ---
SbSCPYI-11      -----------DASF--G-TE--LAMYESPKPY-----------IGIHRFVFVLFKQ---
OsSCPYI-5       -----------DASF--G-RE--VVSYESPKPN-----------IGIHRFVLVLFKQ---
SbSCPYI-7       -----------DASF--G-RE--VVSYETPKPN-----------IGIHRFIFVLFRQ---
OsSCPYI-4       -----------DASF--G-RE--VVSYESPRPN-----------IGIHRFILVLFRQ---
```

```
OsSCPYI-12      -----------SISF--G-EE--LILYEKPEPR-----------SGIHRMVFVLFRQ---
SbSCPYI-5       -----------GANF--G-QE--LMFYERPEPR-----------SGIHRMVFVLFRQ---
OsSCPYI-10      -----------DDSF--G-RE--IVTYESPSPT-----------MGIHRIVMVLYQQ---
SbSCPYI-17      -----------DDSF--G-RE--LIPYENPSPT-----------MGIHRIVLVLYQQ---
OsSCPYI-3       -----------DATY--G-RE--VVCYESPNPT-----------TGIHRMVLVLFRQ---
SbSCPYI-6       -----------DDTY--G-RE--VMCYEAPNPT-----------TGIHRMVLVLFRQ---
SbSCPYI-10      -----------DNTY--G-RE--MMCYEPPAPS-----------TGIHRMVLVLFQQ---
OsSCPYI-11      -----------DARF--G-NE--IVPYEAPRPP-----------AGIHRLVFVLFKQ---
SbSCPYI-2       -----------DIRF--G-NE--IVPYESPRPP-----------AGIHRIVFVLFKQ---
MtSCPYI-2       -----------SASF--G-KE--VVFYESPKPS-----------AGIHRFVIALFKQ---
OsSCPYI-17      -----------NASF--G-NE--IVSYESPKPT-----------AGIHRFVFVLFRQ---
SbSCPYI-8       -----------NVSY--G-NE--VVSYESPKPS-----------AGIHRFVFVLFRQ---
OsSCPYI-6       -----------NASY--G-NE--VVSYESPKPT-----------AGIHRFVFILFRQ---
SbSCPYI-12      -----------NASY--G-NE--IVSYENPKPT-----------AGIHRFVFVLFRQ---
OsSCPYI-16      -----------DARF--G-NE--IVPYESPRPT-----------AGIHRFVFILFRQ---
SbSCPYI-1       -----------DASF--G-NE--IVPYESPRPT-----------AGIHRFAFVLFRQ---
OsSCPYI-14      -----------DTSK--G-TE--VVAYESPRPT-----------AGIHRLVFIVFRQ---
SbSCPYI-14      -----------GANH--G-NE--VVAYESPRPS-----------AGIHRFVFIVFRQ---
MtSCPYI-4       -----------EVTF--G-NE--VVNYERPRPT-----------SGIHRFVFVLFRQ---
MtSCPYI-3       -----------GTTF--G-QE--IVSYESPRPA-----------SGIHRVIFVLFRQ---
MtSCPYI-6       -----------GTTF--G-QE--IVSYENPRPT-----------SGIHRVIFVLFRQ---
FvSCPYI-5       -----------GASF--G-QE--IVSYESPRPA-----------MGIHRFVSVLYRQ---
AtSCPYI-2       -----------GNAF--G-NE--VVCYESPRPP-----------SGIHRIVLVLFRQ---
AtSCPYI-4       -----------GTTF--G-NE--IVCYENPSPT-----------AGIHRVVFILFRQ---
SbSCPYI-3       -----------GVSF--G-TE--VVCYESPRPV-----------LGIHRMVFLLFQQ---
FvSCPYI-2       -----------GAVF--G-QE--IVCYESPRPT-----------AGIHRFLFVLFRQ---
VvSCPYI-2       -----------GANF--G-QE--IVCYESPRPT-----------AGIHRFVFVLFRQ---
OsSCPYI-2       -----------GVSF--G-TE--VVCYESPRPV-----------LGIHRLVFLLFEQ---
SbSCPYI-15      -----------GAAF--G-QE--VICYESPRPT-----------MGIHRFVLVLFQQ---
OsSCPYI-7       -----------GATF--G-QE--VMCYESPRPT-----------MGIHRLVFVLFQQ---
OsSCPYI-8       -----------AASF--G-QE--VMCYESPRPT-----------MGIHRLVFVLFQQ---
VvSCPYI-3       -----------DATQ--G-RE--VVPYMGPQPP-----------TGIHRYIFTLFKQ---
PpSCPYI-1       LPSAADQGAAKPRTS--G-KE--VVPYVGPCPP-----------IGIHRYIFVLFKQ---
PpSCPYI-4       FPRLNEL-IAPSKSC--G-RE--LVPYMGPRPP-----------VGIHRYIFVLFKQ---
PpSCPYI-5       FSWLSEQ-ATSTSSS--G-RE--LVPYIGPRPP-----------IGIHRYIFVLFKQ---
PpSCPYI-6       FSWLQEQ-VTHTSSS--G-RE--LVPYMGPRPP-----------IGIHRYAFILFKQ---
SmSCPYI-3       -----------DASQ--G-RE--ILQYIGPKPP-----------TGIHRYIFVVFRQ---
MtSCPYI-7       -----------NPKR--G-KE--ILPYIGPKPP-----------VGIHRYILVLFEQ---
AtSCPYI-3       -----------NPSR--G-KE--ILPYMEPRPP-----------VGIHRYILVLFRQ---
FvSCPYI-4       -----------NPIR--G-KE--ILPYVGPRPP-----------VGIHRYILVLFQQ---
VvSCPYI-4       -----------NATR--G-KE--ALPYVGPRPP-----------VGIHRYILVLFQQ---
OsSCPYI-1       -----------DASK--G-EE--MVEYMGPRPT-----------VGIHRYVLVLYEQ---
SbSCPYI-4       -----------DASK--G-EE--VVEYMGPRPP-----------VGIHRYVLVLFEQ---
OsSCPYI-9       -----------DPSQ--G-DV--VVPYMGPRPP-----------VGIHRYVMVLFQQ---
SbSCPYI-16      -----------DPSQ--GSET--VMPYLGPCPP-----------VGIHRYVLVVYQQ---
                                             *                 :     :

SmSCPYI-11      -----D-------------------------------------------TK-LKL-----
PpSCPYI-3       -----D-------------------------------------------AY-PKV-----
MpSCPYI-1       -----A-------------------------------------------GE-KPI-----
SmSCPYI-15      -----Q-------------------------------------------GQ---------
SmSCPYI-14      -----Q-------------------------------------------GQ---------
SmSCPYI-16      -----Q-------------------------------------------GQ---------
SmSCPYI-17      -----Q-------------------------------------------GQ---------
SmSCPYI-18      --------------------------------------------------R-NPL-----
SmSCPYI-1       -----RNPLEEQGHWALVSIKHNIHFTLRMLLKHHAKEEPYKIPSLWVGGRIPPV-----
SmSCPYI-6       --------------------------------------------------R-NPLEEQGH
SmSCPYI-2       --------------------------------------------------R-NPL-----
SmSCPYI-10      --------------------------------------------------R-NPL-----
SmSCPYI-8       --------------------------------------------------R-NPL-----
SmSCPYI-9       --------------------------------------------------R-NPL-----
SmSCPYI-5       --------------------------------------------------R-NPL-----
SmSCPYI-7       --------------------------------------------------R-NPL-----
PpSCPYI-2       -----P-------------------------------------------G--QLV-----
MpSCPYI-2       AEVRAD-------------------------------------------GR-GVE-----
CsSCPYI-1       ----PK-------------------------------------------GT-NLN-----
SmSCPYI-12      -----K-------------------------------------------GK-ITA-----
SmSCPYI-13      ----KQ-------------------------------------------IH-AGS-----
SmSCPYI-4       -------------------------------------------------SR-PMM-----
AtSCPYI-5       -----R-------------------------------------------GR-QAV-----
VvSCPYI-1       -----R-------------------------------------------GR-ETV-----
FvSCPYI-1       ----TR-------------------------------------------GR-QTV-----
MtSCPYI-8       -----R-------------------------------------------GR-QTV-----
AtSCPYI-6       -----K-------------------------------------------QR-RVI-----
MtSCPYI-1       -----K-------------------------------------------RR-QTV-----
AtSCPYI-1       -----T-------------------------------------------RR-GSV-----
FvSCPYI-3       -----K-------------------------------------------GR-QTV-----
VvSCPYI-6       -----K-------------------------------------------RR-QTV-----
MtSCPYI-5       -----K-------------------------------------------NR-ESV-----
VvSCPYI-5       -----K-------------------------------------------RR-QTV-----
OsSCPYI-13      -----K-------------------------------------------RR-QAV-----
OsSCPYI-15      -----K-------------------------------------------RR-QTV-----
SbSCPYI-9       -----K-------------------------------------------RR-QTV-----
```

```
SbSCPYI-13      -----K-------------------------------------------GR-QTV-----
SbSCPYI-11      -----K-------------------------------------------SR-QSV-----
OsSCPYI-5       -----K-------------------------------------------RR-QAV-----
SbSCPYI-7       -----K-------------------------------------------RR-QAV-----
OsSCPYI-4       -----K-------------------------------------------RR-QAV-----
OsSCPYI-12      -----L-------------------------------------------GR-RTV-----
SbSCPYI-5       -----L-------------------------------------------GR-GTV-----
OsSCPYI-10      -----L-------------------------------------------GR-GTV-----
SbSCPYI-17      -----L-------------------------------------------GR-GTV-----
OsSCPYI-3       -----L-------------------------------------------GR-ETV-----
SbSCPYI-6       -----L-------------------------------------------GR-ETV-----
SbSCPYI-10      -----L-------------------------------------------GR-DTV-----
OsSCPYI-11      -----E-------------------------------------------AR-QTV-----
SbSCPYI-2       -----Q-------------------------------------------AR-QTV-----
MtSCPYI-2       -----L-------------------------------------------GR-DTV-----
OsSCPYI-17      -----S-------------------------------------------VQ-QTI-----
SbSCPYI-8       -----S-------------------------------------------VR-QTI-----
OsSCPYI-6       -----Y-------------------------------------------VQ-QTI-----
SbSCPYI-12      -----S-------------------------------------------VQ-QTV-----
OsSCPYI-16      -----S-------------------------------------------VR-QTT-----
SbSCPYI-1       -----S-------------------------------------------VR-QTT-----
OsSCPYI-14      -----T-------------------------------------------VR-QSI-----
SbSCPYI-14      -----A-------------------------------------------IR-QSI-----
MtSCPYI-4       -----Q-------------------------------------------CR-QRV-----
MtSCPYI-3       -----P-------------------------------------------CR-HTV-----
MtSCPYI-6       -----P-------------------------------------------CR-HTV-----
FvSCPYI-5       -----L-------------------------------------------GR-KTV-----
AtSCPYI-2       -----L-------------------------------------------GR-QTV-----
AtSCPYI-4       -----L-------------------------------------------GR-QTV-----
SbSCPYI-3       -----L-------------------------------------------GR-QTV-----
FvSCPYI-2       -----L-------------------------------------------GR-QTV-----
VvSCPYI-2       -----L-------------------------------------------GR-QTV-----
OsSCPYI-2       -----L-------------------------------------------GR-QTV-----
SbSCPYI-15      -----L-------------------------------------------GR-QTV-----
OsSCPYI-7       -----L-------------------------------------------GR-QTV-----
OsSCPYI-8       -----L-------------------------------------------GR-QTV-----
VvSCPYI-3       -----K-------------------------------------------AAAMSG-----
PpSCPYI-1       -----PT------------------------------------------GK-PLL-----
PpSCPYI-4       -----P-------------------------------------------LT-PFH-----
PpSCPYI-5       -----P-------------------------------------------SQ-SFL-----
PpSCPYI-6       -----P-------------------------------------------ST-PFL-----
SmSCPYI-3       -----M-------------------------------------------GP-VLM-----
MtSCPYI-7       -----K-------------------------------------------GP-IGM-----
AtSCPYI-3       -----N-------------------------------------------SPVGLM-----
FvSCPYI-4       -----K-------------------------------------------AP-MGL-----
VvSCPYI-4       -----K-------------------------------------------AP-LGL-----
OsSCPYI-1       -----K-------------------------------------------ARFVDGA----
SbSCPYI-4       -----K-------------------------------------------TRVH-------
OsSCPYI-9       -----K-------------------------------------------AR---------
SbSCPYI-16      -----K-------------------------------------------AR---F-----


SmSCPYI-11      ---------------------GHKATKDKVEIAVQGHVLGETELIG--------------
PpSCPYI-3       ---------------------PKKPNVDRLKEAMEGHILKEAVLTG--------------
MpSCPYI-1       ---------------------VVSE------GAETQRR----------------------
SmSCPYI-15      ---------------------INTP-LADRTKIEKKRY----------------------
SmSCPYI-14      ---------------------INTPLADRFVK--DWEA----------------------
SmSCPYI-16      ---------------------INTPLADRFVK--DWEA----------------------
SmSCPYI-17      ---------------------INTPLADRFVN--DWEA----------------------
SmSCPYI-18      ---------------------VKPM---------DSRI----------------------
SmSCPYI-1       ---------------------ASLP-------RVAWEQ----------------------
SmSCPYI-6       WTLVSIKHKIHFTLRMLLKHHVEEA---------IWNDVREMKLIILEVIQFAEPYRPST
SmSCPYI-2       ---------------------VKPM---------DSRI----------------------
SmSCPYI-10      ---------------------VKPM---------DSRL----------------------
SmSCPYI-8       ---------------------VKPM---------DSRL----------------------
SmSCPYI-9       ---------------------VKPM---------DSRL----------------------
SmSCPYI-5       ---------------------VKPM---------DSRL----------------------
SmSCPYI-7       ---------------------VKPM---------DSRL----------------------
PpSCPYI-2       ---------------------VPTA---------HSND----------------------
MpSCPYI-2       ---------------------EPAA---------FERK----------------------
CsSCPYI-1       ---------------------VPAP---------ASRA----------------------
SmSCPYI-12      ---------------------GKI----------TRRQ----------------------
SmSCPYI-13      ---------------------LVGS---------LSRT----------------------
SmSCPYI-4       ---------------------ALAP---------VIRS----------------------
AtSCPYI-5       ---------------------KAAP---------ETRE----------------------
VvSCPYI-1       ---------------------MP-P---------ASRD----------------------
FvSCPYI-1       ---------------------RV-P---------ASRD----------------------
MtSCPYI-8       ---------------------RS-P---------SSRD----------------------
AtSCPYI-6       ---------------------FPNI---------PSRD----------------------
MtSCPYI-1       ---------------------MKIP---------TSRD----------------------
AtSCPYI-1       ---------------------VSVP---------SYRD----------------------
FvSCPYI-3       ---------------------IP-P---------PSKD----------------------
VvSCPYI-6       ---------------------NP-P---------SSRD----------------------
```

```
MtSCPYI-5       ---------------------TASP---------SSRD----------------------
VvSCPYI-5       ---------------------NT-P---------TSRD----------------------
OsSCPYI-13      ---------------------VV-P---------SSRD----------------------
OsSCPYI-15      ---------------------IV-P---------SFRD----------------------
SbSCPYI-9       ---------------------AM-P---------SSRD----------------------
SbSCPYI-13      ---------------------TV-P---------SSRD----------------------
SbSCPYI-11      ---------------------RP-P---------SSRD----------------------
OsSCPYI-5       ---------------------TP-P---------SSRD----------------------
SbSCPYI-7       ---------------------NP-P---------SSKD----------------------
OsSCPYI-4       ---------------------SP-P---------PSRD----------------------
OsSCPYI-12      ---------------------FA-P---------EKRH----------------------
SbSCPYI-5       ---------------------FA-P---------DMRH----------------------
OsSCPYI-10      ---------------------FA-P---------QVRQ----------------------
SbSCPYI-17      ---------------------FA-P---------QVRQ----------------------
OsSCPYI-3       ---------------------YA-P---------AVRH----------------------
SbSCPYI-6       ---------------------YA-P---------SWRH----------------------
SbSCPYI-10      ---------------------FAAP---------SRRH----------------------
OsSCPYI-11      ---------------------YA-P---------GWRQ----------------------
SbSCPYI-2       ---------------------YA-P---------GWRQ----------------------
MtSCPYI-2       ---------------------FA-P---------DWRH----------------------
OsSCPYI-17      ---------------------YA-P---------GWRQ----------------------
SbSCPYI-8       ---------------------YA-P---------GWRQ----------------------
OsSCPYI-6       ---------------------YA-P---------GWRP----------------------
SbSCPYI-12      ---------------------YA-P---------GWRQ----------------------
OsSCPYI-16      ---------------------YA-P---------GWRQ----------------------
SbSCPYI-1       ---------------------YA-P---------GWRS----------------------
OsSCPYI-14      ---------------------YA-P---------GWRS----------------------
SbSCPYI-14      ---------------------YA-P---------GWRA----------------------
MtSCPYI-4       ---------------------YA-P---------GWRQ----------------------
MtSCPYI-3       ---------------------LA-P---------GWRQ----------------------
MtSCPYI-6       ---------------------LA-P---------GWRQ----------------------
FvSCPYI-5       ---------------------YA-P---------EWRQ----------------------
AtSCPYI-2       ---------------------YA-P---------GWRQ----------------------
AtSCPYI-4       ---------------------YA-P---------GWRQ----------------------
SbSCPYI-3       ---------------------YA-P---------GWRQ----------------------
FvSCPYI-2       ---------------------YA-P---------GWRQ----------------------
VvSCPYI-2       ---------------------YA-P---------GWRQ----------------------
OsSCPYI-2       ---------------------YA-P---------GWRQ----------------------
SbSCPYI-15      ---------------------YA-P---------GWRQ----------------------
OsSCPYI-7       ---------------------YA-P---------GWRQ----------------------
OsSCPYI-8       ---------------------YA-P---------GWRQ----------------------
VvSCPYI-3       ---------------------TLPP---------DTRS----------------------
PpSCPYI-1       ---------------------VTAP---------SVRN----------------------
PpSCPYI-4       ---------------------ITPP---------TVRS----------------------
PpSCPYI-5       ---------------------ISPP---------AARN----------------------
PpSCPYI-6       ---------------------ISPP---------TVRN----------------------
SmSCPYI-3       ---------------------LP-P---------LMRN----------------------
MtSCPYI-7       ---------------------VEQP---------TSRV----------------------
AtSCPYI-3       ---------------------VQQP---------PSRA----------------------
FvSCPYI-4       ---------------------VEQP---------PTRA----------------------
VvSCPYI-4       ---------------------VEQP---------GSRA----------------------
OsSCPYI-1       ---------------------LMPP---------ADRP----------------------
SbSCPYI-4       ---------------------AEAP---------RERA----------------------
OsSCPYI-9       ---------------------VAAP----PPDEDAARA----------------------
SbSCPYI-16      ---------------------RAPPVLAPGAEVEASRA----------------------


SmSCPYI-11      ----------HY----------TKEN-----F-STGHDSYV----PPPGGPL--------
PpSCPYI-3       ----------HY----------SKDQ-----Y-GTGNEKGY----YPSGVPQ--------
MpSCPYI-1       ----------EW----------DLKA-----F-LAKNPGAR-----AAAVNH--------
SmSCPYI-15      ----------DYSLEWCMEPGADRRNSHRITV-AAALNYSS-----PSIAKHAKPMKNGT
SmSCPYI-14      -------------------------------V-ASAYNLGS-----PVATTS--------
SmSCPYI-16      -------------------------------V-ASAYNLGS-----PVATTS--------
SmSCPYI-17      -------------------------------V-ASAYNLGS-----PVATTS--------
SmSCPYI-18      ----------VL----------SLAD-----I-IRQYHCGS-----PLQTVS--------
SmSCPYI-1       ----------LW----------RTAACS---LPTQQHSHSLNVQIWELLEQYVS------
SmSCPYI-6       RGVPFSLHGNNF----------DVQRPVAS-LLTQQHSHSL-NVWGPESRMN--------
SmSCPYI-2       ----------VN----------SLAD-----I-IQQYHCGS-----PLQTVS--------
SmSCPYI-10      ----------VN----------SLAD-----I-IGQYHCGS-----PLQTVS--------
SmSCPYI-8       ----------VN----------SLAD-----I-IQQYHCGS-----PLQTVS--------
SmSCPYI-9       ----------VN----------SLAD-----I-VQQYHCGS-----PLQTVS--------
SmSCPYI-5       ----------VN----------SLAD-----I-IQQYHCGS-----PLQTVS--------
SmSCPYI-7       ----------VN----------SLAD-----I-IQQYHCGS-----PLQTVS--------
PpSCPYI-2       ----------EI----------SARS-----F-AATHHL-T-----PVGALC--------
MpSCPYI-2       ----------RW----------DFKK-----F-LEQNPGLR-----PRAVNY--------
CsSCPYI-1       ----------RF----------NTQK-----F-SQLYDLGE-----PVAAAY--------
SmSCPYI-12      ----------QF----------SVRK-----F-SDEYSLGF-----PVGGVY--------
SmSCPYI-13      ----------LF----------SVRV-----F-TENYDLGY-----PVDGVF--------
SmSCPYI-4       ----------NF----------STKC-----F-AQEHGLGL-----AVAALY--------
AtSCPYI-5       ----------CF----------NTNA-----F-SSYFGLSQ-----PVAAVY--------
VvSCPYI-1       ----------HF----------NTRK-----F-AEDNGLGS-----PVAAVY--------
FvSCPYI-1       ----------NF----------NTRK-----F-SQENSLGL-----PVAAVY--------
MtSCPYI-8       ----------NF----------NTRR-----F-SQENNLGL-----PVAAVY--------
```

```
AtSCPYI-6       ----------HF----------NTRK-----F-AVEYDLGL-----PVAAVF--------
MtSCPYI-1       ----------LF----------NTKK-----F-AQDNDLGP-----PVAAVF--------
AtSCPYI-1       ----------QF----------NTRE-----F-AHENDLGL-----PVAAVF--------
FvSCPYI-3       ----------HF----------DSRK-----F-AETNEFGL-----PVAAVF--------
VvSCPYI-6       ----------RF----------STRN-----F-AEENELGP-----PVAAVF--------
MtSCPYI-5       ----------YF----------NTRN-----F-ASQNDLGL-----PVAAVY--------
VvSCPYI-5       ----------HF----------NTRS-----F-AAENDLGL-----PVAAVF--------
OsSCPYI-13      ----------HF----------NTRQ-----F-AEENELGL-----PVAAVY--------
OsSCPYI-15      ----------HF----------NTRR-----F-AEENDLGL-----PVAAVY--------
SbSCPYI-9       ----------HF----------ITRQ-----F-AEENDLGL-----PVAAVY--------
SbSCPYI-13      ----------HF----------NTRQ-----F-AEENDLGL-----PVAAVY--------
SbSCPYI-11      ----------YF----------STRR-----F-AADNDLGL-----PVAAVY--------
OsSCPYI-5       ----------YF----------STRR-----F-AADNDLGL-----PVAAVY--------
SbSCPYI-7       ----------RF----------STRQ-----F-AEDNDLGL-----PVAAVY--------
OsSCPYI-4       ----------RF----------STRQ-----F-AEDNDLGL-----PVAAVY--------
OsSCPYI-12      ----------NF----------NCRI-----F-ARQHHLNI------VAATY--------
SbSCPYI-5       ----------NF----------NCKN-----F-ARQYHLDI------VAATY--------
OsSCPYI-10      ----------NF----------NLRS-----F-ARRFNLGK-----PVAAMY--------
SbSCPYI-17      ----------NF----------NLRN-----F-ARRFNLGK-----PVAAMY--------
OsSCPYI-3       ----------NF----------TTRA-----F-ARRYNLGA-----PVAAVY--------
SbSCPYI-6       ----------NF----------STRG-----F-ARRYNLGA-----PVAAMY--------
SbSCPYI-10      ----------NF----------NTRA-----F-ARRYNLGA-----PVAAMF--------
OsSCPYI-11      ----------NF----------NVRD-----F-SAFYNLGP-----PVAALY--------
SbSCPYI-2       ----------NF----------NIRD-----F-SAIYNLGA-----PVAALY--------
MtSCPYI-2       ----------NF----------NTTN-----F-AEINNLVI------VASVY--------
OsSCPYI-17      ----------NF----------NTRD-----F-SALYNLGP-----PVAAVF--------
SbSCPYI-8       ----------NF----------NTRD-----F-SAFYNLGP-----PVASVF--------
OsSCPYI-6       ----------NF----------NTRD-----F-SALYNLGP-----PVAAVF--------
SbSCPYI-12      ----------NF----------NTRD-----F-SALYNLGP-----PVAAVF--------
OsSCPYI-16      ----------NF----------NTRD-----F-AELYNLGS-----PVAALF--------
SbSCPYI-1       ----------NF----------NTRD-----F-AAIYNLGS-----PVAAVY--------
OsSCPYI-14      ----------NF----------NTRD-----F-AACYSLGS-----PVAAAY--------
SbSCPYI-14      ----------NF----------NTRD-----F-AACYSLGP-----PVAATY--------
MtSCPYI-4       ----------NF----------NTRE-----F-AELYNLGS-----PVAAVF--------
MtSCPYI-3       ----------NF----------ITRD-----F-AEFYNLGL-----PVAALY--------
MtSCPYI-6       ----------NF----------ITRD-----F-AEFYNLGL-----PVAALY--------
FvSCPYI-5       ----------NF----------NTRK-----F-AENYNLGS-----PVAAVY--------
AtSCPYI-2       ----------QF----------NTRE-----F-AEIYNLGL-----PVAASY--------
AtSCPYI-4       ----------NF----------NTRE-----F-AEIYNLGL-----PVAAVF--------
SbSCPYI-3       ----------NF----------STRD-----F-AELYNLGL-----PVAAVY--------
FvSCPYI-2       ----------NF----------NTRD-----F-AELYNLGS-----PVAAVY--------
VvSCPYI-2       ----------NF----------NTRD-----F-AELYNLGL-----PVAAVY--------
OsSCPYI-2       ----------NF----------STRD-----F-AELYNLGL-----PVAAVY--------
SbSCPYI-15      ----------NF----------NTRD-----F-AELYNLGP-----PVAAVY--------
OsSCPYI-7       ----------NF----------STRN-----F-AELYNLGS-----PVATVY--------
OsSCPYI-8       ----------NF----------NTKD-----F-AELYNLGS-----PVAAVY--------
VvSCPYI-3       ----------NF----------STRQ-----F-AAGNGLGP-----PVALVY--------
PpSCPYI-1       ----------NF----------NTRT-----F-AVEHGLGF-----PVAATY--------
PpSCPYI-4       ----------NF----------NTRY-----F-AAQCGLGL-----PVAATY--------
PpSCPYI-5       ----------NF----------STRN-----F-AAYYGLGL-----PVAATY--------
PpSCPYI-6       ----------NF----------STRN-----F-ASHYGLGL-----PVAATY--------
SmSCPYI-3       ----------NF----------STRW-----F-AQEYFLGL-----PVGAVY--------
MtSCPYI-7       ----------SF----------NTRY-----F-ASQMNLGL-----PVATVY--------
AtSCPYI-3       ----------NF----------STRM-----F-AGHFDLGL-----PVATVY--------
FvSCPYI-4       ----------HF----------NTRY-----F-AAQLDLGL-----PVSTVY--------
VvSCPYI-4       ----------HF----------STRA-----F-ANQLDLGL-----PVATVY--------
OsSCPYI-1       ----------NF----------NTRA-----F-AAYHQLGL-----PTAVVH--------
SbSCPYI-4       ----------NF----------NTRA-----F-AAAHELGL-----PTAVVY--------
OsSCPYI-9       ----------RF----------STRA-----F-ADRHDLGL-----PVAALY--------
SbSCPYI-16      ----------RF----------RNRA-----F-ADRHDLGL-----PVAAMY--------


SmSCPYI-11      ----I-------------------------------------------------------
PpSCPYI-3       ----LSGPGRAQ-------------------------------LKSQHNNTRVATVAHQ-
MpSCPYI-1       ----LTCS-PPS-----------------------------------KDRRTGVEDDVHV
SmSCPYI-15      LEAEFLNPVTDV-----------------------------------IRKNNKAYPELWV
SmSCPYI-14      ----FVAQ-LTL-----------------------------------NFRTRMFQC----
SmSCPYI-16      ----FVAQ-LTL-----------------------------------NFRTRMFQC----
SmSCPYI-17      ----FVAQ-LTL-----------------------------------NFRTRMFQC----
SmSCPYI-18      ----FTTSLH--------------------------------------------------
SmSCPYI-1       ----LVAHGYPA---------------------------------GATCTSR--------
SmSCPYI-6       ----ELSPLW--------------------------------------------------
SmSCPYI-2       ----FTTSLA--------------------------------------------------
SmSCPYI-10      ----FTTSLH--------------------------------------------------
SmSCPYI-8       ----FTTSLY--------------------------------------------------
SmSCPYI-9       ----FTTSLH--------------------------------------------------
SmSCPYI-5       ----FTTSLY--------------------------------------------------
SmSCPYI-7       ----FTTSLY--------------------------------------------------
PpSCPYI-2       ----FMVA----------------------------------------------------
MpSCPYI-2       ----FVCS-RERH-----------------------------------------------
CsSCPYI-1       ----FEVA---------------------------------------APGIE--------
SmSCPYI-12      ----FTVE----------------------------------------AGVKVF------
SmSCPYI-13      ----F-------------------------------------------------------
```

```
SmSCPYI-4      ----FKAQGMEP------------------------------------------------
AtSCPYI-5      ----FNAQ-RET-----------------------------------APRRRPSY-----
VvSCPYI-1      ----FNAQ-RPT-----------------------------------AARRR--------
FvSCPYI-1      ----FNAQ-RET-----------------------------------AARRR--------
MtSCPYI-8      ----FNAQ-RET-----------------------------------AARRR--------
AtSCPYI-6      ----FNAQ-RET-----------------------------------AARKR--------
MtSCPYI-1      ----FNAQ-RET-----------------------------------AARRR--------
AtSCPYI-1      ----FNCQ-RET-----------------------------------AARRR--------
FvSCPYI-3      ----FNAQ-RET-----------------------------------AARRR--------
VvSCPYI-6      ----FNAQ-RET-----------------------------------AARKR--------
MtSCPYI-5      ----FNAQ-RET-----------------------------------AARRR--------
VvSCPYI-5      ----FNAQ-RET-----------------------------------AARRR--------
OsSCPYI-13     ----FNAQ-RET-----------------------------------AARRR--------
OsSCPYI-15     ----FNAQ-RET-----------------------------------AARRR--------
SbSCPYI-9      ----FNAQ-RET-----------------------------------AARRR--------
SbSCPYI-13     ----FNAQ-RET-----------------------------------AARRR--------
SbSCPYI-11     ----FNAQ-RET-----------------------------------AARRR--------
OsSCPYI-5      ----FNAQ-RET-----------------------------------AARRR--------
SbSCPYI-7      ----FNAQ-RET-----------------------------------AARRR--------
OsSCPYI-4      ----FNAQ-RET-----------------------------------AARRR--------
OsSCPYI-12     ----FNCQ-REA-----------------------------------GWGGRKFAPEGP-
SbSCPYI-5      ----FNCQ-REA-----------------------------------GSGGRRFRPESS-
OsSCPYI-10     ----FNCQ-RPT-----------------------------------GTGGRRPT-----
SbSCPYI-17     ----FNCQ-RQT-----------------------------------GTGGRRFT-----
OsSCPYI-3      ----FNCQ-RQA-----------------------------------GSGGRRFTGPYTS
SbSCPYI-6      ----FNCQ-RQN-----------------------------------GSGGRRFTGAYTG
SbSCPYI-10     ----FNCQ-RQT-----------------------------------GSGGPRFTGPYTS
OsSCPYI-11     ----FNCQ-KES-----------------------------------GVGGRR-------
SbSCPYI-2      ----FNCQ-KES-----------------------------------GVGGRR-------
MtSCPYI-2      ----FNCQ-RER-----------------------------------GCGGRRC------
OsSCPYI-17     ----FNCQ-REN-----------------------------------GCGGRRYIR----
SbSCPYI-8      ----FNCQ-REN-----------------------------------GCGGRRYIR----
OsSCPYI-6      ----FNCQ-REN-----------------------------------GCGGRRYIR----
SbSCPYI-12     ----FNCQ-REN-----------------------------------GCGGRRYIR----
OsSCPYI-16     ----FNCQ-REN-----------------------------------GCGGRRCVR----
SbSCPYI-1      ----FNCQ-REN-----------------------------------GCGGRRYIR----
OsSCPYI-14     ----FNCQ-REG-----------------------------------GCGGRRYRS----
SbSCPYI-14     ----FNCQ-REG-----------------------------------GCGGRRYR-----
MtSCPYI-4      ----FNCQ-RES-----------------------------------GSGGRTFR-----
MtSCPYI-3      ----FNCQ-REN-----------------------------------GSGGRRMVI----
MtSCPYI-6      ----FNCQ-REN-----------------------------------GSGGRRLII----
FvSCPYI-5      ----FNCQ-RET-----------------------------------GCGGRRTIM----
AtSCPYI-2      ----FNCQ-REN-----------------------------------GCGGRRT------
AtSCPYI-4      ----YNCQ-RES-----------------------------------GCGGRRL------
SbSCPYI-3      ----FNCQ-RES-----------------------------------GTGGRRM------
FvSCPYI-2      ----FNCQ-RES-----------------------------------GSGGRRRSS----
VvSCPYI-2      ----FNCQ-REG-----------------------------------GSGGRRS------
OsSCPYI-2      ----FNCQ-RES-----------------------------------GTGGRRM------
SbSCPYI-15     ----FNCQ-REA-----------------------------------GSGGRRMYS----
OsSCPYI-7      ----FNCQ-REA-----------------------------------GSGGRRVYP----
OsSCPYI-8      ----FNCQ-REA-----------------------------------GSGGRRVYP----
VvSCPYI-3      ----FNSQ-KER-----------------------------------TNRKH--------
PpSCPYI-1      ----FNAA-KEPENELDSEAATGPPWASPEGSLDPLVQHQVVAIPSRGQQCKSID-----
PpSCPYI-4      ----LNAQ-KEP-----------------------------------GSRRR--------
PpSCPYI-5      ----CNSQ-KEP-----------------------------------ASRSR--------
PpSCPYI-6      ----CNAQ-KEP-----------------------------------GSRRR--------
SmSCPYI-3      ----YNAQ-KEP-----------------------------------ASRRRT-------
MtSCPYI-7      ----FNSQ-KEP-----------------------------------QAKRR--------
AtSCPYI-3      ----FNAQ-KEP-----------------------------------ASRRR--------
FvSCPYI-4      ----FNAQ-KEP-----------------------------------ANRRR--------
VvSCPYI-4      ----FNAQ-KEP-----------------------------------ANRRR--------
OsSCPYI-1      ----FNSQ-REP-----------------------------------ANRRR--------
SbSCPYI-4      ----FNAQ-KEP-----------------------------------ANRRR--------
OsSCPYI-9      ----FNAQ-KEP-----------------------------------ANRRRRY------
SbSCPYI-16     ----FNAQ-KEP-----------------------------------ANRHRHY------


SmSCPYI-11     ---------------
PpSCPYI-3      ---------------
MpSCPYI-1      AEGHEPLTIDPLYVV
SmSCPYI-15     RMQGIHVSE------
SmSCPYI-14     ---------------
SmSCPYI-16     ---------------
SmSCPYI-17     ---------------
SmSCPYI-18     ---------------
SmSCPYI-1      ---------------
SmSCPYI-6      ---------------
SmSCPYI-2      ---------------
SmSCPYI-10     ---------------
SmSCPYI-8      ---------------
SmSCPYI-9      ---------------
SmSCPYI-5      ---------------
SmSCPYI-7      ---------------
```

```
PpSCPYI-2       ---------------
MpSCPYI-2       ---------------
CsSCPYI-1       ---------------
SmSCPYI-12      ---------------
SmSCPYI-13      ---------------
SmSCPYI-4       ---------------
AtSCPYI-5       ---------------
VvSCPYI-1       ---------------
FvSCPYI-1       ---------------
MtSCPYI-8       ---------------
AtSCPYI-6       ---------------
MtSCPYI-1       ---------------
AtSCPYI-1       ---------------
FvSCPYI-3       ---------------
VvSCPYI-6       ---------------
MtSCPYI-5       ---------------
VvSCPYI-5       ---------------
OsSCPYI-13      ---------------
OsSCPYI-15      ---------------
SbSCPYI-9       ---------------
SbSCPYI-13      ---------------
SbSCPYI-11      ---------------
OsSCPYI-5       ---------------
SbSCPYI-7       ---------------
OsSCPYI-4       ---------------
OsSCPYI-12      ---------------
SbSCPYI-5       ---------------
OsSCPYI-10      ---------------
SbSCPYI-17      ---------------
OsSCPYI-3       RRRQA----------
SbSCPYI-6       GRHG-----------
SbSCPYI-10      RRRAG----------
OsSCPYI-11      ---------------
SbSCPYI-2       ---------------
MtSCPYI-2       ---------------
OsSCPYI-17      ---------------
SbSCPYI-8       ---------------
OsSCPYI-6       ---------------
SbSCPYI-12      ---------------
OsSCPYI-16      ---------------
SbSCPYI-1       ---------------
OsSCPYI-14      ---------------
SbSCPYI-14      ---------------
MtSCPYI-4       ---------------
MtSCPYI-3       ---------------
MtSCPYI-6       ---------------
FvSCPYI-5       ---------------
AtSCPYI-2       ---------------
AtSCPYI-4       ---------------
SbSCPYI-3       ---------------
FvSCPYI-2       ---------------
VvSCPYI-2       ---------------
OsSCPYI-2       ---------------
SbSCPYI-15      ---------------
OsSCPYI-7       ---------------
OsSCPYI-8       ---------------
VvSCPYI-3       ---------------
PpSCPYI-1       ---------------
PpSCPYI-4       ---------------
PpSCPYI-5       ---------------
PpSCPYI-6       ---------------
SmSCPYI-3       ---------------
MtSCPYI-7       ---------------
AtSCPYI-3       ---------------
FvSCPYI-4       ---------------
VvSCPYI-4       ---------------
OsSCPYI-1       ---------------
SbSCPYI-4       ---------------
OsSCPYI-9       ---------------
SbSCPYI-16      ---------------
```
