# Supplementary material for: Plant protein peptidase inhibitors: an evolutionary overview based on comparative genomics
Source: BMC Genomics. 2014 Sep 25;15(1):812. doi: 10.1186/1471-2164-15-812 (PMC4189545; doi:10.1186/1471-2164-15-812)

Additional File 2. Phylograms of the peptidase inhibitor families. The amino acid sequences were aligned by MUSCLE and analysed with the PhyML method. Approximate likelihood-ratio test values are indicated. Rc, *Ricinus communis*; Pt, *Populus trichocarpa*; Mt, *Medicago truncatula*; Gm, *Glycine max*; Cs, *Cucumis sativus*; Ppe, *Prunus persica*; Fv, *Fragaria vesca*; At, *Arabidopsis thaliana*; Tc, *Theobroma cacao*; Vv, *Vitis vinifera*; Mg, *Mimulus guttatus*; Sb, *Sorghum bicolor*; Zm, *Zea mays*; Os, *Oryza sativa*; Bd, *Brachypodium distachyon*; Sm, *Selaginella moellendorffii*; Pp, *Physcomitrella patens*; Cr, *Chlamydomonas reinhardtii*; Vc, *Volvox carteri*; Csu, *Coccomyxa subellipsoidea*; Mp, *Micromonas pusilla*.

A. I1 Kazal.

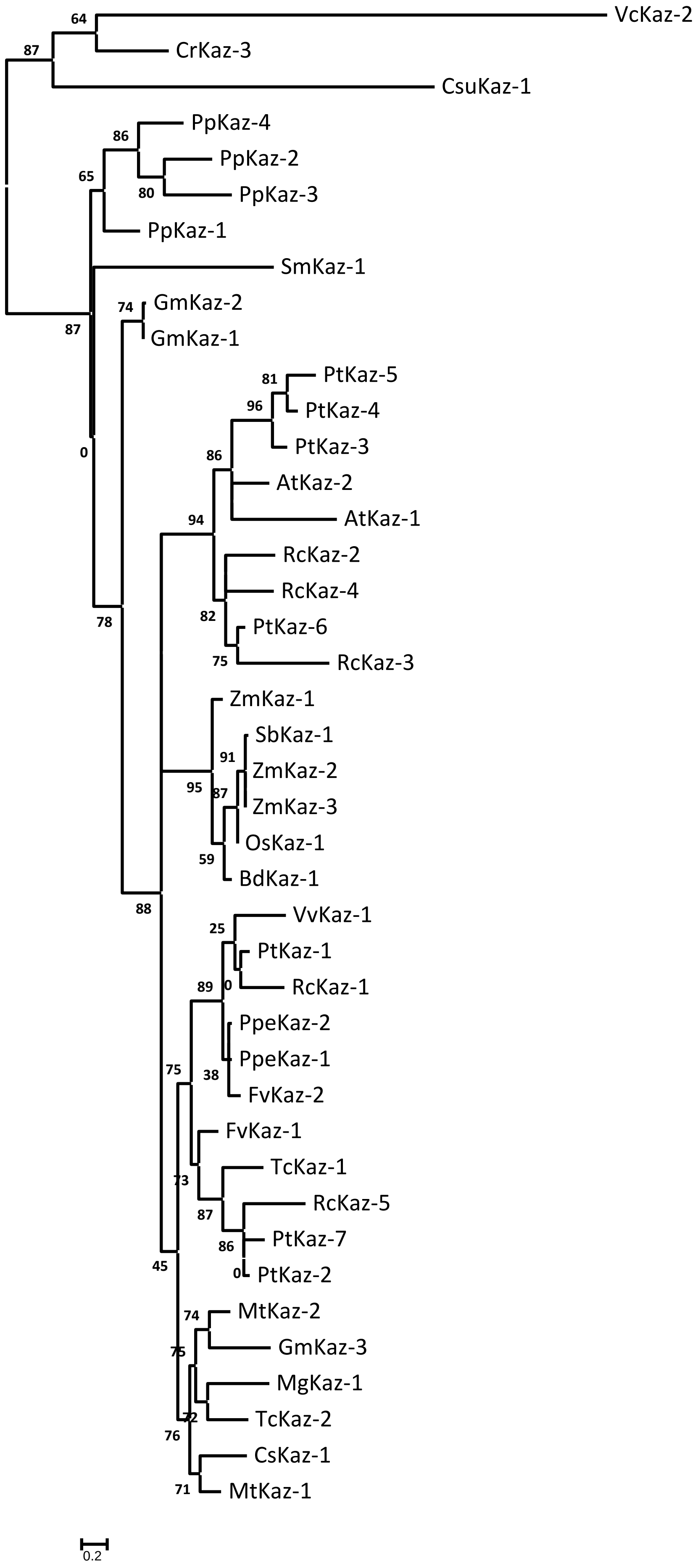
VcKaz-2
CrKaz-3
CsuKaz-1
PpKaz-4
PpKaz-2
PpKaz-3
PpKaz-1
SmKaz-1
GmKaz-2
GmKaz-1
PtKaz-5
PtKaz-4
PtKaz-3
AtKaz-2
AtKaz-1
RcKaz-2
RcKaz-4
PtKaz-6
RcKaz-3
ZmKaz-1
SbKaz-1
ZmKaz-2
ZmKaz-3
OsKaz-1
BdKaz-1
VvKaz-1
PtKaz-1
RcKaz-1
PpeKaz-2
PpeKaz-1
FvKaz-2
FvKaz-1
TcKaz-1
RcKaz-5
PtKaz-7
PtKaz-2
MtKaz-2
GmKaz-3
MgKaz-1
TcKaz-2
CsKaz-1
MtKaz-1
0.2

B. I3 Kunitz-P.

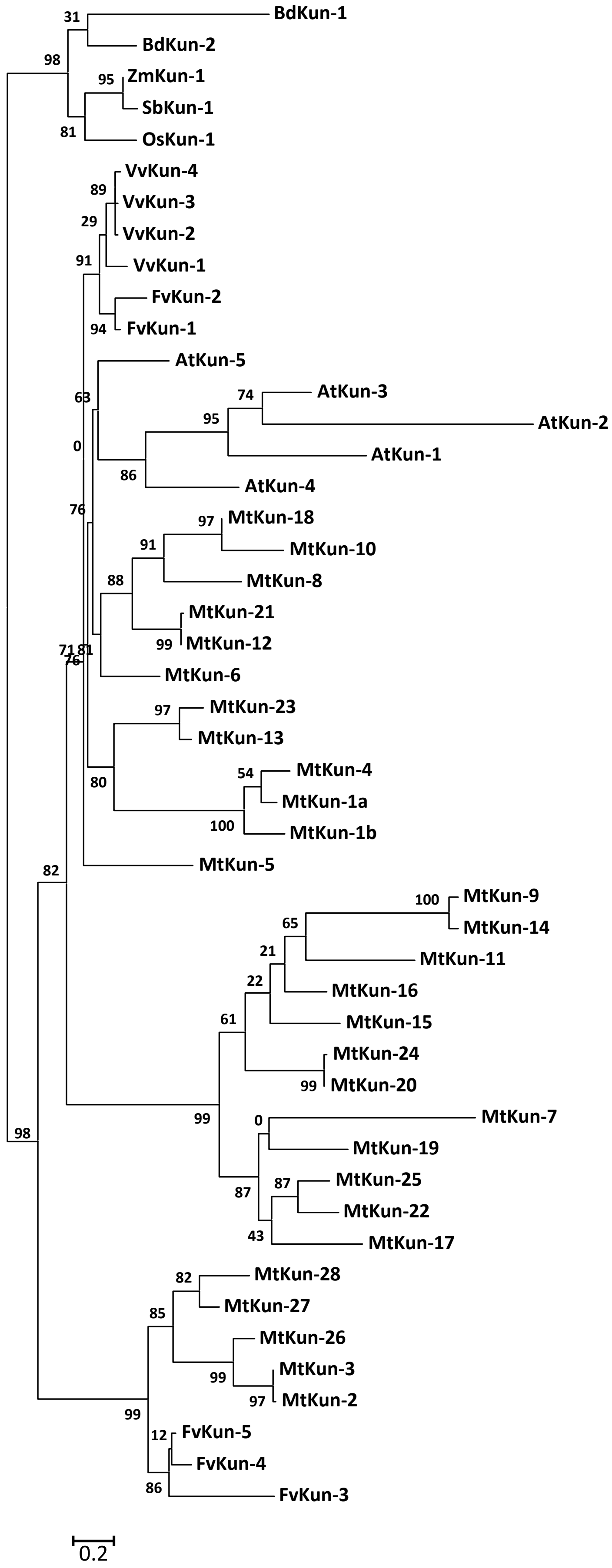
BdKun-1
BdKun-2
ZmKun-1
SbKun-1
OsKun-1
VvKun-4
VvKun-3
VvKun-2
VvKun-1
FvKun-2
FvKun-1
AtKun-5
AtKun-3
AtKun-2
AtKun-1
AtKun-4
MtKun-18
MtKun-10
MtKun-8
MtKun-21
MtKun-12
MtKun-6
MtKun-23
MtKun-13
MtKun-4
MtKun-1a
MtKun-1b
MtKun-5
MtKun-9
MtKun-14
MtKun-11
MtKun-16
MtKun-15
MtKun-24
MtKun-20
MtKun-7
MtKun-19
MtKun-25
MtKun-22
MtKun-17
MtKun-28
MtKun-27
MtKun-26
MtKun-3
MtKun-2
FvKun-5
FvKun-4
FvKun-3
0.2

C. I4 Serpin.

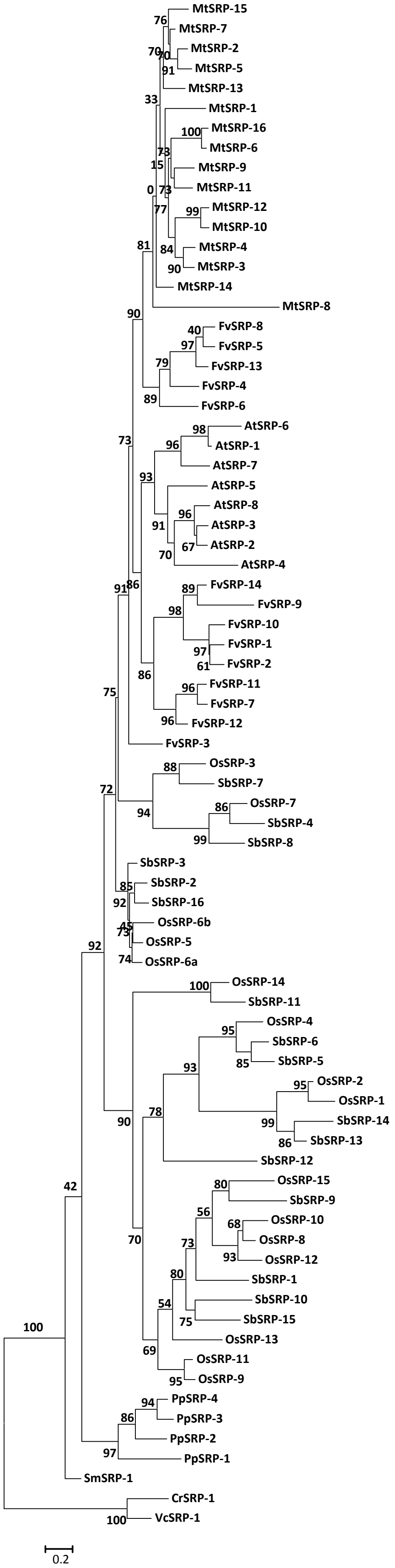
MtSRP-15
MtSRP-7
MtSRP-2
MtSRP-5
MtSRP-13
MtSRP-1
MtSRP-16
MtSRP-6
MtSRP-9
MtSRP-11
MtSRP-12
MtSRP-10
MtSRP-4
MtSRP-3
MtSRP-14
MtSRP-8
FvSRP-8
FvSRP-5
FvSRP-13
FvSRP-4
FvSRP-6
AtSRP-6
AtSRP-1
AtSRP-7
AtSRP-5
AtSRP-8
AtSRP-3
AtSRP-2
AtSRP-4
FvSRP-14
FvSRP-9
FvSRP-10
FvSRP-1
FvSRP-2
FvSRP-11
FvSRP-7
FvSRP-12
FvSRP-3
OsSRP-3
SbSRP-7
OsSRP-7
SbSRP-4
SbSRP-8
SbSRP-3
SbSRP-2
SbSRP-16
OsSRP-6b
OsSRP-5
OsSRP-6a
OsSRP-14
SbSRP-11
OsSRP-4
SbSRP-6
SbSRP-5
OsSRP-2
OsSRP-1
SbSRP-14
SbSRP-13
SbSRP-12
OsSRP-15
SbSRP-9
OsSRP-10
OsSRP-8
OsSRP-12
SbSRP-1
SbSRP-10
SbSRP-15
OsSRP-13
OsSRP-11
OsSRP-9
PpSRP-4
PpSRP-3
PpSRP-2
PpSRP-1
SmSRP-1
CrSRP-1
VcSRP-1
0.2

D. I6 Cereal.

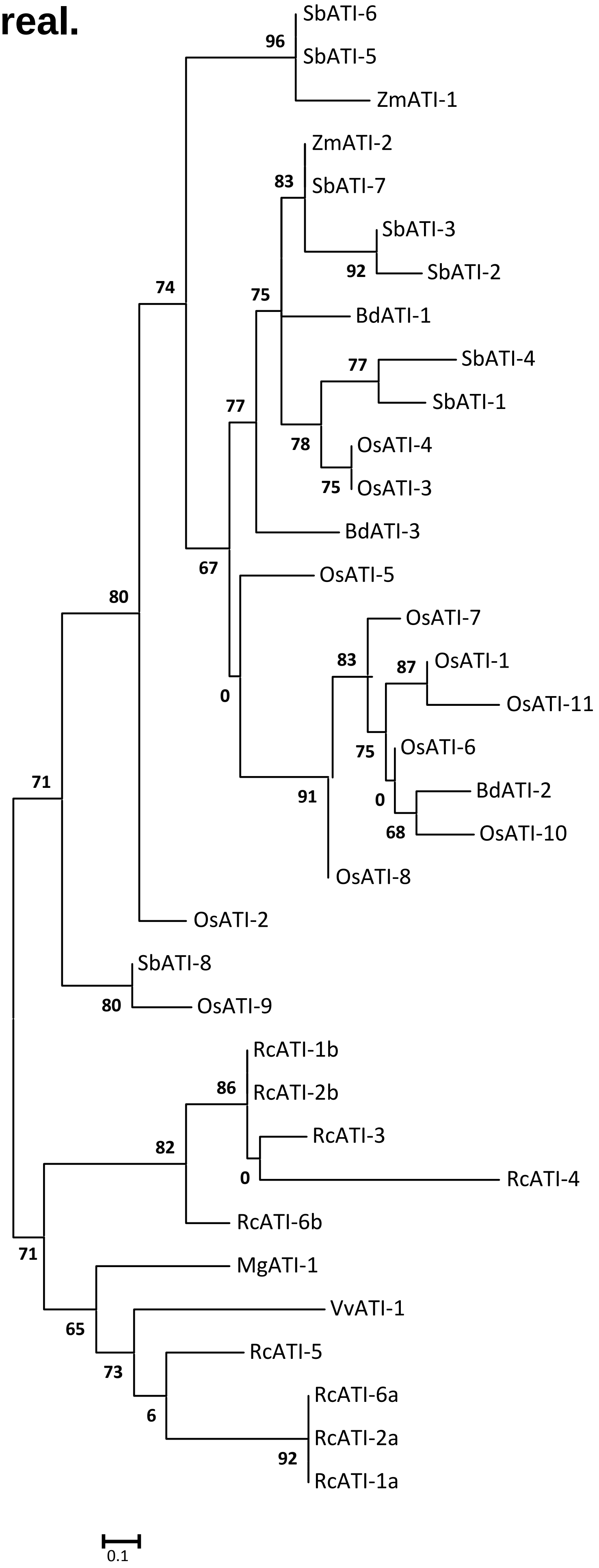
SbATI-6
96
SbATI-5
ZmATI-1
ZmATI-2
83
SbATI-7
SbATI-3
92
SbATI-2
74
75
BdATI-1
77
SbATI-4
77
SbATI-1
78
OsATI-4
75
OsATI-3
BdATI-3
67
OsATI-5
80
OsATI-7
83
87
OsATI-1
0
OsATI-11
75
OsATI-6
71
91
0
BdATI-2
68
OsATI-10
OsATI-8
OsATI-2
SbATI-8
80
OsATI-9
RcATI-1b
86
RcATI-2b
82
RcATI-3
0
RcATI-4
RcATI-6b
71
MgATI-1
VvATI-1
65
RcATI-5
73
6
RcATI-6a
RcATI-2a
92
RcATI-1a
0.1

E. I12 Bowman-Birk.

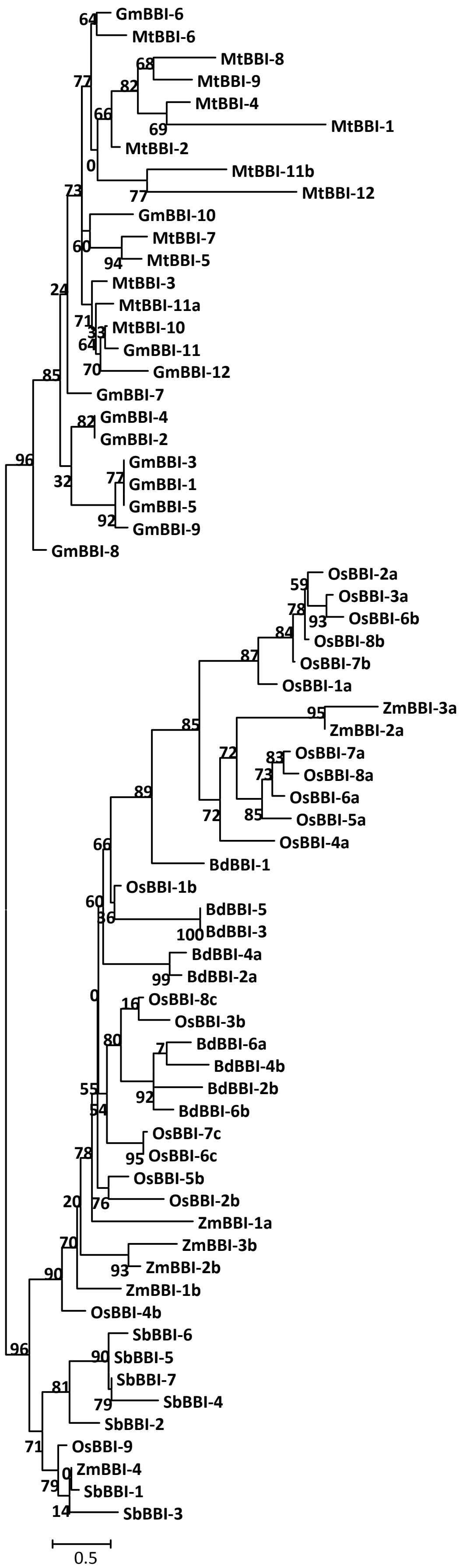

F. I13 Pin-I.

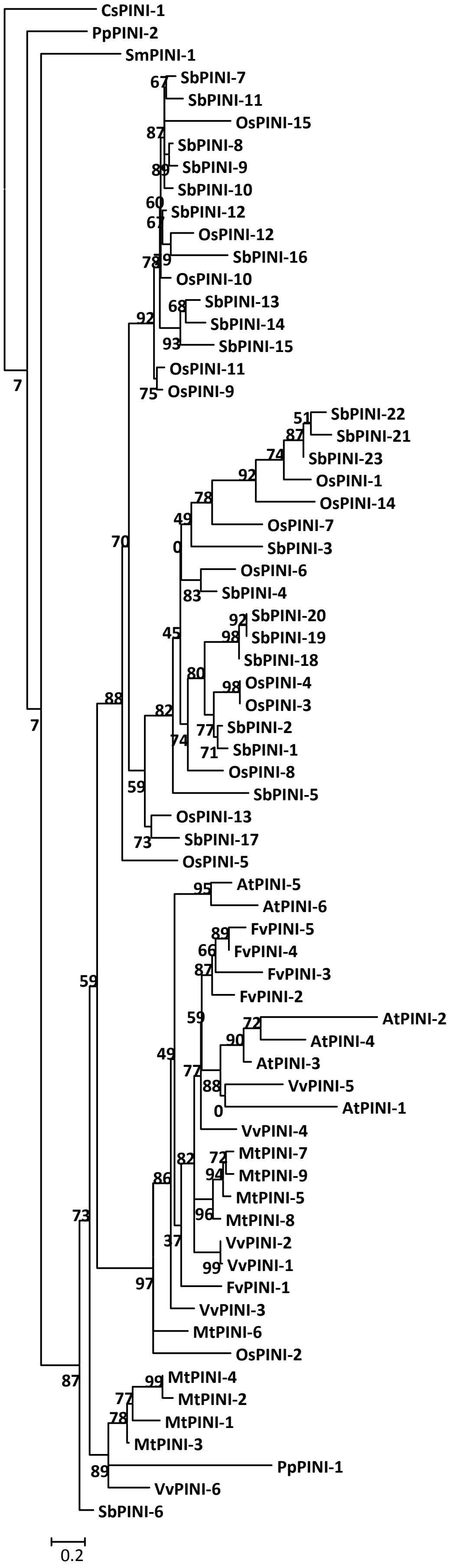
CsPINI-1
PpPINI-2
SmPINI-1
SbPINI-7
SbPINI-11
OsPINI-15
SbPINI-8
SbPINI-9
SbPINI-10
SbPINI-12
OsPINI-12
SbPINI-16
OsPINI-10
SbPINI-13
SbPINI-14
SbPINI-15
OsPINI-11
OsPINI-9
SbPINI-22
SbPINI-21
SbPINI-23
OsPINI-1
OsPINI-14
OsPINI-7
SbPINI-3
OsPINI-6
SbPINI-4
SbPINI-20
SbPINI-19
SbPINI-18
OsPINI-4
OsPINI-3
SbPINI-2
SbPINI-1
OsPINI-8
SbPINI-5
OsPINI-13
SbPINI-17
OsPINI-5
AtPINI-5
AtPINI-6
FvPINI-5
FvPINI-4
FvPINI-3
FvPINI-2
AtPINI-2
AtPINI-4
AtPINI-3
VvPINI-5
AtPINI-1
VvPINI-4
MtPINI-7
MtPINI-9
MtPINI-5
MtPINI-8
VvPINI-2
VvPINI-1
FvPINI-1
VvPINI-3
MtPINI-6
OsPINI-2
MtPINI-4
MtPINI-2
MtPINI-1
MtPINI-3
PpPINI-1
VvPINI-6
SbPINI-6
0.2

G. I20 Pin-II.

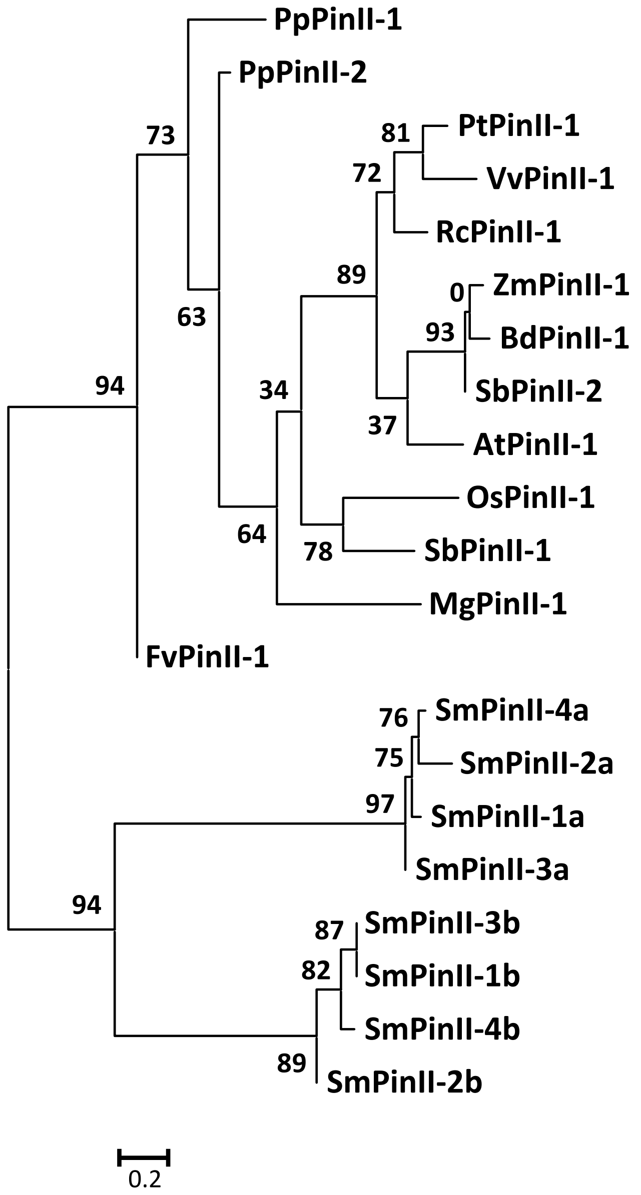

PpPinII-1
PpPinII-2
PtPinII-1
VvPinII-1
RcPinII-1
ZmPinII-1
BdPinII-1
SbPinII-2
AtPinII-1
OsPinII-1
SbPinII-1
MgPinII-1
FvPinII-1
SmPinII-4a
SmPinII-2a
SmPinII-1a
SmPinII-3a
SmPinII-3b
SmPinII-1b
SmPinII-4b
SmPinII-2b
73
63
94
81
72
89
0
93
34
37
64
78
76
75
97
94
87
82
89
0.2

H. I25 Cystatin.

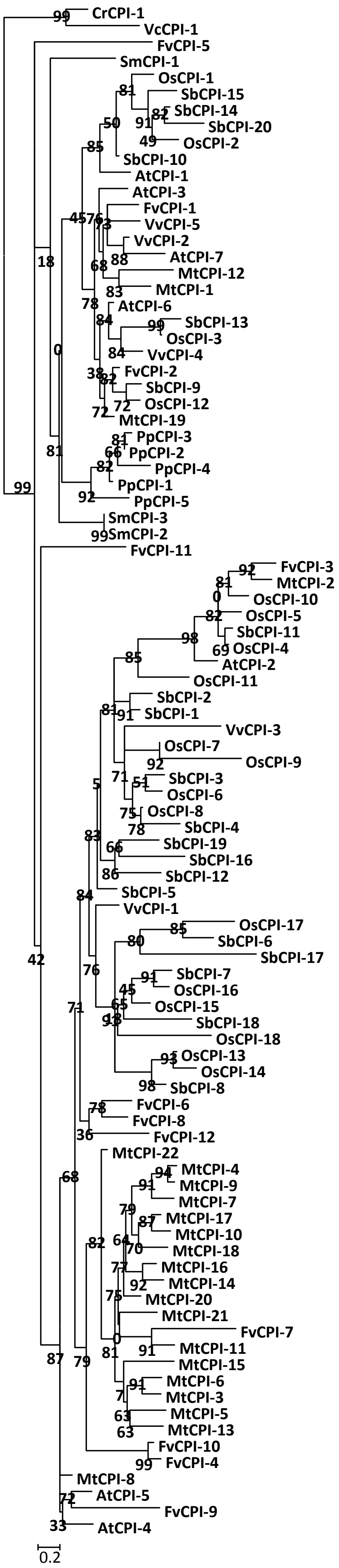

I. I51 Serine Carboxypeptidase Y Inhibitors (SCPYInh).

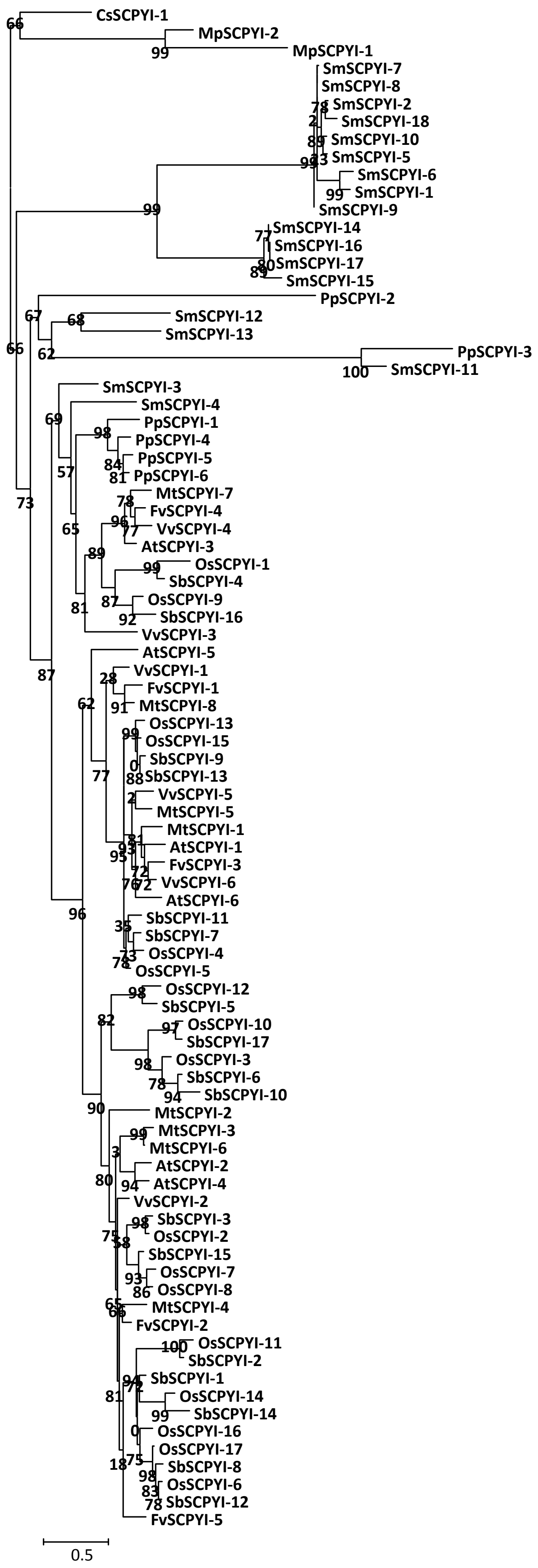

Supplement: Supplementary file 2 — Additional file 2: Phylograms of the peptidase inhibitor families. (PDF 214 KB) [file 12864_2014_6493_MOESM2_ESM.pdf]
