# Supplementary material for: Plant protein peptidase inhibitors: an evolutionary overview based on comparative genomics
Source: BMC Genomics. 2014 Sep 25;15(1):812. doi: 10.1186/1471-2164-15-812 (PMC4189545; doi:10.1186/1471-2164-15-812)
Supplement: Supplementary file 3 — Additional file 3: Information about the sequences used in the phylogenetic trees. (PDF 43 KB) [file 12864_2014_6493_MOESM3_ESM.pdf]

Additional file 3. Information about the sequences used in the phylogenetic trees.

A. I1 Kazal sequences.

**Protein**	**Organism**	**Gene model**
AtKaz-1	*Arabidopsis thaliana*	AT4G01575.1
AtKaz-2	*Arabidopsis thaliana*	AT3G61980.1
CsKaz-1	*Cucumis sativus*	Cucsa.174730.1
FvKaz-1	*Fragaria vesca*	mrna12428.1-v1.0-hybrid
FvKaz-2	*Fragaria vesca*	mrna21627.1-v1.0-hybrid
GmKaz-1	*Glycine max*	Glyma14g36800.1
GmKaz-2	*Glycine max*	Glyma02g38630.1
GmKaz-3	*Glycine max*	Glyma04g40413.1
MtKaz-1	*Medicago truncatula*	Medtr3g093240.1
MtKaz-2	*Medicago truncatula*	Medtr5g068760.1
MgKaz-1	*Mimulus guttatus*	mgv1a025397m
PtKaz-1	*Populus trichocarpa*	Potri.015G001800.1
PtKaz-2	*Populus trichocarpa*	Potri.002G182800.1
PtKaz-3	*Populus trichocarpa*	Potri.014G109000.1
PtKaz-4	*Populus trichocarpa*	Potri.014G108900.1
PtKaz-5	*Populus trichocarpa*	Potri.014G108800.1
PtKaz-6	*Populus trichocarpa*	Potri.014G108700.1
PtKaz-7	*Populus trichocarpa*	Potri.014G108600.1
PpeKaz-1	*Prunus persica*	ppa014626m
PpeKaz-2	*Prunus persica*	ppa013184m
RcKaz-1	*Ricinus communis*	30154.m001128_RICCO
RcKaz-2	*Ricinus communis*	30076.m004498_RICCO
RcKaz-3	*Ricinus communis*	30076.m004499_RICCO
RcKaz-4	*Ricinus communis*	30076.m004497_RICCO
RcKaz-5	*Ricinus communis*	30076.m004496_RICCO
TcKaz-1	*Theobroma cacao*	Thecc1EG005000t1
TcKaz-2	*Theobroma cacao*	Thecc1EG015543t1
VvKaz-1	*Vitis vinifera*	GSVIVP00037147001_VITVI
BdKaz-1	*Brachypodium distachyon*	Bradi2g52990.1_BRADI
OsKaz-1	*Oryza sativa*	LOC_Os01g59610.1
SbKaz-1	*Sorghum bicolor*	Sb03g037650.1
ZmKaz-1	*Zea mays*	GRMZM2G144936_T01
ZmKaz-2	*Zea mays*	GRMZM2G028266_T01
ZmKaz-3	*Zea mays*	GRMZM2G324806_T01
SmKaz-1	*Selaginella moellendorffii*	414607
PpKaz-1	*Physcomitrella patens*	Pp1s80_198V6.2
PpKaz-2	*Physcomitrella patens*	Pp1s70_36V6.1
PpKaz-3	*Physcomitrella patens*	Pp1s227_63V6.1
PpKaz-4	*Physcomitrella patens*	Pp1s288_41V6.1
CrKaz-3	*Chlamydomonas reinhardtii*	Cre07.g329500.t2.1a
CrKaz-3	*Chlamydomonas reinhardtii*	Cre07.g329500.t2.1b
CrKaz-3	*Chlamydomonas reinhardtii*	Cre06.g267750.t1.2a
CsuKaz-1	*Coccomyxa subellipsoidea*	Ext_fgenesh1_pg.C_80144 47526

VcKaz-2	*Volvox carteri*	Vocar20000301m.a

B. I3 Kunitz-P sequences.

**Protein**	**Organism**	**Gene model**
MtKun-1a	*Medicago truncatula*	Medtr3g106580.1a
MtKun-1b	*Medicago truncatula*	Medtr3g106580.1b
MtKun-2	*Medicago truncatula*	Medtr3g014820.1
MtKun-3	*Medicago truncatula*	Medtr3g014870.1
MtKun-4	*Medicago truncatula*	Medtr3g106620.1
MtKun-5	*Medicago truncatula*	Medtr7g037410.1
MtKun-6	*Medicago truncatula*	Medtr6g078290.1
MtKun-7	*Medicago truncatula*	Medtr6g059790.1
MtKun-8	*Medicago truncatula*	Medtr6g078250.1
MtKun-9	*Medicago truncatula*	Medtr6g044810.1
MtKun-10	*Medicago truncatula*	Medtr6g078260.1
MtKun-11	*Medicago truncatula*	Medtr6g059760.1b
MtKun-12	*Medicago truncatula*	Medtr6g078120.1
MtKun-13	*Medicago truncatula*	Medtr6g078070.1
MtKun-14	*Medicago truncatula*	Medtr6g044780.1
MtKun-15	*Medicago truncatula*	Medtr6g065460.1
MtKun-16	*Medicago truncatula*	Medtr6g059650.1
MtKun-17	*Medicago truncatula*	Medtr6g009650.1
MtKun-18	*Medicago truncatula*	Medtr6g078280.1
MtKun-19	*Medicago truncatula*	Medtr6g059810.1
MtKun-20	*Medicago truncatula*	Medtr6g059530.1
MtKun-21	*Medicago truncatula*	Medtr6g078140.1
MtKun-22	*Medicago truncatula*	Medtr6g059680.1
MtKun-23	*Medicago truncatula*	Medtr6g078100.1
MtKun-24	*Medicago truncatula*	Medtr6g059410.1
MtKun-25	*Medicago truncatula*	Medtr6g059730.1
MtKun-26	*Medicago truncatula*	Medtr8g059790.1
MtKun-27	*Medicago truncatula*	AC235677_10.1
MtKun-28	*Medicago truncatula*	AC235677_27.1
AtKun-1	*Arabidopsis thaliana*	AT1G73330.1
AtKun-2	*Arabidopsis thaliana*	AT1G72290.1
AtKun-3	*Arabidopsis thaliana*	AT1G73325.1
AtKun-4	*Arabidopsis thaliana*	AT1G73260.1
AtKun-5	*Arabidopsis thaliana*	AT1G17860.1
FvKun-1	*Fragaria vesca*	gene08979-v1.0
FvKun-2	*Fragaria vesca*	gene08978-v1.0
FvKun-3	*Fragaria vesca*	gene27334-v1.0
FvKun-4	*Fragaria vesca*	gene27360-v1.0
FvKun-5	*Fragaria vesca*	gene27359-v1.0
VvKun-1	*Vitis vinifera*	GSVIVT01012920001
VvKun-2	*Vitis vinifera*	GSVIVT01012922001

VvKun-3	*Vitis vinifera*	GSVIVT01012932001
VvKun-4	*Vitis vinifera*	GSVIVT01012936001
BdKun-1	*Brachypodium distachyon*	Bradi4g01470.1
BdKun-2	*Brachypodium distachyon*	Bradi5g16290.1
OsKun-1	*Oryza sativa*	LOC_Os04g44470.1
SbKun-1	*Sorghum bicolor*	Sb06g023160.1
ZmKun-1	*Zea mays*	GRMZM2G474575_T01

C. I4 Serpin sequences.

**Protein**	**Organism**	**Gene model**
MtSRP-1	*Medicago truncatula*	Medtr3g100520.1
MtSRP-2	*Medicago truncatula*	Medtr3g101030.1
MtSRP-3	*Medicago truncatula*	Medtr3g101150.1
MtSRP-4	*Medicago truncatula*	Medtr3g101180.1
MtSRP-5	*Medicago truncatula*	Medtr3g101050.1
MtSRP-6	*Medicago truncatula*	Medtr7g050750.1
MtSRP-7	*Medicago truncatula*	Medtr3g101010.1
MtSRP-8	*Medicago truncatula*	Medtr3g114310.1
MtSRP-9	*Medicago truncatula*	Medtr3g101130.1
MtSRP-10	*Medicago truncatula*	Medtr3g015760.1
MtSRP-11	*Medicago truncatula*	Medtr3g101200.1
MtSRP-12	*Medicago truncatula*	Medtr3g015620.1
MtSRP-13	*Medicago truncatula*	Medtr3g111160.1
MtSRP-14	*Medicago truncatula*	Medtr3g018740.1
MtSRP-15	*Medicago truncatula*	Medtr3g100990.1
MtSRP-16	*Medicago truncatula*	Medtr7g050810.1
FvSRP-1	*Fragaria vesca*	mrna16217.1-v1.0-hybrid
FvSRP-2	*Fragaria vesca*	mrna31740.1-v1.0-hybrid
FvSRP-3	*Fragaria vesca*	mrna15046.1-v1.0-hybrid
FvSRP-4	*Fragaria vesca*	mrna01845.1-v1.0-hybrid
FvSRP-5	*Fragaria vesca*	mrna32429.1-v1.0-hybrid
FvSRP-6	*Fragaria vesca*	mrna10757.1-v1.0-hybrid
FvSRP-7	*Fragaria vesca*	mrna03508.1-v1.0-hybrid
FvSRP-8	*Fragaria vesca*	mrna30734.1-v1.0-hybrid
FvSRP-9	*Fragaria vesca*	mrna28549.1-v1.0-hybrid
FvSRP-10	*Fragaria vesca*	mrna29691.1-v1.0-hybrid
FvSRP-11	*Fragaria vesca*	mrna07851.1-v1.0-hybrid
FvSRP-12	*Fragaria vesca*	mrna07850.1-v1.0-hybrid
FvSRP-13	*Fragaria vesca*	mrna30732.1-v1.0-hybrid
FvSRP-14	*Fragaria vesca*	mrna28546.1-v1.0-hybrid
AtSRP-1	*Arabidopsis thaliana*	AT2G14540.1
AtSRP-2	*Arabidopsis thaliana*	AT2G26390.1
AtSRP-3	*Arabidopsis thaliana*	AT2G25240.1
AtSRP-4	*Arabidopsis thaliana*	AT2G35580.1
AtSRP-5	*Arabidopsis thaliana*	AT1G47710.1
AtSRP-6	*Arabidopsis thaliana*	AT1G62170.2
AtSRP-7	*Arabidopsis thaliana*	AT1G64030.1

AtSRP-8	*Arabidopsis thaliana*	AT3G45220.1
SbSRP-1	*Sorghum bicolor*	Sb01g006070.1
SbSRP-2	*Sorghum bicolor*	Sb01g014730.1
SbSRP-3	*Sorghum bicolor*	Sb01g014740.1
SbSRP-4	*Sorghum bicolor*	Sb02g004040.1
SbSRP-5	*Sorghum bicolor*	Sb03g010560.1
SbSRP-6	*Sorghum bicolor*	Sb03g010570.1
SbSRP-7	*Sorghum bicolor*	Sb03g035570.1
SbSRP-8	*Sorghum bicolor*	Sb04g001380.1
SbSRP-9	*Sorghum bicolor*	Sb05g007550.1
SbSRP-10	*Sorghum bicolor*	Sb05g008400.1
SbSRP-11	*Sorghum bicolor*	Sb05g008490.1
SbSRP-12	*Sorghum bicolor*	Sb05g022570.1
SbSRP-13	*Sorghum bicolor*	Sb06g023640.1
SbSRP-14	*Sorghum bicolor*	Sb06g023660.1
SbSRP-15	*Sorghum bicolor*	Sb07g025180.1
SbSRP-16	*Sorghum bicolor*	Sb09g000460.1
OsSRP-1	*Oryza sativa*	Os04g45120.1
OsSRP-2	*Oryza sativa*	Os04g45110.1
OsSRP-3	*Oryza sativa*	Os01g56010.1
OsSRP-4	*Oryza sativa*	Os01g16200.1
OsSRP-5	*Oryza sativa*	Os03g41438.1
OsSRP-6a	*Oryza sativa*	Os03g41419.1 a
OsSRP-6b	*Oryza sativa*	Os03g41419.1 b
OsSRP-7	*Oryza sativa*	Os05g43590.1
OsSRP-8	*Oryza sativa*	Os11g12410.1
OsSRP-9	*Oryza sativa*	Os11g13530.1
OsSRP-10	*Oryza sativa*	Os11g12460.1
OsSRP-11	*Oryza sativa*	Os11g13540.1
OsSRP-12	*Oryza sativa*	Os11g12420.1
OsSRP-13	*Oryza sativa*	Os11g11720.1
OsSRP-14	*Oryza sativa*	Os11g11500.1
OsSRP-15	*Oryza sativa*	Os11g11760.1
SmSRP-1	*Selaginella moellendorffii*	173749
PpSRP-1	*Physcomitrella patens*	Pp019G069200.1
PpSRP-2	*Physcomitrella patens*	Pp011G040300.1
PpSRP-3	*Physcomitrella patens*	Pp001G054300.1
PpSRP-4	*Physcomitrella patens*	Pp002G113700.1
CrSRP-1	*Chlamydomonas reinhardtii*	Cre16.g679550.t1.1
VcSRP-1	*Volvox carteri*	Vocar20000111m

D. I6 Cereal sequences.

**Protein**	**Organism**	**Gene model**
MgATI-1	*Mimulus guttatus*	gmgv1a015473m
RcATI-1a	*Ricinus communis*	t00004428166.m001081a
RcATI-1b	*Ricinus communis*	t00004428166.m001081b
RcATI-2a	*Ricinus communis*	t00004528166.m001082a

RcATI-2b	*Ricinus communis*	t00004528166.m001082b
RcATI-3	*Ricinus communis*	t00004628166.m001083
RcATI-4	*Ricinus communis*	t00002230156.m001719
RcATI-5	*Ricinus communis*	t00003628166.m001073
RcATI-6a	*Ricinus communis*	t00004228166.m001079a
RcATI-6b	*Ricinus communis*	t00004228166.m001079b
VvATI-1	*Vitis vinifera*	GSVIVT01001973001
BdATI-1	*Brachypodium distachyon*	Bradi1g53970.1
BdATI-2	*Brachypodium distachyon*	Bradi1g54000.1
BdATI-3	*Brachypodium distachyon*	Bradi1g53990.1
OsATI-1	*Oryza sativa*	LOC_Os07g11380.1
OsATI-2	*Oryza sativa*	LOC_Os07g12080.1
OsATI-3	*Oryza sativa*	LOC_Os07g11650.1
OsATI-4	*Oryza sativa*	LOC_Os07g11630.1
OsATI-5	*Oryza sativa*	LOC_Os07g11310.1
OsATI-6	*Oryza sativa*	LOC_Os07g11330.1
OsATI-7	*Oryza sativa*	LOC_Os07g11510.1
OsATI-8	*Oryza sativa*	LOC_Os07g11410.1
OsATI-9	*Oryza sativa*	LOC_Os05g41970.1
OsATI-10	*Oryza sativa*	Os07g11320.1_ORYSA
OsATI-11	*Oryza sativa*	Os07g11360.1_ORYSA
SbATI-1	*Sorghum bicolor*	Sb02g002495.1
SbATI-2	*Sorghum bicolor*	Sb02g002500.1
SbATI-3	*Sorghum bicolor*	Sb02g006470.1
SbATI-4	*Sorghum bicolor*	Sb02g006480.1
SbATI-5	*Sorghum bicolor*	Sb02g006550.1
SbATI-6	*Sorghum bicolor*	Sb02g006560.1
SbATI-7	*Sorghum bicolor*	Sb02g006570.1
SbATI-8	*Sorghum bicolor*	Sb09g024570.1
ZmATI-1	*Zea mays*	GRMZM2G404688_T01
ZmATI-2	*Zea mays*	GRMZM2G304548_T01

E. I12 Bowman-Birk sequences.

**Protein**	**Organism**	**Gene model**
GmBBI-1	*Glycine max*	Glyma14g26400.1
GmBBI-2	*Glycine max*	Glyma14g26410.1
GmBBI-3	*Glycine max*	Glyma09g28720.1
GmBBI-4	*Glycine max*	Glyma09g28730.1
GmBBI-5	*Glycine max*	Glyma09g28713.1
GmBBI-6	*Glycine max*	Glyma09g39640.1
GmBBI-7	*Glycine max*	Glyma09g39630.1
GmBBI-8	*Glycine max*	Glyma09g39621.1
GmBBI-9	*Glycine max*	Glyma16g33400.1
GmBBI-10	*Glycine max*	Glyma18g46550.1
GmBBI-11	*Glycine max*	Glyma18g46560.1
GmBBI-12	*Glycine max*	Glyma18g46580.1
MtBBI-1	*Medicago truncatula*	Medtr7g077320.1

MtBBI-2	*Medicago truncatula*	Medtr7g077300.1
MtBBI-3	*Medicago truncatula*	Medtr7g077340.1
MtBBI-4	*Medicago truncatula*	Medtr7g077260.1
MtBBI-5	*Medicago truncatula*	Medtr7g077190.1
MtBBI-6	*Medicago truncatula*	Medtr7g077310.1
MtBBI-7	*Medicago truncatula*	Medtr7g077160.1
MtBBI-8	*Medicago truncatula*	Medtr7g077220.1
MtBBI-9	*Medicago truncatula*	Medtr7g077230.1
MtBBI-10	*Medicago truncatula*	Medtr7g077180.1
MtBBI-11a	*Medicago truncatula*	Medtr7g077210.1
MtBBI-11b	*Medicago truncatula*	Medtr7g077210.1
MtBBI-12	*Medicago truncatula*	Medtr7g077350.1
BdBBI-1	*Brachypodium distachyon*	Bradi3g22860.1
BdBBI-2a	*Brachypodium distachyon*	Bradi2g01920.1
BdBBI-2b	*Brachypodium distachyon*	Bradi2g01920.1
BdBBI-3	*Brachypodium distachyon*	Bradi1g50670.2
BdBBI-4a	*Brachypodium distachyon*	Bradi2g01930.1
BdBBI-4b	*Brachypodium distachyon*	Bradi2g01930.1
BdBBI-5	*Brachypodium distachyon*	Bradi1g50670.1
BdBBI-6a	*Brachypodium distachyon*	Bradi2g01910.1
BdBBI-6b	*Brachypodium distachyon*	Bradi2g01910.1
OsBBI-1a	*Oryza sativa*	LOC_Os01g03380.1
OsBBI-1b	*Oryza sativa*	LOC_Os01g03380.1
OsBBI-2a	*Oryza sativa*	LOC_Os01g03310.1
OsBBI-2b	*Oryza sativa*	LOC_Os01g03310.1
OsBBI-3a	*Oryza sativa*	LOC_Os01g03390.1
OsBBI-3b	*Oryza sativa*	LOC_Os01g03390.1
OsBBI-4a	*Oryza sativa*	LOC_Os01g03680.1
OsBBI-4b	*Oryza sativa*	LOC_Os01g03680.1
OsBBI-5a	*Oryza sativa*	LOC_Os01g03320.1
OsBBI-5b	*Oryza sativa*	LOC_Os01g03320.1
OsBBI-6a	*Oryza sativa*	LOC_Os01g03360.1
OsBBI-6b	*Oryza sativa*	LOC_Os01g03360.1
OsBBI-6c	*Oryza sativa*	LOC_Os01g03360.1
OsBBI-7a	*Oryza sativa*	LOC_Os01g03340.1
OsBBI-7b	*Oryza sativa*	LOC_Os01g03340.1
OsBBI-7c	*Oryza sativa*	LOC_Os01g03340.1
OsBBI-8a	*Oryza sativa*	LOC_Os01g03330.1
OsBBI-8b	*Oryza sativa*	LOC_Os01g03330.1
OsBBI-8c	*Oryza sativa*	LOC_Os01g03330.1
OsBBI-9	*Oryza sativa*	LOC_Os03g60840.1
SbBBI-1	*Sorghum bicolor*	Sb01g003000.1
SbBBI-2	*Sorghum bicolor*	Sb03g039790.1
SbBBI-3	*Sorghum bicolor*	Sb03g039800.1
SbBBI-4	*Sorghum bicolor*	Sb05g026650.1
SbBBI-5	*Sorghum bicolor*	Sb05g026670.1
SbBBI-6	*Sorghum bicolor*	Sb05g026680.1
SbBBI-7	*Sorghum bicolor*	Sb05g026700.1

ZmBBI-1a	*Zea mays*	GRMZM2G114552_T01
ZmBBI-1b	*Zea mays*	GRMZM2G114552_T01
ZmBBI-2a	*Zea mays*	AC208221.3_FGT002
ZmBBI-2b	*Zea mays*	AC208221.3_FGT002
ZmBBI-3a	*Zea mays*	GRMZM2G447785_T02
ZmBBI-3b	*Zea mays*	GRMZM2G447785_T02
ZmBBI-4	*Zea mays*	AC212728.3_FGT001

F. I13 Pin-I sequences.

**Protein**	**Organism**	**Gene model**
MtPINI-1	*Medicago truncatula*	Medtr3g020800.1
MtPINI-2	*Medicago truncatula*	Medtr3g020930.1
MtPINI-3	*Medicago truncatula*	Medtr3g020970.1
MtPINI-4	*Medicago truncatula*	Medtr3g020880.1
MtPINI-5	*Medicago truncatula*	Medtr5g090250.1
MtPINI-6	*Medicago truncatula*	Medtr5g045470.1
MtPINI-7	*Medicago truncatula*	Medtr1g075340.1
MtPINI-8	*Medicago truncatula*	Medtr1g075380.1
MtPINI-9	*Medicago truncatula*	Medtr1g075410.1
FvPINI-1	*Fragaria vesca*	mrna32568.1-v1.0-hybrid
FvPINI-2	*Fragaria vesca*	mrna32558.1-v1.0-hybrid
FvPINI-3	*Fragaria vesca*	mrna32561.1-v1.0-hybrid
FvPINI-4	*Fragaria vesca*	mrna32563.1-v1.0-hybrid
FvPINI-5	*Fragaria vesca*	mrna32562.1-v1.0-hybrid
AtPINI-1	*Arabidopsis thaliana*	AT3G46860.1
AtPINI-2	*Arabidopsis thaliana*	AT3G50020.1
AtPINI-3	*Arabidopsis thaliana*	AT5G43580.1
AtPINI-4	*Arabidopsis thaliana*	AT5G43570.1
AtPINI-5	*Arabidopsis thaliana*	AT2G38870.1
AtPINI-6	*Arabidopsis thaliana*	AT2G38900.2
VvPINI-1	*Vitis vinifera*	GSVIVT01018137001
VvPINI-2	*Vitis vinifera*	GSVIVT01018139001
VvPINI-3	*Vitis vinifera*	GSVIVT01018141001
VvPINI-4	*Vitis vinifera*	GSVIVT01018142001
VvPINI-5	*Vitis vinifera*	GSVIVT01032743001
VvPINI-6	*Vitis vinifera*	GSVIVT01034125001
SbPINI-1	*Sorghum bicolor*	Sb04g001965.1
SbPINI-2	*Sorghum bicolor*	Sb04g001970.1
SbPINI-3	*Sorghum bicolor*	Sb04g002002.1
SbPINI-4	*Sorghum bicolor*	Sb04g002030.1
SbPINI-5	*Sorghum bicolor*	Sb04g002045.1
SbPINI-6	*Sorghum bicolor*	Sb08g005570.1
SbPINI-7	*Sorghum bicolor*	Sb09g001010.1
SbPINI-8	*Sorghum bicolor*	Sb09g001020.1

SbPINI-9	*Sorghum bicolor*	Sb09g001030.1
SbPINI-10	*Sorghum bicolor*	Sb09g001040.1
SbPINI-11	*Sorghum bicolor*	Sb09g001050.1
SbPINI-12	*Sorghum bicolor*	Sb09g001060.1
SbPINI-13	*Sorghum bicolor*	Sb09g001070.1
SbPINI-14	*Sorghum bicolor*	Sb09g001080.1
SbPINI-15	*Sorghum bicolor*	Sb09g001083.1
SbPINI-16	*Sorghum bicolor*	Sb09g001086.1
SbPINI-17	*Sorghum bicolor*	Sb09g017500.1
SbPINI-18	*Sorghum bicolor*	Sb04g001980.1
SbPINI-19	*Sorghum bicolor*	Sb04g001990.1
SbPINI-20	*Sorghum bicolor*	Sb1445s002010.1
SbPINI-21	*Sorghum bicolor*	Sb04g002020.1
SbPINI-22	*Sorghum bicolor*	Sb04g002040.1
SbPINI-23	*Sorghum bicolor*	Sb04g002050.1
OsPINI-1	*Oryza sativa*	Os02g03170.1
OsPINI-2	*Oryza sativa*	Os06g21540.1
OsPINI-3	*Oryza sativa*	Os08g34258.1
OsPINI-4	*Oryza sativa*	Os08g34249.1
OsPINI-5	*Oryza sativa*	Os01g42860.1
OsPINI-6	*Oryza sativa*	Os02g03140.1
OsPINI-7	*Oryza sativa*	Os02g03190.1
OsPINI-8	*Oryza sativa*	Os02g03150.1
OsPINI-9	*Oryza sativa*	Os12g36210.1
OsPINI-10	*Oryza sativa*	Os12g25090.2
OsPINI-11	*Oryza sativa*	Os12g36240.1
OsPINI-12	*Oryza sativa*	Os05g01920.1
OsPINI-13	*Oryza sativa*	Os11g17790.1
OsPINI-14	*Oryza sativa*	Os02g03180.1
OsPINI-15	*Oryza sativa*	Os12g36220.1
SmPINI-1	*Selaginella moellendorffii*	84301
PpPINI-1	*Physcomitrella patens*	Pp017G009600.1
PpPINI-2	*Physcomitrella patens*	Pp023G080500.1
CsuPINI-1	*Coccomyxa subellipsoidea*	60469

G. I20 Pin-II sequences.

**Protein**	**Organism**	**Gene model**
RcPinII-1	*Ricinus communis*	30074.m001422
PtPinII-1	*Populus trichocarpa*	Potri.019G080600.1
PpPinII-1	*Prunus persica*	ppa017948m
PpPinII-2	*Prunus persica*	ppb022308m
FvPinII-1	*Fragaria vesca*	mrna25667.1-v1.0-hybrid
AtPinII-1	*Arabidopsis thaliana*	AT1G72060.1

VvPinII-1	*Vitis vinifera*	GSVIVT01023245001
MgPinII-1	*Mimulus guttatus*	mgv1a025135m
SbPinII-1	*Sorghum bicolor*	Sb01g008960.1
SbPinII-2	*Sorghum bicolor*	Sb03g009370.1
ZmPinII-1	*Zea mays*	GRMZM2G108847_T01
OsPinII-1	*Oryza sativa*	Os03g52390.1
BdPinII-1	*Brachypodium distachyon*	Bradi2g08720.1_BRADI
SmPinII-4a	*Selaginella moellendorffii*	444521
SmPinII-4b	*Selaginella moellendorffii*	444521
SmPinII-1a	*Selaginella moellendorffii*	438273
SmPinII-1b	*Selaginella moellendorffii*	438273
SmPinII-2a	*Selaginella moellendorffii*	438274
SmPinII-2b	*Selaginella moellendorffii*	438274
SmPinII-3a	*Selaginella moellendorffii*	438284
SmPinII-3b	*Selaginella moellendorffii*	438284

H. I25 Cystatin sequences.

**Protein**	**Organism**	**Gene model**
AtCPI-2	*Arabidopsis thaliana*	AT2G31980.1
AtCPI-3	*Arabidopsis thaliana*	AT2G40880.1
AtCPI-4	*Arabidopsis thaliana*	AT4G16500.1
AtCPI-6	*Arabidopsis thaliana*	AT3G12490.2
AtCPI-5	*Arabidopsis thaliana*	AT5G47550.1
AtCPI-1	*Arabidopsis thaliana*	AT5G12140.1
AtCPI-7	*Arabidopsis thaliana*	AT5G05110.1
CrCPI-1	*Chlamydomonas reinhardtii*	Cre04.g225650.t1.2
VcCPI-1	*Volvox carteri*	Vocar20011820m
FvCPI-1	*Fragaria vesca*	mrna21484.1-v1.0-hybrid
FvCPI-2	*Fragaria vesca*	mrna21476.1-v1.0-hybrid
FvCPI-3	*Fragaria vesca*	mrna06633.1-v1.0-hybrid
FvCPI-4	*Fragaria vesca*	mrna25711.1-v1.0-hybrid
FvCPI-5	*Fragaria vesca*	mrna34164.1-v1.0-hybrid
FvCPI-6	*Fragaria vesca*	mrna19785.1-v1.0-hybrid
FvCPI-7	*Fragaria vesca*	mrna13968.1-v1.0-hybrid
FvCPI-8	*Fragaria vesca*	mrna29824.1-v1.0-hybrid
FvCPI-9	*Fragaria vesca*	mrna16463.1-v1.0-hybrid
FvCPI-10	*Fragaria vesca*	mrna25708.1-v1.0-hybrid
FvCPI-11	*Fragaria vesca*	mrna20093.1-v1.0-hybrid
FvCPI-12	*Fragaria vesca*	mrna29817.1-v1.0-hybrid
OsCPI-10	*Oryza sativa*	Os04g28250.1
OsCPI-1	*Oryza sativa*	Os01g58890.1
OsCPI-12	*Oryza sativa*	Os01g16430.1
OsCPI-5	*Oryza sativa*	Os01g68670.1

OsCPI-4	*Oryza sativa*	Os01g68660.1
OsCPI-6	*Oryza sativa*	Os03g11180.1
OsCPI-7	*Oryza sativa*	Os03g11170.1
OsCPI-9	*Oryza sativa*	Os03g11160.1
OsCPI-13	*Oryza sativa*	Os03g07620.1
OsCPI-8	*Oryza sativa*	Os03g31510.1
OsCPI-11	*Oryza sativa*	Os09g08100.1
OsCPI-2	*Oryza sativa*	Os05g41460.1
OsCPI-14	*Oryza sativa*	Os03g07610.1
OsCPI-15	*Oryza sativa*	Os03g07660.1
OsCPI-16	*Oryza sativa*	Os03g07650.1
OsCPI-17	*Oryza sativa*	Os03g07700.1
OsCPI-18	*Oryza sativa*	Os02g17770.1
OsCPI-3	*Oryza sativa*	Os05g33880.2
PpCPI-1	*Physcomitrella patens*	Pp1s66_209V6.2
PpCPI-2	*Physcomitrella patens*	Pp1s212_8V6.1
PpCPI-4	*Physcomitrella patens*	Pp1s238_59V6.1
PpCPI-3	*Physcomitrella patens*	Pp1s238_60V6.1
PpCPI-5	*Physcomitrella patens*	Pp1s142_12V6.1
SbCPI-1	*Sorghum bicolor*	Sb01g031440.1
SbCPI-2	*Sorghum bicolor*	Sb01g031450.1
SbCPI-3	*Sorghum bicolor*	Sb01g031460.1
SbCPI-4	*Sorghum bicolor*	Sb01g031470.1
SbCPI-5	*Sorghum bicolor*	Sb01g043320.1
SbCPI-6	*Sorghum bicolor*	Sb01g045590.1
SbCPI-7	*Sorghum bicolor*	Sb01g045610.1
SbCPI-8	*Sorghum bicolor*	Sb01g045630.1
SbCPI-9	*Sorghum bicolor*	Sb03g010730.1
SbCPI-10	*Sorghum bicolor*	Sb03g037370.1
SbCPI-11	*Sorghum bicolor*	Sb03g043680.1
SbCPI-12	*Sorghum bicolor*	Sb04g026140.1
SbCPI-13	*Sorghum bicolor*	Sb09g020170.1
SbCPI-14	*Sorghum bicolor*	Sb09g024230.1
SbCPI-15	*Sorghum bicolor*	Sb09g024240.1
SbCPI-16	*Sorghum bicolor*	Sb1560s002010.1
SbCPI-17	*Sorghum bicolor*	Sb01g023010.1
SbCPI-18	*Sorghum bicolor*	Sb01g045600.1
SbCPI-19	*Sorghum bicolor*	Sb01g015950.1
SbCPI-20	*Sorghum bicolor*	Sb09g024410.1
MtCPI-1	*Medicago truncatula*	Medtr3g043750.1
MtCPI-2	*Medicago truncatula*	Medtr3g084780.1
MtCPI-3	*Medicago truncatula*	Medtr2g076740.1
MtCPI-4	*Medicago truncatula*	Medtr2g028060.1
MtCPI-5	*Medicago truncatula*	Medtr2g103740.1
MtCPI-6	*Medicago truncatula*	Medtr2g076800.1

MtCPI-7	*Medicago truncatula*	Medtr2g028100.1
MtCPI-8	*Medicago truncatula*	Medtr2g028520.1
MtCPI-9	*Medicago truncatula*	Medtr2g026040.1
MtCPI-10	*Medicago truncatula*	Medtr2g028080.1
MtCPI-11	*Medicago truncatula*	Medtr6g078440.1
MtCPI-12	*Medicago truncatula*	Medtr5g091360.1
MtCPI-13	*Medicago truncatula*	Medtr5g088770.1
MtCPI-14	*Medicago truncatula*	Medtr5g098560.1
MtCPI-15	*Medicago truncatula*	Medtr5g091260.1
MtCPI-16	*Medicago truncatula*	Medtr5g098550.1
MtCPI-17	*Medicago truncatula*	Medtr5g091280.1
MtCPI-18	*Medicago truncatula*	Medtr5g091520.1
MtCPI-19	*Medicago truncatula*	Medtr8g041410.1
MtCPI-20	*Medicago truncatula*	Medtr8g079410.1
MtCPI-21	*Medicago truncatula*	Medtr4g095630.1
MtCPI-22	*Medicago truncatula*	Medtr4g133540.1
SmCPI-1	*Selaginella moellendorffii*	19455
SmCPI-2	*Selaginella moellendorffii*	418098
SmCPI-3	*Selaginella moellendorffii*	85303
VvCPI-1	*Vitis vinifera*	GSVIVT01003320001
VvCPI-2	*Vitis vinifera*	GSVIVT01025801001
VvCPI-3	*Vitis vinifera*	GSVIVT01026080001
VvCPI-4	*Vitis vinifera*	GSVIVT01036582001
VvCPI-5	*Vitis vinifera*	GSVIVT01037389001

I. I51 Serine Carboxypeptidase Y inhibitor sequences.

**Protein**	**Organism**	**Gene model**
AtSCPYI-1	*Arabidopsis thaliana*	AT2G27550.1
AtSCPYI-2	*Arabidopsis thaliana*	AT4G20370.1
AtSCPYI-3	*Arabidopsis thaliana*	AT1G18100.1
AtSCPYI-4	*Arabidopsis thaliana*	AT1G65480.1
AtSCPYI-5	*Arabidopsis thaliana*	AT5G62040.1
AtSCPYI-6	*Arabidopsis thaliana*	AT5G03840.1
FvSCPYI-1	*Fragaria vesca*	mrna13304.1-v1.0-hybrid
FvSCPYI-2	*Fragaria vesca*	mrna21535.1-v1.0-hybrid
FvSCPYI-3	*Fragaria vesca*	mrna21992.1-v1.0-hybrid
FvSCPYI-4	*Fragaria vesca*	mrna09405.1-v1.0-hybrid
FvSCPYI-5	*Fragaria vesca*	mrna28959.1-v1.0-hybrid
MtSCPYI-1	*Medicago truncatula*	Medtr2g086270.1
MtSCPYI-2	*Medicago truncatula*	Medtr7g085040.1
MtSCPYI-3	*Medicago truncatula*	Medtr7g006690.1
MtSCPYI-4	*Medicago truncatula*	Medtr7g084970.1
MtSCPYI-5	*Medicago truncatula*	Medtr7g104460.1

MtSCPYI-6	*Medicago truncatula*	Medtr7g006630.1
MtSCPYI-7	*Medicago truncatula*	Medtr8g106840.1
MtSCPYI-8	*Medicago truncatula*	AC146807_25.1
VvSCPYI-1	*Vitis vinifera*	GSVIVP00007370001
VvSCPYI-2	*Vitis vinifera*	GSVIVP00012870001
VvSCPYI-3	*Vitis vinifera*	GSVIVP00013086001
VvSCPYI-4	*Vitis vinifera*	GSVIVP00017800001
VvSCPYI-5	*Vitis vinifera*	GSVIVP00021628001
VvSCPYI-6	*Vitis vinifera*	GSVIVP00037374001
OsSCPYI-1	*Oryza sativa*	Os01g02120.1
OsSCPYI-2	*Oryza sativa*	Os01g11940.1
OsSCPYI-3	*Oryza sativa*	Os02g13830.1
OsSCPYI-4	*Oryza sativa*	Os02g32950.1
OsSCPYI-5	*Oryza sativa*	Os04g33570.1
OsSCPYI-6	*Oryza sativa*	Os04g41130.1
OsSCPYI-7	*Oryza sativa*	Os06g06300.1
OsSCPYI-8	*Oryza sativa*	Os06g06320.1
OsSCPYI-9	*Oryza sativa*	Os06g30370.1
OsSCPYI-10	*Oryza sativa*	Os06g35940.1
OsSCPYI-11	*Oryza sativa*	Os09g33850.1
OsSCPYI-12	*Oryza sativa*	Os05g44180.1
OsSCPYI-13	*Oryza sativa*	Os12g05590.1
OsSCPYI-14	*Oryza sativa*	Os12g13030.1
OsSCPYI-15	*Oryza sativa*	Os11g05470.1
OsSCPYI-16	*Oryza sativa*	Os11g18870.1
OsSCPYI-17	*Oryza sativa*	Os02g39064.1
SbSCPYI-1	*Sorghum bicolor*	Sb0010s003120.1
SbSCPYI-2	*Sorghum bicolor*	Sb02g029725.1
SbSCPYI-3	*Sorghum bicolor*	Sb03g001700.1
SbSCPYI-4	*Sorghum bicolor*	Sb03g008270.1
SbSCPYI-5	*Sorghum bicolor*	Sb03g034580.1
SbSCPYI-6	*Sorghum bicolor*	Sb04g008320.1
SbSCPYI-7	*Sorghum bicolor*	Sb04g021650.1
SbSCPYI-8	*Sorghum bicolor*	Sb04g025210.1
SbSCPYI-9	*Sorghum bicolor*	Sb05g003200.1
SbSCPYI-10	*Sorghum bicolor*	Sb06g012260.1
SbSCPYI-11	*Sorghum bicolor*	Sb06g015490.1
SbSCPYI-12	*Sorghum bicolor*	Sb06g020850.1
SbSCPYI-13	*Sorghum bicolor*	Sb08g003210.1
SbSCPYI-14	*Sorghum bicolor*	Sb08g008180.1
SbSCPYI-15	*Sorghum bicolor*	Sb10g003940.1
SbSCPYI-16	*Sorghum bicolor*	Sb10g013070.1
SbSCPYI-17	*Sorghum bicolor*	Sb10g021790.1
SmSCPYI-1	*Selaginella moellendorffii*	416612
SmSCPYI-2	*Selaginella moellendorffii*	411506
SmSCPYI-3	*Selaginella moellendorffii*	235975
SmSCPYI-4	*Selaginella moellendorffii*	166207
SmSCPYI-5	*Selaginella moellendorffii*	413641

SmSCPYI-6	*Selaginella moellendorffii*	413642
SmSCPYI-7	*Selaginella moellendorffii*	413646
SmSCPYI-8	*Selaginella moellendorffii*	413647
SmSCPYI-9	*Selaginella moellendorffii*	413648
SmSCPYI-10	*Selaginella moellendorffii*	413667
SmSCPYI-11	*Selaginella moellendorffii*	143762
SmSCPYI-12	*Selaginella moellendorffii*	17528
SmSCPYI-13	*Selaginella moellendorffii*	416299
SmSCPYI-14	*Selaginella moellendorffii*	416302
SmSCPYI-15	*Selaginella moellendorffii*	416303
SmSCPYI-16	*Selaginella moellendorffii*	416312
SmSCPYI-17	*Selaginella moellendorffii*	416313
SmSCPYI-18	*Selaginella moellendorffii*	445941
PpSCPYI-1	*Physcomitrella patens*	Pp1s111_36V6.1
PpSCPYI-2	*Physcomitrella patens*	Pp1s441_20V6.1
PpSCPYI-3	*Physcomitrella patens*	Pp1s243_37V6.1
PpSCPYI-4	*Physcomitrella patens*	Pp1s34_16V6.1
PpSCPYI-5	*Physcomitrella patens*	Pp1s34_16V6.2
PpSCPYI-6	*Physcomitrella patens*	Pp1s68_5V6.1
CsSCPYI-1	*Coccomyxa subellipsoidea*	estExt_fgenesh1_pg.C_20153 46092
MpSCPYI-1	*Micromonas pusilla*	CCMP1545.EuGene.0000070162 59313
MpSCPYI-2	*Micromonas pusilla*	CCMP1545.EuGene.0000050752 58447
